# Supplementary material for: Evolution and origin of sliding clamp in bacteria, archaea and eukarya
Source: PLoS One. 2021 Aug 11;16(8):e0241093. doi: 10.1371/journal.pone.0241093 (PMC8357120; doi:10.1371/journal.pone.0241093)
Supplement: S1 Table — (PDF) [file pone.0241093.s001.pdf]

S1 Table. List of species from three domains of life used in this work

List of archeal species used in this work

S. No.	TaxID	Species	Phylum	Class
1	666510	*Acidilobus saccharovorans 345-15 (taxid:666510)*	Crenarchaeota	Desulfurococcales
2	272557	*Aeropyrum pernix K1* (taxid:272557)	Crenarchaeota	Desulfurococcales
3	490899	Desulfurococcus kamchatkensis 1221n (taxid:490899)	Crenarchaeota	Desulfurococcales
4	415426	Hyperthermus butylicus DSM 5456 (taxid:415426)	Crenarchaeota	Desulfurococcales
5	453591	Ignicoccus hospitalis KIN4/I (taxid:453591)	Crenarchaeota	Desulfurococcales
6	583356	Ignisphaera aggregans DSM 17230 (taxid:583356)	Crenarchaeota	Desulfurococcales
7	694429	Pyrolobus fumarii 1A (taxid:694429)	Crenarchaeota	Desulfurococcales
8	591019	Staphylothermus hellenicus DSM 12710 (taxid:591019)	Crenarchaeota	Desulfurococcales
9	6333148	Thermosphaera aggregans DSM 11486 (taxid:633148)	Crenarchaeota	Desulfurococcales
10	933801	Acidianus hospitalis W1 (taxid:933801)	Crenarchaeota	Sulfolobales
11	399549	Metallosphaera sedula DSM 5348 (taxid:399549)	Crenarchaeota	Sulfolobales
12	671065	Metallosphaera yellowstonensis MK1 (taxid:671065)	Crenarchaeota	Sulfolobales
13	330779	Sulfolobus acidocaldarius DSM 639 (taxid:330779)	Crenarchaeota	Sulfolobales
14	273057	Saccharolobus solfataricus P2 (taxid:273057)	Crenarchaeota	Sulfolobales
15	273063	Sulfurisphaera tokodaii str. 7 (taxid:273063)	Crenarchaeota	Sulfolobales
16	397948	Caldivirga maquilingensis IC-167 (taxid:397948)	Crenarchaeota	Thermoproteales
17	178306	Pyrobaculum aerophilum str. IM2 (taxid:178306)	Crenarchaeota	Thermoproteales
18	340102	Pyrobaculum arsenaticum DSM 13514 (taxid:340102)	Crenarchaeota	Thermoproteales
19	384616	Pyrobaculum islandicum DSM 4184 (taxid:384616)	Crenarchaeota	Thermoproteales
20	368408	Thermofilum pendens Hrk 5 (taxid:368408)	Crenarchaeota	Thermoproteales
21	768679	Thermoproteus tenax Kra 1 (taxid:768679)	Crenarchaeota	Thermoproteales
22	572478	Vulcanisaeta distributa DSM 14429 (taxid:572478)	Crenarchaeota	Thermoproteales
23	374847	Candidatus Korarchaeum cryptofilum OPF8 (taxid:374847)	Korachaeota	
24	436308	Nitrosopumilus maritimus SCM1 (taxid:436308)	Thaumarchaeota	Nitrosopumilales
25	886738	Candidatus Nitrosoarchaeum limnia SFB1 (taxid:886738)	Thaumarchaeota	Nitrosopumilales
26	1001994	Candidatus Nitrosoarchaeum koreensis MY1 (taxid:1001994)	Thaumarchaeota	Nitrosopumilales
27	859350	Candidatus Nitrosopumilus salaria BD31 (taxid:859350)	Thaumarchaeota	Nitrosopumilales
28	797209	Haladaptatus paucihalophilus DX253 (taxid:797209)	Euryarchaeota	Halobacteriales

29	469382	Halogeometricum borinquense DSM 11551 (taxid:469382)	Euryarchaeota	Halobacteriales
30	519442	Halorhabdus utahensis DSM 12940 (taxid:519442)	Euryarchaeota	Halobacteriales
31	410358	Methanocorpusculum labreanum Z (taxid:410358)	Euryarchaeota	Methanomicrobiales
32	368407	Methanoculleus marisnigri JR1 (taxid:368407)	Euryarchaeota	Methanomicrobiales
33	937775	Methanoplanus limicola DSM 2279 (taxid:937775)	Euryarchaeota	Methanomicrobiales
34	521011	Methanosphaerula palustris E1-9c (taxid:521011)	Euryarchaeota	Methanomicrobiales
35	323259	Methanospirillum hungatei JF-1 (taxid:323259)	Euryarchaeota	Methanomicrobiales
36	351160	Methanocella arvoryzae MRE50 (taxid:351160)	Euryarchaeota	Methanomicrobia
37	1041930	Methanocella conradii HZ254 (taxid:1041930)	Euryarchaeota	Methanomicrobia
38	304371	Methanocella paludicola SANAE (taxid:304371)	Euryarchaeota	Methanomicrobia
39	259564	Methanococcoides burtonii DSM 6242 (taxid:259564)	Euryarchaeota	Methanosarcinales
40	1110509	Methanosaeta harundinacea 6Ac (taxid:1110509)	Euryarchaeota	Methanosarcinales
41	349307	Methanothrix thermoacetophila PT (taxid:349307)	Euryarchaeota	Methanosarcinales
42	679901	Methanosalsum zhilinae DSM 4017 (taxid:679901)	Euryarchaeota	Methanosarcinales
43	192952	Methanosarcina mazei Go1 (taxid:192952)	Euryarchaeota	Methanosarcinales
44	224325	Archaeoglobus fulgidus DSM 4304 (taxid:224325)	Euryarchaeota	Archaeoglobales
45	572546	Archaeoglobus profundus DSM 5631 (taxid:572546)	Euryarchaeota	Archaeoglobales
46	693661	Archaeoglobus veneficus SNP6 (taxid:693661)	Euryarchaeota	Archaeoglobales
47	589924	Ferroglobus placidus DSM 10642 (taxid:589924)	Euryarchaeota	Archaeoglobales
48	439481	Aciduliprofundum boonei T469 (taxid:439481)	Euryarchaeota	Thermoplasmatales
49	333146	Ferroplasma acidarmanus fer1 (taxid:333146)	Euryarchaeota	Thermoplasmatales
50	263820	Picrophilus torridus DSM 9790 (taxid:263820)	Euryarchaeota	Thermoplasmatales
51	273075	Thermoplasma acidophilum DSM 1728 (taxid:273075)	Euryarchaeota	Thermoplasmatales
52	273116	Thermoplasma volcanium GSS1 (taxid:273116)	Euryarchaeota	Thermoplasmatales
53	573063	Methanocaldococcus infernus ME (taxid:573063)	Euryarchaeota	Methanococcales
54	243232	Methanocaldococcus jannaschii DSM 2661 (taxid:243232)	Euryarchaeota	Methanococcales
55	419665	Methanococcus aeolicus Nankai-3 (taxid:419665)	Euryarchaeota	Methanococcales
56	406327	Methanococcus vannielii SB (taxid:406327)	Euryarchaeota	Methanococcales
57	880724	Methanotorris igneus Kol 5 (taxid:880724)	Euryarchaeota	Methanococcales
58	420247	Methanobrevibacter smithii ATCC 35061 (taxid:420247)	Euryarchaeota	Methanobacteriales

59	339860	Methanosphaera stadtmanae DSM 3091 (taxid:339860)	Euryarchaeota	Methanobacteriales
60	187420	Methanothermobacter thermautotrophicus str. Delta H (taxid:187420)	Euryarchaeota	Methanobacteriales
61	523846	Methanothermus fervidus DSM 2088 (taxid:523846)	Euryarchaeota	Methanobacteriales
62	190192	Methanopyrus kandleri AV19 (taxid:190192)	Euryarchaeota	Methanopyrales
63	272844	Pyrococcus abyssi GE5 (taxid:272844)	Euryarchaeota	Thermococcales
64	529709	Pyrococcus yayanosii CH1 (taxid:529709)	Euryarchaeota	Thermococcales
65	391623	Thermococcus barophilus MP (taxid:391623)	Euryarchaeota	Thermococcales
66	593117	Thermococcus gammatolerans EJ3 (taxid:593117)	Euryarchaeota	Thermococcales
67	523849	Thermococcus litoralis DSM 5473 (taxid:523849)	Euryarchaeota	Thermococcales
68	425595	Candidatus Micrarchaeum acidiphilum ARMAN-2 (taxid:425595)	Euryarchaeota	ARMAN
69	662760	Candidatus Parvarchaeum acidiphilum ARMAN-4 (taxid:662760)	Euryarchaeota	ARMAN
70	662762	Candidatus Parvarchaeum acidophilus ARMAN-5 (taxid:662762)	Euryarchaeota	ARMAN
71	228908	Nanoarchaeum equitans Kin4-M (taxid:228908)	Euryarchaeota	Nanoarchaeota
72	889948	Candidatus Nanosalina sp. J07AB43 (taxid:889948)	Euryarchaeota	Nanohaloarchaea
73	889962	Candidatus Nanosalinarum sp. J07AB56 (taxid:889962)	Euryarchaeota	Nanohaloarchaea
74	274854	uncultured marine group II euryarchaeote (taxid:274854)	Euryarchaeota	Unclassified

List of Bacterial Species used in this work

S. No.	TaxID	Species	Phylum	Class
1	1133849	Nocardia brasiliensis ATCC 700358 (taxid:1133849)	Actinobacteria	Actinobacteria
2	101510	Rhodococcus jostii RHA1 (taxid:101510)	Actinobacteria	Actinobacteria
3	463191	Streptomyces sviceus ATCC 29083 (taxid:463191)	Actinobacteria	Actinobacteria
4	224324	Aquifex aeolicus VF5 (taxid:224324)	Aquificae	Aquificae
5	608538	Hydrogenobacter thermophilus TK-6 (taxid:608538)	Aquificae	Aquificae
6	123214	Persephonella marina EX-H1 (taxid:123214)	Aquificae	Aquificae
7	485918	Chitinophaga pinensis DSM 2588 (taxid:485918)	Bacteriodetes/Chlorobi	Bacteriodetes
8	760192	Haliscomenobacter hydrossis DSM 1100 (taxid:760192)	Bacteriodetes/Chlorobi	Bacteriodetes
9	290317	Chlorobium phaeobacteroides DSM 266 (taxid:290317)	Bacteriodetes/Chlorobi	Chlorobi
10	329726	Acaryochloris marina MBIC11017 (taxid:329726)	Cyanobacteria	Chroococcales
11	251221	Gloeobacter violaceus PCC 7421 (taxid:251221)	Cyanobacteria	Gloeobacteria
12	179408	Oscillatoria nigro-viridis PCC 7112 (taxid:179408)	Cyanobacteria	Oscillatoriales
13	926569	Anaerolinea thermophila UNI-1 (taxid:926569)	Chloroflexi	Anaerolineae
14	926550	Caldilinea aerophila DSM 14535 = NBRC 104270 (taxid:926550)	Chloroflexi	Caldilineae
15	552811	Dehalogenimonas lykanthroporepellens BL-DC-9 (taxid:552811)	Chloroflexi	Dehalococcoidetes
16	511051	Caldisericum exile AZM16c01 (taxid:511051)	Caldiserica	Caldisericia
17	765952	Parachlamydia acanthamoebae UV-7 (taxid:765952)	Chlamydiae/Verrucomicr	Chlamydiae
18	331113	Simkania negevensis Z (taxid:331113)	Chlamydiae/Verrucomicr	Chlamydiae
19	452637	Opitutus terrae PB90-1 (taxid:452637)	Chlamydiae/Verrucomicr	Verrucomicrobia
20	653733	Desulfurispirillum indicum S5 (taxid:653733)	Chrysiogenetes	Chrysiogenetes
21	768670	Calditerrivibrio nitroreducens DSM 19672 (taxid:768670)	Deferribacteres	Deferribacteres
22	639282	Deferribacter desulfuricans SSM1 (taxid:639282)	Deferribacteres	Deferribacteres
23	717231	Flexistipes sinusarabici DSM 4947 (taxid:717231)	Deferribacteres	Deferribacteres
24	526227	Meiothermus silvanus DSM 9946 (taxid:526227)	Deinococcus-Thermus	Deinococci

25	743525	Thermus scotoductus SA-01 (taxid:743525)	Deinococcus-Thermus	Deinococci
26	649638	Truepera radiovictrix DSM 17093 (taxid:649638)	Deinococcus-Thermus	Deinococci
27	309799	Dictyoglomus thermophilum H-6-12 (taxid:309799)	Dictyoglomi	Dictyoglomia
28	515635	Dictyoglomus turgidum DSM 6724 (taxid:515635)	Dictyoglomi	Dictyoglomia
29	445932	Elusimicrobium minutum Pei191 (taxid:445932)	Elusimicrobia	Elusimicrobia
30	471821	uncultured Termite group 1 bacterium phylotype Rs-D17 (taxid:471821)	Elusimicrobia	Env. Samples
31	240015	Acidobacterium capsulatum ATCC 51196 (taxid:240015)	Acidobacteria	Acidobacteria
32	234267	Candidatus Solibacter usitatus Ellin6076 (taxid:234267)	Acidobacteria	Acidobacteria
33	926566	Terriglobus roseus DSM 18391 (taxid:926566)	Acidobacteria	Acidobacteria
34	1195464	Bacillus thuringiensis MC28 (taxid:1195464)	Firmicutes	Bacilli
35	573061	Clostridium cellulovorans 743B (taxid:573061)	Firmicutes	Clostridia
36	768706	Desulfosporosinus orientis DSM 765 (taxid:768706)	Firmicutes	Clostridia
37	190304	Fusobacterium nucleatum subsp. nucleatum ATCC 25586 (taxid:190304)	Fusobacteria	Fusobacteriia
38	572544	Ilyobacter polytropus DSM 2926 (taxid:572544)	Fusobacteria	Fusobacteriia
39	526218	Sebaldella termitidis ATCC 33386 (taxid:526218)	Fusobacteria	Fusobacteriia
40	379066	Gemmatimonas aurantiaca T-27 (taxid:379066)	Gemmatimonadetes	Gemmatimonadetes
41	330214	Candidatus Nitrospira defluvii (taxid:330214)	Nitrospirae	Nitrospira
42	1048260	Leptospirillum ferriphilum ML-04 (taxid:1048260)	Nitrospirae	Nitrospira
43	1162668	Leptospirillum ferrooxidans C2-3 (taxid:1162668)	Nitrospirae	Nitrospira
44	1142394	Phycisphaera mikurensis NBRC 102666 (taxid:1142394)	Planctomycetes	Phycisphaerae
45	756272	Planctomyces brasiliensis ATCC 49424 (taxid:756272)	Planctomycetes	Planctomycetia
46	886293	Singulisphaera acidiphila DSM 18658 (taxid:886293)	Planctomycetes	Planctomycetia
47	311403	Agrobacterium radiobacter K84 (taxid:311403)	Proteobacteria	Alphaproteobacteria
48	137722	Azospirillum sp. B510 (taxid:137722)	Proteobacteria	Alphaproteobacteria
49	216596	Rhizobium leguminosarum bv. viciae 3841 (taxid:216596)	Proteobacteria	Alphaproteobacteria
50	762376	Achromobacter xylosoxidans A8 (taxid:762376)	Proteobacteria	Betaproteobacteria
51	266265	Burkholderia xenovorans LB400 (taxid:266265)	Proteobacteria	Betaproteobacteria

52	381666	Ralstonia eutropha H16 (taxid:381666)	Proteobacteria	Betaproteobacteria
53	572480	Arcobacter nitrofigilis DSM 7299 (taxid:572480)	Proteobacteria	Delta/Epsilon Sub-Divisions
54	706587	Desulfomonile tiedjei DSM 6799 (taxid:706587)	Proteobacteria	Delta/Epsilon Sub-Divisions
55	502025	Haliangium ochraceum DSM 14365 (taxid:502025)	Proteobacteria	Delta/Epsilon Sub-Divisions
56	349521	Hahella chejuensis KCTC 2396 (taxid:349521)	Proteobacteria	Gammaproteobacteria
57	1191061	Klebsiella oxytoca E718 (taxid:1191061)	Proteobacteria	Gammaproteobacteria
58	338187	Vibrio [harveyi] ATCC BAA-1116 (taxid:338187)	Proteobacteria	Gammaproteobacteria
59	573825	Leptospira interrogans serovar Lai str. IPAV (taxid:573825)	Spirochaetes	Spirochaetia
60	158190	Sphaerochaeta pleomorpha str. Grapes (taxid:158190)	Spirochaetes	Spirochaetia
61	869212	Turneriella parva DSM 21527 (taxid:869212)	Spirochaetes	Spirochaetia
62	572547	Aminobacterium colombiense DSM 12261 (taxid:572547)	Synergistetes	Synergistia
63	584708	Aminomonas paucivorans DSM 12260 (taxid:584708)	Synergistetes	Synergistia
64	891968	Anaerobaculum mobile DSM 13181 (taxid:891968)	Synergistetes	Synergistia
65	441768	Acholeplasma laidlawii PG-8A (taxid:441768)	Tenericutes	Mollicutes
66	322098	Aster yellows witches'-broom phytoplasma AYWB (taxid:322098)	Tenericutes	Mollicutes
67	265311	Mesoplasma florum L1 (taxid:265311)	Tenericutes	Mollicutes
68	667014	Thermodesulfatator indicus DSM 15286 (taxid:667014)	Thermodesulfobacteria	Thermodesulfobacteria
69	795359	Thermodesulfobacterium sp. OBP45 (taxid:795359)	Thermodesulfobacteria	Thermodesulfobacteria
70	521045	Kosmotoga olearia TBF 19.5.1 (taxid:521045)	Thermotogae	Thermotogae
71	443254	Marinitoga piezophila KA3 (taxid:443254)	Thermotogae	Thermotogae
72	126740	Thermotoga sp. RQ2 (taxid:126740)	Thermotogae	Thermotogae
73	880073	Caldithrix abyssi DSM 13497 (taxid:880073)	Unclassified	Caldithrix
74	671143	Candidatus Methylomirabilis oxyfera (taxid:671143)	Unclassified	Candidate Division NC 10
75	525904	Thermobaculum terrenum ATCC BAA-798 (taxid:525904)	Unclassified	Thermobaculum

List of eukaryal species used in this work

S. No.	TaxID	Species	Kingdom
1	13616	Monodelphis domestica (taxid:13616)	Animalia
2	10090	Mus musculus (taxid:10090)	Animalia
3	9606	Homo sapiens (taxid:9606)	Animalia
4	7739	Branchiostoma floridae (taxid:7739)	Animalia
5	6087	Hydra vulgaris (taxid:6087)	Animalia
6	7668	Strongylocentrotus purpuratus (taxid:7668)	Animalia
7	242159	Ostreococcus lucimarinus (taxid:242159)	Archaeaplastida
8	38833	Micromonas pusilla (taxid:38833)	Archaeaplastida
9	3067	Volvox carteri (taxid:3067)	Archaeaplastida
10	3702	Arabidopsis thaliana (taxid:3702)	Archaeaplastida
11	45157	Cyanidioschyzon merolae (taxid:45157)	Archaeaplastida
12	4896	Schizosaccharomyces pombe (taxid:4896)	Fungi
13	5141	Neurospora crassa (taxid:5141)	Fungi
14	29883	Laccaria bicolor (taxid:29883)	Fungi
15	5297	Puccinia graminis (taxid:5297)	Fungi
16	5270	Ustilago maydis (taxid:5270)	Fungi
17	76773	Malassezia globosa (taxid:76773)	Fungi
18	5722	Trichomonas vaginalis (taxid:5722)	Eozoa
19	5741	Giardia lamblia (taxid:5741)	Eozoa
20	5693	Trypanosoma cruzi (taxid:5693)	Eozoa
21	5691	Trypanosoma brucei (taxid:5691)	Eozoa
22	5664	Leishmania major (taxid:5664)	Eozoa
23	5671	Leishmania (Leishmania) infantum (taxid:5671)	Eozoa
24	5949	Stylonychia lemnae (taxid:5949)	Ciliophora
25	94289	Oxytricha trifallax (taxid:94289)	Ciliophora
26	5911	Tetrahymena thermophila (taxid:5911)	Ciliophora
27	5888	Paramecium tetraurelia (taxid:5888)	Ciliophora
28	5755	Acanthamoeba castellani (taxid:5755)	Amoebozoa
29	5759	Entamoeba histolytica (taxid:5759)	Amoebozoa

30	33085	Entamoeba invadens (taxid:33085)	Amoebozoa
31	44689	Dictyostelium discoideum (taxid:44689)	Amoebozoa
