# Supplementary material for: Evolution and origin of sliding clamp in bacteria, archaea and eukarya
Source: PLoS One. 2021 Aug 11;16(8):e0241093. doi: 10.1371/journal.pone.0241093 (PMC8357120; doi:10.1371/journal.pone.0241093)
Supplement: S1 Data — (ZIP) [file pone.0241093.s008.zip › New folder/NCBI Blast_Homo sapiens.html]

NCBI Blast:Homo sapiens


 


- NCBI Home
- Sign in to NCBI
- Skip to Main Content
- Skip to Navigation
- About NCBI Accesskeys

National Institutes of Health

U.S. National Library of Medicine

National Center for Biotechnology Information

NCBI homepage

Log in


Show account info

Close

#### Account

Logged in as:  
**username**

- Dashboard (My NCBI)
- Publications (My Bibliography)
- Account settings
- Log out

**COVID-19 is an emerging, rapidly evolving situation.**

Get the latest public health information from CDC: https://www.coronavirus.gov

Get the latest research information from NIH: https://www.nih.gov/coronavirus

Find NCBI SARS-CoV-2 literature, sequence, and clinical content: https://www.ncbi.nlm.nih.gov/sars-cov-2/

BLAST ® » blastp suite »

# results for RID-ZM263WRH013

- Home
- Recent Results
- Saved Strategies
- Help


- Edit Search
- Save Search
- Search Summary

  Search Parameters

  | Search parameter name | Search parameter value |
  | --- | --- |
  | Program | blastp |
  | Word size | 3 |
  | Expect value | 0.05 |
  | Hitlist size | 500 |
  | Gapcosts | 11,1 |
  | Matrix | BLOSUM62 |
  | Filter string | F |
  | Genetic Code | 1 |
  | Window Size | 40 |
  | Threshold | 11 |
  | Composition-based stats | 1 |

  Database

  | Database parameter name | Database parameter value |
  | --- | --- |
  | Posted date | Jan 7, 2021 12:42 AM |
  | Number of letters | 88,505,061,722 |
  | Number of sequences | 265,781,661 |
  | Entrez query | Includes: Bacteria (taxid:2)  Excludes:  None |

  Karlin-Altschul statistics

  | Params | Ungapped | Gapped |
  | --- | --- | --- |
  | Lambda | 0.31529 | 0.267 |
  | K | 0.131308 | 0.0475617 |
  | H | 0.364035 | 0.14 |
  | Alpha | 0.7916 | 1.9 |
  | Alpha\_v | 4.96466 | 42.6028 |
  | Sigma |  | 43.6362 |

  Results Statistics

  | Results Statistics parameter name | Results Statistics parameter value |
  | --- | --- |

- How to read this report?
- BLAST Help Videos
- Back to Traditional Results Page

Your search is limited to records that include: Bacteria (taxid:2)

- Full Entrez Query

  txid2 [ORGN]


Job Title
:   Homo sapiens
    ...

    Homo sapiens

RID
:   ZM263WRH013
    Search expires on 01-11 02:49 am

    - Download All
      - Text
      - XML
      - ASN.1
      - JSON Seq-align
      - Hit Table(text)
      - Hit Table(csv)
      - Multiple-file XML2
      - Single-file XML2
      - Multiple-file JSON
      - Single-file JSON
      - SAM
      - PSSM

Results for
:   lcl|Query\_61499 Homo sapiens(261aa)

Program
:   PSI-BLAST Iteration 2

    - Citation

      Reference 

      Stephen F. Altschul, Thomas L. Madden, Alejandro A. Schäffer, Jinghui Zhang, Zheng Zhang, Webb Miller, and David J. Lipman (1997), "Gapped BLAST and PSI-BLAST: a new generation of protein database search programs", Nucleic Acids Res. 25:3389-3402.

      Reference - composition-based statistics starting in round 2

      Alejandro A. Schäffer, L. Aravind, Thomas L. Madden, Sergei Shavirin, John L. Spouge, Yuri I. Wolf, Eugene V. Koonin, and Stephen F. Altschul (2001), "Improving the accuracy of PSI-BLAST protein database searches with composition-based statistics and other refinements", Nucleic Acids Res. 29:2994-3005.

Database
:   nr

    - See details

      Title:All non-redundant GenBank CDS translations+PDB+SwissProt+PIR+PRF excluding environmental samples from WGS projects  
      Molecule Type:Protein  
      Update date:2021/01/08  
      Number of sequences:339145745

Query ID
:   lcl|Query\_61499
    lcl|Query\_61499

Description
:   Homo sapiens
    ...

    Homo sapiens

Molecule type
:   amino acid

Query Length
:   261

Other reports
:   Distance tree of results
    Multiple alignment
    MSA viewer
     Help

    Reports are generated on using all sequences producing significant alignments. To generate reports on a subset of sequences, use the report links in the Descriptions tab while selecting specific sequences.

## Filter Results

Organism only top 20 will appear


exclude

Add organism


---

Percent Identity from

Percent Identity to

E value from

E value to

Query Coverage from

Query Coverage to

PSI-BLAST incl. threshold

Filter Reset

## Run PSI-Blast iteration 3

Number of sequences


Run

- Descriptions

  ### Sequences producing significant alignments

  - Download
    - FASTA (complete sequence)
    - FASTA (aligned sequences)
    - GenBank (complete sequence)
    - Hit Table (text)
    - Hit Table (CSV)
    - Text
    - Descriptions Table (CSV)
    - XML
    - ASN.1
  - New

    Select columns
    - Description
    - Scientific Name
    - Common Name
    - Taxid
    - Max Score
    - Total Score
    - Query Coverage
    - E value
    - Percent Identity
    - Acc. Len
    - Accession
    - Restore defaults
  - Show

    10
    50
    100
    250
    500
  - Help

    Subject sequences can be removed or added from within the Descriptions tab and the selections will carry through to the other tabbed views.
    Use the formats in Download to save data for selected sequences. Manage Columns adds and subtracts data columns from the Descriptions table.
    Use the click outs to see the selected results in
    GenPept
    , Graphical Sequence Viewer
    , BLAST Tree View
    , COBALT multiple sequence alignment
    .

  - 226 sequences selected
  - sequences newly added this iteration
     Help

    sequences scoring below threshold on previous iteration
  - GenPept
  - Graphics
  - Distance tree of results
  - Multiple alignment
  - New

    MSA Viewer

  , Reading indexes 1-5, displaying indexes 1-5


  Load next setPrevious Match


  -newly added

  -used in PSSM

  Sequences with E-value BETTER than threshold 

  - select all
  - 210 sequences selected
  - Skip to the first new sequence
  - PSI-BLAST iteration 2

  Sequences producing significant alignments with E-value BETTER than threshold

  | Select for downloading or viewing reports | Description | Scientific Name | Common Name | Taxid | Max Score | Total Score | Query Cover | E value | Per. Ident | Acc. Len | Accession | Select for PSI blast | Used to build PSSM | Newly added |
  | --- | --- | --- | --- | --- | --- | --- | --- | --- | --- | --- | --- | --- | --- | --- |
  | 1Select seq gb|NBO72319.1| | proliferating cell nuclear antigen (pcna) [bacterium] | bacterium | NA | 1869227 | 283 | 283 | 96% | 1e-92 | 30.71% | 272 | NBO72319.1(scored below threshold on previous iteration) | Select seq NBO72319.1 for PSI blast |  |  |
  | 2Select seq gb|NBO60550.1| | proliferating cell nuclear antigen (pcna) [Flavobacteriia bacterium] | Flavobacteriia bacterium | NA | 2044941 | 282 | 282 | 96% | 6e-92 | 32.03% | 272 | NBO60550.1(scored below threshold on previous iteration) | Select seq NBO60550.1 for PSI blast |  |  |
  | 3Select seq gb|NBO70988.1| | proliferating cell nuclear antigen (pcna) [bacterium] | bacterium | NA | 1869227 | 281 | 281 | 96% | 1e-91 | 32.03% | 276 | NBO70988.1(scored below threshold on previous iteration) | Select seq NBO70988.1 for PSI blast |  |  |
  | 4Select seq gb|NDB83927.1| | proliferating cell nuclear antigen (pcna) [Alphaproteobacteria bacterium] | Alphaproteobacteria bacterium | NA | 1913988 | 281 | 281 | 96% | 1e-91 | 30.31% | 273 | NDB83927.1(scored below threshold on previous iteration) | Select seq NDB83927.1 for PSI blast |  |  |
  | 5Select seq gb|NBO72693.1| | proliferating cell nuclear antigen (pcna) [bacterium] | bacterium | NA | 1869227 | 281 | 281 | 96% | 1e-91 | 29.13% | 273 | NBO72693.1(scored below threshold on previous iteration) | Select seq NBO72693.1 for PSI blast |  |  |
  | 6Select seq ref|WP\_162815250.1| | proliferating cell nuclear antigen (pcna) [Microbacterium arborescens] | Microbacterium arborescens | NA | 33883 | 279 | 279 | 95% | 4e-91 | 67.87% | 249 | WP\_162815250.1(scored below threshold on previous iteration) | Select seq WP\_162815250.1 for PSI blast |  |  |
  | 7Select seq gb|NBV77139.1| | proliferating cell nuclear antigen (pcna) [bacterium] | bacterium | NA | 1869227 | 279 | 279 | 96% | 7e-91 | 32.03% | 276 | NBV77139.1(scored below threshold on previous iteration) | Select seq NBV77139.1 for PSI blast |  |  |
  | 8Select seq gb|NBU17043.1| | proliferating cell nuclear antigen (pcna) [Actinobacteria bacterium] | Actinobacteria bacterium | NA | 1883427 | 270 | 270 | 97% | 1e-87 | 29.02% | 275 | NBU17043.1(scored below threshold on previous iteration) | Select seq NBU17043.1 for PSI blast |  |  |
  | 9Select seq gb|NDE16198.1| | proliferating cell nuclear antigen (pcna) [bacterium] | bacterium | NA | 1869227 | 268 | 268 | 97% | 1e-86 | 30.08% | 268 | NDE16198.1(scored below threshold on previous iteration) | Select seq NDE16198.1 for PSI blast |  |  |
  | 10Select seq gb|MTA40656.1| | proliferating cell nuclear antigen (pcna) [Actinobacteria bacterium] | Actinobacteria bacterium | NA | 1883427 | 267 | 267 | 96% | 3e-86 | 27.56% | 279 | MTA40656.1(scored below threshold on previous iteration) | Select seq MTA40656.1 for PSI blast |  |  |
  | 11Select seq gb|NBX51340.1| | proliferating cell nuclear antigen (pcna) [bacterium] | bacterium | NA | 1869227 | 266 | 266 | 98% | 6e-86 | 29.18% | 266 | NBX51340.1(scored below threshold on previous iteration) | Select seq NBX51340.1 for PSI blast |  |  |
  | 12Select seq gb|NDB83590.1| | proliferating cell nuclear antigen (pcna) [Alphaproteobacteria bacterium] | Alphaproteobacteria bacterium | NA | 1913988 | 264 | 264 | 96% | 4e-85 | 29.64% | 275 | NDB83590.1(scored below threshold on previous iteration) | Select seq NDB83590.1 for PSI blast |  |  |
  | 13Select seq gb|MBG02288.1| | proliferating cell nuclear antigen (pcna) [Acidimicrobiaceae bacterium] | Acidimicrobiaceae bacterium | NA | 2024894 | 259 | 259 | 96% | 5e-83 | 29.37% | 264 | MBG02288.1(scored below threshold on previous iteration) | Select seq MBG02288.1 for PSI blast |  |  |
  | 14Select seq gb|MBC8296603.1| | proliferating cell nuclear antigen (pcna) [Pelagibacterales bacterium] | Pelagibacterales bacterium | NA | 2026776 | 257 | 257 | 96% | 3e-82 | 28.19% | 267 | MBC8296603.1(scored below threshold on previous iteration) | Select seq MBC8296603.1 for PSI blast |  |  |
  | 15Select seq gb|NBX51392.1| | proliferating cell nuclear antigen (pcna) [bacterium] | bacterium | NA | 1869227 | 256 | 256 | 96% | 5e-82 | 29.53% | 261 | NBX51392.1(scored below threshold on previous iteration) | Select seq NBX51392.1 for PSI blast |  |  |
  | 16Select seq gb|MBE18271.1| | proliferating cell nuclear antigen (pcna) [Nitrospinae bacterium] | Nitrospinae bacterium | NA | 2026769 | 256 | 256 | 96% | 5e-82 | 30.16% | 263 | MBE18271.1(scored below threshold on previous iteration) | Select seq MBE18271.1 for PSI blast |  |  |
  | 17Select seq gb|MAD50601.1| | proliferating cell nuclear antigen (pcna) [Flavobacteriales bacterium] | Flavobacteriales bacterium | NA | 2021391 | 256 | 256 | 96% | 6e-82 | 26.34% | 272 | MAD50601.1(scored below threshold on previous iteration) | Select seq MAD50601.1 for PSI blast |  |  |
  | 18Select seq gb|MBD23483.1| | proliferating cell nuclear antigen (pcna) [Candidatus Marinimicrobia bacterium] | Candidatus Marinimicrobia bacterium | NA | 2026760 | 255 | 255 | 96% | 1e-81 | 28.57% | 262 | MBD23483.1(scored below threshold on previous iteration) | Select seq MBD23483.1 for PSI blast |  |  |
  | 19Select seq gb|MBD23789.1| | proliferating cell nuclear antigen (pcna) [Candidatus Marinimicrobia bacterium] | Candidatus Marinimicrobia bacterium | NA | 2026760 | 254 | 254 | 98% | 3e-81 | 31.40% | 260 | MBD23789.1(scored below threshold on previous iteration) | Select seq MBD23789.1 for PSI blast |  |  |
  | 20Select seq gb|OYV74989.1| | proliferating cell nuclear antigen (pcna) [Chromatiales bacterium 21-64-14] | Chromatiales bacterium 21-64-14 | NA | 1970504 | 256 | 256 | 95% | 4e-81 | 27.01% | 312 | OYV74989.1(scored below threshold on previous iteration) | Select seq OYV74989.1 for PSI blast |  |  |
  | 21Select seq gb|MAD12048.1| | proliferating cell nuclear antigen (pcna) [Flavobacteriaceae bacterium] | Flavobacteriaceae bacterium | NA | 1871037 | 254 | 254 | 96% | 1e-80 | 27.69% | 289 | MAD12048.1(scored below threshold on previous iteration) | Select seq MAD12048.1 for PSI blast |  |  |
  | 22Select seq gb|NBR60109.1| | proliferating cell nuclear antigen (pcna) [Actinobacteria bacterium] | Actinobacteria bacterium | NA | 1883427 | 253 | 253 | 96% | 2e-80 | 30.86% | 292 | NBR60109.1(scored below threshold on previous iteration) | Select seq NBR60109.1 for PSI blast |  |  |
  | 23Select seq gb|NBO53670.1| | proliferating cell nuclear antigen (pcna) [Actinobacteria bacterium] | Actinobacteria bacterium | NA | 1883427 | 253 | 253 | 96% | 2e-80 | 29.23% | 284 | NBO53670.1(scored below threshold on previous iteration) | Select seq NBO53670.1 for PSI blast |  |  |
  | 24Select seq gb|PQM59040.1| | proliferating cell nuclear antigen (pcna) [Rhodobacteraceae bacterium] | Rhodobacteraceae bacterium | NA | 1904441 | 252 | 252 | 96% | 4e-80 | 28.46% | 274 | PQM59040.1(scored below threshold on previous iteration) | Select seq PQM59040.1 for PSI blast |  |  |
  | 25Select seq gb|RPG58329.1| | proliferating cell nuclear antigen (pcna) [Flavobacteriales bacterium TMED191] | Flavobacteriales bacterium TMED191 | NA | 1986723 | 251 | 251 | 96% | 7e-80 | 26.72% | 273 | RPG58329.1(scored below threshold on previous iteration) | Select seq RPG58329.1 for PSI blast |  |  |
  | 26Select seq gb|NDG29484.1| | proliferating cell nuclear antigen (pcna) [bacterium] | bacterium | NA | 1869227 | 249 | 249 | 96% | 3e-79 | 26.27% | 261 | NDG29484.1(scored below threshold on previous iteration) | Select seq NDG29484.1 for PSI blast |  |  |
  | 27Select seq gb|NBR15457.1| | proliferating cell nuclear antigen (pcna) [Crocinitomicaceae bacterium] | Crocinitomicaceae bacterium | NA | 2026728 | 249 | 249 | 96% | 3e-79 | 27.84% | 258 | NBR15457.1(scored below threshold on previous iteration) | Select seq NBR15457.1 for PSI blast |  |  |
  | 28Select seq gb|NBR61302.1| | proliferating cell nuclear antigen (pcna) [Actinobacteria bacterium] | Actinobacteria bacterium | NA | 1883427 | 248 | 248 | 96% | 1e-78 | 26.27% | 261 | NBR61302.1(scored below threshold on previous iteration) | Select seq NBR61302.1 for PSI blast |  |  |
  | 29Select seq gb|OUU62278.1| | proliferating cell nuclear antigen (pcna) [Alphaproteobacteria bacterium TMED62] | Alphaproteobacteria bacterium TMED62 | NA | 1986642 | 246 | 246 | 96% | 1e-77 | 26.22% | 278 | OUU62278.1(scored below threshold on previous iteration) | Select seq OUU62278.1 for PSI blast |  |  |
  | 30Select seq gb|NBO71470.1| | proliferating cell nuclear antigen (pcna) [bacterium] | bacterium | NA | 1869227 | 246 | 246 | 95% | 1e-77 | 26.46% | 300 | NBO71470.1(scored below threshold on previous iteration) | Select seq NBO71470.1 for PSI blast |  |  |
  | 31Select seq gb|NBT47941.1| | proliferating cell nuclear antigen (pcna) [Actinobacteria bacterium] | Actinobacteria bacterium | NA | 1883427 | 245 | 245 | 99% | 2e-77 | 29.39% | 267 | NBT47941.1(scored below threshold on previous iteration) | Select seq NBT47941.1 for PSI blast |  |  |
  | 32Select seq gb|MAW08577.1| | proliferating cell nuclear antigen (pcna) [Halobacteriovoraceae bacterium] | Halobacteriovoraceae bacterium | NA | 2026745 | 243 | 243 | 96% | 8e-77 | 27.65% | 272 | MAW08577.1(scored below threshold on previous iteration) | Select seq MAW08577.1 for PSI blast |  |  |
  | 33Select seq gb|MAU36389.1| | proliferating cell nuclear antigen (pcna) [Flavobacteriales bacterium] | Flavobacteriales bacterium | NA | 2021391 | 243 | 243 | 96% | 1e-76 | 26.14% | 279 | MAU36389.1(scored below threshold on previous iteration) | Select seq MAU36389.1 for PSI blast |  |  |
  | 34Select seq gb|NBP59184.1| | proliferating cell nuclear antigen (pcna) [bacterium] | bacterium | NA | 1869227 | 242 | 242 | 95% | 1e-76 | 29.60% | 263 | NBP59184.1(scored below threshold on previous iteration) | Select seq NBP59184.1 for PSI blast |  |  |
  | 35Select seq gb|NDB81722.1| | proliferating cell nuclear antigen (pcna) [Alphaproteobacteria bacterium] | Alphaproteobacteria bacterium | NA | 1913988 | 242 | 242 | 96% | 2e-76 | 27.52% | 261 | NDB81722.1(scored below threshold on previous iteration) | Select seq NDB81722.1 for PSI blast |  |  |
  | 36Select seq gb|NBX48943.1| | proliferating cell nuclear antigen (pcna) [bacterium] | bacterium | NA | 1869227 | 241 | 241 | 97% | 3e-76 | 31.40% | 246 | NBX48943.1(scored below threshold on previous iteration) | Select seq NBX48943.1 for PSI blast |  |  |
  | 37Select seq gb|MAZ55206.1| | proliferating cell nuclear antigen (pcna) [Flavobacteriales bacterium] | Flavobacteriales bacterium | NA | 2021391 | 239 | 239 | 96% | 3e-75 | 25.77% | 268 | MAZ55206.1(scored below threshold on previous iteration) | Select seq MAZ55206.1 for PSI blast |  |  |
  | 38Select seq gb|NBX48718.1| | proliferating cell nuclear antigen (pcna) [bacterium] | bacterium | NA | 1869227 | 239 | 239 | 98% | 3e-75 | 26.64% | 263 | NBX48718.1(scored below threshold on previous iteration) | Select seq NBX48718.1 for PSI blast |  |  |
  | 39Select seq gb|OUU16001.1| | proliferating cell nuclear antigen (pcna) [Candidatus Endolissoclinum sp. TMED37] | Candidatus Endolissoclinum sp. TMED37 | NA | 1986638 | 238 | 238 | 96% | 4e-75 | 25.77% | 268 | OUU16001.1(scored below threshold on previous iteration) | Select seq OUU16001.1 for PSI blast |  |  |
  | 40Select seq gb|NDB84018.1| | proliferating cell nuclear antigen (pcna) [Alphaproteobacteria bacterium] | Alphaproteobacteria bacterium | NA | 1913988 | 238 | 238 | 95% | 6e-75 | 26.29% | 266 | NDB84018.1(scored below threshold on previous iteration) | Select seq NDB84018.1 for PSI blast |  |  |
  | 41Select seq gb|NDG72174.1| | proliferating cell nuclear antigen (pcna) [Proteobacteria bacterium] | Proteobacteria bacterium | NA | 1977087 | 238 | 238 | 96% | 9e-75 | 28.46% | 267 | NDG72174.1(scored below threshold on previous iteration) | Select seq NDG72174.1 for PSI blast |  |  |
  | 42Select seq gb|OUU49664.1| | proliferating cell nuclear antigen (pcna) [Candidatus Puniceispirillum sp. TMED52] | Candidatus Puniceispirillum sp. TMED52 | NA | 1986639 | 237 | 237 | 100% | 1e-74 | 50.38% | 263 | OUU49664.1(scored below threshold on previous iteration) | Select seq OUU49664.1 for PSI blast |  |  |
  | 43Select seq gb|MAT63990.1| | proliferating cell nuclear antigen (pcna) [Dehalococcoidia bacterium] | Dehalococcoidia bacterium | NA | 2026734 | 235 | 235 | 98% | 4e-74 | 27.80% | 246 | MAT63990.1(scored below threshold on previous iteration) | Select seq MAT63990.1 for PSI blast |  |  |
  | 44Select seq gb|MAE55769.1| | proliferating cell nuclear antigen (pcna) [Porticoccaceae bacterium] | Porticoccaceae bacterium | NA | 2026782 | 235 | 235 | 99% | 8e-74 | 28.03% | 253 | MAE55769.1(scored below threshold on previous iteration) | Select seq MAE55769.1 for PSI blast |  |  |
  | 45Select seq gb|NBR26233.1| | proliferating cell nuclear antigen (pcna) [Micrococcales bacterium] | Micrococcales bacterium | NA | 2026762 | 235 | 235 | 98% | 1e-73 | 37.35% | 260 | NBR26233.1(scored below threshold on previous iteration) | Select seq NBR26233.1 for PSI blast |  |  |
  | 46Select seq gb|MAR51068.1| | proliferating cell nuclear antigen (pcna) [Rhodobacteraceae bacterium] | Rhodobacteraceae bacterium | NA | 1904441 | 235 | 235 | 96% | 2e-73 | 26.47% | 277 | MAR51068.1(scored below threshold on previous iteration) | Select seq MAR51068.1 for PSI blast |  |  |
  | 47Select seq gb|NDC41159.1| | proliferating cell nuclear antigen (pcna) [Chitinophagia bacterium] | Chitinophagia bacterium | NA | 2448778 | 232 | 232 | 96% | 1e-72 | 30.31% | 259 | NDC41159.1(scored below threshold on previous iteration) | Select seq NDC41159.1 for PSI blast |  |  |
  | 48Select seq gb|MBI96915.1| | proliferating cell nuclear antigen (pcna) [bacterium] | bacterium | NA | 1869227 | 232 | 232 | 96% | 1e-72 | 26.15% | 273 | MBI96915.1(scored below threshold on previous iteration) | Select seq MBI96915.1 for PSI blast |  |  |
  | 49Select seq gb|MAT00017.1| | proliferating cell nuclear antigen (pcna) [Rhodobacteraceae bacterium] | Rhodobacteraceae bacterium | NA | 1904441 | 231 | 231 | 98% | 2e-72 | 27.41% | 246 | MAT00017.1(scored below threshold on previous iteration) | Select seq MAT00017.1 for PSI blast |  |  |
  | 50Select seq gb|NBR16174.1| | proliferating cell nuclear antigen (pcna) [Crocinitomicaceae bacterium] | Crocinitomicaceae bacterium | NA | 2026728 | 231 | 231 | 96% | 5e-72 | 28.68% | 270 | NBR16174.1(scored below threshold on previous iteration) | Select seq NBR16174.1 for PSI blast |  |  |
  | 51Select seq gb|MBP02103.1| | proliferating cell nuclear antigen (pcna) [Rhodospirillaceae bacterium] | Rhodospirillaceae bacterium | NA | 1898112 | 230 | 230 | 99% | 5e-72 | 30.38% | 254 | MBP02103.1(scored below threshold on previous iteration) | Select seq MBP02103.1 for PSI blast |  |  |
  | 52Select seq gb|RPH19578.1| | proliferating cell nuclear antigen (pcna) [Alteromonadaceae bacterium TMED7] | Alteromonadaceae bacterium TMED7 | NA | 1986650 | 230 | 230 | 99% | 7e-72 | 26.92% | 247 | RPH19578.1(scored below threshold on previous iteration) | Select seq RPH19578.1 for PSI blast |  |  |
  | 53Select seq gb|MAF45402.1| | proliferating cell nuclear antigen (pcna) [Acidimicrobiaceae bacterium] | Acidimicrobiaceae bacterium | NA | 2024894 | 229 | 229 | 98% | 1e-71 | 26.25% | 246 | MAF45402.1(scored below threshold on previous iteration) | Select seq MAF45402.1 for PSI blast |  |  |
  | 54Select seq gb|NBX51618.1| | proliferating cell nuclear antigen (pcna) [bacterium] | bacterium | NA | 1869227 | 229 | 229 | 97% | 2e-71 | 26.64% | 250 | NBX51618.1(scored below threshold on previous iteration) | Select seq NBX51618.1 for PSI blast |  |  |
  | 55Select seq gb|MAB60694.1| | proliferating cell nuclear antigen (pcna) [Verrucomicrobiales bacterium] | Verrucomicrobiales bacterium | NA | 2026801 | 228 | 228 | 98% | 5e-71 | 25.48% | 267 | MAB60694.1(scored below threshold on previous iteration) | Select seq MAB60694.1 for PSI blast |  |  |
  | 56Select seq tpg|HEN27889.1| | TPA: proliferating cell nuclear antigen (pcna) [candidate division WOR-3 bacterium] | candidate division WOR-3 bacterium | NA | 2052148 | 216 | 216 | 98% | 2e-66 | 26.36% | 251 | HEN27889.1(scored below threshold on previous iteration) | Select seq HEN27889.1 for PSI blast |  |  |
  | 57Select seq gb|MAD20416.1| | proliferating cell nuclear antigen (pcna) [Planctomycetaceae bacterium] | Planctomycetaceae bacterium | NA | 2026779 | 210 | 210 | 96% | 4e-64 | 27.95% | 262 | MAD20416.1(scored below threshold on previous iteration) | Select seq MAD20416.1 for PSI blast |  |  |
  | 58Select seq gb|MBJ75944.1| | proliferating cell nuclear antigen (pcna) [Planctomycetes bacterium] | Planctomycetes bacterium | NA | 2026780 | 210 | 210 | 96% | 6e-64 | 30.12% | 248 | MBJ75944.1(scored below threshold on previous iteration) | Select seq MBJ75944.1 for PSI blast |  |  |
  | 59Select seq gb|NBP13185.1| | proliferating cell nuclear antigen (pcna) [bacterium] | bacterium | NA | 1869227 | 210 | 210 | 96% | 1e-63 | 26.97% | 287 | NBP13185.1(scored below threshold on previous iteration) | Select seq NBP13185.1 for PSI blast |  |  |
  | 60Select seq gb|MBP37067.1| | proliferating cell nuclear antigen (pcna) [Candidatus Pelagibacter sp.] | Candidatus Pelagibacter sp. | NA | 2024849 | 208 | 208 | 98% | 2e-63 | 27.13% | 248 | MBP37067.1(scored below threshold on previous iteration) | Select seq MBP37067.1 for PSI blast |  |  |
  | 61Select seq gb|NBU33707.1| | proliferating cell nuclear antigen (pcna) [bacterium] | bacterium | NA | 1869227 | 208 | 208 | 96% | 4e-63 | 26.97% | 277 | NBU33707.1(scored below threshold on previous iteration) | Select seq NBU33707.1 for PSI blast |  |  |
  | 62Select seq gb|MBR74595.1| | proliferating cell nuclear antigen (pcna) [Dehalococcoidaceae bacterium] | Dehalococcoidaceae bacterium | NA | 2026731 | 207 | 207 | 98% | 4e-63 | 26.07% | 248 | MBR74595.1(scored below threshold on previous iteration) | Select seq MBR74595.1 for PSI blast |  |  |
  | 63Select seq gb|MBA42970.1| | proliferating cell nuclear antigen (pcna) [Magnetococcales bacterium] | Magnetococcales bacterium | NA | 2026759 | 209 | 209 | 96% | 1e-62 | 25.50% | 327 | MBA42970.1(scored below threshold on previous iteration) | Select seq MBA42970.1 for PSI blast |  |  |
  | 64Select seq gb|NDC95013.1| | proliferating cell nuclear antigen (pcna) [bacterium] | bacterium | NA | 1869227 | 207 | 207 | 96% | 2e-62 | 26.59% | 278 | NDC95013.1(scored below threshold on previous iteration) | Select seq NDC95013.1 for PSI blast |  |  |
  | 65Select seq gb|OLD30892.1| | proliferating cell nuclear antigen (pcna) [Candidatus Rokubacteria bacterium 13\_1\_40CM\_2\_70\_45] | Candidatus Rokubacteria bacterium 13\_1\_40CM\_2\_70\_45 | NA | 1805357 | 205 | 205 | 98% | 2e-62 | 25.00% | 246 | OLD30892.1(scored below threshold on previous iteration) | Select seq OLD30892.1 for PSI blast |  |  |
  | 66Select seq gb|NBP00213.1| | proliferating cell nuclear antigen (pcna) [Proteobacteria bacterium] | Proteobacteria bacterium | NA | 1977087 | 205 | 205 | 98% | 2e-61 | 26.84% | 297 | NBP00213.1(scored below threshold on previous iteration) | Select seq NBP00213.1 for PSI blast |  |  |
  | 67Select seq gb|NIW10639.1| | proliferating cell nuclear antigen (pcna) [Gammaproteobacteria bacterium] | Gammaproteobacteria bacterium | NA | 1913989 | 202 | 202 | 96% | 4e-61 | 25.59% | 246 | NIW10639.1(scored below threshold on previous iteration) | Select seq NIW10639.1 for PSI blast |  |  |
  | 68Select seq gb|OLD25330.1| | proliferating cell nuclear antigen (pcna) [Candidatus Rokubacteria bacterium 13\_1\_40CM\_2\_70\_45] | Candidatus Rokubacteria bacterium 13\_1\_40CM\_2\_70\_45 | NA | 1805357 | 202 | 202 | 98% | 4e-61 | 25.00% | 245 | OLD25330.1(scored below threshold on previous iteration) | Select seq OLD25330.1 for PSI blast |  |  |
  | 69Select seq gb|NDG27889.1| | proliferating cell nuclear antigen (pcna) [Proteobacteria bacterium] | Proteobacteria bacterium | NA | 1977087 | 201 | 201 | 99% | 7e-60 | 29.77% | 293 | NDG27889.1(scored below threshold on previous iteration) | Select seq NDG27889.1 for PSI blast |  |  |
  | 70Select seq gb|NBR60072.1| | proliferating cell nuclear antigen (pcna) [Actinobacteria bacterium] | Actinobacteria bacterium | NA | 1883427 | 200 | 200 | 99% | 1e-59 | 30.15% | 293 | NBR60072.1(scored below threshold on previous iteration) | Select seq NBR60072.1 for PSI blast |  |  |
  | 71Select seq gb|PCH85639.1| | proliferating cell nuclear antigen (pcna) [Dehalococcoidia bacterium] | Dehalococcoidia bacterium | NA | 2026734 | 198 | 198 | 98% | 1e-59 | 22.48% | 252 | PCH85639.1(scored below threshold on previous iteration) | Select seq PCH85639.1 for PSI blast |  |  |
  | 72Select seq gb|MBN37929.1| | proliferating cell nuclear antigen (pcna) [Opitutae bacterium] | Opitutae bacterium | NA | 2026771 | 196 | 196 | 100% | 3e-56 | 24.35% | 465 | MBN37929.1(scored below threshold on previous iteration) | Select seq MBN37929.1 for PSI blast |  |  |
  | 73Select seq gb|NCA73651.1| | proliferating cell nuclear antigen (pcna) [Gammaproteobacteria bacterium] | Gammaproteobacteria bacterium | NA | 1913989 | 190 | 190 | 98% | 4e-56 | 23.85% | 245 | NCA73651.1(scored below threshold on previous iteration) | Select seq NCA73651.1 for PSI blast |  |  |
  | 74Select seq gb|NTV78099.1| | proliferating cell nuclear antigen (pcna) [Clostridiales bacterium] | Clostridiales bacterium | NA | 1898207 | 188 | 188 | 98% | 2e-55 | 23.75% | 245 | NTV78099.1(scored below threshold on previous iteration) | Select seq NTV78099.1 for PSI blast |  |  |
  | 75Select seq tpg|HEC38232.1| | TPA: proliferating cell nuclear antigen (pcna) [bacterium] | bacterium | NA | 1869227 | 188 | 188 | 98% | 2e-55 | 22.14% | 266 | HEC38232.1(scored below threshold on previous iteration) | Select seq HEC38232.1 for PSI blast |  |  |
  | 76Select seq tpg|HDQ16435.1| | TPA: proliferating cell nuclear antigen (pcna) [Bacteroidetes bacterium] | Bacteroidetes bacterium | NA | 1898104 | 186 | 186 | 98% | 5e-55 | 25.00% | 245 | HDQ16435.1(scored below threshold on previous iteration) | Select seq HDQ16435.1 for PSI blast |  |  |
  | 77Select seq gb|MBI2034871.1| | proliferating cell nuclear antigen (pcna) [Candidatus Levybacteria bacterium] | Candidatus Levybacteria bacterium | NA | 2052151 | 186 | 186 | 98% | 1e-54 | 21.62% | 249 | MBI2034871.1(scored below threshold on previous iteration) | Select seq MBI2034871.1 for PSI blast |  |  |
  | 78Select seq gb|MAI67428.1| | hypothetical protein [Phycisphaerae bacterium] | Phycisphaerae bacterium | NA | 2026778 | 185 | 185 | 96% | 4e-54 | 21.40% | 252 | MAI67428.1(scored below threshold on previous iteration) | Select seq MAI67428.1 for PSI blast |  |  |
  | 79Select seq gb|MBE9487910.1| | proliferating cell nuclear antigen (pcna) [Bacteroidetes bacterium] | Bacteroidetes bacterium | NA | 1898104 | 184 | 184 | 98% | 7e-54 | 23.08% | 245 | MBE9487910.1(scored below threshold on previous iteration) | Select seq MBE9487910.1 for PSI blast |  |  |
  | 80Select seq gb|MRR35945.1| | proliferating cell nuclear antigen (pcna) [bacterium] | bacterium | NA | 1869227 | 183 | 183 | 98% | 8e-54 | 23.08% | 244 | MRR35945.1(scored below threshold on previous iteration) | Select seq MRR35945.1 for PSI blast |  |  |
  | 81Select seq gb|KKW29271.1| | polymerase sliding clamp protein [Parcubacteria group bacterium GW2011\_GWB1\_52\_7] | Parcubacteria group bacterium GW2011\_GWB1\_52\_7 | NA | 1618885 | 183 | 183 | 98% | 1e-53 | 21.40% | 247 | KKW29271.1(scored below threshold on previous iteration) | Select seq KKW29271.1 for PSI blast |  |  |
  | 82Select seq gb|NIS33632.1| | DNA polymerase sliding clamp [Actinobacteria bacterium] | Actinobacteria bacterium | NA | 1883427 | 180 | 180 | 98% | 3e-52 | 22.81% | 247 | NIS33632.1(scored below threshold on previous iteration) | Select seq NIS33632.1 for PSI blast |  |  |
  | 83Select seq gb|MBB18791.1| | proliferating cell nuclear antigen (pcna) [Rickettsiales bacterium] | Rickettsiales bacterium | NA | 2026788 | 181 | 181 | 93% | 4e-52 | 27.89% | 299 | MBB18791.1(scored below threshold on previous iteration) | Select seq MBB18791.1 for PSI blast |  |  |
  | 84Select seq gb|OUU15212.1| | proliferating cell nuclear antigen (pcna) [Candidatus Endolissoclinum sp. TMED37] | Candidatus Endolissoclinum sp. TMED37 | NA | 1986638 | 178 | 178 | 94% | 2e-51 | 26.36% | 264 | OUU15212.1(scored below threshold on previous iteration) | Select seq OUU15212.1 for PSI blast |  |  |
  | 85Select seq gb|NPA85815.1| | proliferating cell nuclear antigen (pcna) [bacterium] | bacterium | NA | 1869227 | 177 | 177 | 98% | 3e-51 | 19.39% | 254 | NPA85815.1(scored below threshold on previous iteration) | Select seq NPA85815.1 for PSI blast |  |  |
  | 86Select seq gb|NBT48094.1| | proliferating cell nuclear antigen (pcna) [Actinobacteria bacterium] | Actinobacteria bacterium | NA | 1883427 | 177 | 177 | 98% | 4e-51 | 23.64% | 248 | NBT48094.1(scored below threshold on previous iteration) | Select seq NBT48094.1 for PSI blast |  |  |
  | 87Select seq gb|NDA64219.1| | proliferating cell nuclear antigen (pcna) [Chitinophagia bacterium] | Chitinophagia bacterium | NA | 2448778 | 175 | 175 | 74% | 5e-51 | 26.67% | 209 | NDA64219.1(scored below threshold on previous iteration) | Select seq NDA64219.1 for PSI blast |  |  |
  | 88Select seq gb|NDE18324.1| | hypothetical protein [bacterium] | bacterium | NA | 1869227 | 177 | 177 | 99% | 1e-50 | 20.61% | 282 | NDE18324.1(scored below threshold on previous iteration) | Select seq NDE18324.1 for PSI blast |  |  |
  | 89Select seq gb|NDA89590.1| | proliferating cell nuclear antigen (pcna) [Alphaproteobacteria bacterium] | Alphaproteobacteria bacterium | NA | 1913988 | 176 | 176 | 94% | 2e-50 | 25.30% | 280 | NDA89590.1(scored below threshold on previous iteration) | Select seq NDA89590.1 for PSI blast |  |  |
  | 90Select seq gb|MAI14456.1| | proliferating cell nuclear antigen (pcna) [Rhodospirillaceae bacterium] | Rhodospirillaceae bacterium | NA | 1898112 | 175 | 175 | 98% | 4e-50 | 22.56% | 261 | MAI14456.1(scored below threshold on previous iteration) | Select seq MAI14456.1 for PSI blast |  |  |
  | 91Select seq gb|MAB04571.1| | proliferating cell nuclear antigen (pcna) [Rhodobacteraceae bacterium] | Rhodobacteraceae bacterium | NA | 1904441 | 175 | 175 | 95% | 4e-50 | 24.90% | 256 | MAB04571.1(scored below threshold on previous iteration) | Select seq MAB04571.1 for PSI blast |  |  |
  | 92Select seq gb|MBN20023.1| | proliferating cell nuclear antigen (pcna) [Bdellovibrionaceae bacterium] | Bdellovibrionaceae bacterium | NA | 2026715 | 173 | 173 | 96% | 2e-49 | 23.88% | 270 | MBN20023.1(scored below threshold on previous iteration) | Select seq MBN20023.1 for PSI blast |  |  |
  | 93Select seq gb|NIQ14317.1| | DNA polymerase sliding clamp [Candidatus Dadabacteria bacterium] | Candidatus Dadabacteria bacterium | NA | 2080303 | 171 | 171 | 96% | 8e-49 | 21.18% | 254 | NIQ14317.1(scored below threshold on previous iteration) | Select seq NIQ14317.1 for PSI blast |  |  |
  | 94Select seq gb|MBI63664.1| | hypothetical protein [Chloroflexi bacterium] | Chloroflexi bacterium | NA | 2026724 | 168 | 168 | 96% | 9e-48 | 21.09% | 248 | MBI63664.1(scored below threshold on previous iteration) | Select seq MBI63664.1 for PSI blast |  |  |
  | 95Select seq gb|MAE80883.1| | DNA polymerase sliding clamp [Chloroflexi bacterium] | Chloroflexi bacterium | NA | 2026724 | 168 | 168 | 96% | 1e-47 | 19.31% | 253 | MAE80883.1(scored below threshold on previous iteration) | Select seq MAE80883.1 for PSI blast |  |  |
  | 96Select seq gb|MBH51453.1| | hypothetical protein [Candidatus Marinimicrobia bacterium] | Candidatus Marinimicrobia bacterium | NA | 2026760 | 165 | 165 | 96% | 2e-46 | 22.78% | 248 | MBH51453.1(scored below threshold on previous iteration) | Select seq MBH51453.1 for PSI blast |  |  |
  | 97Select seq gb|MAN39109.1| | hypothetical protein [Gammaproteobacteria bacterium] | Gammaproteobacteria bacterium | NA | 1913989 | 165 | 165 | 96% | 3e-46 | 20.46% | 249 | MAN39109.1(scored below threshold on previous iteration) | Select seq MAN39109.1 for PSI blast |  |  |
  | 98Select seq gb|MAP67643.1| | proliferating cell nuclear antigen (pcna) [Candidatus Marinimicrobia bacterium] | Candidatus Marinimicrobia bacterium | NA | 2026760 | 165 | 165 | 94% | 3e-46 | 22.40% | 261 | MAP67643.1(scored below threshold on previous iteration) | Select seq MAP67643.1 for PSI blast |  |  |
  | 99Select seq tpg|HFO52032.1| | TPA: proliferating cell nuclear antigen (pcna) [Ignavibacteria bacterium] | Ignavibacteria bacterium | NA | 2053306 | 168 | 168 | 85% | 9e-46 | 22.22% | 428 | HFO52032.1(scored below threshold on previous iteration) | Select seq HFO52032.1 for PSI blast |  |  |
  | 100Select seq gb|KKR01610.1| | polymerase sliding clamp protein [Candidatus Woesebacteria bacterium GW2011\_GWB1\_39\_12] | Candidatus Woesebacteria bacterium GW2011\_GWB1\_39\_12 | NA | 1618574 | 164 | 164 | 99% | 1e-45 | 22.51% | 294 | KKR01610.1(scored below threshold on previous iteration) | Select seq KKR01610.1 for PSI blast |  |  |
  | 101Select seq gb|NCS98517.1| | hypothetical protein [bacterium] | bacterium | NA | 1869227 | 162 | 162 | 98% | 2e-45 | 20.23% | 245 | NCS98517.1(scored below threshold on previous iteration) | Select seq NCS98517.1 for PSI blast |  |  |
  | 102Select seq gb|NDE15318.1| | hypothetical protein [bacterium] | bacterium | NA | 1869227 | 163 | 163 | 97% | 3e-45 | 22.61% | 273 | NDE15318.1(scored below threshold on previous iteration) | Select seq NDE15318.1 for PSI blast |  |  |
  | 103Select seq gb|OLD29434.1| | DNA polymerase sliding clamp [Candidatus Rokubacteria bacterium 13\_1\_40CM\_2\_70\_45] | Candidatus Rokubacteria bacterium 13\_1\_40CM\_2\_70\_45 | NA | 1805357 | 160 | 160 | 81% | 6e-45 | 24.30% | 201 | OLD29434.1(scored below threshold on previous iteration) | Select seq OLD29434.1 for PSI blast |  |  |
  | 104Select seq gb|MBC8306902.1| | proliferating cell nuclear antigen (pcna) [Pelagibacterales bacterium] | Pelagibacterales bacterium | NA | 2026776 | 162 | 162 | 96% | 6e-45 | 21.30% | 276 | MBC8306902.1(scored below threshold on previous iteration) | Select seq MBC8306902.1 for PSI blast |  |  |
  | 105Select seq gb|MBQ40860.1| | DNA polymerase sliding clamp [Gemmatimonadetes bacterium] | Gemmatimonadetes bacterium | NA | 2026742 | 160 | 160 | 96% | 2e-44 | 17.37% | 258 | MBQ40860.1(scored below threshold on previous iteration) | Select seq MBQ40860.1 for PSI blast |  |  |
  | 106Select seq gb|OLC90189.1| | DNA polymerase sliding clamp [Candidatus Rokubacteria bacterium 13\_1\_40CM\_3\_69\_38] | Candidatus Rokubacteria bacterium 13\_1\_40CM\_3\_69\_38 | NA | 1805359 | 158 | 158 | 81% | 2e-44 | 24.77% | 200 | OLC90189.1(scored below threshold on previous iteration) | Select seq OLC90189.1 for PSI blast |  |  |
  | 107Select seq gb|RYZ28222.1| | proliferating cell nuclear antigen (pcna) [Propionibacteriaceae bacterium] | Propionibacteriaceae bacterium | NA | 2021380 | 159 | 159 | 75% | 3e-44 | 28.43% | 235 | RYZ28222.1(scored below threshold on previous iteration) | Select seq RYZ28222.1 for PSI blast |  |  |
  | 108Select seq gb|MBE29542.1| | hypothetical protein [bacterium] | bacterium | NA | 1869227 | 160 | 160 | 95% | 3e-44 | 23.02% | 275 | MBE29542.1(scored below threshold on previous iteration) | Select seq MBE29542.1 for PSI blast |  |  |
  | 109Select seq gb|MAI14441.1| | hypothetical protein [Rhodospirillaceae bacterium] | Rhodospirillaceae bacterium | NA | 1898112 | 159 | 159 | 99% | 7e-44 | 20.45% | 267 | MAI14441.1(scored below threshold on previous iteration) | Select seq MAI14441.1 for PSI blast |  |  |
  | 110Select seq gb|MAL04566.1| | hypothetical protein [Rhodobiaceae bacterium] | Rhodobiaceae bacterium | NA | 2026785 | 158 | 158 | 96% | 8e-44 | 19.61% | 246 | MAL04566.1(scored below threshold on previous iteration) | Select seq MAL04566.1 for PSI blast |  |  |
  | 111Select seq gb|NDG54324.1| | proliferating cell nuclear antigen (pcna) [Flavobacteriia bacterium] | Flavobacteriia bacterium | NA | 2044941 | 159 | 159 | 97% | 2e-43 | 21.78% | 308 | NDG54324.1(scored below threshold on previous iteration) | Select seq NDG54324.1 for PSI blast |  |  |
  | 112Select seq gb|MAV57173.1| | proliferating cell nuclear antigen (pcna) [Candidatus Pelagibacter sp.] | Candidatus Pelagibacter sp. | NA | 2024849 | 158 | 158 | 96% | 2e-43 | 22.13% | 260 | MAV57173.1(scored below threshold on previous iteration) | Select seq MAV57173.1 for PSI blast |  |  |
  | 113Select seq gb|MAR49569.1| | proliferating cell nuclear antigen (pcna) [Rhodobacteraceae bacterium] | Rhodobacteraceae bacterium | NA | 1904441 | 157 | 157 | 95% | 2e-43 | 20.38% | 268 | MAR49569.1(scored below threshold on previous iteration) | Select seq MAR49569.1 for PSI blast |  |  |
  | 114Select seq gb|NDA89441.1| | proliferating cell nuclear antigen (pcna) [Alphaproteobacteria bacterium] | Alphaproteobacteria bacterium | NA | 1913988 | 155 | 155 | 70% | 2e-43 | 26.98% | 196 | NDA89441.1(scored below threshold on previous iteration) | Select seq NDA89441.1 for PSI blast |  |  |
  | 115Select seq tpg|HIQ51620.1| | TPA: hypothetical protein [Nautiliaceae bacterium] | Nautiliaceae bacterium | NA | 2306051 | 156 | 156 | 97% | 3e-43 | 23.05% | 246 | HIQ51620.1(scored below threshold on previous iteration) | Select seq HIQ51620.1 for PSI blast |  |  |
  | 116Select seq gb|NDG33583.1| | hypothetical protein [bacterium] | bacterium | NA | 1869227 | 151 | 151 | 54% | 7e-42 | 29.37% | 177 | NDG33583.1(scored below threshold on previous iteration) | Select seq NDG33583.1 for PSI blast |  |  |
  | 117Select seq gb|NDB86579.1| | hypothetical protein [Alphaproteobacteria bacterium] | Alphaproteobacteria bacterium | NA | 1913988 | 148 | 148 | 60% | 3e-41 | 25.79% | 159 | NDB86579.1(scored below threshold on previous iteration) | Select seq NDB86579.1 for PSI blast |  |  |
  | 118Select seq gb|NBP58287.1| | hypothetical protein [bacterium] | bacterium | NA | 1869227 | 151 | 151 | 96% | 4e-41 | 17.32% | 259 | NBP58287.1(scored below threshold on previous iteration) | Select seq NBP58287.1 for PSI blast |  |  |
  | 119Select seq gb|OLC55625.1| | DNA polymerase sliding clamp [Candidatus Rokubacteria bacterium 13\_1\_40CM\_4\_69\_39] | Candidatus Rokubacteria bacterium 13\_1\_40CM\_4\_69\_39 | NA | 1805363 | 149 | 149 | 77% | 7e-41 | 24.51% | 190 | OLC55625.1(scored below threshold on previous iteration) | Select seq OLC55625.1 for PSI blast |  |  |
  | 120Select seq gb|MAP62052.1| | hypothetical protein [Candidatus Marinimicrobia bacterium] | Candidatus Marinimicrobia bacterium | NA | 2026760 | 146 | 146 | 96% | 4e-39 | 18.18% | 260 | MAP62052.1(scored below threshold on previous iteration) | Select seq MAP62052.1 for PSI blast |  |  |
  | 121Select seq gb|RLC83458.1| | hypothetical protein DRI37\_10220 [Chloroflexi bacterium] | Chloroflexi bacterium | NA | 2026724 | 145 | 145 | 86% | 5e-39 | 21.24% | 215 | RLC83458.1(scored below threshold on previous iteration) | Select seq RLC83458.1 for PSI blast |  |  |
  | 122Select seq gb|RYX78042.1| | hypothetical protein EON71\_01370 [bacterium] | bacterium | NA | 1869227 | 146 | 146 | 96% | 4e-38 | 17.81% | 327 | RYX78042.1(scored below threshold on previous iteration) | Select seq RYX78042.1 for PSI blast |  |  |
  | 123Select seq gb|NIA11429.1| | DNA polymerase sliding clamp [Nitrospiraceae bacterium] | Nitrospiraceae bacterium | NA | 2026770 | 143 | 143 | 98% | 6e-38 | 18.01% | 242 | NIA11429.1(scored below threshold on previous iteration) | Select seq NIA11429.1 for PSI blast |  |  |
  | 124Select seq gb|MAR96207.1| | hypothetical protein [Candidatus Marinimicrobia bacterium] | Candidatus Marinimicrobia bacterium | NA | 2026760 | 143 | 143 | 94% | 9e-38 | 21.71% | 268 | MAR96207.1(scored below threshold on previous iteration) | Select seq MAR96207.1 for PSI blast |  |  |
  | 125Select seq gb|MAR96282.1| | hypothetical protein [Candidatus Marinimicrobia bacterium] | Candidatus Marinimicrobia bacterium | NA | 2026760 | 140 | 140 | 94% | 1e-36 | 21.32% | 268 | MAR96282.1(scored below threshold on previous iteration) | Select seq MAR96282.1 for PSI blast |  |  |
  | 126Select seq gb|MAJ82112.1| | hypothetical protein [Legionellales bacterium] | Legionellales bacterium | NA | 2026754 | 139 | 139 | 97% | 2e-36 | 18.60% | 254 | MAJ82112.1(scored below threshold on previous iteration) | Select seq MAJ82112.1 for PSI blast |  |  |
  | 127Select seq tpg|HGO27509.1| | TPA: proliferating cell nuclear antigen (pcna) [Proteobacteria bacterium] | Proteobacteria bacterium | NA | 1977087 | 138 | 138 | 96% | 5e-36 | 21.65% | 249 | HGO27509.1(scored below threshold on previous iteration) | Select seq HGO27509.1 for PSI blast |  |  |
  | 128Select seq gb|OGO49108.1| | hypothetical protein A2W34\_06430 [Chloroflexi bacterium RBG\_16\_64\_32] | Chloroflexi bacterium RBG\_16\_64\_32 | NA | 1797658 | 135 | 135 | 95% | 8e-34 | 19.62% | 365 | OGO49108.1(scored below threshold on previous iteration) | Select seq OGO49108.1 for PSI blast |  |  |
  | 129Select seq gb|MBI96709.1| | hypothetical protein [bacterium] | bacterium | NA | 1869227 | 127 | 127 | 98% | 3e-31 | 20.86% | 289 | MBI96709.1(scored below threshold on previous iteration) | Select seq MBI96709.1 for PSI blast |  |  |
  | 130Select seq gb|NBS68524.1| | hypothetical protein [bacterium] | bacterium | NA | 1869227 | 121 | 121 | 43% | 5e-31 | 29.20% | 112 | NBS68524.1(scored below threshold on previous iteration) | Select seq NBS68524.1 for PSI blast |  |  |
  | 131Select seq gb|MAU35866.1| | hypothetical protein [Flavobacteriales bacterium] | Flavobacteriales bacterium | NA | 2021391 | 122 | 122 | 63% | 1e-30 | 23.67% | 179 | MAU35866.1(scored below threshold on previous iteration) | Select seq MAU35866.1 for PSI blast |  |  |
  | 132Select seq gb|MAR17754.1| | hypothetical protein [Rhodobacteraceae bacterium] | Rhodobacteraceae bacterium | NA | 1904441 | 118 | 118 | 94% | 3e-28 | 19.68% | 249 | MAR17754.1(scored below threshold on previous iteration) | Select seq MAR17754.1 for PSI blast |  |  |
  | 133Select seq gb|RKY57622.1| | DNA polymerase sliding clamp [Candidatus Latescibacteria bacterium] | Candidatus Latescibacteria bacterium | NA | 2053570 | 112 | 112 | 57% | 3e-27 | 21.33% | 148 | RKY57622.1(scored below threshold on previous iteration) | Select seq RKY57622.1 for PSI blast |  |  |
  | 134Select seq ref|WP\_071289881.1| | proliferating cell nuclear antigen (pcna) [Acinetobacter baumannii] | Acinetobacter baumannii | NA | 470 | 110 | 110 | 41% | 8e-27 | 65.74% | 108 | WP\_071289881.1(scored below threshold on previous iteration) | Select seq WP\_071289881.1 for PSI blast |  |  |
  | 135Select seq gb|MBC7332724.1| | DNA polymerase sliding clamp [Synergistetes bacterium] | Synergistetes bacterium | NA | 2268202 | 108 | 108 | 65% | 1e-25 | 23.39% | 159 | MBC7332724.1(scored below threshold on previous iteration) | Select seq MBC7332724.1 for PSI blast |  |  |
  | 136Select seq gb|NCA21076.1| | hypothetical protein [Crocinitomicaceae bacterium] | Crocinitomicaceae bacterium | NA | 2026728 | 101 | 101 | 44% | 3e-23 | 29.31% | 118 | NCA21076.1(scored below threshold on previous iteration) | Select seq NCA21076.1 for PSI blast |  |  |
  | 137Select seq gb|RYF05887.1| | hypothetical protein EOO40\_09850 [Deltaproteobacteria bacterium] | Deltaproteobacteria bacterium | NA | 2026735 | 104 | 104 | 43% | 9e-23 | 20.87% | 272 | RYF05887.1(scored below threshold on previous iteration) | Select seq RYF05887.1 for PSI blast |  |  |
  | 138Select seq gb|TAE70583.1| | hypothetical protein EAZ85\_11740 [Bacteroidetes bacterium] | Bacteroidetes bacterium | NA | 1898104 | 99.2 | 99.2 | 35% | 1e-22 | 34.41% | 103 | TAE70583.1(scored below threshold on previous iteration) | Select seq TAE70583.1 for PSI blast |  |  |
  | 139Select seq gb|NBS88271.1| | hypothetical protein [Verrucomicrobia bacterium] | Verrucomicrobia bacterium | NA | 2026799 | 100 | 100 | 35% | 7e-22 | 24.47% | 214 | NBS88271.1(scored below threshold on previous iteration) | Select seq NBS88271.1 for PSI blast |  |  |
  | 140Select seq gb|MBH45101.1| | hypothetical protein [Flavobacteriaceae bacterium] | Flavobacteriaceae bacterium | NA | 1871037 | 101 | 101 | 98% | 9e-22 | 18.15% | 255 | MBH45101.1(scored below threshold on previous iteration) | Select seq MBH45101.1 for PSI blast |  |  |
  | 141Select seq gb|OEU74059.1| | hypothetical protein BA864\_06935 [Desulfuromonadales bacterium C00003093] | Desulfuromonadales bacterium C00003093 | NA | 1869308 | 99 | 99 | 98% | 2e-21 | 19.08% | 247 | OEU74059.1(scored below threshold on previous iteration) | Select seq OEU74059.1 for PSI blast |  |  |
  | 142Select seq tpg|HIP92434.1| | TPA: DNA polymerase sliding clamp [Thermotoga sp.] | Thermotoga sp. | NA | 28240 | 93.8 | 93.8 | 55% | 4e-20 | 16.44% | 145 | HIP92434.1(scored below threshold on previous iteration) | Select seq HIP92434.1 for PSI blast |  |  |
  | 143Select seq ref|WP\_147509764.1| | proliferating cell nuclear antigen (pcna) [Acinetobacter baumannii] | Acinetobacter baumannii | NA | 470 | 91.5 | 91.5 | 29% | 5e-20 | 86.84% | 76 | WP\_147509764.1(scored below threshold on previous iteration) | Select seq WP\_147509764.1 for PSI blast |  |  |
  | 144Select seq tpg|HER14123.1| | TPA: hypothetical protein [Fischerella sp.] | Fischerella sp. | NA | 1191 | 93.4 | 93.4 | 57% | 6e-20 | 24.50% | 143 | HER14123.1(scored below threshold on previous iteration) | Select seq HER14123.1 for PSI blast |  |  |
  | 145Select seq gb|NBS18941.1| | hypothetical protein [Flavobacteriia bacterium] | Flavobacteriia bacterium | NA | 2044941 | 91.8 | 91.8 | 45% | 1e-19 | 25.20% | 123 | NBS18941.1(scored below threshold on previous iteration) | Select seq NBS18941.1 for PSI blast |  |  |
  | 146Select seq gb|NDB84380.1| | hypothetical protein [Alphaproteobacteria bacterium] | Alphaproteobacteria bacterium | NA | 1913988 | 89.9 | 89.9 | 35% | 3e-19 | 29.03% | 96 | NDB84380.1(scored below threshold on previous iteration) | Select seq NDB84380.1 for PSI blast |  |  |
  | 147Select seq gb|NBS67530.1| | hypothetical protein [bacterium] | bacterium | NA | 1869227 | 88.8 | 88.8 | 40% | 9e-19 | 30.28% | 98 | NBS67530.1(scored below threshold on previous iteration) | Select seq NBS67530.1 for PSI blast |  |  |
  | 148Select seq gb|NDB86628.1| | hypothetical protein [Alphaproteobacteria bacterium] | Alphaproteobacteria bacterium | NA | 1913988 | 86.8 | 86.8 | 26% | 4e-18 | 30.43% | 86 | NDB86628.1(scored below threshold on previous iteration) | Select seq NDB86628.1 for PSI blast |  |  |
  | 149Select seq gb|NBY39677.1| | hypothetical protein [Flavobacteriia bacterium] | Flavobacteriia bacterium | NA | 2044941 | 86.8 | 86.8 | 34% | 5e-18 | 31.46% | 94 | NBY39677.1(scored below threshold on previous iteration) | Select seq NBY39677.1 for PSI blast |  |  |
  | 150Select seq gb|MBF6555506.1| | hypothetical protein [Acidimicrobiales bacterium] | Acidimicrobiales bacterium | NA | 2201156 | 93.4 | 93.4 | 90% | 6e-18 | 17.50% | 430 | MBF6555506.1(scored below threshold on previous iteration) | Select seq MBF6555506.1 for PSI blast |  |  |
  | 151Select seq gb|RLC75119.1| | hypothetical protein DRI61\_15465 [Chloroflexi bacterium] | Chloroflexi bacterium | NA | 2026724 | 91.5 | 91.5 | 89% | 9e-18 | 16.60% | 301 | RLC75119.1(scored below threshold on previous iteration) | Select seq RLC75119.1 for PSI blast |  |  |
  | 152Select seq gb|OPZ48648.1| | DNA polymerase sliding clamp [Bacteroidetes bacterium ADurb.BinA104] | Bacteroidetes bacterium ADurb.BinA104 | NA | 1852816 | 89.1 | 89.1 | 96% | 3e-17 | 19.38% | 248 | OPZ48648.1(scored below threshold on previous iteration) | Select seq OPZ48648.1 for PSI blast |  |  |
  | 153Select seq tpg|HID65709.1| | TPA: hypothetical protein [Aquificaceae bacterium] | Aquificaceae bacterium | NA | 2053503 | 84.9 | 84.9 | 36% | 7e-17 | 23.40% | 132 | HID65709.1(scored below threshold on previous iteration) | Select seq HID65709.1 for PSI blast |  |  |
  | 154Select seq tpg|HDT11972.1| | TPA: hypothetical protein [bacterium] | bacterium | NA | 1869227 | 80.7 | 80.7 | 40% | 1e-15 | 23.58% | 101 | HDT11972.1(scored below threshold on previous iteration) | Select seq HDT11972.1 for PSI blast |  |  |
  | 155Select seq gb|MAU37716.1| | hypothetical protein [Flavobacteriales bacterium] | Flavobacteriales bacterium | NA | 2021391 | 78.0 | 78.0 | 44% | 2e-14 | 18.97% | 111 | MAU37716.1(scored below threshold on previous iteration) | Select seq MAU37716.1 for PSI blast |  |  |
  | 156Select seq gb|OEU73910.1| | hypothetical protein BA864\_05090 [Desulfuromonadales bacterium C00003093] | Desulfuromonadales bacterium C00003093 | NA | 1869308 | 78.0 | 78.0 | 95% | 3e-13 | 16.60% | 244 | OEU73910.1(scored below threshold on previous iteration) | Select seq OEU73910.1 for PSI blast |  |  |
  | 157Select seq gb|NBX48675.1| | hypothetical protein [bacterium] | bacterium | NA | 1869227 | 77.6 | 77.6 | 93% | 4e-13 | 19.29% | 248 | NBX48675.1(scored below threshold on previous iteration) | Select seq NBX48675.1 for PSI blast |  |  |
  | 158Select seq ref|WP\_067050733.1| | hypothetical protein [Moritella sp. JT01] | Moritella sp. JT01 | NA | 756698 | 77.6 | 77.6 | 90% | 6e-13 | 17.15% | 257 | WP\_067050733.1(scored below threshold on previous iteration) | Select seq WP\_067050733.1 for PSI blast |  |  |
  | 159Select seq gb|MBE3094252.1| | hypothetical protein [Actinobacteria bacterium] | Actinobacteria bacterium | NA | 1883427 | 76.1 | 76.1 | 95% | 2e-12 | 15.73% | 266 | MBE3094252.1(scored below threshold on previous iteration) | Select seq MBE3094252.1 for PSI blast |  |  |
  | 160Select seq gb|RLD18005.1| | hypothetical protein DRI69\_11125 [Bacteroidetes bacterium] | Bacteroidetes bacterium | NA | 1898104 | 74.9 | 74.9 | 96% | 3e-12 | 17.79% | 241 | RLD18005.1(scored below threshold on previous iteration) | Select seq RLD18005.1 for PSI blast |  |  |
  | 161Select seq tpg|HAI41630.1| | TPA: hypothetical protein [Maribacter sp.] | Maribacter sp. | NA | 1897614 | 74.9 | 74.9 | 95% | 5e-12 | 14.06% | 254 | HAI41630.1(scored below threshold on previous iteration) | Select seq HAI41630.1 for PSI blast |  |  |
  | 162Select seq gb|MAD25962.1| | hypothetical protein [Verrucomicrobiales bacterium] | Verrucomicrobiales bacterium | NA | 2026801 | 75.7 | 75.7 | 98% | 5e-12 | 17.02% | 304 | MAD25962.1(scored below threshold on previous iteration) | Select seq MAD25962.1 for PSI blast |  |  |
  | 163Select seq gb|RLC69169.1| | hypothetical protein DRH97\_00615 [Chloroflexi bacterium] | Chloroflexi bacterium | NA | 2026724 | 74.5 | 74.5 | 97% | 5e-12 | 17.12% | 239 | RLC69169.1(scored below threshold on previous iteration) | Select seq RLC69169.1 for PSI blast |  |  |
  | 164Select seq gb|NBX51827.1| | AAA family ATPase [bacterium] | bacterium | NA | 1869227 | 75.3 | 75.3 | 24% | 9e-12 | 23.08% | 360 | NBX51827.1(scored below threshold on previous iteration) | Select seq NBX51827.1 for PSI blast |  |  |
  | 165Select seq gb|OUU14720.1| | hypothetical protein CBB97\_24650 [Candidatus Endolissoclinum sp. TMED37] | Candidatus Endolissoclinum sp. TMED37 | NA | 1986638 | 69.5 | 69.5 | 55% | 7e-11 | 24.00% | 146 | OUU14720.1(scored below threshold on previous iteration) | Select seq OUU14720.1 for PSI blast |  |  |
  | 166Select seq tpg|HEC40414.1| | TPA: hypothetical protein [bacterium] | bacterium | NA | 1869227 | 71.0 | 71.0 | 59% | 1e-10 | 23.27% | 259 | HEC40414.1(scored below threshold on previous iteration) | Select seq HEC40414.1 for PSI blast |  |  |
  | 167Select seq ref|WP\_036504199.1| | DNA polymerase [Nitrosococcus oceani] | Nitrosococcus oceani | NA | 1229 | 67.2 | 67.2 | 36% | 1e-10 | 26.04% | 102 | WP\_036504199.1(scored below threshold on previous iteration) | Select seq WP\_036504199.1 for PSI blast |  |  |
  | 168Select seq gb|NCU42455.1| | hypothetical protein [Candidatus Moranbacteria bacterium] | Candidatus Moranbacteria bacterium | NA | 2045217 | 70.3 | 70.3 | 97% | 2e-10 | 15.62% | 239 | NCU42455.1(scored below threshold on previous iteration) | Select seq NCU42455.1 for PSI blast |  |  |
  | 169Select seq gb|MBL97255.1| | hypothetical protein [Legionellales bacterium] | Legionellales bacterium | NA | 2026754 | 68.7 | 68.7 | 98% | 1e-09 | 18.46% | 304 | MBL97255.1(scored below threshold on previous iteration) | Select seq MBL97255.1 for PSI blast |  |  |
  | 170Select seq gb|MAF41652.1| | hypothetical protein [Cyanobium sp. ARS6] | Cyanobium sp. ARS6 | NA | 2024869 | 66.0 | 66.0 | 87% | 6e-09 | 15.42% | 232 | MAF41652.1(scored below threshold on previous iteration) | Select seq MAF41652.1 for PSI blast |  |  |
  | 171Select seq ref|WP\_156312854.1| | hypothetical protein [Microvirga massiliensis] | Microvirga massiliensis | NA | 1033741 | 62.6 | 62.6 | 37% | 7e-09 | 27.84% | 92 | WP\_156312854.1(scored below threshold on previous iteration) | Select seq WP\_156312854.1 for PSI blast |  |  |
  | 172Select seq gb|PCJ29226.1| | hypothetical protein COA94\_02085 [Rickettsiales bacterium] | Rickettsiales bacterium | NA | 2026788 | 64.9 | 64.9 | 96% | 2e-08 | 18.85% | 269 | PCJ29226.1(scored below threshold on previous iteration) | Select seq PCJ29226.1 for PSI blast |  |  |
  | 173Select seq tpg|HIK66788.1| | TPA: hypothetical protein [Flavobacteriales bacterium] | Flavobacteriales bacterium | NA | 2021391 | 63.0 | 63.0 | 62% | 8e-08 | 15.98% | 244 | HIK66788.1(scored below threshold on previous iteration) | Select seq HIK66788.1 for PSI blast |  |  |
  | 174Select seq gb|MSP65089.1| | DNA polymerase sliding clamp [Bacteroidetes bacterium] | Bacteroidetes bacterium | NA | 1898104 | 57.9 | 57.9 | 18% | 1e-07 | 32.65% | 51 | MSP65089.1(scored below threshold on previous iteration) | Select seq MSP65089.1 for PSI blast |  |  |
  | 175Select seq gb|MAY90458.1| | hypothetical protein [Rickettsiales bacterium] | Rickettsiales bacterium | NA | 2026788 | 62.2 | 62.2 | 62% | 1e-07 | 17.37% | 244 | MAY90458.1(scored below threshold on previous iteration) | Select seq MAY90458.1 for PSI blast |  |  |
  | 176Select seq gb|MAI63263.1| | hypothetical protein [Alteromonas sp.] | Alteromonas sp. | NA | 232 | 59.9 | 59.9 | 59% | 3e-07 | 16.56% | 175 | MAI63263.1(scored below threshold on previous iteration) | Select seq MAI63263.1 for PSI blast |  |  |
  | 177Select seq gb|MBP98812.1| | hypothetical protein [Candidatus Poribacteria bacterium] | Candidatus Poribacteria bacterium | NA | 2026781 | 61.0 | 61.0 | 62% | 4e-07 | 16.17% | 244 | MBP98812.1(scored below threshold on previous iteration) | Select seq MBP98812.1 for PSI blast |  |  |
  | 178Select seq tpg|HGU52075.1| | TPA: DNA polymerase sliding clamp [Fervidobacterium pennivorans] | Fervidobacterium pennivorans | NA | 93466 | 56.8 | 56.8 | 19% | 4e-07 | 38.46% | 53 | HGU52075.1(scored below threshold on previous iteration) | Select seq HGU52075.1 for PSI blast |  |  |
  | 179Select seq gb|OQA51571.1| | DNA polymerase sliding clamp [Bacteroidetes bacterium ADurb.Bin302] | Bacteroidetes bacterium ADurb.Bin302 | NA | 1852811 | 60.3 | 60.3 | 96% | 5e-07 | 19.39% | 234 | OQA51571.1(scored below threshold on previous iteration) | Select seq OQA51571.1 for PSI blast |  |  |
  | 180Select seq gb|RPF82134.1| | hypothetical protein CBC65\_000905 [Rhodothermaceae bacterium TMED105] | Rhodothermaceae bacterium TMED105 | NA | 1986665 | 58.7 | 58.7 | 95% | 4e-06 | 13.68% | 307 | RPF82134.1(scored below threshold on previous iteration) | Select seq RPF82134.1 for PSI blast |  |  |
  | 181Select seq gb|NBK24950.1| | hypothetical protein [Spirochaetia bacterium] | Spirochaetia bacterium | NA | 2053615 | 54.5 | 54.5 | 42% | 9e-06 | 25.00% | 109 | NBK24950.1(scored below threshold on previous iteration) | Select seq NBK24950.1 for PSI blast |  |  |
  | 182Select seq gb|MBP98540.1| | hypothetical protein [Candidatus Poribacteria bacterium] | Candidatus Poribacteria bacterium | NA | 2026781 | 55.6 | 55.6 | 78% | 3e-05 | 13.81% | 242 | MBP98540.1(scored below threshold on previous iteration) | Select seq MBP98540.1 for PSI blast |  |  |
  | 183Select seq gb|NBV35239.1| | hypothetical protein [Proteobacteria bacterium] | Proteobacteria bacterium | NA | 1977087 | 52.2 | 52.2 | 25% | 3e-05 | 31.82% | 75 | NBV35239.1(scored below threshold on previous iteration) | Select seq NBV35239.1 for PSI blast |  |  |
  | 184Select seq gb|NCU27524.1| | hypothetical protein [Candidatus Nomurabacteria bacterium] | Candidatus Nomurabacteria bacterium | NA | 2052152 | 53.7 | 53.7 | 63% | 6e-05 | 14.53% | 193 | NCU27524.1(scored below threshold on previous iteration) | Select seq NCU27524.1 for PSI blast |  |  |
  | 185Select seq ref|WP\_184202552.1| | DNA polymerase III subunit beta [Armatimonas rosea] | Armatimonas rosea | NA | 685828 | 55.3 | 55.3 | 88% | 7e-05 | 14.06% | 386 | WP\_184202552.1(scored below threshold on previous iteration) | Select seq WP\_184202552.1 for PSI blast |  |  |
  | 186Select seq gb|RKX62390.1| | hypothetical protein DRP41\_07375 [Thermodesulfobacteria bacterium] | Thermodesulfobacteria bacterium | NA | 2202153 | 52.2 | 52.2 | 33% | 7e-05 | 23.86% | 114 | RKX62390.1(scored below threshold on previous iteration) | Select seq RKX62390.1 for PSI blast |  |  |
  | 187Select seq tpg|HID70036.1| | TPA: hypothetical protein [Desulfobacterales bacterium] | Desulfobacterales bacterium | NA | 2044940 | 51.4 | 51.4 | 36% | 1e-04 | 14.89% | 104 | HID70036.1(scored below threshold on previous iteration) | Select seq HID70036.1 for PSI blast |  |  |
  | 188Select seq ref|WP\_199307260.1| | DNA polymerase III subunit beta [Alkalinema sp. FACHB-956] | Alkalinema sp. FACHB-956 | NA | 2692768 | 54.5 | 54.5 | 89% | 1e-04 | 15.16% | 502 | WP\_199307260.1(scored below threshold on previous iteration) | Select seq WP\_199307260.1 for PSI blast |  |  |
  | 189Select seq gb|MBD2328424.1| | DNA polymerase III subunit beta [Alkalinema sp. FACHB-956] | Alkalinema sp. FACHB-956 | NA | 2692768 | 54.1 | 54.1 | 89% | 2e-04 | 15.16% | 504 | MBD2328424.1(scored below threshold on previous iteration) | Select seq MBD2328424.1 for PSI blast |  |  |
  | 190Select seq gb|OCR01436.1| | DNA polymerase III subunit beta [Oscillatoriales cyanobacterium USR001] | Oscillatoriales cyanobacterium USR001 | NA | 1880991 | 53.7 | 53.7 | 94% | 2e-04 | 18.92% | 473 | OCR01436.1(scored below threshold on previous iteration) | Select seq OCR01436.1 for PSI blast |  |  |
  | 191Select seq tpg|HBI72324.1| | TPA: hypothetical protein [Lachnospiraceae bacterium] | Lachnospiraceae bacterium | NA | 1898203 | 53.7 | 53.7 | 95% | 2e-04 | 13.28% | 584 | HBI72324.1(scored below threshold on previous iteration) | Select seq HBI72324.1 for PSI blast |  |  |
  | 192Select seq gb|NBY39678.1| | hypothetical protein [Flavobacteriia bacterium] | Flavobacteriia bacterium | NA | 2044941 | 47.9 | 47.9 | 15% | 4e-04 | 29.27% | 42 | NBY39678.1(scored below threshold on previous iteration) | Select seq NBY39678.1 for PSI blast |  |  |
  | 193Select seq gb|TVQ53692.1| | DNA polymerase III subunit beta [Spirulina sp. DLM2.Bin59] | Spirulina sp. DLM2.Bin59 | NA | 2480215 | 52.6 | 52.6 | 93% | 4e-04 | 16.21% | 382 | TVQ53692.1(scored below threshold on previous iteration) | Select seq TVQ53692.1 for PSI blast |  |  |
  | 194Select seq ref|WP\_084555250.1| | DNA polymerase III subunit beta [Phormidium ambiguum] | Phormidium ambiguum | NA | 71191 | 52.6 | 52.6 | 88% | 6e-04 | 15.97% | 476 | WP\_084555250.1(scored below threshold on previous iteration) | Select seq WP\_084555250.1 for PSI blast |  |  |
  | 195Select seq ref|WP\_017303270.1| | DNA polymerase III subunit beta [Spirulina subsalsa] | Spirulina subsalsa | NA | 54311 | 52.2 | 52.2 | 85% | 6e-04 | 17.54% | 387 | WP\_017303270.1(scored below threshold on previous iteration) | Select seq WP\_017303270.1 for PSI blast |  |  |
  | 196Select seq gb|OKH33175.1| | DNA polymerase III subunit beta [Phormidium ambiguum IAM M-71] | Phormidium ambiguum IAM M-71 | NA | 454136 | 52.2 | 52.2 | 88% | 7e-04 | 15.97% | 408 | OKH33175.1(scored below threshold on previous iteration) | Select seq OKH33175.1 for PSI blast |  |  |
  | 197Select seq gb|NEQ38316.1| | DNA polymerase III subunit beta [Okeania sp. SIO3I5] | Okeania sp. SIO3I5 | NA | 2607805 | 51.4 | 51.4 | 94% | 0.001 | 14.71% | 387 | NEQ38316.1(scored below threshold on previous iteration) | Select seq NEQ38316.1 for PSI blast |  |  |
  | 198Select seq ref|WP\_028393951.1| | DNA polymerase III subunit beta [Bacillus cihuensis] | Bacillus cihuensis | NA | 1208599 | 51.4 | 51.4 | 82% | 0.001 | 17.12% | 370 | WP\_028393951.1(scored below threshold on previous iteration) | Select seq WP\_028393951.1 for PSI blast |  |  |
  | 199Select seq gb|MBI3645860.1| | DNA polymerase III subunit beta [Acidobacteriales bacterium] | Acidobacteriales bacterium | NA | 2282142 | 51.0 | 51.0 | 80% | 0.001 | 17.84% | 371 | MBI3645860.1(scored below threshold on previous iteration) | Select seq MBI3645860.1 for PSI blast |  |  |
  | 200Select seq gb|MBI1740949.1| | DNA polymerase III subunit beta [Candidatus Koribacter versatilis] | Candidatus Koribacter versatilis | NA | 658062 | 51.0 | 51.0 | 80% | 0.002 | 17.84% | 353 | MBI1740949.1(scored below threshold on previous iteration) | Select seq MBI1740949.1 for PSI blast |  |  |
  | 201Select seq ref|WP\_084555156.1| | DNA polymerase III subunit beta [Phormidium ambiguum] | Phormidium ambiguum | NA | 71191 | 51.0 | 51.0 | 88% | 0.002 | 15.55% | 469 | WP\_084555156.1(scored below threshold on previous iteration) | Select seq WP\_084555156.1 for PSI blast |  |  |
  | 202Select seq ref|WP\_162425412.1| | DNA polymerase III subunit beta [Myxacorys almedinensis] | Myxacorys almedinensis | NA | 2651157 | 50.2 | 50.2 | 89% | 0.002 | 15.98% | 258 | WP\_162425412.1(scored below threshold on previous iteration) | Select seq WP\_162425412.1 for PSI blast |  |  |
  | 203Select seq ref|WP\_106166543.1| | DNA polymerase III subunit beta [Chroococcidiopsis cubana] | Chroococcidiopsis cubana | NA | 171392 | 51.0 | 51.0 | 88% | 0.002 | 15.97% | 448 | WP\_106166543.1(scored below threshold on previous iteration) | Select seq WP\_106166543.1 for PSI blast |  |  |
  | 204Select seq gb|OKH36250.1| | DNA polymerase III subunit beta [Phormidium ambiguum IAM M-71] | Phormidium ambiguum IAM M-71 | NA | 454136 | 51.0 | 51.0 | 88% | 0.002 | 15.55% | 408 | OKH36250.1(scored below threshold on previous iteration) | Select seq OKH36250.1 for PSI blast |  |  |
  | 205Select seq gb|MBI3476530.1| | DNA polymerase III subunit beta [Acidobacteria bacterium] | Acidobacteria bacterium | NA | 1978231 | 50.6 | 50.6 | 80% | 0.002 | 19.16% | 371 | MBI3476530.1(scored below threshold on previous iteration) | Select seq MBI3476530.1 for PSI blast |  |  |
  | 206Select seq gb|PYX28173.1| | DNA polymerase III subunit beta [Acidobacteria bacterium] | Acidobacteria bacterium | NA | 1978231 | 50.6 | 50.6 | 80% | 0.002 | 19.25% | 371 | PYX28173.1(scored below threshold on previous iteration) | Select seq PYX28173.1 for PSI blast |  |  |
  | 207Select seq gb|OWY67883.1| | DNA polymerase III subunit beta [cyanobacterium TDX16] | cyanobacterium TDX16 | NA | 1503470 | 50.2 | 50.2 | 88% | 0.003 | 16.60% | 447 | OWY67883.1(scored below threshold on previous iteration) | Select seq OWY67883.1 for PSI blast |  |  |
  | 208Select seq gb|NEO28492.1| | DNA polymerase III subunit beta [Kamptonema sp. SIO4C4] | Kamptonema sp. SIO4C4 | NA | 2607773 | 50.2 | 50.2 | 88% | 0.003 | 18.72% | 389 | NEO28492.1(scored below threshold on previous iteration) | Select seq NEO28492.1 for PSI blast |  |  |
  | 209Select seq ref|WP\_012874944.1| | DNA polymerase III subunit beta [Thermobaculum terrenum] | Thermobaculum terrenum | NA | 166501 | 50.2 | 50.2 | 91% | 0.003 | 16.92% | 376 | WP\_012874944.1(scored below threshold on previous iteration) | Select seq WP\_012874944.1 for PSI blast |  |  |
  | 210Select seq ref|WP\_106458545.1| | DNA polymerase III subunit beta [Aphanothece hegewaldii] | Aphanothece hegewaldii | NA | 1521625 | 49.9 | 49.9 | 94% | 0.003 | 17.20% | 384 | WP\_106458545.1(scored below threshold on previous iteration) | Select seq WP\_106458545.1 for PSI blast |  |  |

  Run PSI-BLAST Iteration 3 with max number of sequences

  Run

  Sequences with E-value WORSE than threshold

  - select all
  - 16 sequences selected
  - PSI-BLAST iteration 2

  Sequences with E-value WORSE than threshold

  | Select for downloading or viewing reports | Description | Scientific Name | Common Name | Taxid | Max Score | Total Score | Query Cover | E value | Per. Ident | Acc. Len | Accession | Select for PSI blast | Used to build PSSM | Newly added |
  | --- | --- | --- | --- | --- | --- | --- | --- | --- | --- | --- | --- | --- | --- | --- |
  | 211Select seq ref|WP\_094674721.1| | DNA polymerase III subunit beta [Hydrocoleum sp. CS-953] | Hydrocoleum sp. CS-953 | NA | 1671698 | 49.1 | 49.1 | 94% | 0.007 | 13.60% | 353 | WP\_094674721.1(scored below threshold on previous iteration) | Select seq WP\_094674721.1 for PSI blast |  |  |
  | 212Select seq gb|MBA2248775.1| | DNA polymerase III subunit beta [Chitinophagaceae bacterium] | Chitinophagaceae bacterium | NA | 1869212 | 48.7 | 48.7 | 86% | 0.009 | 14.29% | 370 | MBA2248775.1(scored below threshold on previous iteration) | Select seq MBA2248775.1 for PSI blast |  |  |
  | 213Select seq gb|MBC7827772.1| | DNA polymerase III subunit beta [Chitinophagaceae bacterium] | Chitinophagaceae bacterium | NA | 1869212 | 48.7 | 48.7 | 86% | 0.010 | 15.15% | 371 | MBC7827772.1(scored below threshold on previous iteration) | Select seq MBC7827772.1 for PSI blast |  |  |
  | 214Select seq gb|RKZ11181.1| | hypothetical protein DRQ25\_00255 [Candidatus Fermentibacteria bacterium] | Candidatus Fermentibacteria bacterium | NA | 2044591 | 47.9 | 47.9 | 90% | 0.010 | 18.29% | 257 | RKZ11181.1(scored below threshold on previous iteration) | Select seq RKZ11181.1 for PSI blast |  |  |
  | 215Select seq gb|NBO73008.1| | hypothetical protein [bacterium] | bacterium | NA | 1869227 | 48.3 | 48.3 | 90% | 0.010 | 12.76% | 339 | NBO73008.1(scored below threshold on previous iteration) | Select seq NBO73008.1 for PSI blast |  |  |
  | 216Select seq gb|RKY86303.1| | hypothetical protein DRQ11\_08570 [candidate division KSB1 bacterium] | candidate division KSB1 bacterium | NA | 2172550 | 47.9 | 47.9 | 95% | 0.011 | 14.06% | 252 | RKY86303.1(scored below threshold on previous iteration) | Select seq RKY86303.1 for PSI blast |  |  |
  | 217Select seq ref|WP\_192160184.1| | DNA polymerase III subunit beta [Chroococcidiopsis sp. FACHB-1243] | Chroococcidiopsis sp. FACHB-1243 | NA | 2692781 | 48.3 | 48.3 | 88% | 0.012 | 16.39% | 449 | WP\_192160184.1(scored below threshold on previous iteration) | Select seq WP\_192160184.1 for PSI blast |  |  |
  | 218Select seq gb|RKX94156.1| | hypothetical protein DRP84\_07425 [Spirochaetes bacterium] | Spirochaetes bacterium | NA | 2202144 | 47.2 | 47.2 | 95% | 0.018 | 15.44% | 250 | RKX94156.1(scored below threshold on previous iteration) | Select seq RKX94156.1 for PSI blast |  |  |
  | 219Select seq ref|WP\_017717195.1| | DNA polymerase III subunit beta [Oscillatoria sp. PCC 10802] | Oscillatoria sp. PCC 10802 | NA | 1173028 | 47.5 | 47.5 | 88% | 0.019 | 14.11% | 396 | WP\_017717195.1(scored below threshold on previous iteration) | Select seq WP\_017717195.1 for PSI blast |  |  |
  | 220Select seq gb|NBP67062.1| | hypothetical protein [Bacteroidetes bacterium] | Bacteroidetes bacterium | NA | 1898104 | 47.5 | 47.5 | 90% | 0.019 | 12.76% | 358 | NBP67062.1(scored below threshold on previous iteration) | Select seq NBP67062.1 for PSI blast |  |  |
  | 221Select seq gb|NET24246.1| | DNA polymerase III subunit beta [Okeania sp. SIO1I7] | Okeania sp. SIO1I7 | NA | 2607772 | 47.2 | 47.2 | 94% | 0.028 | 15.75% | 384 | NET24246.1(scored below threshold on previous iteration) | Select seq NET24246.1 for PSI blast |  |  |
  | 222Select seq tpg|HHS22895.1| | TPA: DNA polymerase III subunit beta [bacterium] | bacterium | NA | 1869227 | 47.2 | 47.2 | 86% | 0.032 | 14.10% | 378 | HHS22895.1(scored below threshold on previous iteration) | Select seq HHS22895.1 for PSI blast |  |  |
  | 223Select seq ref|WP\_149868010.1| | DNA polymerase III subunit beta [Bacillus sp. S3] | Bacillus sp. S3 | NA | 486398 | 46.8 | 46.8 | 75% | 0.035 | 16.50% | 378 | WP\_149868010.1(scored below threshold on previous iteration) | Select seq WP\_149868010.1 for PSI blast |  |  |
  | 224Select seq emb|CDA47400.1| | dNA polymerase III subunit beta [Dialister sp. CAG:486] | Dialister sp. CAG:486 | NA | 1262870 | 46.8 | 46.8 | 83% | 0.039 | 15.77% | 370 | CDA47400.1(scored below threshold on previous iteration) | Select seq CDA47400.1 for PSI blast |  |  |
  | 225Select seq ref|WP\_169423815.1| | DNA polymerase III subunit beta [Pseudoflavitalea sp. G-6-1-2] | Pseudoflavitalea sp. G-6-1-2 | NA | 2728841 | 46.4 | 46.4 | 86% | 0.044 | 13.85% | 371 | WP\_169423815.1(scored below threshold on previous iteration) | Select seq WP\_169423815.1 for PSI blast |  |  |
  | 226Select seq ref|WP\_101650072.1| | DNA polymerase III subunit beta [Neobacillus cucumis] | Neobacillus cucumis | NA | 1740721 | 46.4 | 46.4 | 75% | 0.046 | 17.50% | 378 | WP\_101650072.1(scored below threshold on previous iteration) | Select seq WP\_101650072.1 for PSI blast |  |  |
- Graphic Summary

  - hover to see the title
  - click to show alignments
  - Show Conserved Domains
  - Alignment Scores
  - < 40
  - 40 - 50
  - 50 - 80
  - 80 - 200
  - >= 200
  - Help

    The graphic is an overview of the database sequences aligned to the query sequence. These are represented horizontal bars colored coded by score and showing the extent
    of the alignment on the query sequence. Separate aligned regions on the same database sequence are connected by a thin grey line.
    Mousing over an alignment shows the database sequence title. Clicking an alignment displays a box with more details about the alignment and
    link to the sequence alignment itself in the Alignments section of the report.

  - 226 sequences selected
  - Help

    To select sequences, go to the Descriptions tab

  ### Distribution of the top 226 Blast Hits on 226 subject sequences

  Query

  1

  50

  100

  150

  200

  250

  proliferating cell nuclear antigen (pcna) [bacterium]

  Score:283 Evalue:1.5e-92

  Accession:NBO72319.1

  Alignment

  proliferating cell nuclear antigen (pcna) [Flavobacteri..

  Score:282 Evalue:5.7e-92

  Accession:NBO60550.1

  Alignment

  proliferating cell nuclear antigen (pcna) [bacterium]

  Score:281 Evalue:9.6e-92

  Accession:NBO70988.1

  Alignment

  proliferating cell nuclear antigen (pcna) [Alphaproteob..

  Score:281 Evalue:9.9e-92

  Accession:NDB83927.1

  Alignment

  proliferating cell nuclear antigen (pcna) [bacterium]

  Score:281 Evalue:1.2e-91

  Accession:NBO72693.1

  Alignment

  proliferating cell nuclear antigen (pcna) [Microbacteri..

  Score:279 Evalue:4e-91

  Accession:WP\_162815250.1

  Alignment

  proliferating cell nuclear antigen (pcna) [bacterium]

  Score:279 Evalue:7e-91

  Accession:NBV77139.1

  Alignment

  proliferating cell nuclear antigen (pcna) [Actinobacter..

  Score:270 Evalue:1.5e-87

  Accession:NBU17043.1

  Alignment

  proliferating cell nuclear antigen (pcna) [bacterium]

  Score:268 Evalue:1.1e-86

  Accession:NDE16198.1

  Alignment

  proliferating cell nuclear antigen (pcna) [Actinobacter..

  Score:267 Evalue:2.7e-86

  Accession:MTA40656.1

  Alignment

  proliferating cell nuclear antigen (pcna) [bacterium]

  Score:266 Evalue:6.4e-86

  Accession:NBX51340.1

  Alignment

  proliferating cell nuclear antigen (pcna) [Alphaproteob..

  Score:264 Evalue:4.5e-85

  Accession:NDB83590.1

  Alignment

  proliferating cell nuclear antigen (pcna) [Acidimicrobi..

  Score:259 Evalue:5e-83

  Accession:MBG02288.1

  Alignment

  proliferating cell nuclear antigen (pcna) [Pelagibacter..

  Score:257 Evalue:2.5e-82

  Accession:MBC8296603.1

  Alignment

  proliferating cell nuclear antigen (pcna) [bacterium]

  Score:256 Evalue:4.9e-82

  Accession:NBX51392.1

  Alignment

  proliferating cell nuclear antigen (pcna) [Nitrospinae ..

  Score:256 Evalue:5e-82

  Accession:MBE18271.1

  Alignment

  proliferating cell nuclear antigen (pcna) [Flavobacteri..

  Score:256 Evalue:5.5e-82

  Accession:MAD50601.1

  Alignment

  proliferating cell nuclear antigen (pcna) [Candidatus M..

  Score:255 Evalue:1.5e-81

  Accession:MBD23483.1

  Alignment

  proliferating cell nuclear antigen (pcna) [Candidatus M..

  Score:254 Evalue:3e-81

  Accession:MBD23789.1

  Alignment

  proliferating cell nuclear antigen (pcna) [Chromatiales..

  Score:256 Evalue:3.6e-81

  Accession:OYV74989.1

  Alignment

  proliferating cell nuclear antigen (pcna) [Flavobacteri..

  Score:254 Evalue:1.2e-80

  Accession:MAD12048.1

  Alignment

  proliferating cell nuclear antigen (pcna) [Actinobacter..

  Score:253 Evalue:1.8e-80

  Accession:NBR60109.1

  Alignment

  proliferating cell nuclear antigen (pcna) [Actinobacter..

  Score:253 Evalue:1.9e-80

  Accession:NBO53670.1

  Alignment

  proliferating cell nuclear antigen (pcna) [Rhodobactera..

  Score:252 Evalue:3.5e-80

  Accession:PQM59040.1

  Alignment

  proliferating cell nuclear antigen (pcna) [Flavobacteri..

  Score:251 Evalue:6.5e-80

  Accession:RPG58329.1

  Alignment

  proliferating cell nuclear antigen (pcna) [bacterium]

  Score:249 Evalue:2.7e-79

  Accession:NDG29484.1

  Alignment

  proliferating cell nuclear antigen (pcna) [Crocinitomic..

  Score:249 Evalue:3.2e-79

  Accession:NBR15457.1

  Alignment

  proliferating cell nuclear antigen (pcna) [Actinobacter..

  Score:248 Evalue:1.1e-78

  Accession:NBR61302.1

  Alignment

  proliferating cell nuclear antigen (pcna) [Alphaproteob..

  Score:246 Evalue:1e-77

  Accession:OUU62278.1

  Alignment

  proliferating cell nuclear antigen (pcna) [bacterium]

  Score:246 Evalue:1.2e-77

  Accession:NBO71470.1

  Alignment

  proliferating cell nuclear antigen (pcna) [Actinobacter..

  Score:245 Evalue:2e-77

  Accession:NBT47941.1

  Alignment

  proliferating cell nuclear antigen (pcna) [Halobacterio..

  Score:243 Evalue:8.2e-77

  Accession:MAW08577.1

  Alignment

  proliferating cell nuclear antigen (pcna) [Flavobacteri..

  Score:243 Evalue:9.6e-77

  Accession:MAU36389.1

  Alignment

  proliferating cell nuclear antigen (pcna) [bacterium]

  Score:242 Evalue:1.2e-76

  Accession:NBP59184.1

  Alignment

  proliferating cell nuclear antigen (pcna) [Alphaproteob..

  Score:242 Evalue:2.1e-76

  Accession:NDB81722.1

  Alignment

  proliferating cell nuclear antigen (pcna) [bacterium]

  Score:241 Evalue:2.8e-76

  Accession:NBX48943.1

  Alignment

  proliferating cell nuclear antigen (pcna) [Flavobacteri..

  Score:239 Evalue:2.6e-75

  Accession:MAZ55206.1

  Alignment

  proliferating cell nuclear antigen (pcna) [bacterium]

  Score:239 Evalue:3e-75

  Accession:NBX48718.1

  Alignment

  proliferating cell nuclear antigen (pcna) [Candidatus E..

  Score:238 Evalue:4.3e-75

  Accession:OUU16001.1

  Alignment

  proliferating cell nuclear antigen (pcna) [Alphaproteob..

  Score:238 Evalue:6.3e-75

  Accession:NDB84018.1

  Alignment

  proliferating cell nuclear antigen (pcna) [Proteobacter..

  Score:238 Evalue:9.2e-75

  Accession:NDG72174.1

  Alignment

  proliferating cell nuclear antigen (pcna) [Candidatus P..

  Score:237 Evalue:1.1e-74

  Accession:OUU49664.1

  Alignment

  proliferating cell nuclear antigen (pcna) [Dehalococcoi..

  Score:235 Evalue:3.8e-74

  Accession:MAT63990.1

  Alignment

  proliferating cell nuclear antigen (pcna) [Porticoccace..

  Score:235 Evalue:8.3e-74

  Accession:MAE55769.1

  Alignment

  proliferating cell nuclear antigen (pcna) [Micrococcale..

  Score:235 Evalue:1.3e-73

  Accession:NBR26233.1

  Alignment

  proliferating cell nuclear antigen (pcna) [Rhodobactera..

  Score:235 Evalue:2.2e-73

  Accession:MAR51068.1

  Alignment

  proliferating cell nuclear antigen (pcna) [Chitinophagi..

  Score:232 Evalue:1e-72

  Accession:NDC41159.1

  Alignment

  proliferating cell nuclear antigen (pcna) [bacterium]

  Score:232 Evalue:1.3e-72

  Accession:MBI96915.1

  Alignment

  proliferating cell nuclear antigen (pcna) [Rhodobactera..

  Score:231 Evalue:2.3e-72

  Accession:MAT00017.1

  Alignment

  proliferating cell nuclear antigen (pcna) [Crocinitomic..

  Score:231 Evalue:5.1e-72

  Accession:NBR16174.1

  Alignment

  proliferating cell nuclear antigen (pcna) [Rhodospirill..

  Score:230 Evalue:5.5e-72

  Accession:MBP02103.1

  Alignment

  proliferating cell nuclear antigen (pcna) [Alteromonada..

  Score:230 Evalue:7.2e-72

  Accession:RPH19578.1

  Alignment

  proliferating cell nuclear antigen (pcna) [Acidimicrobi..

  Score:229 Evalue:1.4e-71

  Accession:MAF45402.1

  Alignment

  proliferating cell nuclear antigen (pcna) [bacterium]

  Score:229 Evalue:1.5e-71

  Accession:NBX51618.1

  Alignment

  proliferating cell nuclear antigen (pcna) [Verrucomicro..

  Score:228 Evalue:4.8e-71

  Accession:MAB60694.1

  Alignment

  TPA: proliferating cell nuclear antigen (pcna) [candida..

  Score:216 Evalue:2.2e-66

  Accession:HEN27889.1

  Alignment

  proliferating cell nuclear antigen (pcna) [Planctomycet..

  Score:210 Evalue:3.8e-64

  Accession:MAD20416.1

  Alignment

  proliferating cell nuclear antigen (pcna) [Planctomycet..

  Score:210 Evalue:6.2e-64

  Accession:MBJ75944.1

  Alignment

  proliferating cell nuclear antigen (pcna) [bacterium]

  Score:210 Evalue:1e-63

  Accession:NBP13185.1

  Alignment

  proliferating cell nuclear antigen (pcna) [Candidatus P..

  Score:208 Evalue:1.8e-63

  Accession:MBP37067.1

  Alignment

  proliferating cell nuclear antigen (pcna) [bacterium]

  Score:208 Evalue:3.5e-63

  Accession:NBU33707.1

  Alignment

  proliferating cell nuclear antigen (pcna) [Dehalococcoi..

  Score:207 Evalue:3.9e-63

  Accession:MBR74595.1

  Alignment

  proliferating cell nuclear antigen (pcna) [Magnetococca..

  Score:209 Evalue:1e-62

  Accession:MBA42970.1

  Alignment

  proliferating cell nuclear antigen (pcna) [bacterium]

  Score:207 Evalue:1.6e-62

  Accession:NDC95013.1

  Alignment

  proliferating cell nuclear antigen (pcna) [Candidatus R..

  Score:205 Evalue:2.3e-62

  Accession:OLD30892.1

  Alignment

  proliferating cell nuclear antigen (pcna) [Proteobacter..

  Score:205 Evalue:1.6e-61

  Accession:NBP00213.1

  Alignment

  proliferating cell nuclear antigen (pcna) [Gammaproteob..

  Score:202 Evalue:4.1e-61

  Accession:NIW10639.1

  Alignment

  proliferating cell nuclear antigen (pcna) [Candidatus R..

  Score:202 Evalue:4.2e-61

  Accession:OLD25330.1

  Alignment

  proliferating cell nuclear antigen (pcna) [Proteobacter..

  Score:201 Evalue:7.4e-60

  Accession:NDG27889.1

  Alignment

  proliferating cell nuclear antigen (pcna) [Actinobacter..

  Score:200 Evalue:9.5e-60

  Accession:NBR60072.1

  Alignment

  proliferating cell nuclear antigen (pcna) [Dehalococcoi..

  Score:198 Evalue:1.4e-59

  Accession:PCH85639.1

  Alignment

  proliferating cell nuclear antigen (pcna) [Opitutae bac..

  Score:196 Evalue:3.5e-56

  Accession:MBN37929.1

  Alignment

  proliferating cell nuclear antigen (pcna) [Gammaproteob..

  Score:190 Evalue:3.7e-56

  Accession:NCA73651.1

  Alignment

  proliferating cell nuclear antigen (pcna) [Clostridiale..

  Score:188 Evalue:1.8e-55

  Accession:NTV78099.1

  Alignment

  TPA: proliferating cell nuclear antigen (pcna) [bacteri..

  Score:188 Evalue:2.1e-55

  Accession:HEC38232.1

  Alignment

  TPA: proliferating cell nuclear antigen (pcna) [Bactero..

  Score:186 Evalue:5.3e-55

  Accession:HDQ16435.1

  Alignment

  proliferating cell nuclear antigen (pcna) [Candidatus L..

  Score:186 Evalue:1.2e-54

  Accession:MBI2034871.1

  Alignment

  hypothetical protein [Phycisphaerae bacterium]

  Score:185 Evalue:4.1e-54

  Accession:MAI67428.1

  Alignment

  proliferating cell nuclear antigen (pcna) [Bacteroidete..

  Score:184 Evalue:6.7e-54

  Accession:MBE9487910.1

  Alignment

  proliferating cell nuclear antigen (pcna) [bacterium]

  Score:183 Evalue:8.4e-54

  Accession:MRR35945.1

  Alignment

  polymerase sliding clamp protein [Parcubacteria group b..

  Score:183 Evalue:1.1e-53

  Accession:KKW29271.1

  Alignment

  DNA polymerase sliding clamp [Actinobacteria bacterium]

  Score:180 Evalue:3.1e-52

  Accession:NIS33632.1

  Alignment

  proliferating cell nuclear antigen (pcna) [Rickettsiale..

  Score:181 Evalue:3.8e-52

  Accession:MBB18791.1

  Alignment

  proliferating cell nuclear antigen (pcna) [Candidatus E..

  Score:178 Evalue:1.6e-51

  Accession:OUU15212.1

  Alignment

  proliferating cell nuclear antigen (pcna) [bacterium]

  Score:177 Evalue:2.6e-51

  Accession:NPA85815.1

  Alignment

  proliferating cell nuclear antigen (pcna) [Actinobacter..

  Score:177 Evalue:4.2e-51

  Accession:NBT48094.1

  Alignment

  proliferating cell nuclear antigen (pcna) [Chitinophagi..

  Score:175 Evalue:5.1e-51

  Accession:NDA64219.1

  Alignment

  TPA: DNA polymerase sliding clamp [Fervidobacterium pen..

  Score:56 Evalue:3.6e-07

  Accession:HGU52075.1

  Alignment

  hypothetical protein [bacterium]

  Score:177 Evalue:1.1e-50

  Accession:NDE18324.1

  Alignment

  proliferating cell nuclear antigen (pcna) [Alphaproteob..

  Score:176 Evalue:1.7e-50

  Accession:NDA89590.1

  Alignment

  proliferating cell nuclear antigen (pcna) [Rhodospirill..

  Score:175 Evalue:3.5e-50

  Accession:MAI14456.1

  Alignment

  proliferating cell nuclear antigen (pcna) [Rhodobactera..

  Score:175 Evalue:4e-50

  Accession:MAB04571.1

  Alignment

  proliferating cell nuclear antigen (pcna) [Bdellovibrio..

  Score:173 Evalue:1.6e-49

  Accession:MBN20023.1

  Alignment

  DNA polymerase sliding clamp [Candidatus Dadabacteria b..

  Score:171 Evalue:8.3e-49

  Accession:NIQ14317.1

  Alignment

  hypothetical protein [Chloroflexi bacterium]

  Score:168 Evalue:8.8e-48

  Accession:MBI63664.1

  Alignment

  DNA polymerase sliding clamp [Chloroflexi bacterium]

  Score:168 Evalue:9.6e-48

  Accession:MAE80883.1

  Alignment

  hypothetical protein [Candidatus Marinimicrobia bacteri..

  Score:165 Evalue:2e-46

  Accession:MBH51453.1

  Alignment

  hypothetical protein [Gammaproteobacteria bacterium]

  Score:165 Evalue:2.7e-46

  Accession:MAN39109.1

  Alignment

  proliferating cell nuclear antigen (pcna) [Candidatus M..

  Score:165 Evalue:3.4e-46

  Accession:MAP67643.1

  Alignment

  TPA: proliferating cell nuclear antigen (pcna) [Ignavib..

  Score:168 Evalue:9.2e-46

  Accession:HFO52032.1

  Alignment

  polymerase sliding clamp protein [Candidatus Woesebacte..

  Score:164 Evalue:1.4e-45

  Accession:KKR01610.1

  Alignment

  hypothetical protein [bacterium]

  Score:162 Evalue:1.8e-45

  Accession:NCS98517.1

  Alignment

  hypothetical protein [bacterium]

  Score:163 Evalue:2.9e-45

  Accession:NDE15318.1

  Alignment

  DNA polymerase sliding clamp [Candidatus Rokubacteria b..

  Score:160 Evalue:5.6e-45

  Accession:OLD29434.1

  Alignment

  proliferating cell nuclear antigen (pcna) [Pelagibacter..

  Score:162 Evalue:6.3e-45

  Accession:MBC8306902.1

  Alignment

  DNA polymerase sliding clamp [Gemmatimonadetes bacteriu..

  Score:160 Evalue:1.6e-44

  Accession:MBQ40860.1

  Alignment

  DNA polymerase sliding clamp [Candidatus Rokubacteria b..

  Score:158 Evalue:2.3e-44

  Accession:OLC90189.1

  Alignment

  proliferating cell nuclear antigen (pcna) [Propionibact..

  Score:159 Evalue:2.9e-44

  Accession:RYZ28222.1

  Alignment

  hypothetical protein [bacterium]

  Score:160 Evalue:3.2e-44

  Accession:MBE29542.1

  Alignment

  hypothetical protein [Rhodospirillaceae bacterium]

  Score:159 Evalue:7.3e-44

  Accession:MAI14441.1

  Alignment

  hypothetical protein [Rhodobiaceae bacterium]

  Score:158 Evalue:8.2e-44

  Accession:MAL04566.1

  Alignment

  proliferating cell nuclear antigen (pcna) [Flavobacteri..

  Score:159 Evalue:1.5e-43

  Accession:NDG54324.1

  Alignment

  proliferating cell nuclear antigen (pcna) [Candidatus P..

  Score:158 Evalue:1.6e-43

  Accession:MAV57173.1

  Alignment

  proliferating cell nuclear antigen (pcna) [Rhodobactera..

  Score:157 Evalue:2.4e-43

  Accession:MAR49569.1

  Alignment

  AAA family ATPase [bacterium]

  Score:75 Evalue:8.8e-12

  Accession:NBX51827.1

  Alignment

  proliferating cell nuclear antigen (pcna) [Alphaproteob..

  Score:155 Evalue:2.4e-43

  Accession:NDA89441.1

  Alignment

  TPA: hypothetical protein [Nautiliaceae bacterium]

  Score:156 Evalue:3e-43

  Accession:HIQ51620.1

  Alignment

  hypothetical protein [bacterium]

  Score:151 Evalue:7.1e-42

  Accession:NDG33583.1

  Alignment

  proliferating cell nuclear antigen (pcna) [Acinetobacte..

  Score:91 Evalue:4.6e-20

  Accession:WP\_147509764.1

  Alignment

  hypothetical protein [Alphaproteobacteria bacterium]

  Score:86 Evalue:3.7e-18

  Accession:NDB86628.1

  Alignment

  hypothetical protein [Alphaproteobacteria bacterium]

  Score:148 Evalue:3.2e-41

  Accession:NDB86579.1

  Alignment

  hypothetical protein [bacterium]

  Score:151 Evalue:4.2e-41

  Accession:NBP58287.1

  Alignment

  DNA polymerase sliding clamp [Bacteroidetes bacterium]

  Score:57 Evalue:1e-07

  Accession:MSP65089.1

  Alignment

  DNA polymerase sliding clamp [Candidatus Rokubacteria b..

  Score:149 Evalue:6.7e-41

  Accession:OLC55625.1

  Alignment

  hypothetical protein [Candidatus Marinimicrobia bacteri..

  Score:146 Evalue:3.8e-39

  Accession:MAP62052.1

  Alignment

  hypothetical protein DRI37\_10220 [Chloroflexi bacterium..

  Score:145 Evalue:5.2e-39

  Accession:RLC83458.1

  Alignment

  hypothetical protein EON71\_01370 [bacterium]

  Score:146 Evalue:3.7e-38

  Accession:RYX78042.1

  Alignment

  DNA polymerase sliding clamp [Nitrospiraceae bacterium]

  Score:143 Evalue:5.6e-38

  Accession:NIA11429.1

  Alignment

  hypothetical protein [Candidatus Marinimicrobia bacteri..

  Score:143 Evalue:8.6e-38

  Accession:MAR96207.1

  Alignment

  hypothetical protein [Candidatus Marinimicrobia bacteri..

  Score:140 Evalue:1.1e-36

  Accession:MAR96282.1

  Alignment

  hypothetical protein [Legionellales bacterium]

  Score:139 Evalue:1.8e-36

  Accession:MAJ82112.1

  Alignment

  TPA: proliferating cell nuclear antigen (pcna) [Proteob..

  Score:138 Evalue:4.9e-36

  Accession:HGO27509.1

  Alignment

  hypothetical protein A2W34\_06430 [Chloroflexi bacterium..

  Score:135 Evalue:7.6e-34

  Accession:OGO49108.1

  Alignment

  hypothetical protein [bacterium]

  Score:127 Evalue:2.6e-31

  Accession:MBI96709.1

  Alignment

  hypothetical protein [bacterium]

  Score:121 Evalue:5e-31

  Accession:NBS68524.1

  Alignment

  hypothetical protein [Crocinitomicaceae bacterium]

  Score:101 Evalue:3.4e-23

  Accession:NCA21076.1

  Alignment

  hypothetical protein [Flavobacteriales bacterium]

  Score:122 Evalue:1.2e-30

  Accession:MAU35866.1

  Alignment

  hypothetical protein [Proteobacteria bacterium]

  Score:52 Evalue:2.9e-05

  Accession:NBV35239.1

  Alignment

  hypothetical protein [Rhodobacteraceae bacterium]

  Score:118 Evalue:3.5e-28

  Accession:MAR17754.1

  Alignment

  DNA polymerase sliding clamp [Candidatus Latescibacteri..

  Score:112 Evalue:3.1e-27

  Accession:RKY57622.1

  Alignment

  hypothetical protein [Alphaproteobacteria bacterium]

  Score:89 Evalue:3.1e-19

  Accession:NDB84380.1

  Alignment

  proliferating cell nuclear antigen (pcna) [Acinetobacte..

  Score:110 Evalue:7.7e-27

  Accession:WP\_071289881.1

  Alignment

  DNA polymerase sliding clamp [Synergistetes bacterium]

  Score:108 Evalue:1.2e-25

  Accession:MBC7332724.1

  Alignment

  hypothetical protein EOO40\_09850 [Deltaproteobacteria b..

  Score:104 Evalue:8.6e-23

  Accession:RYF05887.1

  Alignment

  hypothetical protein [bacterium]

  Score:88 Evalue:8.7e-19

  Accession:NBS67530.1

  Alignment

  hypothetical protein EAZ85\_11740 [Bacteroidetes bacteri..

  Score:99 Evalue:1.1e-22

  Accession:TAE70583.1

  Alignment

  TPA: hypothetical protein [Fischerella sp.]

  Score:93 Evalue:6.4e-20

  Accession:HER14123.1

  Alignment

  hypothetical protein [Verrucomicrobia bacterium]

  Score:100 Evalue:7.3e-22

  Accession:NBS88271.1

  Alignment

  hypothetical protein [Flavobacteriia bacterium]

  Score:86 Evalue:5.3e-18

  Accession:NBY39677.1

  Alignment

  hypothetical protein [Flavobacteriaceae bacterium]

  Score:101 Evalue:8.9e-22

  Accession:MBH45101.1

  Alignment

  hypothetical protein BA864\_06935 [Desulfuromonadales ba..

  Score:99 Evalue:2.3e-21

  Accession:OEU74059.1

  Alignment

  TPA: DNA polymerase sliding clamp [Thermotoga sp.]

  Score:93 Evalue:4.4e-20

  Accession:HIP92434.1

  Alignment

  hypothetical protein [Microvirga massiliensis]

  Score:62 Evalue:7.1e-09

  Accession:WP\_156312854.1

  Alignment

  hypothetical protein [Flavobacteriia bacterium]

  Score:91 Evalue:1.3e-19

  Accession:NBS18941.1

  Alignment

  hypothetical protein [Acidimicrobiales bacterium]

  Score:93 Evalue:5.6e-18

  Accession:MBF6555506.1

  Alignment

  hypothetical protein DRI61\_15465 [Chloroflexi bacterium..

  Score:91 Evalue:9e-18

  Accession:RLC75119.1

  Alignment

  DNA polymerase sliding clamp [Bacteroidetes bacterium A..

  Score:89 Evalue:2.8e-17

  Accession:OPZ48648.1

  Alignment

  TPA: hypothetical protein [Aquificaceae bacterium]

  Score:84 Evalue:7.3e-17

  Accession:HID65709.1

  Alignment

  TPA: hypothetical protein [bacterium]

  Score:80 Evalue:1.2e-15

  Accession:HDT11972.1

  Alignment

  hypothetical protein DRP41\_07375 [Thermodesulfobacteria..

  Score:52 Evalue:7.1e-05

  Accession:RKX62390.1

  Alignment

  hypothetical protein [Flavobacteriales bacterium]

  Score:77 Evalue:1.6e-14

  Accession:MAU37716.1

  Alignment

  hypothetical protein BA864\_05090 [Desulfuromonadales ba..

  Score:77 Evalue:2.8e-13

  Accession:OEU73910.1

  Alignment

  hypothetical protein [bacterium]

  Score:77 Evalue:4.4e-13

  Accession:NBX48675.1

  Alignment

  hypothetical protein [Moritella sp. JT01]

  Score:77 Evalue:5.6e-13

  Accession:WP\_067050733.1

  Alignment

  hypothetical protein [Actinobacteria bacterium]

  Score:76 Evalue:2e-12

  Accession:MBE3094252.1

  Alignment

  hypothetical protein DRI69\_11125 [Bacteroidetes bacteri..

  Score:74 Evalue:3.5e-12

  Accession:RLD18005.1

  Alignment

  TPA: hypothetical protein [Maribacter sp.]

  Score:74 Evalue:4.7e-12

  Accession:HAI41630.1

  Alignment

  hypothetical protein [Verrucomicrobiales bacterium]

  Score:75 Evalue:4.8e-12

  Accession:MAD25962.1

  Alignment

  hypothetical protein DRH97\_00615 [Chloroflexi bacterium..

  Score:74 Evalue:5.5e-12

  Accession:RLC69169.1

  Alignment

  TPA: hypothetical protein [Desulfobacterales bacterium]

  Score:51 Evalue:0.0001

  Accession:HID70036.1

  Alignment

  hypothetical protein CBB97\_24650 [Candidatus Endolissoc..

  Score:69 Evalue:7e-11

  Accession:OUU14720.1

  Alignment

  TPA: hypothetical protein [bacterium]

  Score:71 Evalue:1.2e-10

  Accession:HEC40414.1

  Alignment

  DNA polymerase [Nitrosococcus oceani]

  Score:67 Evalue:1.4e-10

  Accession:WP\_036504199.1

  Alignment

  hypothetical protein [Candidatus Moranbacteria bacteriu..

  Score:70 Evalue:1.8e-10

  Accession:NCU42455.1

  Alignment

  hypothetical protein [Legionellales bacterium]

  Score:68 Evalue:1.3e-09

  Accession:MBL97255.1

  Alignment

  hypothetical protein [Cyanobium sp. ARS6]

  Score:66 Evalue:5.8e-09

  Accession:MAF41652.1

  Alignment

  hypothetical protein COA94\_02085 [Rickettsiales bacteri..

  Score:64 Evalue:2.2e-08

  Accession:PCJ29226.1

  Alignment

  TPA: hypothetical protein [Flavobacteriales bacterium]

  Score:62 Evalue:7.7e-08

  Accession:HIK66788.1

  Alignment

  hypothetical protein [Rickettsiales bacterium]

  Score:62 Evalue:1.2e-07

  Accession:MAY90458.1

  Alignment

  hypothetical protein [Alteromonas sp.]

  Score:59 Evalue:3.4e-07

  Accession:MAI63263.1

  Alignment

  hypothetical protein [Candidatus Poribacteria bacterium..

  Score:61 Evalue:3.6e-07

  Accession:MBP98812.1

  Alignment

  DNA polymerase sliding clamp [Bacteroidetes bacterium A..

  Score:60 Evalue:5.3e-07

  Accession:OQA51571.1

  Alignment

  hypothetical protein CBC65\_000905 [Rhodothermaceae bact..

  Score:58 Evalue:3.6e-06

  Accession:RPF82134.1

  Alignment

  hypothetical protein [Spirochaetia bacterium]

  Score:54 Evalue:8.8e-06

  Accession:NBK24950.1

  Alignment

  hypothetical protein [Candidatus Poribacteria bacterium..

  Score:55 Evalue:2.7e-05

  Accession:MBP98540.1

  Alignment

  hypothetical protein [Candidatus Nomurabacteria bacteri..

  Score:53 Evalue:6.4e-05

  Accession:NCU27524.1

  Alignment

  DNA polymerase III subunit beta [Armatimonas rosea]

  Score:55 Evalue:6.9e-05

  Accession:WP\_184202552.1

  Alignment

  DNA polymerase III subunit beta [Alkalinema sp. FACHB-9..

  Score:54 Evalue:0.00015

  Accession:WP\_199307260.1

  Alignment

  DNA polymerase III subunit beta [Alkalinema sp. FACHB-9..

  Score:54 Evalue:0.00019

  Accession:MBD2328424.1

  Alignment

  DNA polymerase III subunit beta [Oscillatoriales cyanob..

  Score:53 Evalue:0.00019

  Accession:OCR01436.1

  Alignment

  TPA: hypothetical protein [Lachnospiraceae bacterium]

  Score:53 Evalue:0.00022

  Accession:HBI72324.1

  Alignment

  hypothetical protein [Flavobacteriia bacterium]

  Score:47 Evalue:0.00038

  Accession:NBY39678.1

  Alignment

  DNA polymerase III subunit beta [Spirulina sp. DLM2.Bin..

  Score:52 Evalue:0.00042

  Accession:TVQ53692.1

  Alignment

  DNA polymerase III subunit beta [Phormidium ambiguum]

  Score:52 Evalue:0.0006

  Accession:WP\_084555250.1

  Alignment

  DNA polymerase III subunit beta [Spirulina subsalsa]

  Score:52 Evalue:0.00064

  Accession:WP\_017303270.1

  Alignment

  DNA polymerase III subunit beta [Phormidium ambiguum IA..

  Score:52 Evalue:0.00071

  Accession:OKH33175.1

  Alignment

  DNA polymerase III subunit beta [Okeania sp. SIO3I5]

  Score:51 Evalue:0.001

  Accession:NEQ38316.1

  Alignment

  DNA polymerase III subunit beta [Bacillus cihuensis]

  Score:51 Evalue:0.0012

  Accession:WP\_028393951.1

  Alignment

  DNA polymerase III subunit beta [Acidobacteriales bacte..

  Score:51 Evalue:0.0015

  Accession:MBI3645860.1

  Alignment

  DNA polymerase III subunit beta [Candidatus Koribacter ..

  Score:51 Evalue:0.0016

  Accession:MBI1740949.1

  Alignment

  DNA polymerase III subunit beta [Phormidium ambiguum]

  Score:51 Evalue:0.0017

  Accession:WP\_084555156.1

  Alignment

  DNA polymerase III subunit beta [Myxacorys almedinensis..

  Score:50 Evalue:0.0017

  Accession:WP\_162425412.1

  Alignment

  DNA polymerase III subunit beta [Chroococcidiopsis cuba..

  Score:51 Evalue:0.0018

  Accession:WP\_106166543.1

  Alignment

  DNA polymerase III subunit beta [Phormidium ambiguum IA..

  Score:51 Evalue:0.0018

  Accession:OKH36250.1

  Alignment

  DNA polymerase III subunit beta [Acidobacteria bacteriu..

  Score:50 Evalue:0.0019

  Accession:MBI3476530.1

  Alignment

  DNA polymerase III subunit beta [Acidobacteria bacteriu..

  Score:50 Evalue:0.0023

  Accession:PYX28173.1

  Alignment

  DNA polymerase III subunit beta [cyanobacterium TDX16]

  Score:50 Evalue:0.0028

  Accession:OWY67883.1

  Alignment

  DNA polymerase III subunit beta [Kamptonema sp. SIO4C4]

  Score:50 Evalue:0.0029

  Accession:NEO28492.1

  Alignment

  DNA polymerase III subunit beta [Thermobaculum terrenum..

  Score:50 Evalue:0.0031

  Accession:WP\_012874944.1

  Alignment

  DNA polymerase III subunit beta [Aphanothece hegewaldii..

  Score:49 Evalue:0.0034

  Accession:WP\_106458545.1

  Alignment

  DNA polymerase III subunit beta [Hydrocoleum sp. CS-953..

  Score:49 Evalue:0.0066

  Accession:WP\_094674721.1

  Alignment

  DNA polymerase III subunit beta [Chitinophagaceae bacte..

  Score:48 Evalue:0.0092

  Accession:MBA2248775.1

  Alignment

  DNA polymerase III subunit beta [Chitinophagaceae bacte..

  Score:48 Evalue:0.0098

  Accession:MBC7827772.1

  Alignment

  hypothetical protein DRQ25\_00255 [Candidatus Fermentiba..

  Score:47 Evalue:0.0098

  Accession:RKZ11181.1

  Alignment

  hypothetical protein [bacterium]

  Score:48 Evalue:0.01

  Accession:NBO73008.1

  Alignment

  hypothetical protein DRQ11\_08570 [candidate division KS..

  Score:47 Evalue:0.011

  Accession:RKY86303.1

  Alignment

  DNA polymerase III subunit beta [Chroococcidiopsis sp. ..

  Score:48 Evalue:0.012

  Accession:WP\_192160184.1

  Alignment

  hypothetical protein DRP84\_07425 [Spirochaetes bacteriu..

  Score:47 Evalue:0.018

  Accession:RKX94156.1

  Alignment

  DNA polymerase III subunit beta [Oscillatoria sp. PCC 1..

  Score:47 Evalue:0.019

  Accession:WP\_017717195.1

  Alignment

  hypothetical protein [Bacteroidetes bacterium]

  Score:47 Evalue:0.019

  Accession:NBP67062.1

  Alignment

  DNA polymerase III subunit beta [Okeania sp. SIO1I7]

  Score:47 Evalue:0.028

  Accession:NET24246.1

  Alignment

  TPA: DNA polymerase III subunit beta [bacterium]

  Score:47 Evalue:0.032

  Accession:HHS22895.1

  Alignment

  DNA polymerase III subunit beta [Bacillus sp. S3]

  Score:46 Evalue:0.035

  Accession:WP\_149868010.1

  Alignment

  dNA polymerase III subunit beta [Dialister sp. CAG:486]

  Score:46 Evalue:0.039

  Accession:CDA47400.1

  Alignment

  DNA polymerase III subunit beta [Pseudoflavitalea sp. G..

  Score:46 Evalue:0.044

  Accession:WP\_169423815.1

  Alignment

  DNA polymerase III subunit beta [Neobacillus cucumis]

  Score:46 Evalue:0.046

  Accession:WP\_101650072.1

  Alignment
- Alignments

  - Alignment view

    Pairwise
    Pairwise with dots for identities
    Query-anchored with dots for identities
    Query-anchored with letters for identities
    Flat query-anchored with dots for identities
    Flat query-anchored with letters for identities
  - CDS feature
  - Line length:

    60
    90
    120
    150
  - Help

    - Alignment view: Choose how to view alignments.
      The default "pairwise" view shows how each subject sequence aligns
      individually to the query sequence. The "query-anchored" view shows how
      all subject sequences align to the query sequence. For each view type,
      you can choose to show "identities" (matching residues) as letters or dots.
      more...
    - CDS feature: Show annotated coding region and translation.
      more...
    - Line length: Number of letters to show on one line in an alignment.
  - Restore defaults
  - Download
    - FASTA (complete sequence)
    - FASTA (aligned sequences)
    - GenBank (complete sequence)
    - Hit Table (text)
    - Hit Table (CSV)
    - Text
    - XML
    - ASN.1


  - 226 sequences selected
  - Help

    To select sequences, go to the Descriptions tab

  Loading alignment... for sequences gb|NBO72319.1|,gb|NBO60550.1|,gb|NBO70988.1|,gb|NDB83927.1|,gb|NBO72693.1| Reading indexes 1-5

  Download

  FASTA (complete sequence)

  FASTA (aligned sequences)

  GenBank (complete sequence)

  Text (aligned sequences)

  Continue
  Cancel

  GenPeptGraphics-used in PSSM

  Next
  Previous
  Descriptions

  proliferating cell nuclear antigen (pcna) [bacterium]

  Sequence ID: NBO72319.1Length: 272Number of Matches: 1

  Related Information

  Range 1: 15 to 266GenPeptGraphics

  Next Match
  Previous Match
  First Match

  Alignment statistics for match #1

  | Score | Expect | Method | Identities | Positives | Gaps | Frame |
  | --- | --- | --- | --- | --- | --- | --- |
  | 283 bits(725) | 1e-92() | Composition-based stats. | 78/254(31%) | 142/254(55%) | 3/254(1%) |  |

  Features:

  ```
  Query  1    MFEARLVQGSILKKVLEALKDLINEACWDISSSGVNLQSMDSSHVSLVQLTLRSEGFDTY  60
              + E + VQ + ++ ++EALK+++ EA  +  S G+ + ++D +H  LV L L ++ F+ Y
  Sbjct  15   LVEVKTVQAAAVRTLVEALKEILTEANLEFDSQGIKIVAVDETHTVLVYLRLHADRFENY  74

  Query  61   RCDRNLAMGVNLTSMSKILKCAGNEDIITLRAE-DNADTLALVFEAPNQEKVSDYEMKLM  119
               C     +GVN+  + K++K  GN D +TL    +N + L +  E   + +V+++ +KL 
  Sbjct  75   YCPVKHVLGVNMIYLFKLIKTMGNNDSLTLYLPANNPNKLGIRMENTEKSQVTNFFLKLF  134

  Query  120  DLDVEQLGIPEQEYSCVVKMPSGEFARICRDLSHIGDAVVISCAKDGVKFSASGELGNGN  179
              D DVE + IP   ++ ++ M S +F +ICRD++ +G+ + I+ +   + F   G+     
  Sbjct  135  DTDVEDISIPSLNFTSIIHMHSADFQKICRDMNILGEKMEITSSGSNLIFRCMGDFAEQE  194

  Query  180  IKLSQTSNVDKEEEAVTIEMNEPVQLTFALRYLNFFTKATPLSSTVTLSMSADVPLVVEY  239
                ++      K +   T   NE VQ  F L++L  FTK T L  ++ L +  D PL++ Y
  Sbjct  195  TVIADNQASMKVQTKGT--TNEIVQGVFQLKHLVLFTKCTTLCPSIELYLKNDYPLILRY  252

  Query  240  KIADMGHLKYYLAP  253
               +A++G +K  LAP
  Sbjct  253  MVANLGEVKLVLAP  266
  ```

  Download

  FASTA (complete sequence)

  FASTA (aligned sequences)

  GenBank (complete sequence)

  Text (aligned sequences)

  Continue
  Cancel

  GenPeptGraphics-used in PSSM

  Next
  Previous
  Descriptions

  proliferating cell nuclear antigen (pcna) [Flavobacteriia bacterium]

  Sequence ID: NBO60550.1Length: 272Number of Matches: 1

  Related Information

  Range 1: 13 to 266GenPeptGraphics

  Next Match
  Previous Match
  First Match

  Alignment statistics for match #1

  | Score | Expect | Method | Identities | Positives | Gaps | Frame |
  | --- | --- | --- | --- | --- | --- | --- |
  | 282 bits(721) | 6e-92() | Composition-based stats. | 82/256(32%) | 142/256(55%) | 5/256(1%) |  |

  Features:

  ```
  Query  1    MFEARLVQGSILKKVLEALKDLINEACWDISSSGVNLQSMDSSHVSLVQLTLRSEGFDTY  60
              +FE + VQ    + ++EALK+++ EA  +  S G+ + ++D +H  LV L L S+ F++Y
  Sbjct  13   LFEIKTVQSGAFRTLIEALKEILTEANLEFDSQGIKVMAVDETHTVLVYLRLHSDRFESY  72

  Query  61   RCDRNLAMGVNLTSMSKILKCAGNEDIITLRAED-NADTLALVFEAPNQEKVSDYEMKLM  119
               C +   +GVN+  + K++K  GN D +TL     N + L +  E   +  V++Y +KL 
  Sbjct  73   YCPQKYVLGVNMIYLFKLIKTMGNNDSLTLYLPSTNPNKLGIRMENSEKSTVTNYFLKLF  132

  Query  120  DLDVEQLGIPEQEYSCVVKMPSGEFARICRDLSHIGDA--VVISCAKDGVKFSASGELGN  177
              D DVE + IP   +  ++ +PS +  +ICRD++ +G+   V I+ +   + F   G+   
  Sbjct  133  DTDVEDIQIPNLNFPSIIHLPSADIQKICRDMNALGEKLDVEITSSGSDLIFKCMGDFAE  192

  Query  178  GNIKLSQTSNVDKEEEAVTIEMNEPVQLTFALRYLNFFTKATPLSSTVTLSMSADVPLVV  237
                  +S+ +N  K  +      NE VQ  F L++L  FTK T L  ++ L +  D PL++
  Sbjct  193  QETIISENNNTIKVHKNT--NTNEIVQGLFQLKHLVLFTKCTSLCPSIELYLKNDYPLIL  250

  Query  238  EYKIADMGHLKYYLAP  253
               Y +A++G +K  LAP
  Sbjct  251  RYTVANLGEVKLVLAP  266
  ```

  Download

  FASTA (complete sequence)

  FASTA (aligned sequences)

  GenBank (complete sequence)

  Text (aligned sequences)

  Continue
  Cancel

  GenPeptGraphics-used in PSSM

  Next
  Previous
  Descriptions

  proliferating cell nuclear antigen (pcna) [bacterium]

  Sequence ID: NBO70988.1Length: 276Number of Matches: 1

  - See 1 more title(s)
    See all Identical Proteins(IPG)

    proliferating cell nuclear antigen (pcna) [Bacteroidetes bacterium]

    Sequence ID: NBP64101.1Length: 276Number of Matches:

  Related Information

  Identical Proteins-Identical proteins to NBO70988.1

  Range 1: 12 to 267GenPeptGraphics

  Next Match
  Previous Match
  First Match

  Alignment statistics for match #1

  | Score | Expect | Method | Identities | Positives | Gaps | Frame |
  | --- | --- | --- | --- | --- | --- | --- |
  | 281 bits(720) | 1e-91() | Composition-based stats. | 82/256(32%) | 144/256(56%) | 3/256(1%) |  |

  Features:

  ```
  Query  1    MFEARLVQGSILKKVLEALKDLINEACWDISSSGVNLQSMDSSHVSLVQLTLRSEGFDTY  60
              +FE R VQ   ++ ++EALK+++ EA  +  S G+ + ++D +H  LV L L ++ F+TY
  Sbjct  12   LFEIRTVQAGAVRTLIEALKEILTEANLEFDSQGIKVMAVDETHTVLVYLRLHADRFETY  71

  Query  61   RCDRNLAMGVNLTSMSKILKCAGNEDIITLRAE-DNADTLALVFEAPNQEKVSDYEMKLM  119
               C     +GVN+  + K++K  GN D +TL     N + L +  E   +  V++Y +KL 
  Sbjct  72   YCPAKHVLGVNMIYLFKLIKTMGNNDNLTLYLPASNPNKLGIRMENSEKSTVTNYFLKLF  131

  Query  120  DLDVEQLGIPEQEYSCVVKMPSGEFARICRDLSHIGDA--VVISCAKDGVKFSASGELGN  177
              D DVE + IP   ++ ++ MPS +  +ICRD++ +G+   V I+ +   + F   G+   
  Sbjct  132  DTDVEDIQIPNLNFTSIIHMPSVDLQKICRDMNALGEKLDVEITSSGSDLIFKCMGDFAE  191

  Query  178  GNIKLSQTSNVDKEEEAVTIEMNEPVQLTFALRYLNFFTKATPLSSTVTLSMSADVPLVV  237
                  +S+ +N + +        +E VQ  F L++L  FTK T L  ++ L +  D+PL++
  Sbjct  192  QETIISEKNNSNMKVHKFGNGASEIVQGIFQLKHLVLFTKCTSLCPSIELYLKNDIPLIL  251

  Query  238  EYKIADMGHLKYYLAP  253
               Y +A++G +K  LAP
  Sbjct  252  RYTVANLGEVKLVLAP  267
  ```

  Download

  FASTA (complete sequence)

  FASTA (aligned sequences)

  GenBank (complete sequence)

  Text (aligned sequences)

  Continue
  Cancel

  GenPeptGraphics-used in PSSM

  Next
  Previous
  Descriptions

  proliferating cell nuclear antigen (pcna) [Alphaproteobacteria bacterium]

  Sequence ID: NDB83927.1Length: 273Number of Matches: 1

  Related Information

  Range 1: 16 to 267GenPeptGraphics

  Next Match
  Previous Match
  First Match

  Alignment statistics for match #1

  | Score | Expect | Method | Identities | Positives | Gaps | Frame |
  | --- | --- | --- | --- | --- | --- | --- |
  | 281 bits(720) | 1e-91() | Composition-based stats. | 77/254(30%) | 141/254(55%) | 3/254(1%) |  |

  Features:

  ```
  Query  1    MFEARLVQGSILKKVLEALKDLINEACWDISSSGVNLQSMDSSHVSLVQLTLRSEGFDTY  60
              +FE + VQ    + ++EALK+++ EA  +  S G+ + ++D +H  LV L L ++ F+ +
  Sbjct  16   LFEVKTVQSGAFRTLIEALKEILTEANLEFDSQGMKIVAVDETHTVLVYLRLHADRFENF  75

  Query  61   RCDRNLAMGVNLTSMSKILKCAGNEDIITLRAE-DNADTLALVFEAPNQEKVSDYEMKLM  119
               C     +GVN+  + K++K  GN D +TL    +N + L +  E   + +V+++ +KL 
  Sbjct  76   YCPVKHVLGVNMIYLFKLIKTMGNNDSLTLYLPANNPNKLGIRMENTEKSQVTNFFLKLF  135

  Query  120  DLDVEQLGIPEQEYSCVVKMPSGEFARICRDLSHIGDAVVISCAKDGVKFSASGELGNGN  179
              D DVE + IP   ++ ++ M S +F +ICRD++ +G+ + I+ +   + F   G+     
  Sbjct  136  DTDVEDISIPSLNFTSIIHMQSVDFQKICRDMNVLGEKMEITSSGTNLIFKCVGDFAEQE  195

  Query  180  IKLSQTSNVDKEEEAVTIEMNEPVQLTFALRYLNFFTKATPLSSTVTLSMSADVPLVVEY  239
                ++      K +   T   +E VQ  F L++L  FTK T L  ++ L +  D PL++ Y
  Sbjct  196  TVIADNQASMKVQTKGT--TSEIVQGVFQLKHLVLFTKCTTLCPSIELYLKNDYPLILRY  253

  Query  240  KIADMGHLKYYLAP  253
               +A++G +K  LAP
  Sbjct  254  MVANLGEVKLVLAP  267
  ```

  Download

  FASTA (complete sequence)

  FASTA (aligned sequences)

  GenBank (complete sequence)

  Text (aligned sequences)

  Continue
  Cancel

  GenPeptGraphics-used in PSSM

  Next
  Previous
  Descriptions

  proliferating cell nuclear antigen (pcna) [bacterium]

  Sequence ID: NBO72693.1Length: 273Number of Matches: 1

  - See 1 more title(s)
    See all Identical Proteins(IPG)

    proliferating cell nuclear antigen (pcna) [Bacteroidetes bacterium]

    Sequence ID: NBP65294.1Length: 273Number of Matches:

  Related Information

  Identical Proteins-Identical proteins to NBO72693.1

  Range 1: 16 to 267GenPeptGraphics

  Next Match
  Previous Match
  First Match

  Alignment statistics for match #1

  | Score | Expect | Method | Identities | Positives | Gaps | Frame |
  | --- | --- | --- | --- | --- | --- | --- |
  | 281 bits(719) | 1e-91() | Composition-based stats. | 74/254(29%) | 140/254(55%) | 3/254(1%) |  |

  Features:

  ```
  Query  1    MFEARLVQGSILKKVLEALKDLINEACWDISSSGVNLQSMDSSHVSLVQLTLRSEGFDTY  60
              +FE + VQ    + ++EALK+++ EA  +  S G+ + ++D +H  LV L L ++ F+ +
  Sbjct  16   LFEVKTVQSGAFRTLIEALKEILTEANLEFDSQGMKIVAVDETHTVLVYLRLHADRFENF  75

  Query  61   RCDRNLAMGVNLTSMSKILKCAGNEDIITLRAE-DNADTLALVFEAPNQEKVSDYEMKLM  119
               C     +GVN+  + K++K  GN D +T+    +N + L +  E   + +V+++ +KL 
  Sbjct  76   YCPVKHVLGVNMIYLFKLIKTMGNNDSLTIYLPANNPNKLGIRMENTEKSQVTNFFLKLF  135

  Query  120  DLDVEQLGIPEQEYSCVVKMPSGEFARICRDLSHIGDAVVISCAKDGVKFSASGELGNGN  179
              D DVE + IP   ++ ++ M S +F +ICRD++ +G+ + ++ +   + F   G+     
  Sbjct  136  DTDVEDINIPSLNFTSIIHMHSADFQKICRDMNVLGEKMEVTSSGTNLIFRCVGDFAEQE  195

  Query  180  IKLSQTSNVDKEEEAVTIEMNEPVQLTFALRYLNFFTKATPLSSTVTLSMSADVPLVVEY  239
                ++      K +   +    E VQ  F L++L  FTK T L  ++ L +  D PL++ Y
  Sbjct  196  TVIADNQASMKVQTKGS--TTEIVQGIFQLKHLVLFTKCTTLCPSIELYLKNDYPLILRY  253

  Query  240  KIADMGHLKYYLAP  253
               +A++G +K  LAP
  Sbjct  254  MVANLGEVKLVLAP  267
  ```

  ```

  ```
- Taxonomy

  ### Reports

  - 226 sequences selected
  - Help

    To select sequences, go to the Descriptions tab
  - Lineage

    Lineage Report

    | Organism | Blast Name | Score | Number of Hits | Description |
    | --- | --- | --- | --- | --- |
    | Bacteria | bacteria |  | 257 |  |
    | .unclassified Bacteria | bacteria |  | 44 |  |
    | ..bacterium | bacteria | 283 | 42 | bacterium hits |
    | ..bacterium TMED178 | bacteria | 175 | 2 | bacterium TMED178 hits |
    | .Flavobacteriia bacterium | CFB group bacteria | 282 | 5 | Flavobacteriia bacterium hits |
    | .Bacteroidetes bacterium | CFB group bacteria | 281 | 9 | Bacteroidetes bacterium hits |
    | .Alphaproteobacteria bacterium | a-proteobacteria | 281 | 9 | Alphaproteobacteria bacterium hits |
    | .Microbacterium arborescens | high GC Gram+ | 279 | 1 | Microbacterium arborescens hits |
    | .Actinobacteria bacterium | high GC Gram+ | 270 | 13 | Actinobacteria bacterium hits |
    | .Acidimicrobiaceae bacterium | actinobacteria | 259 | 2 | Acidimicrobiaceae bacterium hits |
    | .Pelagibacterales bacterium | a-proteobacteria | 257 | 2 | Pelagibacterales bacterium hits |
    | .Nitrospinae bacterium | bacteria | 256 | 1 | Nitrospinae bacterium hits |
    | .Flavobacteriales bacterium | CFB group bacteria | 256 | 6 | Flavobacteriales bacterium hits |
    | .Candidatus Marinimicrobia bacterium | bacteria | 255 | 8 | Candidatus Marinimicrobia bacterium hits |
    | .Chromatiales bacterium 21-64-14 | g-proteobacteria | 256 | 1 | Chromatiales bacterium 21-64-14 hits |
    | .Flavobacteriaceae bacterium | CFB group bacteria | 254 | 2 | Flavobacteriaceae bacterium hits |
    | .Rhodobacteraceae bacterium | a-proteobacteria | 252 | 6 | Rhodobacteraceae bacterium hits |
    | .Flavobacteriales bacterium TMED191 | CFB group bacteria | 251 | 1 | Flavobacteriales bacterium TMED191 hits |
    | .Crocinitomicaceae bacterium | CFB group bacteria | 249 | 4 | Crocinitomicaceae bacterium hits |
    | .Alphaproteobacteria bacterium TMED62 | a-proteobacteria | 246 | 1 | Alphaproteobacteria bacterium TMED62 hits |
    | .Halobacteriovoraceae bacterium | proteobacteria | 243 | 1 | Halobacteriovoraceae bacterium hits |
    | .Candidatus Endolissoclinum sp. TMED37 | a-proteobacteria | 238 | 3 | Candidatus Endolissoclinum sp. TMED37 hits |
    | .Proteobacteria bacterium | proteobacteria | 238 | 5 | Proteobacteria bacterium hits |
    | .Candidatus Puniceispirillum sp. TMED52 | a-proteobacteria | 237 | 1 | Candidatus Puniceispirillum sp. TMED52 hits |
    | .Rhodothermaceae bacterium TMED105 | CFB group bacteria | 237 | 2 | Rhodothermaceae bacterium TMED105 hits |
    | .Dehalococcoidia bacterium | GNS bacteria | 235 | 2 | Dehalococcoidia bacterium hits |
    | .Porticoccaceae bacterium | g-proteobacteria | 235 | 1 | Porticoccaceae bacterium hits |
    | .Micrococcales bacterium | high GC Gram+ | 235 | 1 | Micrococcales bacterium hits |
    | .Chitinophagia bacterium | CFB group bacteria | 232 | 2 | Chitinophagia bacterium hits |
    | .Aestuariivita sp. | a-proteobacteria | 231 | 1 | Aestuariivita sp. hits |
    | .Rhodospirillaceae bacterium | a-proteobacteria | 230 | 3 | Rhodospirillaceae bacterium hits |
    | .Alteromonadaceae bacterium TMED7 | g-proteobacteria | 230 | 1 | Alteromonadaceae bacterium TMED7 hits |
    | .Verrucomicrobiales bacterium | verrucomicrobia | 228 | 2 | Verrucomicrobiales bacterium hits |
    | .candidate division WOR-3 bacterium | bacteria | 216 | 1 | candidate division WOR-3 bacterium hits |
    | .Planctomycetaceae bacterium | planctomycetes | 210 | 1 | Planctomycetaceae bacterium hits |
    | .Planctomycetes bacterium | bacteria | 210 | 1 | Planctomycetes bacterium hits |
    | .Candidatus Pelagibacter sp. | a-proteobacteria | 208 | 2 | Candidatus Pelagibacter sp. hits |
    | .Dehalococcoidaceae bacterium | GNS bacteria | 207 | 1 | Dehalococcoidaceae bacterium hits |
    | .Magnetococcales bacterium | a-proteobacteria | 209 | 1 | Magnetococcales bacterium hits |
    | .Candidatus Rokubacteria bacterium 13\_1\_40CM\_2\_70\_45 | bacteria | 205 | 3 | Candidatus Rokubacteria bacterium 13\_1\_40CM\_2\_70\_45 hits |
    | .Gammaproteobacteria bacterium | g-proteobacteria | 202 | 3 | Gammaproteobacteria bacterium hits |
    | .Opitutae bacterium | verrucomicrobia | 196 | 1 | Opitutae bacterium hits |
    | .Clostridiales bacterium | firmicutes | 188 | 1 | Clostridiales bacterium hits |
    | .Candidatus Levybacteria bacterium | bacteria | 186 | 1 | Candidatus Levybacteria bacterium hits |
    | .Phycisphaerae bacterium | bacteria | 185 | 1 | Phycisphaerae bacterium hits |
    | .Parcubacteria group bacterium GW2011\_GWB1\_52\_7 | bacteria | 183 | 1 | Parcubacteria group bacterium GW2011\_GWB1\_52\_7 hits |
    | .Rickettsiales bacterium | a-proteobacteria | 181 | 3 | Rickettsiales bacterium hits |
    | .Bdellovibrionaceae bacterium | proteobacteria | 173 | 1 | Bdellovibrionaceae bacterium hits |
    | .Candidatus Dadabacteria bacterium | bacteria | 171 | 1 | Candidatus Dadabacteria bacterium hits |
    | .Chloroflexi bacterium | GNS bacteria | 168 | 5 | Chloroflexi bacterium hits |
    | .Ignavibacteria bacterium | bacteria | 168 | 1 | Ignavibacteria bacterium hits |
    | .Candidatus Woesebacteria bacterium GW2011\_GWB1\_39\_12 | bacteria | 164 | 1 | Candidatus Woesebacteria bacterium GW2011\_GWB1\_39\_12 hits |
    | .Gemmatimonadetes bacterium | bacteria | 160 | 1 | Gemmatimonadetes bacterium hits |
    | .Candidatus Rokubacteria bacterium 13\_1\_40CM\_3\_69\_38 | bacteria | 158 | 1 | Candidatus Rokubacteria bacterium 13\_1\_40CM\_3\_69\_38 hits |
    | .Propionibacteriaceae bacterium | high GC Gram+ | 159 | 1 | Propionibacteriaceae bacterium hits |
    | .Rhodobiaceae bacterium | a-proteobacteria | 158 | 1 | Rhodobiaceae bacterium hits |
    | .Nautiliaceae bacterium | e-proteobacteria | 156 | 1 | Nautiliaceae bacterium hits |
    | .Candidatus Rokubacteria bacterium 13\_1\_40CM\_4\_69\_39 | bacteria | 149 | 1 | Candidatus Rokubacteria bacterium 13\_1\_40CM\_4\_69\_39 hits |
    | .Nitrospiraceae bacterium | bacteria | 143 | 1 | Nitrospiraceae bacterium hits |
    | .Legionellales bacterium | g-proteobacteria | 139 | 2 | Legionellales bacterium hits |
    | .Chloroflexi bacterium RBG\_16\_64\_32 | GNS bacteria | 135 | 1 | Chloroflexi bacterium RBG\_16\_64\_32 hits |
    | .Candidatus Latescibacteria bacterium | bacteria | 112 | 1 | Candidatus Latescibacteria bacterium hits |
    | .Acinetobacter baumannii | g-proteobacteria | 110 | 3 | Acinetobacter baumannii hits |
    | .Synergistetes bacterium | bacteria | 108 | 1 | Synergistetes bacterium hits |
    | .Deltaproteobacteria bacterium | d-proteobacteria | 104 | 1 | Deltaproteobacteria bacterium hits |
    | .Verrucomicrobia bacterium | verrucomicrobia | 100 | 1 | Verrucomicrobia bacterium hits |
    | .Desulfuromonadales bacterium C00003093 | d-proteobacteria | 99 | 2 | Desulfuromonadales bacterium C00003093 hits |
    | .Thermotoga sp. | thermotogales | 93.8 | 1 | Thermotoga sp. hits |
    | .Fischerella sp. | cyanobacteria | 93.4 | 1 | Fischerella sp. hits |
    | .Acidimicrobiales bacterium | actinobacteria | 93.4 | 1 | Acidimicrobiales bacterium hits |
    | .Bacteroidetes bacterium ADurb.BinA104 | CFB group bacteria | 89.1 | 1 | Bacteroidetes bacterium ADurb.BinA104 hits |
    | .Aquificaceae bacterium | aquificales | 84.9 | 1 | Aquificaceae bacterium hits |
    | .Moritella sp. JT01 | g-proteobacteria | 77.6 | 2 | Moritella sp. JT01 hits |
    | .Maribacter sp. | CFB group bacteria | 74.9 | 1 | Maribacter sp. hits |
    | .Nitrosococcus oceani | g-proteobacteria | 67.2 | 2 | Nitrosococcus oceani hits |
    | .Candidatus Moranbacteria bacterium | bacteria | 70.3 | 1 | Candidatus Moranbacteria bacterium hits |
    | .Cyanobium sp. ARS6 | cyanobacteria | 66.0 | 1 | Cyanobium sp. ARS6 hits |
    | .Microvirga massiliensis | a-proteobacteria | 62.6 | 1 | Microvirga massiliensis hits |
    | .Alteromonas sp. | g-proteobacteria | 59.9 | 1 | Alteromonas sp. hits |
    | .Candidatus Poribacteria bacterium | bacteria | 61.0 | 2 | Candidatus Poribacteria bacterium hits |
    | .Fervidobacterium pennivorans | thermotogales | 56.8 | 1 | Fervidobacterium pennivorans hits |
    | .Bacteroidetes bacterium ADurb.Bin302 | CFB group bacteria | 60.3 | 1 | Bacteroidetes bacterium ADurb.Bin302 hits |
    | .Spirochaetia bacterium | bacteria | 54.5 | 1 | Spirochaetia bacterium hits |
    | .Candidatus Nomurabacteria bacterium | bacteria | 53.7 | 1 | Candidatus Nomurabacteria bacterium hits |
    | .Armatimonas rosea | bacteria | 55.3 | 2 | Armatimonas rosea hits |
    | .Thermodesulfobacteria bacterium | bacteria | 52.2 | 1 | Thermodesulfobacteria bacterium hits |
    | .Desulfobacterales bacterium | d-proteobacteria | 51.4 | 1 | Desulfobacterales bacterium hits |
    | .Alkalinema sp. FACHB-956 | cyanobacteria | 54.5 | 2 | Alkalinema sp. FACHB-956 hits |
    | .Oscillatoriales cyanobacterium USR001 | cyanobacteria | 53.7 | 1 | Oscillatoriales cyanobacterium USR001 hits |
    | .Lachnospiraceae bacterium | firmicutes | 53.7 | 1 | Lachnospiraceae bacterium hits |
    | .Spirulina sp. DLM2.Bin59 | cyanobacteria | 52.6 | 1 | Spirulina sp. DLM2.Bin59 hits |
    | .Phormidium ambiguum | cyanobacteria | 52.6 | 2 | Phormidium ambiguum hits |
    | .Spirulina subsalsa | cyanobacteria | 52.2 | 1 | Spirulina subsalsa hits |
    | .Phormidium ambiguum IAM M-71 | cyanobacteria | 52.2 | 2 | Phormidium ambiguum IAM M-71 hits |
    | .Okeania sp. SIO3I5 | cyanobacteria | 51.4 | 1 | Okeania sp. SIO3I5 hits |
    | .Bacillus cihuensis | firmicutes | 51.4 | 1 | Bacillus cihuensis hits |
    | .Acidobacteriales bacterium | bacteria | 51.0 | 1 | Acidobacteriales bacterium hits |
    | .Candidatus Koribacter versatilis | bacteria | 51.0 | 1 | Candidatus Koribacter versatilis hits |
    | .Myxacorys almedinensis | cyanobacteria | 50.2 | 1 | Myxacorys almedinensis hits |
    | .Myxacorys almedinensis A | cyanobacteria | 50.2 | 1 | Myxacorys almedinensis A hits |
    | .Chroococcidiopsis cubana | cyanobacteria | 51.0 | 1 | Chroococcidiopsis cubana hits |
    | .Chroococcidiopsis cubana CCALA 043 | cyanobacteria | 51.0 | 1 | Chroococcidiopsis cubana CCALA 043 hits |
    | .Chroococcidiopsis cubana SAG 39.79 | cyanobacteria | 51.0 | 1 | Chroococcidiopsis cubana SAG 39.79 hits |
    | .Acidobacteria bacterium | bacteria | 50.6 | 2 | Acidobacteria bacterium hits |
    | .cyanobacterium TDX16 | cyanobacteria | 50.2 | 1 | cyanobacterium TDX16 hits |
    | .Kamptonema sp. SIO4C4 | cyanobacteria | 50.2 | 1 | Kamptonema sp. SIO4C4 hits |
    | .Thermobaculum terrenum | bacteria | 50.2 | 1 | Thermobaculum terrenum hits |
    | .Thermobaculum terrenum ATCC BAA-798 | bacteria | 50.2 | 1 | Thermobaculum terrenum ATCC BAA-798 hits |
    | .Aphanothece hegewaldii | cyanobacteria | 49.9 | 1 | Aphanothece hegewaldii hits |
    | .Aphanothece hegewaldii CCALA 016 | cyanobacteria | 49.9 | 1 | Aphanothece hegewaldii CCALA 016 hits |
    | .Hydrocoleum sp. CS-953 | cyanobacteria | 49.1 | 1 | Hydrocoleum sp. CS-953 hits |
    | .Chitinophagaceae bacterium | CFB group bacteria | 48.7 | 2 | Chitinophagaceae bacterium hits |
    | .Candidatus Fermentibacteria bacterium | bacteria | 47.9 | 1 | Candidatus Fermentibacteria bacterium hits |
    | .candidate division KSB1 bacterium | bacteria | 47.9 | 1 | candidate division KSB1 bacterium hits |
    | .Chroococcidiopsis sp. FACHB-1243 | cyanobacteria | 48.3 | 2 | Chroococcidiopsis sp. FACHB-1243 hits |
    | .Spirochaetes bacterium | bacteria | 47.2 | 1 | Spirochaetes bacterium hits |
    | .Oscillatoria sp. PCC 10802 | cyanobacteria | 47.5 | 1 | Oscillatoria sp. PCC 10802 hits |
    | .Okeania sp. SIO1I7 | cyanobacteria | 47.2 | 1 | Okeania sp. SIO1I7 hits |
    | .Bacillus sp. S3 | firmicutes | 46.8 | 2 | Bacillus sp. S3 hits |
    | .Dialister sp. CAG:486 | firmicutes | 46.8 | 1 | Dialister sp. CAG:486 hits |
    | .Pseudoflavitalea sp. G-6-1-2 | CFB group bacteria | 46.4 | 2 | Pseudoflavitalea sp. G-6-1-2 hits |
    | .Neobacillus cucumis | firmicutes | 46.4 | 2 | Neobacillus cucumis hits |
  - Organism

    Organism Report

    | Description | Score | E value | Accession |
    | --- | --- | --- | --- |
    | bacterium [bacteria]  Next Previous First | | | |
    | --- | --- | --- | --- |
    | proliferating cell nuclear antigen (pcna) [bacterium] | 283 | 1e-92 | NBO72319 |
    | proliferating cell nuclear antigen (pcna) [bacterium] | 281 | 1e-91 | NBO70988 |
    | proliferating cell nuclear antigen (pcna) [bacterium] | 281 | 1e-91 | NBO72693 |
    | proliferating cell nuclear antigen (pcna) [bacterium] | 279 | 7e-91 | NBV77139 |
    | proliferating cell nuclear antigen (pcna) [bacterium] | 268 | 1e-86 | NDE16198 |
    | proliferating cell nuclear antigen (pcna) [bacterium] | 266 | 6e-86 | NBX51340 |
    | proliferating cell nuclear antigen (pcna) [bacterium] | 256 | 5e-82 | NBX51392 |
    | proliferating cell nuclear antigen (pcna) [bacterium] | 249 | 3e-79 | NDG29484 |
    | proliferating cell nuclear antigen (pcna) [bacterium] | 246 | 1e-77 | NBO71470 |
    | proliferating cell nuclear antigen (pcna) [bacterium] | 242 | 1e-76 | NBP59184 |
    | proliferating cell nuclear antigen (pcna) [bacterium] | 241 | 3e-76 | NBX48943 |
    | proliferating cell nuclear antigen (pcna) [bacterium] | 239 | 3e-75 | NBX48718 |
    | proliferating cell nuclear antigen (pcna) [bacterium] | 232 | 1e-72 | MBI96915 |
    | proliferating cell nuclear antigen (pcna) [bacterium] | 229 | 2e-71 | NBX51618 |
    | proliferating cell nuclear antigen (pcna) [bacterium] | 210 | 1e-63 | NBP13185 |
    | proliferating cell nuclear antigen (pcna) [bacterium] | 208 | 4e-63 | NBU33707 |
    | proliferating cell nuclear antigen (pcna) [bacterium] | 208 | 4e-63 | NDC93759 |
    | proliferating cell nuclear antigen (pcna) [bacterium] | 208 | 4e-63 | NDD83092 |
    | proliferating cell nuclear antigen (pcna) [bacterium] | 208 | 4e-63 | NDG29507 |
    | proliferating cell nuclear antigen (pcna) [bacterium] | 207 | 2e-62 | NDC95013 |
    | proliferating cell nuclear antigen (pcna) [bacterium] | 207 | 2e-62 | NDD83476 |
    | proliferating cell nuclear antigen (pcna) [bacterium] | 207 | 2e-62 | NDG30839 |
    | proliferating cell nuclear antigen (pcna) [bacterium] | 205 | 2e-61 | NBP13601 |
    | proliferating cell nuclear antigen (pcna) [bacterium] | 188 | 2e-55 | HEC38232 |
    | proliferating cell nuclear antigen (pcna) [bacterium] | 183 | 8e-54 | MRR35945 |
    | proliferating cell nuclear antigen (pcna) [bacterium] | 177 | 3e-51 | NPA85815 |
    | hypothetical protein [bacterium] | 177 | 1e-50 | NDE18324 |
    | hypothetical protein [bacterium] | 162 | 2e-45 | NCS98517 |
    | hypothetical protein [bacterium] | 163 | 3e-45 | NDE15318 |
    | hypothetical protein [bacterium] | 160 | 3e-44 | MBE29542 |
    | hypothetical protein [bacterium] | 151 | 7e-42 | NDG33583 |
    | hypothetical protein [bacterium] | 151 | 4e-41 | NBP58287 |
    | hypothetical protein EON71\_01370, partial [bacterium] | 146 | 4e-38 | RYX78042 |
    | hypothetical protein [bacterium] | 127 | 3e-31 | MBI96709 |
    | hypothetical protein [bacterium] | 121 | 5e-31 | NBS68524 |
    | hypothetical protein [bacterium] | 88.8 | 9e-19 | NBS67530 |
    | hypothetical protein [bacterium] | 80.7 | 1e-15 | HDT11972 |
    | hypothetical protein [bacterium] | 77.6 | 4e-13 | NBX48675 |
    | AAA family ATPase [bacterium] | 75.3 | 9e-12 | NBX51827 |
    | hypothetical protein [bacterium] | 71.0 | 1e-10 | HEC40414 |
    | hypothetical protein [bacterium] | 48.3 | 0.010 | NBO73008 |
    | DNA polymerase III subunit beta [bacterium] | 47.2 | 0.032 | HHS22895 |
    | Flavobacteriia bacterium [CFB group bacteria]  Next Previous First | | | |
    | proliferating cell nuclear antigen (pcna) [Flavobacteriia bacterium] | 282 | 6e-92 | NBO60550 |
    | proliferating cell nuclear antigen (pcna) [Flavobacteriia bacterium] | 159 | 2e-43 | NDG54324 |
    | hypothetical protein [Flavobacteriia bacterium] | 91.8 | 1e-19 | NBS18941 |
    | hypothetical protein [Flavobacteriia bacterium] | 86.8 | 5e-18 | NBY39677 |
    | hypothetical protein [Flavobacteriia bacterium] | 47.9 | 4e-04 | NBY39678 |
    | Bacteroidetes bacterium [CFB group bacteria]  Next Previous First | | | |
    | proliferating cell nuclear antigen (pcna) [Bacteroidetes bacterium] | 281 | 1e-91 | NBP64101 |
    | proliferating cell nuclear antigen (pcna) [Bacteroidetes bacterium] | 281 | 1e-91 | NBP65294 |
    | proliferating cell nuclear antigen (pcna) [Bacteroidetes bacterium] | 246 | 1e-77 | NBP65516 |
    | proliferating cell nuclear antigen (pcna) [Bacteroidetes bacterium] | 186 | 5e-55 | HDQ16435 |
    | proliferating cell nuclear antigen (pcna) [Bacteroidetes bacterium] | 184 | 7e-54 | MBE9487910 |
    | hypothetical protein EAZ85\_11740 [Bacteroidetes bacterium] | 99.2 | 1e-22 | TAE70583 |
    | hypothetical protein DRI69\_11125 [Bacteroidetes bacterium] | 74.9 | 3e-12 | RLD18005 |
    | DNA polymerase sliding clamp [Bacteroidetes bacterium] | 57.9 | 1e-07 | MSP65089 |
    | hypothetical protein [Bacteroidetes bacterium] | 47.5 | 0.019 | NBP67062 |
    | Alphaproteobacteria bacterium [a-proteobacteria]  Next Previous First | | | |
    | proliferating cell nuclear antigen (pcna) [Alphaproteobacteria bacterium] | 281 | 1e-91 | NDB83927 |
    | proliferating cell nuclear antigen (pcna) [Alphaproteobacteria bacterium] | 264 | 4e-85 | NDB83590 |
    | proliferating cell nuclear antigen (pcna) [Alphaproteobacteria bacterium] | 242 | 2e-76 | NDB81722 |
    | proliferating cell nuclear antigen (pcna) [Alphaproteobacteria bacterium] | 238 | 6e-75 | NDB84018 |
    | proliferating cell nuclear antigen (pcna) [Alphaproteobacteria bacterium] | 176 | 2e-50 | NDA89590 |
    | proliferating cell nuclear antigen (pcna) [Alphaproteobacteria bacterium] | 155 | 2e-43 | NDA89441 |
    | hypothetical protein [Alphaproteobacteria bacterium] | 148 | 3e-41 | NDB86579 |
    | hypothetical protein [Alphaproteobacteria bacterium] | 89.9 | 3e-19 | NDB84380 |
    | hypothetical protein [Alphaproteobacteria bacterium] | 86.8 | 4e-18 | NDB86628 |
    | Microbacterium arborescens [high GC Gram+]  Next Previous First | | | |
    | proliferating cell nuclear antigen (pcna) [Microbacterium arborescens] | 279 | 4e-91 | WP\_162815250 |
    | Actinobacteria bacterium [high GC Gram+]  Next Previous First | | | |
    | proliferating cell nuclear antigen (pcna) [Actinobacteria bacterium] | 270 | 1e-87 | NBU17043 |
    | proliferating cell nuclear antigen (pcna) [Actinobacteria bacterium] | 267 | 3e-86 | MTA40656 |
    | proliferating cell nuclear antigen (pcna) [Actinobacteria bacterium] | 253 | 2e-80 | NBR60109 |
    | proliferating cell nuclear antigen (pcna) [Actinobacteria bacterium] | 253 | 2e-80 | NBO53670 |
    | proliferating cell nuclear antigen (pcna) [Actinobacteria bacterium] | 248 | 1e-78 | NBR61302 |
    | proliferating cell nuclear antigen (pcna) [Actinobacteria bacterium] | 245 | 2e-77 | NBT47941 |
    | proliferating cell nuclear antigen (pcna) [Actinobacteria bacterium] | 200 | 1e-59 | NBR60072 |
    | DNA polymerase sliding clamp [Actinobacteria bacterium] | 180 | 3e-52 | NIS33632 |
    | DNA polymerase sliding clamp [Actinobacteria bacterium] | 180 | 3e-52 | NIU68491 |
    | DNA polymerase sliding clamp [Actinobacteria bacterium] | 180 | 3e-52 | NIW30316 |
    | DNA polymerase sliding clamp [Actinobacteria bacterium] | 180 | 3e-52 | NIX22740 |
    | proliferating cell nuclear antigen (pcna) [Actinobacteria bacterium] | 177 | 4e-51 | NBT48094 |
    | hypothetical protein [Actinobacteria bacterium] | 76.1 | 2e-12 | MBE3094252 |
    | Acidimicrobiaceae bacterium [actinobacteria]  Next Previous First | | | |
    | proliferating cell nuclear antigen (pcna) [Acidimicrobiaceae bacterium] | 259 | 5e-83 | MBG02288 |
    | proliferating cell nuclear antigen (pcna) [Acidimicrobiaceae bacterium] | 229 | 1e-71 | MAF45402 |
    | Pelagibacterales bacterium [a-proteobacteria]  Next Previous First | | | |
    | proliferating cell nuclear antigen (pcna) [Pelagibacterales bacterium] | 257 | 3e-82 | MBC8296603 |
    | proliferating cell nuclear antigen (pcna) [Pelagibacterales bacterium] | 162 | 6e-45 | MBC8306902 |
    | Nitrospinae bacterium [bacteria]  Next Previous First | | | |
    | proliferating cell nuclear antigen (pcna) [Nitrospinae bacterium] | 256 | 5e-82 | MBE18271 |
    | Flavobacteriales bacterium [CFB group bacteria]  Next Previous First | | | |
    | proliferating cell nuclear antigen (pcna) [Flavobacteriales bacterium] | 256 | 6e-82 | MAD50601 |
    | proliferating cell nuclear antigen (pcna) [Flavobacteriales bacterium] | 243 | 1e-76 | MAU36389 |
    | proliferating cell nuclear antigen (pcna) [Flavobacteriales bacterium] | 239 | 3e-75 | MAZ55206 |
    | hypothetical protein [Flavobacteriales bacterium] | 122 | 1e-30 | MAU35866 |
    | hypothetical protein [Flavobacteriales bacterium] | 78.0 | 2e-14 | MAU37716 |
    | hypothetical protein [Flavobacteriales bacterium] | 63.0 | 8e-08 | HIK66788 |
    | Candidatus Marinimicrobia bacterium [bacteria]  Next Previous First | | | |
    | proliferating cell nuclear antigen (pcna) [Candidatus Marinimicrobia bacterium] | 255 | 1e-81 | MBD23483 |
    | proliferating cell nuclear antigen (pcna) [Candidatus Marinimicrobia bacterium] | 254 | 3e-81 | MBD23789 |
    | hypothetical protein [Candidatus Marinimicrobia bacterium] | 165 | 2e-46 | MBH51453 |
    | proliferating cell nuclear antigen (pcna) [Candidatus Marinimicrobia bacterium] | 165 | 3e-46 | MAP67643 |
    | hypothetical protein [Candidatus Marinimicrobia bacterium] | 146 | 4e-39 | MAP62052 |
    | hypothetical protein [Candidatus Marinimicrobia bacterium] | 143 | 9e-38 | MAR96207 |
    | hypothetical protein [Candidatus Marinimicrobia bacterium] | 143 | 9e-38 | MAR97466 |
    | hypothetical protein [Candidatus Marinimicrobia bacterium] | 140 | 1e-36 | MAR96282 |
    | Chromatiales bacterium 21-64-14 [g-proteobacteria]  Next Previous First | | | |
    | proliferating cell nuclear antigen (pcna) [Chromatiales bacterium 21-64-14] | 256 | 4e-81 | OYV74989 |
    | Flavobacteriaceae bacterium [CFB group bacteria]  Next Previous First | | | |
    | proliferating cell nuclear antigen (pcna) [Flavobacteriaceae bacterium] | 254 | 1e-80 | MAD12048 |
    | hypothetical protein [Flavobacteriaceae bacterium] | 101 | 9e-22 | MBH45101 |
    | Rhodobacteraceae bacterium [a-proteobacteria]  Next Previous First | | | |
    | proliferating cell nuclear antigen (pcna) [Rhodobacteraceae bacterium] | 252 | 4e-80 | PQM59040 |
    | proliferating cell nuclear antigen (pcna) [Rhodobacteraceae bacterium] | 235 | 2e-73 | MAR51068 |
    | proliferating cell nuclear antigen (pcna) [Rhodobacteraceae bacterium] | 231 | 2e-72 | MAT00017 |
    | proliferating cell nuclear antigen (pcna) [Rhodobacteraceae bacterium] | 175 | 4e-50 | MAB04571 |
    | proliferating cell nuclear antigen (pcna) [Rhodobacteraceae bacterium] | 157 | 2e-43 | MAR49569 |
    | hypothetical protein [Rhodobacteraceae bacterium] | 118 | 3e-28 | MAR17754 |
    | Flavobacteriales bacterium TMED191 [CFB group bacteria]  Next Previous First | | | |
    | proliferating cell nuclear antigen (pcna) [Flavobacteriales bacterium TMED191] | 251 | 7e-80 | RPG58329 |
    | Crocinitomicaceae bacterium [CFB group bacteria]  Next Previous First | | | |
    | proliferating cell nuclear antigen (pcna) [Crocinitomicaceae bacterium] | 249 | 3e-79 | NBR15457 |
    | proliferating cell nuclear antigen (pcna) [Crocinitomicaceae bacterium] | 231 | 5e-72 | NBR16174 |
    | proliferating cell nuclear antigen (pcna) [Crocinitomicaceae bacterium] | 231 | 5e-72 | NCA22052 |
    | hypothetical protein [Crocinitomicaceae bacterium] | 101 | 3e-23 | NCA21076 |
    | Alphaproteobacteria bacterium TMED62 [a-proteobacteria]  Next Previous First | | | |
    | proliferating cell nuclear antigen (pcna) [Alphaproteobacteria bacterium TMED62] | 246 | 1e-77 | OUU62278 |
    | Halobacteriovoraceae bacterium [proteobacteria]  Next Previous First | | | |
    | proliferating cell nuclear antigen (pcna) [Halobacteriovoraceae bacterium] | 243 | 8e-77 | MAW08577 |
    | Candidatus Endolissoclinum sp. TMED37 [a-proteobacteria]  Next Previous First | | | |
    | proliferating cell nuclear antigen (pcna) [Candidatus Endolissoclinum sp. TMED37] | 238 | 4e-75 | OUU16001 |
    | proliferating cell nuclear antigen (pcna) [Candidatus Endolissoclinum sp. TMED37] | 178 | 2e-51 | OUU15212 |
    | hypothetical protein CBB97\_24650, partial [Candidatus Endolissoclinum sp. TMED37] | 69.5 | 7e-11 | OUU14720 |
    | Proteobacteria bacterium [proteobacteria]  Next Previous First | | | |
    | proliferating cell nuclear antigen (pcna) [Proteobacteria bacterium] | 238 | 9e-75 | NDG72174 |
    | proliferating cell nuclear antigen (pcna) [Proteobacteria bacterium] | 205 | 2e-61 | NBP00213 |
    | proliferating cell nuclear antigen (pcna) [Proteobacteria bacterium] | 201 | 7e-60 | NDG27889 |
    | proliferating cell nuclear antigen (pcna) [Proteobacteria bacterium] | 138 | 5e-36 | HGO27509 |
    | hypothetical protein [Proteobacteria bacterium] | 52.2 | 3e-05 | NBV35239 |
    | Candidatus Puniceispirillum sp. TMED52 [a-proteobacteria]  Next Previous First | | | |
    | proliferating cell nuclear antigen (pcna) [Candidatus Puniceispirillum sp. TMED52] | 237 | 1e-74 | OUU49664 |
    | Rhodothermaceae bacterium TMED105 [CFB group bacteria]  Next Previous First | | | |
    | proliferating cell nuclear antigen (pcna) [Rhodothermaceae bacterium TMED105] | 237 | 1e-74 | RPF82043 |
    | hypothetical protein CBC65\_000905 [Rhodothermaceae bacterium TMED105] | 58.7 | 4e-06 | RPF82134 |
    | Dehalococcoidia bacterium [GNS bacteria]  Next Previous First | | | |
    | proliferating cell nuclear antigen (pcna) [Dehalococcoidia bacterium] | 235 | 4e-74 | MAT63990 |
    | proliferating cell nuclear antigen (pcna) [Dehalococcoidia bacterium] | 198 | 1e-59 | PCH85639 |
    | Porticoccaceae bacterium [g-proteobacteria]  Next Previous First | | | |
    | proliferating cell nuclear antigen (pcna) [Porticoccaceae bacterium] | 235 | 8e-74 | MAE55769 |
    | Micrococcales bacterium [high GC Gram+]  Next Previous First | | | |
    | proliferating cell nuclear antigen (pcna) [Micrococcales bacterium] | 235 | 1e-73 | NBR26233 |
    | Chitinophagia bacterium [CFB group bacteria]  Next Previous First | | | |
    | proliferating cell nuclear antigen (pcna) [Chitinophagia bacterium] | 232 | 1e-72 | NDC41159 |
    | proliferating cell nuclear antigen (pcna) [Chitinophagia bacterium] | 175 | 5e-51 | NDA64219 |
    | Aestuariivita sp. [a-proteobacteria]  Next Previous First | | | |
    | proliferating cell nuclear antigen (pcna) [Aestuariivita sp.] | 231 | 2e-72 | MAT87114 |
    | Rhodospirillaceae bacterium [a-proteobacteria]  Next Previous First | | | |
    | proliferating cell nuclear antigen (pcna) [Rhodospirillaceae bacterium] | 230 | 5e-72 | MBP02103 |
    | proliferating cell nuclear antigen (pcna) [Rhodospirillaceae bacterium] | 175 | 4e-50 | MAI14456 |
    | hypothetical protein [Rhodospirillaceae bacterium] | 159 | 7e-44 | MAI14441 |
    | Alteromonadaceae bacterium TMED7 [g-proteobacteria]  Next Previous First | | | |
    | proliferating cell nuclear antigen (pcna) [Alteromonadaceae bacterium TMED7] | 230 | 7e-72 | RPH19578 |
    | Verrucomicrobiales bacterium [verrucomicrobia]  Next Previous First | | | |
    | proliferating cell nuclear antigen (pcna) [Verrucomicrobiales bacterium] | 228 | 5e-71 | MAB60694 |
    | hypothetical protein [Verrucomicrobiales bacterium] | 75.7 | 5e-12 | MAD25962 |
    | candidate division WOR-3 bacterium [bacteria]  Next Previous First | | | |
    | proliferating cell nuclear antigen (pcna) [candidate division WOR-3 bacterium] | 216 | 2e-66 | HEN27889 |
    | Planctomycetaceae bacterium [planctomycetes]  Next Previous First | | | |
    | proliferating cell nuclear antigen (pcna) [Planctomycetaceae bacterium] | 210 | 4e-64 | MAD20416 |
    | Planctomycetes bacterium [bacteria]  Next Previous First | | | |
    | proliferating cell nuclear antigen (pcna) [Planctomycetes bacterium] | 210 | 6e-64 | MBJ75944 |
    | Candidatus Pelagibacter sp. [a-proteobacteria]  Next Previous First | | | |
    | proliferating cell nuclear antigen (pcna) [Candidatus Pelagibacter sp.] | 208 | 2e-63 | MBP37067 |
    | proliferating cell nuclear antigen (pcna) [Candidatus Pelagibacter sp.] | 158 | 2e-43 | MAV57173 |
    | Dehalococcoidaceae bacterium [GNS bacteria]  Next Previous First | | | |
    | proliferating cell nuclear antigen (pcna) [Dehalococcoidaceae bacterium] | 207 | 4e-63 | MBR74595 |
    | Magnetococcales bacterium [a-proteobacteria]  Next Previous First | | | |
    | proliferating cell nuclear antigen (pcna) [Magnetococcales bacterium] | 209 | 1e-62 | MBA42970 |
    | Candidatus Rokubacteria bacterium 13\_1\_40CM\_2\_70\_45 [bacteria]  Next Previous First | | | |
    | proliferating cell nuclear antigen (pcna) [Candidatus Rokubacteria bacterium 13\_1\_40CM\_2\_70\_45] | 205 | 2e-62 | OLD30892 |
    | proliferating cell nuclear antigen (pcna) [Candidatus Rokubacteria bacterium 13\_1\_40CM\_2\_70\_45] | 202 | 4e-61 | OLD25330 |
    | DNA polymerase sliding clamp [Candidatus Rokubacteria bacterium 13\_1\_40CM\_2\_70\_45] | 160 | 6e-45 | OLD29434 |
    | Gammaproteobacteria bacterium [g-proteobacteria]  Next Previous First | | | |
    | proliferating cell nuclear antigen (pcna) [Gammaproteobacteria bacterium] | 202 | 4e-61 | NIW10639 |
    | proliferating cell nuclear antigen (pcna) [Gammaproteobacteria bacterium] | 190 | 4e-56 | NCA73651 |
    | hypothetical protein [Gammaproteobacteria bacterium] | 165 | 3e-46 | MAN39109 |
    | Opitutae bacterium [verrucomicrobia]  Next Previous First | | | |
    | proliferating cell nuclear antigen (pcna) [Opitutae bacterium] | 196 | 3e-56 | MBN37929 |
    | Clostridiales bacterium [firmicutes]  Next Previous First | | | |
    | proliferating cell nuclear antigen (pcna) [Clostridiales bacterium] | 188 | 2e-55 | NTV78099 |
    | Candidatus Levybacteria bacterium [bacteria]  Next Previous First | | | |
    | proliferating cell nuclear antigen (pcna) [Candidatus Levybacteria bacterium] | 186 | 1e-54 | MBI2034871 |
    | Phycisphaerae bacterium [bacteria]  Next Previous First | | | |
    | hypothetical protein [Phycisphaerae bacterium] | 185 | 4e-54 | MAI67428 |
    | Parcubacteria group bacterium GW2011\_GWB1\_52\_7 [bacteria]  Next Previous First | | | |
    | polymerase sliding clamp protein [Parcubacteria group bacterium GW2011\_GWB1\_52\_7] | 183 | 1e-53 | KKW29271 |
    | Rickettsiales bacterium [a-proteobacteria]  Next Previous First | | | |
    | proliferating cell nuclear antigen (pcna) [Rickettsiales bacterium] | 181 | 4e-52 | MBB18791 |
    | hypothetical protein COA94\_02085 [Rickettsiales bacterium] | 64.9 | 2e-08 | PCJ29226 |
    | hypothetical protein [Rickettsiales bacterium] | 62.2 | 1e-07 | MAY90458 |
    | bacterium TMED178 [bacteria]  Next Previous First | | | |
    | proliferating cell nuclear antigen (pcna) [bacterium TMED178] | 175 | 4e-50 | OUX68015 |
    | hypothetical protein CBD38\_00850 [bacterium TMED178] | 159 | 7e-44 | OUX68000 |
    | Bdellovibrionaceae bacterium [proteobacteria]  Next Previous First | | | |
    | proliferating cell nuclear antigen (pcna) [Bdellovibrionaceae bacterium] | 173 | 2e-49 | MBN20023 |
    | Candidatus Dadabacteria bacterium [bacteria]  Next Previous First | | | |
    | DNA polymerase sliding clamp [Candidatus Dadabacteria bacterium] | 171 | 8e-49 | NIQ14317 |
    | Chloroflexi bacterium [GNS bacteria]  Next Previous First | | | |
    | hypothetical protein [Chloroflexi bacterium] | 168 | 9e-48 | MBI63664 |
    | DNA polymerase sliding clamp [Chloroflexi bacterium] | 168 | 1e-47 | MAE80883 |
    | hypothetical protein DRI37\_10220, partial [Chloroflexi bacterium] | 145 | 5e-39 | RLC83458 |
    | hypothetical protein DRI61\_15465, partial [Chloroflexi bacterium] | 91.5 | 9e-18 | RLC75119 |
    | hypothetical protein DRH97\_00615 [Chloroflexi bacterium] | 74.5 | 5e-12 | RLC69169 |
    | Ignavibacteria bacterium [bacteria]  Next Previous First | | | |
    | proliferating cell nuclear antigen (pcna) [Ignavibacteria bacterium] | 168 | 9e-46 | HFO52032 |
    | Candidatus Woesebacteria bacterium GW2011\_GWB1\_39\_12 [bacteria]  Next Previous First | | | |
    | polymerase sliding clamp protein [Candidatus Woesebacteria bacterium GW2011\_GWB1\_39\_12] | 164 | 1e-45 | KKR01610 |
    | Gemmatimonadetes bacterium [bacteria]  Next Previous First | | | |
    | DNA polymerase sliding clamp [Gemmatimonadetes bacterium] | 160 | 2e-44 | MBQ40860 |
    | Candidatus Rokubacteria bacterium 13\_1\_40CM\_3\_69\_38 [bacteria]  Next Previous First | | | |
    | DNA polymerase sliding clamp [Candidatus Rokubacteria bacterium 13\_1\_40CM\_3\_69\_38] | 158 | 2e-44 | OLC90189 |
    | Propionibacteriaceae bacterium [high GC Gram+]  Next Previous First | | | |
    | proliferating cell nuclear antigen (pcna), partial [Propionibacteriaceae bacterium] | 159 | 3e-44 | RYZ28222 |
    | Rhodobiaceae bacterium [a-proteobacteria]  Next Previous First | | | |
    | hypothetical protein [Rhodobiaceae bacterium] | 158 | 8e-44 | MAL04566 |
    | Nautiliaceae bacterium [e-proteobacteria]  Next Previous First | | | |
    | hypothetical protein [Nautiliaceae bacterium] | 156 | 3e-43 | HIQ51620 |
    | Candidatus Rokubacteria bacterium 13\_1\_40CM\_4\_69\_39 [bacteria]  Next Previous First | | | |
    | DNA polymerase sliding clamp, partial [Candidatus Rokubacteria bacterium 13\_1\_40CM\_4\_69\_39] | 149 | 7e-41 | OLC55625 |
    | Nitrospiraceae bacterium [bacteria]  Next Previous First | | | |
    | DNA polymerase sliding clamp [Nitrospiraceae bacterium] | 143 | 6e-38 | NIA11429 |
    | Legionellales bacterium [g-proteobacteria]  Next Previous First | | | |
    | hypothetical protein [Legionellales bacterium] | 139 | 2e-36 | MAJ82112 |
    | hypothetical protein [Legionellales bacterium] | 68.7 | 1e-09 | MBL97255 |
    | Chloroflexi bacterium RBG\_16\_64\_32 [GNS bacteria]  Next Previous First | | | |
    | hypothetical protein A2W34\_06430 [Chloroflexi bacterium RBG\_16\_64\_32] | 135 | 8e-34 | OGO49108 |
    | Candidatus Latescibacteria bacterium [bacteria]  Next Previous First | | | |
    | DNA polymerase sliding clamp, partial [Candidatus Latescibacteria bacterium] | 112 | 3e-27 | RKY57622 |
    | Acinetobacter baumannii [g-proteobacteria]  Next Previous First | | | |
    | proliferating cell nuclear antigen (pcna), partial [Acinetobacter baumannii] | 110 | 8e-27 | WP\_071289881 |
    | proliferating cell nuclear antigen (pcna), partial [Acinetobacter baumannii] | 110 | 8e-27 | OIF93849 |
    | proliferating cell nuclear antigen (pcna), partial [Acinetobacter baumannii] | 91.5 | 5e-20 | WP\_147509764 |
    | Synergistetes bacterium [bacteria]  Next Previous First | | | |
    | DNA polymerase sliding clamp [Synergistetes bacterium] | 108 | 1e-25 | MBC7332724 |
    | Deltaproteobacteria bacterium [d-proteobacteria]  Next Previous First | | | |
    | hypothetical protein EOO40\_09850, partial [Deltaproteobacteria bacterium] | 104 | 9e-23 | RYF05887 |
    | Verrucomicrobia bacterium [verrucomicrobia]  Next Previous First | | | |
    | hypothetical protein [Verrucomicrobia bacterium] | 100 | 7e-22 | NBS88271 |
    | Desulfuromonadales bacterium C00003093 [d-proteobacteria]  Next Previous First | | | |
    | hypothetical protein BA864\_06935 [Desulfuromonadales bacterium C00003093] | 99 | 2e-21 | OEU74059 |
    | hypothetical protein BA864\_05090 [Desulfuromonadales bacterium C00003093] | 78.0 | 3e-13 | OEU73910 |
    | Thermotoga sp. [thermotogales]  Next Previous First | | | |
    | DNA polymerase sliding clamp [Thermotoga sp.] | 93.8 | 4e-20 | HIP92434 |
    | Fischerella sp. [cyanobacteria]  Next Previous First | | | |
    | hypothetical protein [Fischerella sp.] | 93.4 | 6e-20 | HER14123 |
    | Acidimicrobiales bacterium [actinobacteria]  Next Previous First | | | |
    | hypothetical protein [Acidimicrobiales bacterium] | 93.4 | 6e-18 | MBF6555506 |
    | Bacteroidetes bacterium ADurb.BinA104 [CFB group bacteria]  Next Previous First | | | |
    | DNA polymerase sliding clamp [Bacteroidetes bacterium ADurb.BinA104] | 89.1 | 3e-17 | OPZ48648 |
    | Aquificaceae bacterium [aquificales]  Next Previous First | | | |
    | hypothetical protein [Aquificaceae bacterium] | 84.9 | 7e-17 | HID65709 |
    | Moritella sp. JT01 [g-proteobacteria]  Next Previous First | | | |
    | hypothetical protein [Moritella sp. JT01] | 77.6 | 6e-13 | WP\_067050733 |
    | hypothetical protein AKG98\_2780 [Moritella sp. JT01] | 77.6 | 6e-13 | KXO07005 |
    | Maribacter sp. [CFB group bacteria]  Next Previous First | | | |
    | hypothetical protein [Maribacter sp.] | 74.9 | 5e-12 | HAI41630 |
    | Nitrosococcus oceani [g-proteobacteria]  Next Previous First | | | |
    | DNA polymerase, partial [Nitrosococcus oceani] | 67.2 | 1e-10 | WP\_036504199 |
    | DNA polymerase, partial [Nitrosococcus oceani] | 67.2 | 1e-10 | KFI21162 |
    | Candidatus Moranbacteria bacterium [bacteria]  Next Previous First | | | |
    | hypothetical protein [Candidatus Moranbacteria bacterium] | 70.3 | 2e-10 | NCU42455 |
    | Cyanobium sp. ARS6 [cyanobacteria]  Next Previous First | | | |
    | hypothetical protein [Cyanobium sp. ARS6] | 66.0 | 6e-09 | MAF41652 |
    | Microvirga massiliensis [a-proteobacteria]  Next Previous First | | | |
    | hypothetical protein, partial [Microvirga massiliensis] | 62.6 | 7e-09 | WP\_156312854 |
    | Alteromonas sp. [g-proteobacteria]  Next Previous First | | | |
    | hypothetical protein [Alteromonas sp.] | 59.9 | 3e-07 | MAI63263 |
    | Candidatus Poribacteria bacterium [bacteria]  Next Previous First | | | |
    | hypothetical protein [Candidatus Poribacteria bacterium] | 61.0 | 4e-07 | MBP98812 |
    | hypothetical protein [Candidatus Poribacteria bacterium] | 55.6 | 3e-05 | MBP98540 |
    | Fervidobacterium pennivorans [thermotogales]  Next Previous First | | | |
    | DNA polymerase sliding clamp [Fervidobacterium pennivorans] | 56.8 | 4e-07 | HGU52075 |
    | Bacteroidetes bacterium ADurb.Bin302 [CFB group bacteria]  Next Previous First | | | |
    | DNA polymerase sliding clamp [Bacteroidetes bacterium ADurb.Bin302] | 60.3 | 5e-07 | OQA51571 |
    | Spirochaetia bacterium [bacteria]  Next Previous First | | | |
    | hypothetical protein [Spirochaetia bacterium] | 54.5 | 9e-06 | NBK24950 |
    | Candidatus Nomurabacteria bacterium [bacteria]  Next Previous First | | | |
    | hypothetical protein [Candidatus Nomurabacteria bacterium] | 53.7 | 6e-05 | NCU27524 |
    | Armatimonas rosea [bacteria]  Next Previous First | | | |
    | DNA polymerase III subunit beta [Armatimonas rosea] | 55.3 | 7e-05 | WP\_184202552 |
    | DNA polymerase-3 subunit beta [Armatimonas rosea] | 55.3 | 7e-05 | MBB6052857 |
    | Thermodesulfobacteria bacterium [bacteria]  Next Previous First | | | |
    | hypothetical protein DRP41\_07375, partial [Thermodesulfobacteria bacterium] | 52.2 | 7e-05 | RKX62390 |
    | Desulfobacterales bacterium [d-proteobacteria]  Next Previous First | | | |
    | hypothetical protein [Desulfobacterales bacterium] | 51.4 | 1e-04 | HID70036 |
    | Alkalinema sp. FACHB-956 [cyanobacteria]  Next Previous First | | | |
    | DNA polymerase III subunit beta [Alkalinema sp. FACHB-956] | 54.5 | 1e-04 | WP\_199307260 |
    | DNA polymerase III subunit beta [Alkalinema sp. FACHB-956] | 54.1 | 2e-04 | MBD2328424 |
    | Oscillatoriales cyanobacterium USR001 [cyanobacteria]  Next Previous First | | | |
    | DNA polymerase III subunit beta [Oscillatoriales cyanobacterium USR001] | 53.7 | 2e-04 | OCR01436 |
    | Lachnospiraceae bacterium [firmicutes]  Next Previous First | | | |
    | hypothetical protein [Lachnospiraceae bacterium] | 53.7 | 2e-04 | HBI72324 |
    | Spirulina sp. DLM2.Bin59 [cyanobacteria]  Next Previous First | | | |
    | DNA polymerase III subunit beta [Spirulina sp. DLM2.Bin59] | 52.6 | 4e-04 | TVQ53692 |
    | Phormidium ambiguum [cyanobacteria]  Next Previous First | | | |
    | DNA polymerase III subunit beta [Phormidium ambiguum] | 52.6 | 6e-04 | WP\_084555250 |
    | DNA polymerase III subunit beta [Phormidium ambiguum] | 51.0 | 0.002 | WP\_084555156 |
    | Spirulina subsalsa [cyanobacteria]  Next Previous First | | | |
    | DNA polymerase III subunit beta [Spirulina subsalsa] | 52.2 | 6e-04 | WP\_017303270 |
    | Phormidium ambiguum IAM M-71 [cyanobacteria]  Next Previous First | | | |
    | DNA polymerase III subunit beta [Phormidium ambiguum IAM M-71] | 52.2 | 7e-04 | OKH33175 |
    | DNA polymerase III subunit beta [Phormidium ambiguum IAM M-71] | 51.0 | 0.002 | OKH36250 |
    | Okeania sp. SIO3I5 [cyanobacteria]  Next Previous First | | | |
    | DNA polymerase III subunit beta [Okeania sp. SIO3I5] | 51.4 | 0.001 | NEQ38316 |
    | Bacillus cihuensis [firmicutes]  Next Previous First | | | |
    | DNA polymerase III subunit beta [Bacillus cihuensis] | 51.4 | 0.001 | WP\_028393951 |
    | Acidobacteriales bacterium [bacteria]  Next Previous First | | | |
    | DNA polymerase III subunit beta [Acidobacteriales bacterium] | 51.0 | 0.001 | MBI3645860 |
    | Candidatus Koribacter versatilis [bacteria]  Next Previous First | | | |
    | DNA polymerase III subunit beta [Candidatus Koribacter versatilis] | 51.0 | 0.002 | MBI1740949 |
    | Myxacorys almedinensis [cyanobacteria]  Next Previous First | | | |
    | DNA polymerase III subunit beta [Myxacorys almedinensis] | 50.2 | 0.002 | WP\_162425412 |
    | Myxacorys almedinensis A [cyanobacteria]  Next Previous First | | | |
    | DNA polymerase III subunit beta [Myxacorys almedinensis A] | 50.2 | 0.002 | NDJ19888 |
    | Chroococcidiopsis cubana [cyanobacteria]  Next Previous First | | | |
    | DNA polymerase III subunit beta [Chroococcidiopsis cubana] | 51.0 | 0.002 | WP\_106166543 |
    | Chroococcidiopsis cubana CCALA 043 [cyanobacteria]  Next Previous First | | | |
    | DNA polymerase III subunit beta [Chroococcidiopsis cubana CCALA 043] | 51.0 | 0.002 | PSB65429 |
    | Chroococcidiopsis cubana SAG 39.79 [cyanobacteria]  Next Previous First | | | |
    | DNA polymerase III subunit beta [Chroococcidiopsis cubana SAG 39.79] | 51.0 | 0.002 | RUT14619 |
    | Acidobacteria bacterium [bacteria]  Next Previous First | | | |
    | DNA polymerase III subunit beta [Acidobacteria bacterium] | 50.6 | 0.002 | MBI3476530 |
    | DNA polymerase III subunit beta [Acidobacteria bacterium] | 50.6 | 0.002 | PYX28173 |
    | cyanobacterium TDX16 [cyanobacteria]  Next Previous First | | | |
    | DNA polymerase III subunit beta [cyanobacterium TDX16] | 50.2 | 0.003 | OWY67883 |
    | Kamptonema sp. SIO4C4 [cyanobacteria]  Next Previous First | | | |
    | DNA polymerase III subunit beta [Kamptonema sp. SIO4C4] | 50.2 | 0.003 | NEO28492 |
    | Thermobaculum terrenum [bacteria]  Next Previous First | | | |
    | DNA polymerase III subunit beta [Thermobaculum terrenum] | 50.2 | 0.003 | WP\_012874944 |
    | Thermobaculum terrenum ATCC BAA-798 [bacteria]  Next Previous First | | | |
    | DNA polymerase III, beta subunit [Thermobaculum terrenum ATCC BAA-798] | 50.2 | 0.003 | ACZ41909 |
    | Aphanothece hegewaldii [cyanobacteria]  Next Previous First | | | |
    | DNA polymerase III subunit beta [Aphanothece hegewaldii] | 49.9 | 0.003 | WP\_106458545 |
    | Aphanothece hegewaldii CCALA 016 [cyanobacteria]  Next Previous First | | | |
    | DNA polymerase III subunit beta [Aphanothece hegewaldii CCALA 016] | 49.9 | 0.003 | PSF34263 |
    | Hydrocoleum sp. CS-953 [cyanobacteria]  Next Previous First | | | |
    | DNA polymerase III subunit beta [Hydrocoleum sp. CS-953] | 49.1 | 0.007 | WP\_094674721 |
    | Chitinophagaceae bacterium [CFB group bacteria]  Next Previous First | | | |
    | DNA polymerase III subunit beta [Chitinophagaceae bacterium] | 48.7 | 0.009 | MBA2248775 |
    | DNA polymerase III subunit beta [Chitinophagaceae bacterium] | 48.7 | 0.010 | MBC7827772 |
    | Candidatus Fermentibacteria bacterium [bacteria]  Next Previous First | | | |
    | hypothetical protein DRQ25\_00255 [Candidatus Fermentibacteria bacterium] | 47.9 | 0.010 | RKZ11181 |
    | candidate division KSB1 bacterium [bacteria]  Next Previous First | | | |
    | hypothetical protein DRQ11\_08570 [candidate division KSB1 bacterium] | 47.9 | 0.011 | RKY86303 |
    | Chroococcidiopsis sp. FACHB-1243 [cyanobacteria]  Next Previous First | | | |
    | DNA polymerase III subunit beta [Chroococcidiopsis sp. FACHB-1243] | 48.3 | 0.012 | WP\_192160184 |
    | DNA polymerase III subunit beta [Chroococcidiopsis sp. FACHB-1243] | 48.3 | 0.012 | MBD2307785 |
    | Spirochaetes bacterium [bacteria]  Next Previous First | | | |
    | hypothetical protein DRP84\_07425 [Spirochaetes bacterium] | 47.2 | 0.018 | RKX94156 |
    | Oscillatoria sp. PCC 10802 [cyanobacteria]  Next Previous First | | | |
    | DNA polymerase III subunit beta [Oscillatoria sp. PCC 10802] | 47.5 | 0.019 | WP\_017717195 |
    | Okeania sp. SIO1I7 [cyanobacteria]  Next Previous First | | | |
    | DNA polymerase III subunit beta [Okeania sp. SIO1I7] | 47.2 | 0.028 | NET24246 |
    | Bacillus sp. S3 [firmicutes]  Next Previous First | | | |
    | DNA polymerase III subunit beta [Bacillus sp. S3] | 46.8 | 0.035 | WP\_149868010 |
    | DNA polymerase III subunit beta [Bacillus sp. S3] | 46.8 | 0.035 | QCJ40446 |
    | Dialister sp. CAG:486 [firmicutes]  Next Previous First | | | |
    | dNA polymerase III subunit beta [Dialister sp. CAG:486] | 46.8 | 0.039 | CDA47400 |
    | Pseudoflavitalea sp. G-6-1-2 [CFB group bacteria]  Next Previous First | | | |
    | DNA polymerase III subunit beta [Pseudoflavitalea sp. G-6-1-2] | 46.4 | 0.044 | WP\_169423815 |
    | DNA polymerase III subunit beta [Pseudoflavitalea sp. G-6-1-2] | 46.4 | 0.044 | NML19990 |
    | Neobacillus cucumis [firmicutes]  Next Previous First | | | |
    | DNA polymerase III subunit beta [Neobacillus cucumis] | 46.4 | 0.046 | WP\_101650072 |
    | DNA polymerase III subunit beta [Neobacillus cucumis] | 46.4 | 0.046 | PLS02186 |
  - Taxonomy

    Taxonomy Report

    | Taxonomy | Number of hits | Number of Organisms | Description |
    | --- | --- | --- | --- |
    | Bacteria | 257 | 120 |  |
    | .  unclassified Bacteria | 44 | 2 |  |
    | ..  bacterium | 42 | 1 | bacterium hits |
    | ..  bacterium TMED178 | 2 | 1 | bacterium TMED178 hits |
    | .  FCB group | 50 | 18 |  |
    | ..  Bacteroidetes/Chlorobi group | 39 | 14 |  |
    | ...  Bacteroidetes | 38 | 13 |  |
    | ....  Flavobacteriia | 19 | 6 |  |
    | .....  Flavobacteriia bacterium | 5 | 1 | Flavobacteriia bacterium hits |
    | .....  Flavobacteriales | 14 | 5 |  |
    | ......  unclassified Flavobacteriales | 7 | 2 |  |
    | .......  Flavobacteriales bacterium | 6 | 1 | Flavobacteriales bacterium hits |
    | .......  Flavobacteriales bacterium TMED191 | 1 | 1 | Flavobacteriales bacterium TMED191 hits |
    | ......  Flavobacteriaceae | 3 | 2 |  |
    | .......  Flavobacteriaceae bacterium | 2 | 1 | Flavobacteriaceae bacterium hits |
    | .......  Maribacter sp. | 1 | 1 | Maribacter sp. hits |
    | ......  Crocinitomicaceae bacterium | 4 | 1 | Crocinitomicaceae bacterium hits |
    | ....  unclassified Bacteroidetes | 11 | 3 |  |
    | .....  Bacteroidetes bacterium | 9 | 1 | Bacteroidetes bacterium hits |
    | .....  Bacteroidetes bacterium ADurb.BinA104 | 1 | 1 | Bacteroidetes bacterium ADurb.BinA104 hits |
    | .....  Bacteroidetes bacterium ADurb.Bin302 | 1 | 1 | Bacteroidetes bacterium ADurb.Bin302 hits |
    | ....  Rhodothermaceae bacterium TMED105 | 2 | 1 | Rhodothermaceae bacterium TMED105 hits |
    | ....  Chitinophagia | 6 | 3 |  |
    | .....  Chitinophagia bacterium | 2 | 1 | Chitinophagia bacterium hits |
    | .....  Chitinophagaceae | 4 | 2 |  |
    | ......  Chitinophagaceae bacterium | 2 | 1 | Chitinophagaceae bacterium hits |
    | ......  Pseudoflavitalea sp. G-6-1-2 | 2 | 1 | Pseudoflavitalea sp. G-6-1-2 hits |
    | ...  Ignavibacteria bacterium | 1 | 1 | Ignavibacteria bacterium hits |
    | ..  Candidatus Marinimicrobia bacterium | 8 | 1 | Candidatus Marinimicrobia bacterium hits |
    | ..  Gemmatimonadetes bacterium | 1 | 1 | Gemmatimonadetes bacterium hits |
    | ..  Candidatus Latescibacteria bacterium | 1 | 1 | Candidatus Latescibacteria bacterium hits |
    | ..  Candidatus Fermentibacteria bacterium | 1 | 1 | Candidatus Fermentibacteria bacterium hits |
    | .  Proteobacteria | 62 | 29 |  |
    | ..  Alphaproteobacteria | 34 | 13 |  |
    | ...  unclassified Alphaproteobacteria | 10 | 2 |  |
    | ....  Alphaproteobacteria bacterium | 9 | 1 | Alphaproteobacteria bacterium hits |
    | ....  Alphaproteobacteria bacterium TMED62 | 1 | 1 | Alphaproteobacteria bacterium TMED62 hits |
    | ...  Pelagibacterales | 4 | 2 |  |
    | ....  Pelagibacterales bacterium | 2 | 1 | Pelagibacterales bacterium hits |
    | ....  Candidatus Pelagibacter sp. | 2 | 1 | Candidatus Pelagibacter sp. hits |
    | ...  Rhodobacteraceae | 7 | 2 |  |
    | ....  Rhodobacteraceae bacterium | 6 | 1 | Rhodobacteraceae bacterium hits |
    | ....  Aestuariivita sp. | 1 | 1 | Aestuariivita sp. hits |
    | ...  Rhodospirillaceae | 6 | 2 |  |
    | ....  Candidatus Endolissoclinum sp. TMED37 | 3 | 1 | Candidatus Endolissoclinum sp. TMED37 hits |
    | ....  Rhodospirillaceae bacterium | 3 | 1 | Rhodospirillaceae bacterium hits |
    | ...  Candidatus Puniceispirillum sp. TMED52 | 1 | 1 | Candidatus Puniceispirillum sp. TMED52 hits |
    | ...  Magnetococcales bacterium | 1 | 1 | Magnetococcales bacterium hits |
    | ...  Rickettsiales bacterium | 3 | 1 | Rickettsiales bacterium hits |
    | ...  Rhizobiales | 2 | 2 |  |
    | ....  Rhodobiaceae bacterium | 1 | 1 | Rhodobiaceae bacterium hits |
    | ....  Microvirga massiliensis | 1 | 1 | Microvirga massiliensis hits |
    | ..  Gammaproteobacteria | 16 | 9 |  |
    | ...  Chromatiales | 3 | 2 |  |
    | ....  Chromatiales bacterium 21-64-14 | 1 | 1 | Chromatiales bacterium 21-64-14 hits |
    | ....  Nitrosococcus oceani | 2 | 1 | Nitrosococcus oceani hits |
    | ...  Porticoccaceae bacterium | 1 | 1 | Porticoccaceae bacterium hits |
    | ...  Alteromonadales | 4 | 3 |  |
    | ....  Alteromonadaceae | 2 | 2 |  |
    | .....  Alteromonadaceae bacterium TMED7 | 1 | 1 | Alteromonadaceae bacterium TMED7 hits |
    | .....  Alteromonas sp. | 1 | 1 | Alteromonas sp. hits |
    | ....  Moritella sp. JT01 | 2 | 1 | Moritella sp. JT01 hits |
    | ...  Gammaproteobacteria bacterium | 3 | 1 | Gammaproteobacteria bacterium hits |
    | ...  Legionellales bacterium | 2 | 1 | Legionellales bacterium hits |
    | ...  Acinetobacter baumannii | 3 | 1 | Acinetobacter baumannii hits |
    | ..  Oligoflexia | 2 | 2 |  |
    | ...  Halobacteriovoraceae bacterium | 1 | 1 | Halobacteriovoraceae bacterium hits |
    | ...  Bdellovibrionaceae bacterium | 1 | 1 | Bdellovibrionaceae bacterium hits |
    | ..  Proteobacteria bacterium | 5 | 1 | Proteobacteria bacterium hits |
    | ..  delta/epsilon subdivisions | 5 | 4 |  |
    | ...  Nautiliaceae bacterium | 1 | 1 | Nautiliaceae bacterium hits |
    | ...  Deltaproteobacteria | 4 | 3 |  |
    | ....  Deltaproteobacteria bacterium | 1 | 1 | Deltaproteobacteria bacterium hits |
    | ....  Desulfuromonadales bacterium C00003093 | 2 | 1 | Desulfuromonadales bacterium C00003093 hits |
    | ....  Desulfobacterales bacterium | 1 | 1 | Desulfobacterales bacterium hits |
    | .  Terrabacteria group | 66 | 41 |  |
    | ..  Actinobacteria | 19 | 6 |  |
    | ...  Actinobacteria | 16 | 4 |  |
    | ....  Micrococcales | 2 | 2 |  |
    | .....  Microbacterium arborescens | 1 | 1 | Microbacterium arborescens hits |
    | .....  Micrococcales bacterium | 1 | 1 | Micrococcales bacterium hits |
    | ....  Actinobacteria bacterium | 13 | 1 | Actinobacteria bacterium hits |
    | ....  Propionibacteriaceae bacterium | 1 | 1 | Propionibacteriaceae bacterium hits |
    | ...  Acidimicrobiales | 3 | 2 |  |
    | ....  Acidimicrobiaceae bacterium | 2 | 1 | Acidimicrobiaceae bacterium hits |
    | ....  Acidimicrobiales bacterium | 1 | 1 | Acidimicrobiales bacterium hits |
    | ..  Chloroflexi | 9 | 4 |  |
    | ...  Dehalococcoidia | 3 | 2 |  |
    | ....  Dehalococcoidia bacterium | 2 | 1 | Dehalococcoidia bacterium hits |
    | ....  Dehalococcoidaceae bacterium | 1 | 1 | Dehalococcoidaceae bacterium hits |
    | ...  unclassified Chloroflexi | 6 | 2 |  |
    | ....  Chloroflexi bacterium | 5 | 1 | Chloroflexi bacterium hits |
    | ....  Chloroflexi bacterium RBG\_16\_64\_32 | 1 | 1 | Chloroflexi bacterium RBG\_16\_64\_32 hits |
    | ..  Firmicutes | 8 | 6 |  |
    | ...  Clostridiales | 2 | 2 |  |
    | ....  Clostridiales bacterium | 1 | 1 | Clostridiales bacterium hits |
    | ....  Lachnospiraceae bacterium | 1 | 1 | Lachnospiraceae bacterium hits |
    | ...  Bacillaceae | 5 | 3 |  |
    | ....  Bacillus | 3 | 2 |  |
    | .....  Bacillus cihuensis | 1 | 1 | Bacillus cihuensis hits |
    | .....  Bacillus sp. S3 | 2 | 1 | Bacillus sp. S3 hits |
    | ....  Neobacillus cucumis | 2 | 1 | Neobacillus cucumis hits |
    | ...  Dialister sp. CAG:486 | 1 | 1 | Dialister sp. CAG:486 hits |
    | ..  Cyanobacteria | 26 | 22 |  |
    | ...  Fischerella sp. | 1 | 1 | Fischerella sp. hits |
    | ...  Synechococcales | 5 | 4 |  |
    | ....  Cyanobium sp. ARS6 | 1 | 1 | Cyanobium sp. ARS6 hits |
    | ....  Leptolyngbyaceae | 4 | 3 |  |
    | .....  Alkalinema sp. FACHB-956 | 2 | 1 | Alkalinema sp. FACHB-956 hits |
    | .....  Myxacorys | 2 | 2 |  |
    | ......  Myxacorys almedinensis | 1 | 2 | Myxacorys almedinensis hits |
    | .......  Myxacorys almedinensis A | 1 | 1 | Myxacorys almedinensis A hits |
    | ...  Oscillatoriophycideae | 12 | 10 |  |
    | ....  Oscillatoriales | 10 | 8 |  |
    | .....  Oscillatoriales cyanobacterium USR001 | 1 | 1 | Oscillatoriales cyanobacterium USR001 hits |
    | .....  Oscillatoriaceae | 7 | 5 |  |
    | ......  Phormidium | 4 | 2 |  |
    | .......  Phormidium ambiguum | 2 | 2 | Phormidium ambiguum hits |
    | ........  Phormidium ambiguum IAM M-71 | 2 | 1 | Phormidium ambiguum IAM M-71 hits |
    | ......  unclassified Okeania | 2 | 2 |  |
    | .......  Okeania sp. SIO3I5 | 1 | 1 | Okeania sp. SIO3I5 hits |
    | .......  Okeania sp. SIO1I7 | 1 | 1 | Okeania sp. SIO1I7 hits |
    | ......  Oscillatoria sp. PCC 10802 | 1 | 1 | Oscillatoria sp. PCC 10802 hits |
    | .....  Microcoleaceae | 2 | 2 |  |
    | ......  Kamptonema sp. SIO4C4 | 1 | 1 | Kamptonema sp. SIO4C4 hits |
    | ......  Hydrocoleum sp. CS-953 | 1 | 1 | Hydrocoleum sp. CS-953 hits |
    | ....  Aphanothece | 2 | 2 |  |
    | .....  Aphanothece hegewaldii | 1 | 2 | Aphanothece hegewaldii hits |
    | ......  Aphanothece hegewaldii CCALA 016 | 1 | 1 | Aphanothece hegewaldii CCALA 016 hits |
    | ...  Spirulina | 2 | 2 |  |
    | ....  Spirulina sp. DLM2.Bin59 | 1 | 1 | Spirulina sp. DLM2.Bin59 hits |
    | ....  Spirulina subsalsa | 1 | 1 | Spirulina subsalsa hits |
    | ...  Chroococcidiopsis | 5 | 4 |  |
    | ....  Chroococcidiopsis cubana | 1 | 3 | Chroococcidiopsis cubana hits |
    | .....  Chroococcidiopsis cubana CCALA 043 | 1 | 1 | Chroococcidiopsis cubana CCALA 043 hits |
    | .....  Chroococcidiopsis cubana SAG 39.79 | 1 | 1 | Chroococcidiopsis cubana SAG 39.79 hits |
    | ....  Chroococcidiopsis sp. FACHB-1243 | 2 | 1 | Chroococcidiopsis sp. FACHB-1243 hits |
    | ...  cyanobacterium TDX16 | 1 | 1 | cyanobacterium TDX16 hits |
    | ..  Armatimonas rosea | 2 | 1 | Armatimonas rosea hits |
    | ..  Thermobaculum | 2 | 2 |  |
    | ...  Thermobaculum terrenum | 1 | 2 | Thermobaculum terrenum hits |
    | ....  Thermobaculum terrenum ATCC BAA-798 | 1 | 1 | Thermobaculum terrenum ATCC BAA-798 hits |
    | .  Nitrospinae bacterium | 1 | 1 | Nitrospinae bacterium hits |
    | .  PVC group | 7 | 6 |  |
    | ..  Verrucomicrobia | 4 | 3 |  |
    | ...  Verrucomicrobiales bacterium | 2 | 1 | Verrucomicrobiales bacterium hits |
    | ...  Opitutae bacterium | 1 | 1 | Opitutae bacterium hits |
    | ...  Verrucomicrobia bacterium | 1 | 1 | Verrucomicrobia bacterium hits |
    | ..  Planctomycetes | 3 | 3 |  |
    | ...  Planctomycetaceae bacterium | 1 | 1 | Planctomycetaceae bacterium hits |
    | ...  Planctomycetes bacterium | 1 | 1 | Planctomycetes bacterium hits |
    | ...  Phycisphaerae bacterium | 1 | 1 | Phycisphaerae bacterium hits |
    | .  Bacteria candidate phyla | 15 | 12 |  |
    | ..  candidate division WOR-3 bacterium | 1 | 1 | candidate division WOR-3 bacterium hits |
    | ..  unclassified Candidatus Rokubacteria | 5 | 3 |  |
    | ...  Candidatus Rokubacteria bacterium 13\_1\_40CM\_2\_70\_45 | 3 | 1 | Candidatus Rokubacteria bacterium 13\_1\_40CM\_2\_70\_45 hits |
    | ...  Candidatus Rokubacteria bacterium 13\_1\_40CM\_3\_69\_38 | 1 | 1 | Candidatus Rokubacteria bacterium 13\_1\_40CM\_3\_69\_38 hits |
    | ...  Candidatus Rokubacteria bacterium 13\_1\_40CM\_4\_69\_39 | 1 | 1 | Candidatus Rokubacteria bacterium 13\_1\_40CM\_4\_69\_39 hits |
    | ..  Patescibacteria group | 5 | 5 |  |
    | ...  Microgenomates group | 2 | 2 |  |
    | ....  Candidatus Levybacteria bacterium | 1 | 1 | Candidatus Levybacteria bacterium hits |
    | ....  Candidatus Woesebacteria bacterium GW2011\_GWB1\_39\_12 | 1 | 1 | Candidatus Woesebacteria bacterium GW2011\_GWB1\_39\_12 hits |
    | ...  Parcubacteria group | 3 | 3 |  |
    | ....  Parcubacteria group bacterium GW2011\_GWB1\_52\_7 | 1 | 1 | Parcubacteria group bacterium GW2011\_GWB1\_52\_7 hits |
    | ....  Candidatus Moranbacteria bacterium | 1 | 1 | Candidatus Moranbacteria bacterium hits |
    | ....  Candidatus Nomurabacteria bacterium | 1 | 1 | Candidatus Nomurabacteria bacterium hits |
    | ..  Candidatus Dadabacteria bacterium | 1 | 1 | Candidatus Dadabacteria bacterium hits |
    | ..  Candidatus Poribacteria bacterium | 2 | 1 | Candidatus Poribacteria bacterium hits |
    | ..  candidate division KSB1 bacterium | 1 | 1 | candidate division KSB1 bacterium hits |
    | .  Nitrospiraceae bacterium | 1 | 1 | Nitrospiraceae bacterium hits |
    | .  Synergistetes bacterium | 1 | 1 | Synergistetes bacterium hits |
    | .  Thermotogales | 2 | 2 |  |
    | ..  Thermotoga sp. | 1 | 1 | Thermotoga sp. hits |
    | ..  Fervidobacterium pennivorans | 1 | 1 | Fervidobacterium pennivorans hits |
    | .  Aquificaceae bacterium | 1 | 1 | Aquificaceae bacterium hits |
    | .  Spirochaetes | 2 | 2 |  |
    | ..  Spirochaetia bacterium | 1 | 1 | Spirochaetia bacterium hits |
    | ..  Spirochaetes bacterium | 1 | 1 | Spirochaetes bacterium hits |
    | .  Thermodesulfobacteria bacterium | 1 | 1 | Thermodesulfobacteria bacterium hits |
    | .  Acidobacteria | 4 | 3 |  |
    | ..  Acidobacteriales | 2 | 2 |  |
    | ...  Acidobacteriales bacterium | 1 | 1 | Acidobacteriales bacterium hits |
    | ...  Candidatus Koribacter versatilis | 1 | 1 | Candidatus Koribacter versatilis hits |
    | ..  Acidobacteria bacterium | 2 | 1 | Acidobacteria bacterium hits |


Feedback
Top


### Connect

- Twitter
- Facebook
- YouTube
- LinkedIn
- GitHub

- Blog
- Support Center

### National Center for Biotechnology Information

 8600 Rockville Pike
Bethesda  MD, 20894 USA 

- About us
- Contact us
- Polices
- FOIA

#### Popular

- PubMed
- PubMed Central
- Bookshelf
- PubChem
- Gene
- BLAST
- Nucleotide
- Protein
- GEO

#### Resources

- Literature
- Health
- Genomes
- Genes
- Proteins
- Chemicals

#### Actions

- Submit
- Download
- Learn
- Develop
- Analyze
- Research

NLM
 | 
NIH
 | 
HHS
 | 
USA.gov


PreferencesTurn off

External link. Please review our privacy policy.
