# Supplementary material for: Evolution and origin of sliding clamp in bacteria, archaea and eukarya
Source: PLoS One. 2021 Aug 11;16(8):e0241093. doi: 10.1371/journal.pone.0241093 (PMC8357120; doi:10.1371/journal.pone.0241093)
Supplement: S1 Data — (ZIP) [file pone.0241093.s008.zip › New folder/NCBI Blast_sp_O73947_PCNA_PYRFU DNA polymerase sliding....html2.html]

NCBI Blast:sp|O73947|PCNA\_PYRFU DNA polymerase sliding...


 


- NCBI Home
- Sign in to NCBI
- Skip to Main Content
- Skip to Navigation
- About NCBI Accesskeys

National Institutes of Health

U.S. National Library of Medicine

National Center for Biotechnology Information

NCBI homepage

Log in


Show account info

Close

#### Account

Logged in as:  
**username**

- Dashboard (My NCBI)
- Publications (My Bibliography)
- Account settings
- Log out


BLAST ® » blastp suite »

# results for RID-YS3PPWSC013

- Home
- Recent Results
- Saved Strategies
- Help


- Edit Search
- Save Search
- Search Summary

- How to read this report?
- BLAST Help Videos
- Back to Traditional Results Page

Your search is limited to records that include: Bacteria (taxid:2)

- Full Entrez Query

  txid2 [ORGN]


Search Parameters

| Search parameter name | Search parameter value |
| --- | --- |
| Program | blastp |
| Word size | 3 |
| Expect value | 0.05 |
| Hitlist size | 500 |
| Gapcosts | 11,1 |
| Matrix | BLOSUM62 |
| Filter string | F |
| Genetic Code | 1 |
| Window Size | 40 |
| Threshold | 11 |
| Composition-based stats | 1 |

Database

| Database parameter name | Database parameter value |
| --- | --- |
| Posted date | Dec 28, 2020 12:42 AM |
| Number of letters | 87,974,785,795 |
| Number of sequences | 264,187,339 |
| Entrez query | Includes: Bacteria (taxid:2)  Excludes:  None |

Karlin-Altschul statistics

| Params | Ungapped | Gapped |
| --- | --- | --- |
| Lambda | 0.31574 | 0.267 |
| K | 0.135604 | 0.0423571 |
| H | 0.360763 | 0.14 |
| Alpha | 0.7916 | 1.9 |
| Alpha\_v | 4.96466 | 42.6028 |
| Sigma |  | 43.6362 |

Results Statistics

| Results Statistics parameter name | Results Statistics parameter value |
| --- | --- |

Job Title
:   sp|O73947|PCNA\_PYRFU DNA polymerase sliding...
    ...

    sp|O73947|PCNA\_PYRFU DNA polymerase sliding...

RID
:   YS3PPWSC013
    Search expires on 12-31 12:23 pm

    - Download All
      - Text
      - XML
      - ASN.1
      - JSON Seq-align
      - Hit Table(text)
      - Hit Table(csv)
      - Multiple-file XML2
      - Single-file XML2
      - Multiple-file JSON
      - Single-file JSON
      - SAM
      - PSSM

Results for
:   lcl|Query\_91217 sp|O73947|PCNA\_PYRFU DNA polymerase sliding clamp OS=Pyrococcus furiosus (strain ATCC 43587 / DSM 3...(249aa)

Program
:   PSI-BLAST Iteration 3

    - Citation

      Reference 

      Stephen F. Altschul, Thomas L. Madden, Alejandro A. Schäffer, Jinghui Zhang, Zheng Zhang, Webb Miller, and David J. Lipman (1997), "Gapped BLAST and PSI-BLAST: a new generation of protein database search programs", Nucleic Acids Res. 25:3389-3402.

      Reference - composition-based statistics starting in round 2

      Alejandro A. Schäffer, L. Aravind, Thomas L. Madden, Sergei Shavirin, John L. Spouge, Yuri I. Wolf, Eugene V. Koonin, and Stephen F. Altschul (2001), "Improving the accuracy of PSI-BLAST protein database searches with composition-based statistics and other refinements", Nucleic Acids Res. 29:2994-3005.

Database
:   nr

    - See details

Query ID
:   lcl|Query\_91217
    lcl|Query\_91217

Description
:   sp|O73947|PCNA\_PYRFU DNA polymerase sliding clamp OS=Pyrococcus furiosus (strain ATCC 43587 / DSM 3638 / JCM 8422 / Vc1) OX=186497 GN=pcn PE=1 SV=2
    ...

    sp|O73947|PCNA\_PYRFU DNA polymerase sliding clamp OS=Pyrococcus furiosus (strain ATCC 43587 / DSM 3638 / JCM 8422 / Vc1) OX=186497 GN=pcn PE=1 SV=2

Molecule type
:   amino acid

Query Length
:   249

Other reports
:   Distance tree of results
    Multiple alignment
    MSA viewer
     Help

    Reports are generated on using all sequences producing significant alignments. To generate reports on a subset of sequences, use the report links in the Descriptions tab while selecting specific sequences.

## Filter Results

Organism only top 20 will appear


exclude

Add organism


---

Percent Identity from

Percent Identity to

E value from

E value to

Query Coverage from

Query Coverage to

PSI-BLAST incl. threshold

Filter Reset

## Run PSI-Blast iteration 4

Number of sequences


Run

- Descriptions

  ### Sequences producing significant alignments

  - Download
    - FASTA (complete sequence)
    - FASTA (aligned sequences)
    - GenBank (complete sequence)
    - Hit Table (text)
    - Hit Table (CSV)
    - Text
    - Descriptions Table (CSV)
    - XML
    - ASN.1
  - New

    Manage Columns
    - Description
    - Scientific Name
    - Common Name
    - Taxid
    - Max Score
    - Total Score
    - Query Coverage
    - E value
    - Percent Identity
    - Acc. Len
    - Accession
    - Restore defaults
  - Show

    10
    50
    100
    250
    500
    1000
    5000
    10000
    20000
  - Help

    Subject sequences can be removed or added from within the Descriptions tab and the selections will carry through to the other tabbed views.
    Use the formats in Download to save data for selected sequences. Manage Columns adds and subtracts data columns from the Descriptions table.
    Use the click outs to see the selected results in
    GenPept
    , Graphical Sequence Viewer
    , BLAST Tree View
    , COBALT multiple sequence alignment
    .

  - 0 sequences selected
  - sequences newly added this iteration
     Help

    sequences scoring below threshold on previous iteration
  - GenPept
  - Graphics
  - Distance tree of results
  - Multiple alignment
  - New

    MSA Viewer


  Load next setPrevious Match


  -newly added

  -used in PSSM

  Sequences with E-value BETTER than threshold 

  - select all
  - 0 sequences selected
  - Skip to the first new sequence
  - PSI-BLAST iteration 3

  Sequences producing significant alignments with E-value BETTER than threshold

  | Select for downloading or viewing reports | Description | Scientific Name | Common Name | Taxid | Max Score | Total Score | Query Cover | E value | Per. Ident | Acc. Len | Accession | Select for PSI blast | Used to build PSSM | Newly added |
  | --- | --- | --- | --- | --- | --- | --- | --- | --- | --- | --- | --- | --- | --- | --- |
  | 1Select seq gb|NBO72319.1| | proliferating cell nuclear antigen (pcna) [bacterium] | bacterium | NA | 1869227 | 227 | 227 | 97% | 1e-70 | 20.40% | 272 | NBO72319.1(scored below threshold on previous iteration) | Select seq NBO72319.1 for PSI blast |  |  |
  | 2Select seq gb|NBO72693.1| | proliferating cell nuclear antigen (pcna) [bacterium] | bacterium | NA | 1869227 | 227 | 227 | 97% | 2e-70 | 19.92% | 273 | NBO72693.1(scored below threshold on previous iteration) | Select seq NBO72693.1 for PSI blast |  |  |
  | 3Select seq gb|NDB83927.1| | proliferating cell nuclear antigen (pcna) [Alphaproteobacteria bacterium] | Alphaproteobacteria bacterium | NA | 1913988 | 227 | 454 | 97% | 2e-70 | 20.72% | 273 | NDB83927.1(scored below threshold on previous iteration) | Select seq NDB83927.1 for PSI blast |  |  |
  | 4Select seq gb|NBO60550.1| | proliferating cell nuclear antigen (pcna) [Flavobacteriia bacterium] | Flavobacteriia bacterium | NA | 2044941 | 224 | 224 | 97% | 1e-69 | 20.95% | 272 | NBO60550.1(scored below threshold on previous iteration) | Select seq NBO60550.1 for PSI blast |  |  |
  | 5Select seq gb|NBO70988.1| | proliferating cell nuclear antigen (pcna) [bacterium] | bacterium | NA | 1869227 | 224 | 224 | 97% | 2e-69 | 21.09% | 276 | NBO70988.1(scored below threshold on previous iteration) | Select seq NBO70988.1 for PSI blast |  |  |
  | 6Select seq gb|NBU17043.1| | proliferating cell nuclear antigen (pcna) [Actinobacteria bacterium] | Actinobacteria bacterium | NA | 1883427 | 223 | 223 | 98% | 6e-69 | 20.72% | 275 | NBU17043.1(scored below threshold on previous iteration) | Select seq NBU17043.1 for PSI blast |  |  |
  | 7Select seq gb|NDE16198.1| | proliferating cell nuclear antigen (pcna) [bacterium] | bacterium | NA | 1869227 | 222 | 222 | 98% | 7e-69 | 20.72% | 268 | NDE16198.1(scored below threshold on previous iteration) | Select seq NDE16198.1 for PSI blast |  |  |
  | 8Select seq gb|NBV77139.1| | proliferating cell nuclear antigen (pcna) [bacterium] | bacterium | NA | 1869227 | 221 | 221 | 97% | 2e-68 | 21.48% | 276 | NBV77139.1(scored below threshold on previous iteration) | Select seq NBV77139.1 for PSI blast |  |  |
  | 9Select seq gb|NBX51340.1| | proliferating cell nuclear antigen (pcna) [bacterium] | bacterium | NA | 1869227 | 216 | 216 | 99% | 2e-66 | 19.92% | 266 | NBX51340.1(scored below threshold on previous iteration) | Select seq NBX51340.1 for PSI blast |  |  |
  | 10Select seq gb|MTA40656.1| | proliferating cell nuclear antigen (pcna) [Actinobacteria bacterium] | Actinobacteria bacterium | NA | 1883427 | 214 | 214 | 96% | 1e-65 | 23.29% | 279 | MTA40656.1(scored below threshold on previous iteration) | Select seq MTA40656.1 for PSI blast |  |  |
  | 11Select seq gb|NDB83590.1| | proliferating cell nuclear antigen (pcna) [Alphaproteobacteria bacterium] | Alphaproteobacteria bacterium | NA | 1913988 | 214 | 214 | 97% | 2e-65 | 21.43% | 275 | NDB83590.1(scored below threshold on previous iteration) | Select seq NDB83590.1 for PSI blast |  |  |
  | 12Select seq gb|NBX51392.1| | proliferating cell nuclear antigen (pcna) [bacterium] | bacterium | NA | 1869227 | 210 | 210 | 97% | 3e-64 | 22.13% | 261 | NBX51392.1(scored below threshold on previous iteration) | Select seq NBX51392.1 for PSI blast |  |  |
  | 13Select seq gb|MBG02288.1| | proliferating cell nuclear antigen (pcna) [Acidimicrobiaceae bacterium] | Acidimicrobiaceae bacterium | NA | 2024894 | 211 | 211 | 97% | 3e-64 | 23.11% | 264 | MBG02288.1(scored below threshold on previous iteration) | Select seq MBG02288.1 for PSI blast |  |  |
  | 14Select seq gb|MBE18271.1| | proliferating cell nuclear antigen (pcna) [Nitrospinae bacterium] | Nitrospinae bacterium | NA | 2026769 | 209 | 209 | 97% | 9e-64 | 20.24% | 263 | MBE18271.1(scored below threshold on previous iteration) | Select seq MBE18271.1 for PSI blast |  |  |
  | 15Select seq gb|MAD50601.1| | proliferating cell nuclear antigen (pcna) [Flavobacteriales bacterium] | Flavobacteriales bacterium | NA | 2021391 | 209 | 209 | 96% | 2e-63 | 23.35% | 272 | MAD50601.1(scored below threshold on previous iteration) | Select seq MAD50601.1 for PSI blast |  |  |
  | 16Select seq gb|MBD23483.1| | proliferating cell nuclear antigen (pcna) [Candidatus Marinimicrobia bacterium] | Candidatus Marinimicrobia bacterium | NA | 2026760 | 208 | 208 | 97% | 2e-63 | 21.12% | 262 | MBD23483.1(scored below threshold on previous iteration) | Select seq MBD23483.1 for PSI blast |  |  |
  | 17Select seq gb|NBX48718.1| | proliferating cell nuclear antigen (pcna) [bacterium] | bacterium | NA | 1869227 | 208 | 208 | 98% | 3e-63 | 19.12% | 263 | NBX48718.1(scored below threshold on previous iteration) | Select seq NBX48718.1 for PSI blast |  |  |
  | 18Select seq gb|MBC8296603.1| | proliferating cell nuclear antigen (pcna) [Pelagibacterales bacterium] | Pelagibacterales bacterium | NA | 2026776 | 208 | 208 | 97% | 3e-63 | 22.27% | 267 | MBC8296603.1(scored below threshold on previous iteration) | Select seq MBC8296603.1 for PSI blast |  |  |
  | 19Select seq gb|NBR15457.1| | proliferating cell nuclear antigen (pcna) [Crocinitomicaceae bacterium] | Crocinitomicaceae bacterium | NA | 2026728 | 207 | 207 | 98% | 5e-63 | 22.92% | 258 | NBR15457.1(scored below threshold on previous iteration) | Select seq NBR15457.1 for PSI blast |  |  |
  | 20Select seq gb|NBR60109.1| | proliferating cell nuclear antigen (pcna) [Actinobacteria bacterium] | Actinobacteria bacterium | NA | 1883427 | 207 | 207 | 97% | 9e-63 | 22.35% | 292 | NBR60109.1(scored below threshold on previous iteration) | Select seq NBR60109.1 for PSI blast |  |  |
  | 21Select seq gb|PQM59040.1| | proliferating cell nuclear antigen (pcna) [Rhodobacteraceae bacterium] | Rhodobacteraceae bacterium | NA | 1904441 | 207 | 207 | 97% | 1e-62 | 23.11% | 274 | PQM59040.1(scored below threshold on previous iteration) | Select seq PQM59040.1 for PSI blast |  |  |
  | 22Select seq gb|MBD23789.1| | proliferating cell nuclear antigen (pcna) [Candidatus Marinimicrobia bacterium] | Candidatus Marinimicrobia bacterium | NA | 2026760 | 206 | 206 | 98% | 1e-62 | 20.95% | 260 | MBD23789.1(scored below threshold on previous iteration) | Select seq MBD23789.1 for PSI blast |  |  |
  | 23Select seq gb|NBX48943.1| | proliferating cell nuclear antigen (pcna) [bacterium] | bacterium | NA | 1869227 | 206 | 206 | 98% | 1e-62 | 22.22% | 246 | NBX48943.1(scored below threshold on previous iteration) | Select seq NBX48943.1 for PSI blast |  |  |
  | 24Select seq gb|NDG29484.1| | proliferating cell nuclear antigen (pcna) [bacterium] | bacterium | NA | 1869227 | 205 | 205 | 97% | 3e-62 | 21.20% | 261 | NDG29484.1(scored below threshold on previous iteration) | Select seq NDG29484.1 for PSI blast |  |  |
  | 25Select seq gb|OYV74989.1| | proliferating cell nuclear antigen (pcna) [Chromatiales bacterium 21-64-14] | Chromatiales bacterium 21-64-14 | NA | 1970504 | 206 | 206 | 97% | 8e-62 | 18.05% | 312 | OYV74989.1(scored below threshold on previous iteration) | Select seq OYV74989.1 for PSI blast |  |  |
  | 26Select seq gb|RPG58329.1| | proliferating cell nuclear antigen (pcna) [Flavobacteriales bacterium TMED191] | Flavobacteriales bacterium TMED191 | NA | 1986723 | 204 | 204 | 96% | 1e-61 | 22.96% | 273 | RPG58329.1(scored below threshold on previous iteration) | Select seq RPG58329.1 for PSI blast |  |  |
  | 27Select seq gb|NBR61302.1| | proliferating cell nuclear antigen (pcna) [Actinobacteria bacterium] | Actinobacteria bacterium | NA | 1883427 | 204 | 204 | 97% | 1e-61 | 20.80% | 261 | NBR61302.1(scored below threshold on previous iteration) | Select seq NBR61302.1 for PSI blast |  |  |
  | 28Select seq gb|NBO53670.1| | proliferating cell nuclear antigen (pcna) [Actinobacteria bacterium] | Actinobacteria bacterium | NA | 1883427 | 204 | 204 | 96% | 1e-61 | 22.75% | 284 | NBO53670.1(scored below threshold on previous iteration) | Select seq NBO53670.1 for PSI blast |  |  |
  | 29Select seq gb|MAD12048.1| | proliferating cell nuclear antigen (pcna) [Flavobacteriaceae bacterium] | Flavobacteriaceae bacterium | NA | 1871037 | 204 | 204 | 96% | 2e-61 | 22.75% | 289 | MAD12048.1(scored below threshold on previous iteration) | Select seq MAD12048.1 for PSI blast |  |  |
  | 30Select seq gb|OUU62278.1| | proliferating cell nuclear antigen (pcna) [Alphaproteobacteria bacterium TMED62] | Alphaproteobacteria bacterium TMED62 | NA | 1986642 | 203 | 203 | 96% | 4e-61 | 21.37% | 278 | OUU62278.1(scored below threshold on previous iteration) | Select seq OUU62278.1 for PSI blast |  |  |
  | 31Select seq ref|WP\_162815250.1| | proliferating cell nuclear antigen (pcna) [Microbacterium arborescens] | Microbacterium arborescens | NA | 33883 | 199 | 199 | 95% | 4e-60 | 22.36% | 249 | WP\_162815250.1(scored below threshold on previous iteration) | Select seq WP\_162815250.1 for PSI blast |  |  |
  | 32Select seq gb|NDC41159.1| | proliferating cell nuclear antigen (pcna) [Chitinophagia bacterium] | Chitinophagia bacterium | NA | 2448778 | 199 | 199 | 97% | 7e-60 | 22.00% | 259 | NDC41159.1(scored below threshold on previous iteration) | Select seq NDC41159.1 for PSI blast |  |  |
  | 33Select seq gb|MAT63990.1| | proliferating cell nuclear antigen (pcna) [Dehalococcoidia bacterium] | Dehalococcoidia bacterium | NA | 2026734 | 198 | 198 | 99% | 1e-59 | 21.51% | 246 | MAT63990.1(scored below threshold on previous iteration) | Select seq MAT63990.1 for PSI blast |  |  |
  | 34Select seq gb|MAW08577.1| | proliferating cell nuclear antigen (pcna) [Halobacteriovoraceae bacterium] | Halobacteriovoraceae bacterium | NA | 2026745 | 199 | 199 | 96% | 1e-59 | 22.39% | 272 | MAW08577.1(scored below threshold on previous iteration) | Select seq MAW08577.1 for PSI blast |  |  |
  | 35Select seq gb|NBO71470.1| | proliferating cell nuclear antigen (pcna) [bacterium] | bacterium | NA | 1869227 | 199 | 199 | 96% | 3e-59 | 19.61% | 300 | NBO71470.1(scored below threshold on previous iteration) | Select seq NBO71470.1 for PSI blast |  |  |
  | 36Select seq gb|MAZ55206.1| | proliferating cell nuclear antigen (pcna) [Flavobacteriales bacterium] | Flavobacteriales bacterium | NA | 2021391 | 197 | 197 | 96% | 4e-59 | 17.65% | 268 | MAZ55206.1(scored below threshold on previous iteration) | Select seq MAZ55206.1 for PSI blast |  |  |
  | 37Select seq gb|MAU36389.1| | proliferating cell nuclear antigen (pcna) [Flavobacteriales bacterium] | Flavobacteriales bacterium | NA | 2021391 | 197 | 197 | 96% | 6e-59 | 23.94% | 279 | MAU36389.1(scored below threshold on previous iteration) | Select seq MAU36389.1 for PSI blast |  |  |
  | 38Select seq gb|MAT00017.1| | proliferating cell nuclear antigen (pcna) [Rhodobacteraceae bacterium] | Rhodobacteraceae bacterium | NA | 1904441 | 196 | 196 | 99% | 7e-59 | 20.32% | 246 | MAT00017.1(scored below threshold on previous iteration) | Select seq MAT00017.1 for PSI blast |  |  |
  | 39Select seq gb|OUU16001.1| | proliferating cell nuclear antigen (pcna) [Candidatus Endolissoclinum sp. TMED37] | Candidatus Endolissoclinum sp. TMED37 | NA | 1986638 | 197 | 197 | 96% | 8e-59 | 17.25% | 268 | OUU16001.1(scored below threshold on previous iteration) | Select seq OUU16001.1 for PSI blast |  |  |
  | 40Select seq tpg|HEN27889.1| | TPA: proliferating cell nuclear antigen (pcna) [candidate division WOR-3 bacterium] | candidate division WOR-3 bacterium | NA | 2052148 | 196 | 196 | 98% | 1e-58 | 33.47% | 251 | HEN27889.1(scored below threshold on previous iteration) | Select seq HEN27889.1 for PSI blast |  |  |
  | 41Select seq gb|MBP37067.1| | proliferating cell nuclear antigen (pcna) [Candidatus Pelagibacter sp.] | Candidatus Pelagibacter sp. | NA | 2024849 | 196 | 196 | 99% | 1e-58 | 29.72% | 248 | MBP37067.1(scored below threshold on previous iteration) | Select seq MBP37067.1 for PSI blast |  |  |
  | 42Select seq gb|NBT47941.1| | proliferating cell nuclear antigen (pcna) [Actinobacteria bacterium] | Actinobacteria bacterium | NA | 1883427 | 196 | 196 | 97% | 1e-58 | 21.29% | 267 | NBT47941.1(scored below threshold on previous iteration) | Select seq NBT47941.1 for PSI blast |  |  |
  | 43Select seq gb|NBP59184.1| | proliferating cell nuclear antigen (pcna) [bacterium] | bacterium | NA | 1869227 | 196 | 196 | 96% | 1e-58 | 19.68% | 263 | NBP59184.1(scored below threshold on previous iteration) | Select seq NBP59184.1 for PSI blast |  |  |
  | 44Select seq gb|MBR74595.1| | proliferating cell nuclear antigen (pcna) [Dehalococcoidaceae bacterium] | Dehalococcoidaceae bacterium | NA | 2026731 | 195 | 195 | 99% | 2e-58 | 31.33% | 248 | MBR74595.1(scored below threshold on previous iteration) | Select seq MBR74595.1 for PSI blast |  |  |
  | 45Select seq gb|NDB81722.1| | proliferating cell nuclear antigen (pcna) [Alphaproteobacteria bacterium] | Alphaproteobacteria bacterium | NA | 1913988 | 196 | 196 | 97% | 2e-58 | 20.24% | 261 | NDB81722.1(scored below threshold on previous iteration) | Select seq NDB81722.1 for PSI blast |  |  |
  | 46Select seq gb|MBP02103.1| | proliferating cell nuclear antigen (pcna) [Rhodospirillaceae bacterium] | Rhodospirillaceae bacterium | NA | 1898112 | 195 | 195 | 99% | 2e-58 | 22.53% | 254 | MBP02103.1(scored below threshold on previous iteration) | Select seq MBP02103.1 for PSI blast |  |  |
  | 47Select seq gb|PCH85639.1| | proliferating cell nuclear antigen (pcna) [Dehalococcoidia bacterium] | Dehalococcoidia bacterium | NA | 2026734 | 195 | 195 | 100% | 3e-58 | 28.29% | 252 | PCH85639.1(scored below threshold on previous iteration) | Select seq PCH85639.1 for PSI blast |  |  |
  | 48Select seq gb|NDB84018.1| | proliferating cell nuclear antigen (pcna) [Alphaproteobacteria bacterium] | Alphaproteobacteria bacterium | NA | 1913988 | 193 | 193 | 97% | 2e-57 | 23.32% | 266 | NDB84018.1(scored below threshold on previous iteration) | Select seq NDB84018.1 for PSI blast |  |  |
  | 49Select seq gb|NBX51618.1| | proliferating cell nuclear antigen (pcna) [bacterium] | bacterium | NA | 1869227 | 192 | 192 | 98% | 2e-57 | 21.34% | 250 | NBX51618.1(scored below threshold on previous iteration) | Select seq NBX51618.1 for PSI blast |  |  |
  | 50Select seq gb|MAB60694.1| | proliferating cell nuclear antigen (pcna) [Verrucomicrobiales bacterium] | Verrucomicrobiales bacterium | NA | 2026801 | 193 | 193 | 99% | 3e-57 | 21.34% | 267 | MAB60694.1(scored below threshold on previous iteration) | Select seq MAB60694.1 for PSI blast |  |  |
  | 51Select seq gb|NDG72174.1| | proliferating cell nuclear antigen (pcna) [Proteobacteria bacterium] | Proteobacteria bacterium | NA | 1977087 | 191 | 191 | 96% | 1e-56 | 22.35% | 267 | NDG72174.1(scored below threshold on previous iteration) | Select seq NDG72174.1 for PSI blast |  |  |
  | 52Select seq gb|RPH19578.1| | proliferating cell nuclear antigen (pcna) [Alteromonadaceae bacterium TMED7] | Alteromonadaceae bacterium TMED7 | NA | 1986650 | 189 | 189 | 97% | 3e-56 | 18.55% | 247 | RPH19578.1(scored below threshold on previous iteration) | Select seq RPH19578.1 for PSI blast |  |  |
  | 53Select seq gb|NBR16174.1| | proliferating cell nuclear antigen (pcna) [Crocinitomicaceae bacterium] | Crocinitomicaceae bacterium | NA | 2026728 | 189 | 189 | 95% | 6e-56 | 22.92% | 270 | NBR16174.1(scored below threshold on previous iteration) | Select seq NBR16174.1 for PSI blast |  |  |
  | 54Select seq gb|MAR51068.1| | proliferating cell nuclear antigen (pcna) [Rhodobacteraceae bacterium] | Rhodobacteraceae bacterium | NA | 1904441 | 189 | 189 | 97% | 1e-55 | 20.45% | 277 | MAR51068.1(scored below threshold on previous iteration) | Select seq MAR51068.1 for PSI blast |  |  |
  | 55Select seq gb|MAF45402.1| | proliferating cell nuclear antigen (pcna) [Acidimicrobiaceae bacterium] | Acidimicrobiaceae bacterium | NA | 2024894 | 188 | 188 | 98% | 1e-55 | 20.48% | 246 | MAF45402.1(scored below threshold on previous iteration) | Select seq MAF45402.1 for PSI blast |  |  |
  | 56Select seq gb|OLD30892.1| | proliferating cell nuclear antigen (pcna) [Candidatus Rokubacteria bacterium 13\_1\_40CM\_2\_70\_45] | Candidatus Rokubacteria bacterium 13\_1\_40CM\_2\_70\_45 | NA | 1805357 | 186 | 186 | 98% | 4e-55 | 32.66% | 246 | OLD30892.1(scored below threshold on previous iteration) | Select seq OLD30892.1 for PSI blast |  |  |
  | 57Select seq gb|MAE55769.1| | proliferating cell nuclear antigen (pcna) [Porticoccaceae bacterium] | Porticoccaceae bacterium | NA | 2026782 | 186 | 186 | 98% | 9e-55 | 23.53% | 253 | MAE55769.1(scored below threshold on previous iteration) | Select seq MAE55769.1 for PSI blast |  |  |
  | 58Select seq gb|MBI96915.1| | proliferating cell nuclear antigen (pcna) [bacterium] | bacterium | NA | 1869227 | 186 | 186 | 96% | 9e-55 | 21.18% | 273 | MBI96915.1(scored below threshold on previous iteration) | Select seq MBI96915.1 for PSI blast |  |  |
  | 59Select seq gb|OUU49664.1| | proliferating cell nuclear antigen (pcna) [Candidatus Puniceispirillum sp. TMED52] | Candidatus Puniceispirillum sp. TMED52 | NA | 1986639 | 186 | 186 | 99% | 1e-54 | 22.01% | 263 | OUU49664.1(scored below threshold on previous iteration) | Select seq OUU49664.1 for PSI blast |  |  |
  | 60Select seq gb|OLD25330.1| | proliferating cell nuclear antigen (pcna) [Candidatus Rokubacteria bacterium 13\_1\_40CM\_2\_70\_45] | Candidatus Rokubacteria bacterium 13\_1\_40CM\_2\_70\_45 | NA | 1805357 | 184 | 184 | 98% | 6e-54 | 33.06% | 245 | OLD25330.1(scored below threshold on previous iteration) | Select seq OLD25330.1 for PSI blast |  |  |
  | 61Select seq gb|NBR26233.1| | proliferating cell nuclear antigen (pcna) [Micrococcales bacterium] | Micrococcales bacterium | NA | 2026762 | 182 | 182 | 99% | 3e-53 | 23.05% | 260 | NBR26233.1(scored below threshold on previous iteration) | Select seq NBR26233.1 for PSI blast |  |  |
  | 62Select seq gb|MBJ75944.1| | proliferating cell nuclear antigen (pcna) [Planctomycetes bacterium] | Planctomycetes bacterium | NA | 2026780 | 182 | 182 | 97% | 3e-53 | 25.20% | 248 | MBJ75944.1(scored below threshold on previous iteration) | Select seq MBJ75944.1 for PSI blast |  |  |
  | 63Select seq gb|NIW10639.1| | proliferating cell nuclear antigen (pcna) [Gammaproteobacteria bacterium] | Gammaproteobacteria bacterium | NA | 1913989 | 181 | 181 | 97% | 5e-53 | 33.61% | 246 | NIW10639.1(scored below threshold on previous iteration) | Select seq NIW10639.1 for PSI blast |  |  |
  | 64Select seq gb|MBI2034871.1| | proliferating cell nuclear antigen (pcna) [Candidatus Levybacteria bacterium] | Candidatus Levybacteria bacterium | NA | 2052151 | 181 | 181 | 99% | 7e-53 | 35.08% | 249 | MBI2034871.1(scored below threshold on previous iteration) | Select seq MBI2034871.1 for PSI blast |  |  |
  | 65Select seq gb|MAD20416.1| | proliferating cell nuclear antigen (pcna) [Planctomycetaceae bacterium] | Planctomycetaceae bacterium | NA | 2026779 | 178 | 178 | 97% | 1e-51 | 21.37% | 262 | MAD20416.1(scored below threshold on previous iteration) | Select seq MAD20416.1 for PSI blast |  |  |
  | 66Select seq gb|NBP13185.1| | proliferating cell nuclear antigen (pcna) [bacterium] | bacterium | NA | 1869227 | 175 | 175 | 96% | 3e-50 | 19.55% | 287 | NBP13185.1(scored below threshold on previous iteration) | Select seq NBP13185.1 for PSI blast |  |  |
  | 67Select seq gb|NBU33707.1| | proliferating cell nuclear antigen (pcna) [bacterium] | bacterium | NA | 1869227 | 173 | 173 | 96% | 2e-49 | 19.55% | 277 | NBU33707.1(scored below threshold on previous iteration) | Select seq NBU33707.1 for PSI blast |  |  |
  | 68Select seq gb|NBP00213.1| | proliferating cell nuclear antigen (pcna) [Proteobacteria bacterium] | Proteobacteria bacterium | NA | 1977087 | 173 | 173 | 98% | 3e-49 | 19.56% | 297 | NBP00213.1(scored below threshold on previous iteration) | Select seq NBP00213.1 for PSI blast |  |  |
  | 69Select seq gb|KKW29271.1| | polymerase sliding clamp protein [Parcubacteria group bacterium GW2011\_GWB1\_52\_7] | Parcubacteria group bacterium GW2011\_GWB1\_52\_7 | NA | 1618885 | 171 | 171 | 98% | 4e-49 | 32.93% | 247 | KKW29271.1(scored below threshold on previous iteration) | Select seq KKW29271.1 for PSI blast |  |  |
  | 70Select seq gb|NDC95013.1| | proliferating cell nuclear antigen (pcna) [bacterium] | bacterium | NA | 1869227 | 172 | 172 | 99% | 5e-49 | 20.59% | 278 | NDC95013.1(scored below threshold on previous iteration) | Select seq NDC95013.1 for PSI blast |  |  |
  | 71Select seq tpg|HDQ16435.1| | TPA: proliferating cell nuclear antigen (pcna) [Bacteroidetes bacterium] | Bacteroidetes bacterium | NA | 1898104 | 167 | 167 | 98% | 1e-47 | 30.65% | 245 | HDQ16435.1(scored below threshold on previous iteration) | Select seq HDQ16435.1 for PSI blast |  |  |
  | 72Select seq gb|NCA73651.1| | proliferating cell nuclear antigen (pcna) [Gammaproteobacteria bacterium] | Gammaproteobacteria bacterium | NA | 1913989 | 167 | 167 | 96% | 2e-47 | 27.16% | 245 | NCA73651.1(scored below threshold on previous iteration) | Select seq NCA73651.1 for PSI blast |  |  |
  | 73Select seq gb|MRR35945.1| | proliferating cell nuclear antigen (pcna) [bacterium] | bacterium | NA | 1869227 | 167 | 167 | 98% | 2e-47 | 27.82% | 244 | MRR35945.1(scored below threshold on previous iteration) | Select seq MRR35945.1 for PSI blast |  |  |
  | 74Select seq gb|MAI67428.1| | hypothetical protein [Phycisphaerae bacterium] | Phycisphaerae bacterium | NA | 2026778 | 167 | 167 | 97% | 3e-47 | 26.23% | 252 | MAI67428.1(scored below threshold on previous iteration) | Select seq MAI67428.1 for PSI blast |  |  |
  | 75Select seq gb|MBE9487910.1| | proliferating cell nuclear antigen (pcna) [Bacteroidetes bacterium] | Bacteroidetes bacterium | NA | 1898104 | 167 | 167 | 98% | 3e-47 | 27.82% | 245 | MBE9487910.1(scored below threshold on previous iteration) | Select seq MBE9487910.1 for PSI blast |  |  |
  | 76Select seq gb|NPA85815.1| | proliferating cell nuclear antigen (pcna) [bacterium] | bacterium | NA | 1869227 | 167 | 167 | 100% | 3e-47 | 27.63% | 254 | NPA85815.1(scored below threshold on previous iteration) | Select seq NPA85815.1 for PSI blast |  |  |
  | 77Select seq gb|NTV78099.1| | proliferating cell nuclear antigen (pcna) [Clostridiales bacterium] | Clostridiales bacterium | NA | 1898207 | 166 | 166 | 97% | 4e-47 | 30.61% | 245 | NTV78099.1(scored below threshold on previous iteration) | Select seq NTV78099.1 for PSI blast |  |  |
  | 78Select seq gb|MBI63664.1| | hypothetical protein [Chloroflexi bacterium] | Chloroflexi bacterium | NA | 2026724 | 162 | 162 | 97% | 2e-45 | 22.76% | 248 | MBI63664.1(scored below threshold on previous iteration) | Select seq MBI63664.1 for PSI blast |  |  |
  | 79Select seq gb|MBA42970.1| | proliferating cell nuclear antigen (pcna) [Magnetococcales bacterium] | Magnetococcales bacterium | NA | 2026759 | 163 | 163 | 97% | 5e-45 | 16.84% | 327 | MBA42970.1(scored below threshold on previous iteration) | Select seq MBA42970.1 for PSI blast |  |  |
  | 80Select seq tpg|HEC38232.1| | TPA: proliferating cell nuclear antigen (pcna) [bacterium] | bacterium | NA | 1869227 | 159 | 159 | 100% | 3e-44 | 29.96% | 266 | HEC38232.1(scored below threshold on previous iteration) | Select seq HEC38232.1 for PSI blast |  |  |
  | 81Select seq gb|MBN37929.1| | proliferating cell nuclear antigen (pcna) [Opitutae bacterium] | Opitutae bacterium | NA | 2026771 | 164 | 164 | 97% | 5e-44 | 19.92% | 465 | MBN37929.1(scored below threshold on previous iteration) | Select seq MBN37929.1 for PSI blast |  |  |
  | 82Select seq gb|MAE80883.1| | DNA polymerase sliding clamp [Chloroflexi bacterium] | Chloroflexi bacterium | NA | 2026724 | 157 | 157 | 96% | 1e-43 | 26.21% | 253 | MAE80883.1(scored below threshold on previous iteration) | Select seq MAE80883.1 for PSI blast |  |  |
  | 83Select seq gb|MAN39109.1| | hypothetical protein [Gammaproteobacteria bacterium] | Gammaproteobacteria bacterium | NA | 1913989 | 157 | 157 | 97% | 1e-43 | 23.58% | 249 | MAN39109.1(scored below threshold on previous iteration) | Select seq MAN39109.1 for PSI blast |  |  |
  | 84Select seq gb|NBT48094.1| | proliferating cell nuclear antigen (pcna) [Actinobacteria bacterium] | Actinobacteria bacterium | NA | 1883427 | 155 | 155 | 99% | 5e-43 | 24.40% | 248 | NBT48094.1(scored below threshold on previous iteration) | Select seq NBT48094.1 for PSI blast |  |  |
  | 85Select seq gb|MAI14456.1| | proliferating cell nuclear antigen (pcna) [Rhodospirillaceae bacterium] | Rhodospirillaceae bacterium | NA | 1898112 | 155 | 155 | 99% | 1e-42 | 22.61% | 261 | MAI14456.1(scored below threshold on previous iteration) | Select seq MAI14456.1 for PSI blast |  |  |
  | 86Select seq gb|MBH51453.1| | hypothetical protein [Candidatus Marinimicrobia bacterium] | Candidatus Marinimicrobia bacterium | NA | 2026760 | 154 | 154 | 96% | 3e-42 | 24.08% | 248 | MBH51453.1(scored below threshold on previous iteration) | Select seq MBH51453.1 for PSI blast |  |  |
  | 87Select seq gb|OLD29434.1| | DNA polymerase sliding clamp [Candidatus Rokubacteria bacterium 13\_1\_40CM\_2\_70\_45] | Candidatus Rokubacteria bacterium 13\_1\_40CM\_2\_70\_45 | NA | 1805357 | 152 | 152 | 80% | 4e-42 | 32.02% | 201 | OLD29434.1(scored below threshold on previous iteration) | Select seq OLD29434.1 for PSI blast |  |  |
  | 88Select seq gb|MBB18791.1| | proliferating cell nuclear antigen (pcna) [Rickettsiales bacterium] | Rickettsiales bacterium | NA | 2026788 | 155 | 155 | 97% | 5e-42 | 20.24% | 299 | MBB18791.1(scored below threshold on previous iteration) | Select seq MBB18791.1 for PSI blast |  |  |
  | 89Select seq gb|OUU15212.1| | proliferating cell nuclear antigen (pcna) [Candidatus Endolissoclinum sp. TMED37] | Candidatus Endolissoclinum sp. TMED37 | NA | 1986638 | 153 | 153 | 96% | 8e-42 | 19.29% | 264 | OUU15212.1(scored below threshold on previous iteration) | Select seq OUU15212.1 for PSI blast |  |  |
  | 90Select seq gb|NDG27889.1| | proliferating cell nuclear antigen (pcna) [Proteobacteria bacterium] | Proteobacteria bacterium | NA | 1977087 | 154 | 154 | 98% | 1e-41 | 21.03% | 293 | NDG27889.1(scored below threshold on previous iteration) | Select seq NDG27889.1 for PSI blast |  |  |
  | 91Select seq gb|MBQ40860.1| | DNA polymerase sliding clamp [Gemmatimonadetes bacterium] | Gemmatimonadetes bacterium | NA | 2026742 | 152 | 152 | 96% | 2e-41 | 28.23% | 258 | MBQ40860.1(scored below threshold on previous iteration) | Select seq MBQ40860.1 for PSI blast |  |  |
  | 92Select seq gb|OLC90189.1| | DNA polymerase sliding clamp [Candidatus Rokubacteria bacterium 13\_1\_40CM\_3\_69\_38] | Candidatus Rokubacteria bacterium 13\_1\_40CM\_3\_69\_38 | NA | 1805359 | 150 | 150 | 80% | 2e-41 | 32.02% | 200 | OLC90189.1(scored below threshold on previous iteration) | Select seq OLC90189.1 for PSI blast |  |  |
  | 93Select seq gb|NCS98517.1| | hypothetical protein [bacterium] | bacterium | NA | 1869227 | 152 | 152 | 98% | 2e-41 | 27.24% | 245 | NCS98517.1(scored below threshold on previous iteration) | Select seq NCS98517.1 for PSI blast |  |  |
  | 94Select seq gb|NBR60072.1| | proliferating cell nuclear antigen (pcna) [Actinobacteria bacterium] | Actinobacteria bacterium | NA | 1883427 | 152 | 152 | 98% | 4e-41 | 21.03% | 293 | NBR60072.1(scored below threshold on previous iteration) | Select seq NBR60072.1 for PSI blast |  |  |
  | 95Select seq gb|MBN20023.1| | proliferating cell nuclear antigen (pcna) [Bdellovibrionaceae bacterium] | Bdellovibrionaceae bacterium | NA | 2026715 | 151 | 151 | 97% | 6e-41 | 19.39% | 270 | MBN20023.1(scored below threshold on previous iteration) | Select seq MBN20023.1 for PSI blast |  |  |
  | 96Select seq gb|MAB04571.1| | proliferating cell nuclear antigen (pcna) [Rhodobacteraceae bacterium] | Rhodobacteraceae bacterium | NA | 1904441 | 150 | 150 | 95% | 7e-41 | 23.14% | 256 | MAB04571.1(scored below threshold on previous iteration) | Select seq MAB04571.1 for PSI blast |  |  |
  | 97Select seq gb|KKR01610.1| | polymerase sliding clamp protein [Candidatus Woesebacteria bacterium GW2011\_GWB1\_39\_12] | Candidatus Woesebacteria bacterium GW2011\_GWB1\_39\_12 | NA | 1618574 | 151 | 151 | 99% | 7e-41 | 24.43% | 294 | KKR01610.1(scored below threshold on previous iteration) | Select seq KKR01610.1 for PSI blast |  |  |
  | 98Select seq gb|NDA89590.1| | proliferating cell nuclear antigen (pcna) [Alphaproteobacteria bacterium] | Alphaproteobacteria bacterium | NA | 1913988 | 151 | 151 | 95% | 9e-41 | 22.54% | 280 | NDA89590.1(scored below threshold on previous iteration) | Select seq NDA89590.1 for PSI blast |  |  |
  | 99Select seq gb|NIQ14317.1| | DNA polymerase sliding clamp [Candidatus Dadabacteria bacterium] | Candidatus Dadabacteria bacterium | NA | 2080303 | 148 | 148 | 96% | 5e-40 | 23.27% | 254 | NIQ14317.1(scored below threshold on previous iteration) | Select seq NIQ14317.1 for PSI blast |  |  |
  | 100Select seq gb|OLC55625.1| | DNA polymerase sliding clamp [Candidatus Rokubacteria bacterium 13\_1\_40CM\_4\_69\_39] | Candidatus Rokubacteria bacterium 13\_1\_40CM\_4\_69\_39 | NA | 1805363 | 143 | 143 | 76% | 1e-38 | 32.12% | 190 | OLC55625.1(scored below threshold on previous iteration) | Select seq OLC55625.1 for PSI blast |  |  |
  | 101Select seq tpg|HIQ51620.1| | TPA: hypothetical protein [Nautiliaceae bacterium] | Nautiliaceae bacterium | NA | 2306051 | 144 | 144 | 98% | 2e-38 | 31.05% | 246 | HIQ51620.1(scored below threshold on previous iteration) | Select seq HIQ51620.1 for PSI blast |  |  |
  | 102Select seq gb|OGO49108.1| | hypothetical protein A2W34\_06430 [Chloroflexi bacterium RBG\_16\_64\_32] | Chloroflexi bacterium RBG\_16\_64\_32 | NA | 1797658 | 147 | 147 | 98% | 2e-38 | 22.57% | 365 | OGO49108.1(scored below threshold on previous iteration) | Select seq OGO49108.1 for PSI blast |  |  |
  | 103Select seq gb|MAL04566.1| | hypothetical protein [Rhodobiaceae bacterium] | Rhodobiaceae bacterium | NA | 2026785 | 143 | 143 | 97% | 3e-38 | 23.67% | 246 | MAL04566.1(scored below threshold on previous iteration) | Select seq MAL04566.1 for PSI blast |  |  |
  | 104Select seq gb|MBC8306902.1| | proliferating cell nuclear antigen (pcna) [Pelagibacterales bacterium] | Pelagibacterales bacterium | NA | 2026776 | 142 | 142 | 100% | 1e-37 | 16.67% | 276 | MBC8306902.1(scored below threshold on previous iteration) | Select seq MBC8306902.1 for PSI blast |  |  |
  | 105Select seq tpg|HGO27509.1| | TPA: proliferating cell nuclear antigen (pcna) [Proteobacteria bacterium] | Proteobacteria bacterium | NA | 1977087 | 140 | 140 | 97% | 5e-37 | 22.95% | 249 | HGO27509.1(scored below threshold on previous iteration) | Select seq HGO27509.1 for PSI blast |  |  |
  | 106Select seq gb|MBE29542.1| | hypothetical protein [bacterium] | bacterium | NA | 1869227 | 141 | 141 | 97% | 5e-37 | 19.68% | 275 | MBE29542.1(scored below threshold on previous iteration) | Select seq MBE29542.1 for PSI blast |  |  |
  | 107Select seq gb|NIA11429.1| | DNA polymerase sliding clamp [Nitrospiraceae bacterium] | Nitrospiraceae bacterium | NA | 2026770 | 140 | 140 | 97% | 6e-37 | 29.27% | 242 | NIA11429.1(scored below threshold on previous iteration) | Select seq NIA11429.1 for PSI blast |  |  |
  | 108Select seq gb|MAR49569.1| | proliferating cell nuclear antigen (pcna) [Rhodobacteraceae bacterium] | Rhodobacteraceae bacterium | NA | 1904441 | 137 | 137 | 97% | 9e-36 | 16.54% | 268 | MAR49569.1(scored below threshold on previous iteration) | Select seq MAR49569.1 for PSI blast |  |  |
  | 109Select seq tpg|HFO52032.1| | TPA: proliferating cell nuclear antigen (pcna) [Ignavibacteria bacterium] | Ignavibacteria bacterium | NA | 2053306 | 140 | 140 | 85% | 2e-35 | 17.33% | 428 | HFO52032.1(scored below threshold on previous iteration) | Select seq HFO52032.1 for PSI blast |  |  |
  | 110Select seq gb|NDE15318.1| | hypothetical protein [bacterium] | bacterium | NA | 1869227 | 135 | 135 | 97% | 6e-35 | 23.53% | 273 | NDE15318.1(scored below threshold on previous iteration) | Select seq NDE15318.1 for PSI blast |  |  |
  | 111Select seq gb|NDE18324.1| | hypothetical protein [bacterium] | bacterium | NA | 1869227 | 135 | 135 | 98% | 1e-34 | 21.74% | 282 | NDE18324.1(scored below threshold on previous iteration) | Select seq NDE18324.1 for PSI blast |  |  |
  | 112Select seq gb|MAV57173.1| | proliferating cell nuclear antigen (pcna) [Candidatus Pelagibacter sp.] | Candidatus Pelagibacter sp. | NA | 2024849 | 134 | 134 | 96% | 1e-34 | 24.19% | 260 | MAV57173.1(scored below threshold on previous iteration) | Select seq MAV57173.1 for PSI blast |  |  |
  | 113Select seq gb|NDA64219.1| | proliferating cell nuclear antigen (pcna) [Chitinophagia bacterium] | Chitinophagia bacterium | NA | 2448778 | 132 | 132 | 76% | 2e-34 | 19.49% | 209 | NDA64219.1(scored below threshold on previous iteration) | Select seq NDA64219.1 for PSI blast |  |  |
  | 114Select seq gb|RLC83458.1| | hypothetical protein DRI37\_10220 [Chloroflexi bacterium] | Chloroflexi bacterium | NA | 2026724 | 131 | 131 | 86% | 9e-34 | 32.41% | 215 | RLC83458.1(scored below threshold on previous iteration) | Select seq RLC83458.1 for PSI blast |  |  |
  | 115Select seq gb|MAR96207.1| | hypothetical protein [Candidatus Marinimicrobia bacterium] | Candidatus Marinimicrobia bacterium | NA | 2026760 | 132 | 132 | 95% | 1e-33 | 23.17% | 268 | MAR96207.1(scored below threshold on previous iteration) | Select seq MAR96207.1 for PSI blast |  |  |
  | 116Select seq gb|NDG54324.1| | proliferating cell nuclear antigen (pcna) [Flavobacteriia bacterium] | Flavobacteriia bacterium | NA | 2044941 | 133 | 133 | 97% | 2e-33 | 16.95% | 308 | NDG54324.1(scored below threshold on previous iteration) | Select seq NDG54324.1 for PSI blast |  |  |
  | 117Select seq gb|NDA89441.1| | proliferating cell nuclear antigen (pcna) [Alphaproteobacteria bacterium] | Alphaproteobacteria bacterium | NA | 1913988 | 128 | 128 | 71% | 3e-33 | 18.23% | 196 | NDA89441.1(scored below threshold on previous iteration) | Select seq NDA89441.1 for PSI blast |  |  |
  | 118Select seq gb|RYX78042.1| | hypothetical protein EON71\_01370 [bacterium] | bacterium | NA | 1869227 | 132 | 132 | 96% | 4e-33 | 14.98% | 327 | RYX78042.1(scored below threshold on previous iteration) | Select seq RYX78042.1 for PSI blast |  |  |
  | 119Select seq gb|MAR96282.1| | hypothetical protein [Candidatus Marinimicrobia bacterium] | Candidatus Marinimicrobia bacterium | NA | 2026760 | 130 | 130 | 95% | 9e-33 | 23.58% | 268 | MAR96282.1(scored below threshold on previous iteration) | Select seq MAR96282.1 for PSI blast |  |  |
  | 120Select seq gb|MAR17754.1| | hypothetical protein [Rhodobacteraceae bacterium] | Rhodobacteraceae bacterium | NA | 1904441 | 129 | 129 | 95% | 9e-33 | 24.37% | 249 | MAR17754.1(scored below threshold on previous iteration) | Select seq MAR17754.1 for PSI blast |  |  |
  | 121Select seq gb|MAP67643.1| | proliferating cell nuclear antigen (pcna) [Candidatus Marinimicrobia bacterium] | Candidatus Marinimicrobia bacterium | NA | 2026760 | 128 | 128 | 95% | 2e-32 | 22.40% | 261 | MAP67643.1(scored below threshold on previous iteration) | Select seq MAP67643.1 for PSI blast |  |  |
  | 122Select seq gb|OEU74059.1| | hypothetical protein BA864\_06935 [Desulfuromonadales bacterium C00003093] | Desulfuromonadales bacterium C00003093 | NA | 1869308 | 128 | 128 | 99% | 3e-32 | 22.53% | 247 | OEU74059.1(scored below threshold on previous iteration) | Select seq OEU74059.1 for PSI blast |  |  |
  | 123Select seq gb|MAI14441.1| | hypothetical protein [Rhodospirillaceae bacterium] | Rhodospirillaceae bacterium | NA | 1898112 | 128 | 128 | 99% | 4e-32 | 20.38% | 267 | MAI14441.1(scored below threshold on previous iteration) | Select seq MAI14441.1 for PSI blast |  |  |
  | 124Select seq gb|MAP62052.1| | hypothetical protein [Candidatus Marinimicrobia bacterium] | Candidatus Marinimicrobia bacterium | NA | 2026760 | 128 | 128 | 97% | 5e-32 | 15.14% | 260 | MAP62052.1(scored below threshold on previous iteration) | Select seq MAP62052.1 for PSI blast |  |  |
  | 125Select seq gb|NDB86579.1| | hypothetical protein [Alphaproteobacteria bacterium] | Alphaproteobacteria bacterium | NA | 1913988 | 123 | 123 | 60% | 1e-31 | 14.74% | 159 | NDB86579.1(scored below threshold on previous iteration) | Select seq NDB86579.1 for PSI blast |  |  |
  | 126Select seq gb|NBP58287.1| | hypothetical protein [bacterium] | bacterium | NA | 1869227 | 124 | 124 | 97% | 1e-30 | 17.60% | 259 | NBP58287.1(scored below threshold on previous iteration) | Select seq NBP58287.1 for PSI blast |  |  |
  | 127Select seq gb|MBH45101.1| | hypothetical protein [Flavobacteriaceae bacterium] | Flavobacteriaceae bacterium | NA | 1871037 | 121 | 243 | 99% | 1e-29 | 19.60% | 255 | MBH45101.1(scored below threshold on previous iteration) | Select seq MBH45101.1 for PSI blast |  |  |
  | 128Select seq gb|RLD18005.1| | hypothetical protein DRI69\_11125 [Bacteroidetes bacterium] | Bacteroidetes bacterium | NA | 1898104 | 120 | 120 | 97% | 2e-29 | 19.18% | 241 | RLD18005.1(scored below threshold on previous iteration) | Select seq RLD18005.1 for PSI blast |  |  |
  | 129Select seq gb|MAJ82112.1| | hypothetical protein [Legionellales bacterium] | Legionellales bacterium | NA | 2026754 | 120 | 120 | 98% | 4e-29 | 22.80% | 254 | MAJ82112.1(scored below threshold on previous iteration) | Select seq MAJ82112.1 for PSI blast |  |  |
  | 130Select seq gb|OPZ48648.1| | DNA polymerase sliding clamp [Bacteroidetes bacterium ADurb.BinA104] | Bacteroidetes bacterium ADurb.BinA104 | NA | 1852816 | 119 | 119 | 98% | 7e-29 | 16.40% | 248 | OPZ48648.1(scored below threshold on previous iteration) | Select seq OPZ48648.1 for PSI blast |  |  |
  | 131Select seq gb|NDG33583.1| | hypothetical protein [bacterium] | bacterium | NA | 1869227 | 116 | 116 | 54% | 1e-28 | 24.82% | 177 | NDG33583.1(scored below threshold on previous iteration) | Select seq NDG33583.1 for PSI blast |  |  |
  | 132Select seq gb|RYZ28222.1| | proliferating cell nuclear antigen (pcna) [Propionibacteriaceae bacterium] | Propionibacteriaceae bacterium | NA | 2021380 | 115 | 115 | 71% | 1e-27 | 17.02% | 235 | RYZ28222.1(scored below threshold on previous iteration) | Select seq RYZ28222.1 for PSI blast |  |  |
  | 133Select seq gb|MBI96709.1| | hypothetical protein [bacterium] | bacterium | NA | 1869227 | 117 | 117 | 96% | 1e-27 | 16.35% | 289 | MBI96709.1(scored below threshold on previous iteration) | Select seq MBI96709.1 for PSI blast |  |  |
  | 134Select seq gb|RKY57622.1| | DNA polymerase sliding clamp [Candidatus Latescibacteria bacterium] | Candidatus Latescibacteria bacterium | NA | 2053570 | 112 | 225 | 59% | 2e-27 | 34.46% | 148 | RKY57622.1(scored below threshold on previous iteration) | Select seq RKY57622.1 for PSI blast |  |  |
  | 135Select seq gb|OEU73910.1| | hypothetical protein BA864\_05090 [Desulfuromonadales bacterium C00003093] | Desulfuromonadales bacterium C00003093 | NA | 1869308 | 113 | 113 | 96% | 9e-27 | 19.26% | 244 | OEU73910.1(scored below threshold on previous iteration) | Select seq OEU73910.1 for PSI blast |  |  |
  | 136Select seq gb|RLC69169.1| | hypothetical protein DRH97\_00615 [Chloroflexi bacterium] | Chloroflexi bacterium | NA | 2026724 | 110 | 110 | 96% | 1e-25 | 16.18% | 239 | RLC69169.1(scored below threshold on previous iteration) | Select seq RLC69169.1 for PSI blast |  |  |
  | 137Select seq gb|NBS68524.1| | hypothetical protein [bacterium] | bacterium | NA | 1869227 | 103 | 103 | 43% | 3e-24 | 25.93% | 112 | NBS68524.1(scored below threshold on previous iteration) | Select seq NBS68524.1 for PSI blast |  |  |
  | 138Select seq gb|MBC7332724.1| | DNA polymerase sliding clamp [Synergistetes bacterium] | Synergistetes bacterium | NA | 2268202 | 104 | 104 | 65% | 3e-24 | 33.74% | 159 | MBC7332724.1(scored below threshold on previous iteration) | Select seq MBC7332724.1 for PSI blast |  |  |
  | 139Select seq gb|MBF6555506.1| | hypothetical protein [Acidimicrobiales bacterium] | Acidimicrobiales bacterium | NA | 2201156 | 109 | 109 | 96% | 9e-24 | 22.90% | 430 | MBF6555506.1(scored below threshold on previous iteration) | Select seq MBF6555506.1 for PSI blast |  |  |
  | 140Select seq gb|RLC75119.1| | hypothetical protein DRI61\_15465 [Chloroflexi bacterium] | Chloroflexi bacterium | NA | 2026724 | 106 | 106 | 90% | 1e-23 | 22.22% | 301 | RLC75119.1(scored below threshold on previous iteration) | Select seq RLC75119.1 for PSI blast |  |  |
  | 141Select seq ref|WP\_067050733.1| | hypothetical protein [Moritella sp. JT01] | Moritella sp. JT01 | NA | 756698 | 103 | 103 | 90% | 6e-23 | 16.52% | 257 | WP\_067050733.1(scored below threshold on previous iteration) | Select seq WP\_067050733.1 for PSI blast |  |  |
  | 142Select seq gb|NBX48675.1| | hypothetical protein [bacterium] | bacterium | NA | 1869227 | 100 | 100 | 94% | 7e-22 | 16.60% | 248 | NBX48675.1(scored below threshold on previous iteration) | Select seq NBX48675.1 for PSI blast |  |  |
  | 143Select seq gb|MAD25962.1| | hypothetical protein [Verrucomicrobiales bacterium] | Verrucomicrobiales bacterium | NA | 2026801 | 100 | 100 | 99% | 2e-21 | 16.79% | 304 | MAD25962.1(scored below threshold on previous iteration) | Select seq MAD25962.1 for PSI blast |  |  |
  | 144Select seq tpg|HAI41630.1| | TPA: hypothetical protein [Maribacter sp.] | Maribacter sp. | NA | 1897614 | 98.6 | 98.6 | 95% | 7e-21 | 19.12% | 254 | HAI41630.1(scored below threshold on previous iteration) | Select seq HAI41630.1 for PSI blast |  |  |
  | 145Select seq gb|MAU35866.1| | hypothetical protein [Flavobacteriales bacterium] | Flavobacteriales bacterium | NA | 2021391 | 95.9 | 95.9 | 68% | 1e-20 | 21.23% | 179 | MAU35866.1(scored below threshold on previous iteration) | Select seq MAU35866.1 for PSI blast |  |  |
  | 146Select seq tpg|HER14123.1| | TPA: hypothetical protein [Fischerella sp.] | Fischerella sp. | NA | 1191 | 91.2 | 91.2 | 58% | 3e-19 | 31.51% | 143 | HER14123.1(scored below threshold on previous iteration) | Select seq HER14123.1 for PSI blast |  |  |
  | 147Select seq gb|MAF41652.1| | hypothetical protein [Cyanobium sp. ARS6] | Cyanobium sp. ARS6 | NA | 2024869 | 92.8 | 92.8 | 87% | 7e-19 | 16.14% | 232 | MAF41652.1(scored below threshold on previous iteration) | Select seq MAF41652.1 for PSI blast |  |  |
  | 148Select seq gb|PCJ29226.1| | hypothetical protein COA94\_02085 [Rickettsiales bacterium] | Rickettsiales bacterium | NA | 2026788 | 93.6 | 93.6 | 97% | 8e-19 | 18.29% | 269 | PCJ29226.1(scored below threshold on previous iteration) | Select seq PCJ29226.1 for PSI blast |  |  |
  | 149Select seq gb|NCA21076.1| | hypothetical protein [Crocinitomicaceae bacterium] | Crocinitomicaceae bacterium | NA | 2026728 | 89.3 | 89.3 | 44% | 9e-19 | 12.39% | 118 | NCA21076.1(scored below threshold on previous iteration) | Select seq NCA21076.1 for PSI blast |  |  |
  | 150Select seq tpg|HIP92434.1| | TPA: DNA polymerase sliding clamp [Thermotoga sp.] | Thermotoga sp. | NA | 28240 | 89.3 | 89.3 | 57% | 2e-18 | 27.97% | 145 | HIP92434.1(scored below threshold on previous iteration) | Select seq HIP92434.1 for PSI blast |  |  |
  | 151Select seq ref|WP\_015211709.1| | DNA polymerase III subunit beta [Oscillatoria nigro-viridis] | Oscillatoria nigro-viridis | NA | 482564 | 95.1 | 95.1 | 95% | 2e-18 | 17.86% | 478 | WP\_015211709.1(scored below threshold on previous iteration) | Select seq WP\_015211709.1 for PSI blast |  |  |
  | 152Select seq tpg|HEP40149.1| | TPA: DNA polymerase III subunit beta [Blastocatellia bacterium] | Blastocatellia bacterium | NA | 2052146 | 93.9 | 93.9 | 94% | 2e-18 | 18.33% | 366 | HEP40149.1(scored below threshold on previous iteration) | Select seq HEP40149.1 for PSI blast |  |  |
  | 153Select seq tpg|HDT11972.1| | TPA: hypothetical protein [bacterium] | bacterium | NA | 1869227 | 87.0 | 87.0 | 40% | 4e-18 | 36.63% | 101 | HDT11972.1(scored below threshold on previous iteration) | Select seq HDT11972.1 for PSI blast |  |  |
  | 154Select seq gb|MBL97255.1| | hypothetical protein [Legionellales bacterium] | Legionellales bacterium | NA | 2026754 | 92.0 | 92.0 | 100% | 4e-18 | 13.33% | 304 | MBL97255.1(scored below threshold on previous iteration) | Select seq MBL97255.1 for PSI blast |  |  |
  | 155Select seq gb|OCR01436.1| | DNA polymerase III subunit beta [Oscillatoriales cyanobacterium USR001] | Oscillatoriales cyanobacterium USR001 | NA | 1880991 | 93.2 | 93.2 | 95% | 7e-18 | 18.65% | 473 | OCR01436.1(scored below threshold on previous iteration) | Select seq OCR01436.1 for PSI blast |  |  |
  | 156Select seq ref|WP\_017303270.1| | DNA polymerase III subunit beta [Spirulina subsalsa] | Spirulina subsalsa | NA | 54311 | 92.0 | 184 | 95% | 1e-17 | 16.87% | 387 | WP\_017303270.1(scored below threshold on previous iteration) | Select seq WP\_017303270.1 for PSI blast |  |  |
  | 157Select seq tpg|HIK66788.1| | TPA: hypothetical protein [Flavobacteriales bacterium] | Flavobacteriales bacterium | NA | 2021391 | 88.2 | 88.2 | 98% | 5e-17 | 17.13% | 244 | HIK66788.1(scored below threshold on previous iteration) | Select seq HIK66788.1 for PSI blast |  |  |
  | 158Select seq gb|NBY39677.1| | hypothetical protein [Flavobacteriia bacterium] | Flavobacteriia bacterium | NA | 2044941 | 83.9 | 83.9 | 30% | 5e-17 | 17.95% | 94 | NBY39677.1(scored below threshold on previous iteration) | Select seq NBY39677.1 for PSI blast |  |  |
  | 159Select seq gb|MBD0306648.1| | DNA polymerase III subunit beta [Microcoleus sp. T1-bin1] | Microcoleus sp. T1-bin1 | NA | 2769071 | 90.5 | 90.5 | 95% | 6e-17 | 17.46% | 478 | MBD0306648.1(scored below threshold on previous iteration) | Select seq MBD0306648.1 for PSI blast |  |  |
  | 160Select seq ref|WP\_019488131.1| | DNA polymerase III subunit beta [Kamptonema formosum] | Kamptonema formosum | NA | 331992 | 90.5 | 90.5 | 95% | 7e-17 | 16.67% | 473 | WP\_019488131.1(scored below threshold on previous iteration) | Select seq WP\_019488131.1 for PSI blast |  |  |
  | 161Select seq gb|TAE70583.1| | hypothetical protein EAZ85\_11740 [Bacteroidetes bacterium] | Bacteroidetes bacterium | NA | 1898104 | 83.2 | 83.2 | 37% | 1e-16 | 25.53% | 103 | TAE70583.1(scored below threshold on previous iteration) | Select seq TAE70583.1 for PSI blast |  |  |
  | 162Select seq gb|NBS88271.1| | hypothetical protein [Verrucomicrobia bacterium] | Verrucomicrobia bacterium | NA | 2026799 | 86.2 | 86.2 | 36% | 1e-16 | 20.88% | 214 | NBS88271.1(scored below threshold on previous iteration) | Select seq NBS88271.1 for PSI blast |  |  |
  | 163Select seq gb|NDB84380.1| | hypothetical protein [Alphaproteobacteria bacterium] | Alphaproteobacteria bacterium | NA | 1913988 | 82.8 | 82.8 | 32% | 1e-16 | 19.28% | 96 | NDB84380.1(scored below threshold on previous iteration) | Select seq NDB84380.1 for PSI blast |  |  |
  | 164Select seq ref|WP\_094674721.1| | DNA polymerase III subunit beta [Hydrocoleum sp. CS-953] | Hydrocoleum sp. CS-953 | NA | 1671698 | 87.8 | 87.8 | 95% | 3e-16 | 17.39% | 353 | WP\_094674721.1(scored below threshold on previous iteration) | Select seq WP\_094674721.1 for PSI blast |  |  |
  | 165Select seq tpg|HBI72324.1| | TPA: hypothetical protein [Lachnospiraceae bacterium] | Lachnospiraceae bacterium | NA | 1898203 | 88.9 | 88.9 | 97% | 3e-16 | 16.67% | 584 | HBI72324.1(scored below threshold on previous iteration) | Select seq HBI72324.1 for PSI blast |  |  |
  | 166Select seq gb|RYF05887.1| | hypothetical protein EOO40\_09850 [Deltaproteobacteria bacterium] | Deltaproteobacteria bacterium | NA | 2026735 | 85.8 | 85.8 | 40% | 5e-16 | 15.84% | 272 | RYF05887.1(scored below threshold on previous iteration) | Select seq RYF05887.1 for PSI blast |  |  |
  | 167Select seq tpg|HID65709.1| | TPA: hypothetical protein [Aquificaceae bacterium] | Aquificaceae bacterium | NA | 2053503 | 82.4 | 82.4 | 45% | 5e-16 | 37.17% | 132 | HID65709.1(scored below threshold on previous iteration) | Select seq HID65709.1 for PSI blast |  |  |
  | 168Select seq gb|NEQ38316.1| | DNA polymerase III subunit beta [Okeania sp. SIO3I5] | Okeania sp. SIO3I5 | NA | 2607805 | 87.0 | 87.0 | 95% | 7e-16 | 17.00% | 387 | NEQ38316.1(scored below threshold on previous iteration) | Select seq NEQ38316.1 for PSI blast |  |  |
  | 169Select seq gb|NBS67530.1| | hypothetical protein [bacterium] | bacterium | NA | 1869227 | 80.8 | 80.8 | 40% | 9e-16 | 18.27% | 98 | NBS67530.1(scored below threshold on previous iteration) | Select seq NBS67530.1 for PSI blast |  |  |
  | 170Select seq gb|MAY90458.1| | hypothetical protein [Rickettsiales bacterium] | Rickettsiales bacterium | NA | 2026788 | 84.7 | 84.7 | 98% | 1e-15 | 17.53% | 244 | MAY90458.1(scored below threshold on previous iteration) | Select seq MAY90458.1 for PSI blast |  |  |
  | 171Select seq gb|MBP98812.1| | hypothetical protein [Candidatus Poribacteria bacterium] | Candidatus Poribacteria bacterium | NA | 2026781 | 84.3 | 84.3 | 98% | 1e-15 | 17.13% | 244 | MBP98812.1(scored below threshold on previous iteration) | Select seq MBP98812.1 for PSI blast |  |  |
  | 172Select seq gb|MAU37716.1| | hypothetical protein [Flavobacteriales bacterium] | Flavobacteriales bacterium | NA | 2021391 | 80.8 | 80.8 | 44% | 1e-15 | 20.72% | 111 | MAU37716.1(scored below threshold on previous iteration) | Select seq MAU37716.1 for PSI blast |  |  |
  | 173Select seq gb|MBE3094252.1| | hypothetical protein [Actinobacteria bacterium] | Actinobacteria bacterium | NA | 1883427 | 83.9 | 83.9 | 97% | 2e-15 | 16.42% | 266 | MBE3094252.1(scored below threshold on previous iteration) | Select seq MBE3094252.1 for PSI blast |  |  |
  | 174Select seq ref|WP\_012594366.1| | DNA polymerase III subunit beta [Rippkaea orientalis] | Rippkaea orientalis | NA | 2546366 | 85.5 | 85.5 | 95% | 3e-15 | 16.08% | 393 | WP\_012594366.1(scored below threshold on previous iteration) | Select seq WP\_012594366.1 for PSI blast |  |  |
  | 175Select seq ref|WP\_084555156.1| | DNA polymerase III subunit beta [Phormidium ambiguum] | Phormidium ambiguum | NA | 71191 | 85.5 | 170 | 95% | 3e-15 | 17.32% | 469 | WP\_084555156.1(scored below threshold on previous iteration) | Select seq WP\_084555156.1 for PSI blast |  |  |
  | 176Select seq ref|WP\_015141803.1| | DNA polymerase III subunit beta [Pleurocapsa minor] | Pleurocapsa minor | NA | 54308 | 85.1 | 85.1 | 95% | 3e-15 | 14.23% | 383 | WP\_015141803.1(scored below threshold on previous iteration) | Select seq WP\_015141803.1 for PSI blast |  |  |
  | 177Select seq ref|WP\_096559157.1| | DNA polymerase III subunit beta [Nostoc sp. NIES-4103] | Nostoc sp. NIES-4103 | NA | 2005458 | 85.1 | 85.1 | 95% | 3e-15 | 15.85% | 386 | WP\_096559157.1(scored below threshold on previous iteration) | Select seq WP\_096559157.1 for PSI blast |  |  |
  | 178Select seq gb|TVQ53692.1| | DNA polymerase III subunit beta [Spirulina sp. DLM2.Bin59] | Spirulina sp. DLM2.Bin59 | NA | 2480215 | 84.7 | 84.7 | 95% | 4e-15 | 15.85% | 382 | TVQ53692.1(scored below threshold on previous iteration) | Select seq TVQ53692.1 for PSI blast |  |  |
  | 179Select seq gb|OKH36250.1| | DNA polymerase III subunit beta [Phormidium ambiguum IAM M-71] | Phormidium ambiguum IAM M-71 | NA | 454136 | 85.1 | 85.1 | 95% | 4e-15 | 17.32% | 408 | OKH36250.1(scored below threshold on previous iteration) | Select seq OKH36250.1 for PSI blast |  |  |
  | 180Select seq ref|WP\_073597891.1| | DNA polymerase III subunit beta [Hydrococcus rivularis] | Hydrococcus rivularis | NA | 1616834 | 84.3 | 84.3 | 95% | 6e-15 | 14.23% | 383 | WP\_073597891.1(scored below threshold on previous iteration) | Select seq WP\_073597891.1 for PSI blast |  |  |
  | 181Select seq ref|WP\_084555250.1| | DNA polymerase III subunit beta [Phormidium ambiguum] | Phormidium ambiguum | NA | 71191 | 84.7 | 84.7 | 95% | 6e-15 | 16.93% | 476 | WP\_084555250.1(scored below threshold on previous iteration) | Select seq WP\_084555250.1 for PSI blast |  |  |
  | 182Select seq dbj|GBF85719.1| | DNA polymerase III subunit beta [Aphanothece sacrum FPU3] | Aphanothece sacrum FPU3 | NA | 1920664 | 84.3 | 84.3 | 95% | 6e-15 | 16.02% | 402 | GBF85719.1(scored below threshold on previous iteration) | Select seq GBF85719.1 for PSI blast |  |  |
  | 183Select seq ref|WP\_124975755.1| | DNA polymerase III subunit beta [Aphanothece sacrum] | Aphanothece sacrum | NA | 1122 | 83.9 | 83.9 | 95% | 8e-15 | 16.02% | 394 | WP\_124975755.1(scored below threshold on previous iteration) | Select seq WP\_124975755.1 for PSI blast |  |  |
  | 184Select seq gb|OKH33175.1| | DNA polymerase III subunit beta [Phormidium ambiguum IAM M-71] | Phormidium ambiguum IAM M-71 | NA | 454136 | 84.3 | 84.3 | 95% | 8e-15 | 16.93% | 408 | OKH33175.1(scored below threshold on previous iteration) | Select seq OKH33175.1 for PSI blast |  |  |
  | 185Select seq ref|WP\_190827164.1| | DNA polymerase III subunit beta [Iningainema sp. BLCCT55] | Iningainema sp. BLCCT55 | NA | 2748662 | 84.3 | 84.3 | 96% | 8e-15 | 14.50% | 444 | WP\_190827164.1(scored below threshold on previous iteration) | Select seq WP\_190827164.1 for PSI blast |  |  |
  | 186Select seq gb|NEO28492.1| | DNA polymerase III subunit beta [Kamptonema sp. SIO4C4] | Kamptonema sp. SIO4C4 | NA | 2607773 | 83.5 | 83.5 | 95% | 1e-14 | 15.56% | 389 | NEO28492.1(scored below threshold on previous iteration) | Select seq NEO28492.1 for PSI blast |  |  |
  | 187Select seq gb|RPF82134.1| | hypothetical protein CBC65\_000905 [Rhodothermaceae bacterium TMED105] | Rhodothermaceae bacterium TMED105 | NA | 1986665 | 82.8 | 82.8 | 96% | 1e-14 | 13.68% | 307 | RPF82134.1(scored below threshold on previous iteration) | Select seq RPF82134.1 for PSI blast |  |  |
  | 188Select seq ref|WP\_198123548.1| | DNA polymerase III subunit beta [Nostoc sp. CENA67] | Nostoc sp. CENA67 | NA | 2653702 | 83.5 | 83.5 | 95% | 1e-14 | 15.45% | 386 | WP\_198123548.1(scored below threshold on previous iteration) | Select seq WP\_198123548.1 for PSI blast |  |  |
  | 189Select seq gb|RLA70173.1| | hypothetical protein DRG24\_07300 [Epsilonproteobacteria bacterium] | Epsilonproteobacteria bacterium | NA | 2026809 | 81.6 | 81.6 | 97% | 1e-14 | 16.67% | 264 | RLA70173.1(scored below threshold on previous iteration) | Select seq RLA70173.1 for PSI blast |  |  |
  | 190Select seq tpg|HHN51246.1| | TPA: DNA polymerase III subunit beta [Blastocatellia bacterium] | Blastocatellia bacterium | NA | 2052146 | 81.2 | 81.2 | 94% | 2e-14 | 15.42% | 240 | HHN51246.1(scored below threshold on previous iteration) | Select seq HHN51246.1 for PSI blast |  |  |
  | 191Select seq gb|NEP78436.1| | DNA polymerase III subunit beta [Okeania sp. SIO3B3] | Okeania sp. SIO3B3 | NA | 2607804 | 82.8 | 82.8 | 95% | 2e-14 | 17.00% | 387 | NEP78436.1(scored below threshold on previous iteration) | Select seq NEP78436.1 for PSI blast |  |  |
  | 192Select seq gb|NBO73008.1| | hypothetical protein [bacterium] | bacterium | NA | 1869227 | 82.0 | 82.0 | 92% | 2e-14 | 10.93% | 339 | NBO73008.1(scored below threshold on previous iteration) | Select seq NBO73008.1 for PSI blast |  |  |
  | 193Select seq tpg|HAZ48782.1| | TPA: DNA polymerase III subunit beta [Cyanobacteria bacterium UBA11371] | Cyanobacteria bacterium UBA11371 | NA | 2055770 | 82.8 | 82.8 | 95% | 3e-14 | 14.23% | 473 | HAZ48782.1(scored below threshold on previous iteration) | Select seq HAZ48782.1 for PSI blast |  |  |
  | 194Select seq gb|NER01579.1| | DNA polymerase III subunit beta [Okeania sp. SIO3C4] | Okeania sp. SIO3C4 | NA | 2607786 | 82.0 | 82.0 | 95% | 4e-14 | 17.00% | 387 | NER01579.1(scored below threshold on previous iteration) | Select seq NER01579.1 for PSI blast |  |  |
  | 195Select seq gb|OQA51571.1| | DNA polymerase sliding clamp [Bacteroidetes bacterium ADurb.Bin302] | Bacteroidetes bacterium ADurb.Bin302 | NA | 1852811 | 79.7 | 79.7 | 97% | 4e-14 | 18.47% | 234 | OQA51571.1(scored below threshold on previous iteration) | Select seq OQA51571.1 for PSI blast |  |  |
  | 196Select seq ref|WP\_190574496.1| | unnamed protein product |  |  | 0 | 82.4 | 82.4 | 95% | 5e-14 | 13.85% | 504 | WP\_190574496.1(scored below threshold on previous iteration) | Select seq WP\_190574496.1 for PSI blast |  |  |
  | 197Select seq ref|WP\_015201154.1| | DNA polymerase III subunit beta [Crinalium epipsammum] | Crinalium epipsammum | NA | 241425 | 81.6 | 81.6 | 95% | 5e-14 | 15.42% | 385 | WP\_015201154.1(scored below threshold on previous iteration) | Select seq WP\_015201154.1 for PSI blast |  |  |
  | 198Select seq gb|NEP07459.1| | DNA polymerase III subunit beta [Okeania sp. SIO4D6] | Okeania sp. SIO4D6 | NA | 2607801 | 80.1 | 80.1 | 95% | 5e-14 | 16.21% | 265 | NEP07459.1(scored below threshold on previous iteration) | Select seq NEP07459.1 for PSI blast |  |  |
  | 199Select seq ref|WP\_083580228.1| | DNA polymerase III subunit beta [Planktothrix tepida] | Planktothrix tepida | NA | 1678309 | 82.0 | 82.0 | 96% | 6e-14 | 15.23% | 464 | WP\_083580228.1(scored below threshold on previous iteration) | Select seq WP\_083580228.1 for PSI blast |  |  |
  | 200Select seq gb|KEI65254.1| | DnaN [Planktothrix agardhii NIVA-CYA 126/8] | Planktothrix agardhii NIVA-CYA 126/8 | NA | 388467 | 82.0 | 82.0 | 96% | 6e-14 | 14.51% | 467 | KEI65254.1(scored below threshold on previous iteration) | Select seq KEI65254.1 for PSI blast |  |  |
  | 201Select seq ref|WP\_052369750.1| | DNA polymerase III subunit beta [Planktothrix agardhii] | Planktothrix agardhii | NA | 1160 | 82.0 | 82.0 | 96% | 6e-14 | 14.51% | 464 | WP\_052369750.1(scored below threshold on previous iteration) | Select seq WP\_052369750.1 for PSI blast |  |  |
  | 202Select seq ref|WP\_179047252.1| | DNA polymerase III subunit beta [Nostoc sp. TCL26-01] | Nostoc sp. TCL26-01 | NA | 2576904 | 81.2 | 81.2 | 95% | 7e-14 | 13.36% | 384 | WP\_179047252.1(scored below threshold on previous iteration) | Select seq WP\_179047252.1 for PSI blast |  |  |
  | 203Select seq dbj|GGA10618.1| | DNA polymerase III subunit beta [Okeania sp. KiyG1] | Okeania sp. KiyG1 | NA | 2720165 | 81.2 | 81.2 | 95% | 7e-14 | 16.80% | 384 | GGA10618.1(scored below threshold on previous iteration) | Select seq GGA10618.1 for PSI blast |  |  |
  | 204Select seq gb|NBP67062.1| | hypothetical protein [Bacteroidetes bacterium] | Bacteroidetes bacterium | NA | 1898104 | 80.8 | 80.8 | 92% | 7e-14 | 10.93% | 358 | NBP67062.1(scored below threshold on previous iteration) | Select seq NBP67062.1 for PSI blast |  |  |
  | 205Select seq gb|NEN89807.1| | DNA polymerase III subunit beta [Okeania sp. SIO3H1] | Okeania sp. SIO3H1 | NA | 2607810 | 80.8 | 80.8 | 95% | 9e-14 | 16.60% | 387 | NEN89807.1(scored below threshold on previous iteration) | Select seq NEN89807.1 for PSI blast |  |  |
  | 206Select seq gb|NET46204.1| | DNA polymerase III subunit beta [Okeania sp. SIO2B3] | Okeania sp. SIO2B3 | NA | 2607784 | 80.8 | 80.8 | 95% | 9e-14 | 16.60% | 395 | NET46204.1(scored below threshold on previous iteration) | Select seq NET46204.1 for PSI blast |  |  |
  | 207Select seq tpg|HFM96554.1| | TPA: DNA polymerase III subunit beta [Oscillatoriales cyanobacterium SpSt-418] | Oscillatoriales cyanobacterium SpSt-418 | NA | 2282169 | 81.2 | 81.2 | 95% | 1e-13 | 13.85% | 499 | HFM96554.1(scored below threshold on previous iteration) | Select seq HFM96554.1 for PSI blast |  |  |
  | 208Select seq gb|NJK47872.1| | DNA polymerase III subunit beta [Candidatus Gracilibacteria bacterium] | Candidatus Gracilibacteria bacterium | NA | 2044595 | 80.8 | 80.8 | 95% | 1e-13 | 16.94% | 382 | NJK47872.1(scored below threshold on previous iteration) | Select seq NJK47872.1 for PSI blast |  |  |
  | 209Select seq ref|WP\_010996171.1| | MULTISPECIES: DNA polymerase III subunit beta [Nostocaceae] | Nostocaceae | NA | 1162 | 80.5 | 80.5 | 95% | 1e-13 | 13.47% | 387 | WP\_010996171.1(scored below threshold on previous iteration) | Select seq WP\_010996171.1 for PSI blast |  |  |
  | 210Select seq ref|WP\_073343936.1| | DNA polymerase III subunit beta [Caldanaerobius fijiensis] | Caldanaerobius fijiensis | NA | 456330 | 80.5 | 80.5 | 95% | 1e-13 | 15.70% | 369 | WP\_073343936.1(scored below threshold on previous iteration) | Select seq WP\_073343936.1 for PSI blast |  |  |
  | 211Select seq emb|VXD16794.1| | DNA polymerase III subunit beta [Planktothrix paucivesiculata PCC 9631] | Planktothrix paucivesiculata PCC 9631 | NA | 671071 | 80.8 | 80.8 | 96% | 1e-13 | 14.51% | 467 | VXD16794.1(scored below threshold on previous iteration) | Select seq VXD16794.1 for PSI blast |  |  |
  | 212Select seq ref|WP\_083616961.1| | DNA polymerase III subunit beta [Planktothrix paucivesiculata] | Planktothrix paucivesiculata | NA | 1678308 | 80.8 | 80.8 | 96% | 1e-13 | 14.51% | 464 | WP\_083616961.1(scored below threshold on previous iteration) | Select seq WP\_083616961.1 for PSI blast |  |  |
  | 213Select seq gb|NEQ77915.1| | DNA polymerase III subunit beta [Okeania sp. SIO2C9] | Okeania sp. SIO2C9 | NA | 2607791 | 80.5 | 80.5 | 95% | 1e-13 | 16.60% | 387 | NEQ77915.1(scored below threshold on previous iteration) | Select seq NEQ77915.1 for PSI blast |  |  |
  | 214Select seq gb|MBP98540.1| | hypothetical protein [Candidatus Poribacteria bacterium] | Candidatus Poribacteria bacterium | NA | 2026781 | 78.5 | 78.5 | 99% | 1e-13 | 13.49% | 242 | MBP98540.1(scored below threshold on previous iteration) | Select seq MBP98540.1 for PSI blast |  |  |
  | 215Select seq tpg|HAC62525.1| | TPA: DNA polymerase III subunit beta [Cyanothece sp. UBA12306] | Cyanothece sp. UBA12306 | NA | 2055757 | 80.5 | 80.5 | 95% | 1e-13 | 16.22% | 394 | HAC62525.1(scored below threshold on previous iteration) | Select seq HAC62525.1 for PSI blast |  |  |
  | 216Select seq ref|WP\_147509764.1| | proliferating cell nuclear antigen (pcna) [Acinetobacter baumannii] | Acinetobacter baumannii | NA | 470 | 74.3 | 74.3 | 27% | 2e-13 | 26.09% | 76 | WP\_147509764.1(scored below threshold on previous iteration) | Select seq WP\_147509764.1 for PSI blast |  |  |
  | 217Select seq ref|WP\_072619599.1| | DNA polymerase III subunit beta [Spirulina major] | Spirulina major | NA | 270636 | 80.5 | 80.5 | 95% | 2e-13 | 14.80% | 388 | WP\_072619599.1(scored below threshold on previous iteration) | Select seq WP\_072619599.1 for PSI blast |  |  |
  | 218Select seq ref|WP\_124145677.1| | DNA polymerase III subunit beta [Okeania hirsuta] | Okeania hirsuta | NA | 1458930 | 80.1 | 80.1 | 95% | 2e-13 | 16.21% | 387 | WP\_124145677.1(scored below threshold on previous iteration) | Select seq WP\_124145677.1 for PSI blast |  |  |
  | 219Select seq gb|NEP73946.1| | DNA polymerase III subunit beta [Okeania sp. SIO2G5] | Okeania sp. SIO2G5 | NA | 2607796 | 80.1 | 80.1 | 95% | 2e-13 | 16.21% | 387 | NEP73946.1(scored below threshold on previous iteration) | Select seq NEP73946.1 for PSI blast |  |  |
  | 220Select seq dbj|GGA48654.1| | hypothetical protein CYANOKiyG1\_67730 [Okeania sp. KiyG1] | Okeania sp. KiyG1 | NA | 2720165 | 78.5 | 78.5 | 93% | 2e-13 | 17.00% | 240 | GGA48654.1(scored below threshold on previous iteration) | Select seq GGA48654.1 for PSI blast |  |  |
  | 221Select seq gb|NEP91145.1| | DNA polymerase III subunit beta [Okeania sp. SIO2C2] | Okeania sp. SIO2C2 | NA | 2607787 | 80.1 | 80.1 | 95% | 2e-13 | 16.27% | 386 | NEP91145.1(scored below threshold on previous iteration) | Select seq NEP91145.1 for PSI blast |  |  |
  | 222Select seq ref|WP\_011316901.1| | MULTISPECIES: DNA polymerase III subunit beta [Nostocaceae] | Nostocaceae | NA | 1162 | 80.1 | 80.1 | 95% | 2e-13 | 13.47% | 387 | WP\_011316901.1(scored below threshold on previous iteration) | Select seq WP\_011316901.1 for PSI blast |  |  |
  | 223Select seq ref|WP\_190570558.1| | DNA polymerase III subunit beta [Nostoc parmelioides] | Nostoc parmelioides | NA | 1521621 | 80.1 | 80.1 | 95% | 2e-13 | 13.47% | 387 | WP\_190570558.1(scored below threshold on previous iteration) | Select seq WP\_190570558.1 for PSI blast |  |  |
  | 224Select seq gb|NEO53538.1| | DNA polymerase III subunit beta [Okeania sp. SIO3B5] | Okeania sp. SIO3B5 | NA | 2607811 | 80.1 | 80.1 | 95% | 2e-13 | 16.21% | 387 | NEO53538.1(scored below threshold on previous iteration) | Select seq NEO53538.1 for PSI blast |  |  |
  | 225Select seq ref|WP\_038084084.1| | DNA polymerase III subunit beta [Tolypothrix bouteillei] | Tolypothrix bouteillei | NA | 1246981 | 80.1 | 80.1 | 95% | 2e-13 | 14.29% | 388 | WP\_038084084.1(scored below threshold on previous iteration) | Select seq WP\_038084084.1 for PSI blast |  |  |
  | 226Select seq ref|WP\_075891106.1| | DNA polymerase III subunit beta [Limnothrix rosea] | Limnothrix rosea | NA | 71188 | 80.1 | 80.1 | 93% | 2e-13 | 16.67% | 391 | WP\_075891106.1(scored below threshold on previous iteration) | Select seq WP\_075891106.1 for PSI blast |  |  |
  | 227Select seq ref|WP\_017653134.1| | DNA polymerase III subunit beta [Fortiea contorta] | Fortiea contorta | NA | 1892405 | 79.7 | 79.7 | 95% | 2e-13 | 13.88% | 383 | WP\_017653134.1(scored below threshold on previous iteration) | Select seq WP\_017653134.1 for PSI blast |  |  |
  | 228Select seq ref|WP\_106458545.1| | DNA polymerase III subunit beta [Aphanothece hegewaldii] | Aphanothece hegewaldii | NA | 1521625 | 79.7 | 79.7 | 95% | 3e-13 | 16.67% | 384 | WP\_106458545.1(scored below threshold on previous iteration) | Select seq WP\_106458545.1 for PSI blast |  |  |
  | 229Select seq ref|WP\_015136219.1| | DNA polymerase III subunit beta [Leptolyngbya sp. PCC 7376] | Leptolyngbya sp. PCC 7376 | NA | 111781 | 79.3 | 79.3 | 95% | 3e-13 | 15.48% | 392 | WP\_015136219.1(scored below threshold on previous iteration) | Select seq WP\_015136219.1 for PSI blast |  |  |
  | 230Select seq gb|NEO86948.1| | DNA polymerase III subunit beta [Spirulina sp. SIO3F2] | Spirulina sp. SIO3F2 | NA | 2607816 | 79.3 | 79.3 | 95% | 4e-13 | 18.40% | 387 | NEO86948.1(scored below threshold on previous iteration) | Select seq NEO86948.1 for PSI blast |  |  |
  | 231Select seq ref|WP\_193887624.1| | DNA polymerase III subunit beta [Fortiea sp. LEGE XX443] | Fortiea sp. LEGE XX443 | NA | 1828611 | 79.3 | 79.3 | 95% | 4e-13 | 16.19% | 384 | WP\_193887624.1(scored below threshold on previous iteration) | Select seq WP\_193887624.1 for PSI blast |  |  |
  | 232Select seq ref|WP\_029630377.1| | MULTISPECIES: DNA polymerase III subunit beta [Nostocales] | Nostocales | NA | 1161 | 78.9 | 78.9 | 96% | 5e-13 | 15.65% | 410 | WP\_029630377.1(scored below threshold on previous iteration) | Select seq WP\_029630377.1 for PSI blast |  |  |
  | 233Select seq ref|WP\_190485124.1| | DNA polymerase III subunit beta [Microcoleus sp. FACHB-831] | Microcoleus sp. FACHB-831 | NA | 2692827 | 78.9 | 78.9 | 95% | 5e-13 | 15.32% | 382 | WP\_190485124.1(scored below threshold on previous iteration) | Select seq WP\_190485124.1 for PSI blast |  |  |
  | 234Select seq emb|SKB13699.1| | DNA polymerase III subunit beta [Planktothrix sp. PCC 11201] | Planktothrix sp. PCC 11201 | NA | 1729650 | 79.3 | 79.3 | 96% | 5e-13 | 15.62% | 467 | SKB13699.1(scored below threshold on previous iteration) | Select seq SKB13699.1 for PSI blast |  |  |
  | 235Select seq ref|WP\_079680107.1| | DNA polymerase III subunit beta [Planktothrix sp. PCC 11201] | Planktothrix sp. PCC 11201 | NA | 1729650 | 79.3 | 79.3 | 96% | 5e-13 | 15.62% | 464 | WP\_079680107.1(scored below threshold on previous iteration) | Select seq WP\_079680107.1 for PSI blast |  |  |
  | 236Select seq gb|NCU42455.1| | hypothetical protein [Candidatus Moranbacteria bacterium] | Candidatus Moranbacteria bacterium | NA | 2045217 | 77.0 | 77.0 | 98% | 5e-13 | 19.29% | 239 | NCU42455.1(scored below threshold on previous iteration) | Select seq NCU42455.1 for PSI blast |  |  |
  | 237Select seq tpg|HAG85377.1| | TPA: DNA polymerase III subunit beta [Cyanobacteria bacterium UBA12227] | Cyanobacteria bacterium UBA12227 | NA | 2055765 | 78.5 | 78.5 | 98% | 7e-13 | 15.91% | 398 | HAG85377.1(scored below threshold on previous iteration) | Select seq HAG85377.1 for PSI blast |  |  |
  | 238Select seq tpg|HDU08060.1| | TPA: DNA polymerase III subunit beta [Blastocatellia bacterium] | Blastocatellia bacterium | NA | 2052146 | 78.1 | 78.1 | 94% | 7e-13 | 15.42% | 364 | HDU08060.1(scored below threshold on previous iteration) | Select seq HDU08060.1 for PSI blast |  |  |
  | 239Select seq ref|WP\_190468836.1| | unnamed protein product |  |  | 0 | 78.1 | 78.1 | 95% | 9e-13 | 13.88% | 386 | WP\_190468836.1(scored below threshold on previous iteration) | Select seq WP\_190468836.1 for PSI blast |  |  |
  | 240Select seq ref|WP\_010314157.1| | DNA polymerase III subunit beta [Synechococcus sp. CB0205] | Synechococcus sp. CB0205 | NA | 232363 | 78.1 | 78.1 | 96% | 9e-13 | 17.00% | 376 | WP\_010314157.1(scored below threshold on previous iteration) | Select seq WP\_010314157.1 for PSI blast |  |  |
  | 241Select seq ref|WP\_190406000.1| | DNA polymerase III subunit beta [Anabaena subtropica] | Anabaena subtropica | NA | 425380 | 78.1 | 78.1 | 95% | 9e-13 | 14.57% | 388 | WP\_190406000.1(scored below threshold on previous iteration) | Select seq WP\_190406000.1 for PSI blast |  |  |
  | 242Select seq ref|WP\_107671185.1| | DNA polymerase III subunit beta [Cyanothece sp. BG0011] | Cyanothece sp. BG0011 | NA | 2082950 | 78.1 | 78.1 | 95% | 9e-13 | 16.60% | 393 | WP\_107671185.1(scored below threshold on previous iteration) | Select seq WP\_107671185.1 for PSI blast |  |  |
  | 243Select seq ref|WP\_089130772.1| | DNA polymerase III subunit beta [Tolypothrix sp. NIES-4075] | Tolypothrix sp. NIES-4075 | NA | 2005459 | 78.1 | 78.1 | 95% | 9e-13 | 15.44% | 442 | WP\_089130772.1(scored below threshold on previous iteration) | Select seq WP\_089130772.1 for PSI blast |  |  |
  | 244Select seq gb|QFZ91518.1| | DNA polymerase III subunit beta [Synechococcus elongatus PCC 11802] | Synechococcus elongatus PCC 11802 | NA | 2283154 | 77.8 | 77.8 | 95% | 1e-12 | 16.12% | 390 | QFZ91518.1(scored below threshold on previous iteration) | Select seq QFZ91518.1 for PSI blast |  |  |
  | 245Select seq gb|NDB86628.1| | hypothetical protein [Alphaproteobacteria bacterium] | Alphaproteobacteria bacterium | NA | 1913988 | 72.4 | 72.4 | 27% | 1e-12 | 26.47% | 86 | NDB86628.1(scored below threshold on previous iteration) | Select seq NDB86628.1 for PSI blast |  |  |
  | 246Select seq ref|WP\_190969873.1| | MULTISPECIES: DNA polymerase III subunit beta [Nostoc] | Nostoc | NA | 1177 | 77.8 | 77.8 | 95% | 1e-12 | 15.16% | 383 | WP\_190969873.1(scored below threshold on previous iteration) | Select seq WP\_190969873.1 for PSI blast |  |  |
  | 247Select seq gb|OWY66685.1| | DNA polymerase III subunit beta [cyanobacterium TDX16] | cyanobacterium TDX16 | NA | 1503470 | 77.8 | 77.8 | 95% | 1e-12 | 17.48% | 381 | OWY66685.1(scored below threshold on previous iteration) | Select seq OWY66685.1 for PSI blast |  |  |
  | 248Select seq ref|WP\_190619044.1| | DNA polymerase III subunit beta [Calothrix membranacea] | Calothrix membranacea | NA | 212352 | 77.8 | 77.8 | 95% | 1e-12 | 14.57% | 385 | WP\_190619044.1(scored below threshold on previous iteration) | Select seq WP\_190619044.1 for PSI blast |  |  |
  | 249Select seq ref|WP\_193898320.1| | DNA polymerase III subunit beta [Nostoc sp. LEGE 06077] | Nostoc sp. LEGE 06077 | NA | 915325 | 77.4 | 77.4 | 95% | 1e-12 | 15.04% | 384 | WP\_193898320.1(scored below threshold on previous iteration) | Select seq WP\_193898320.1 for PSI blast |  |  |
  | 250Select seq ref|WP\_155746387.1| | DNA polymerase III subunit beta [Scytonema sp. UIC 10036] | Scytonema sp. UIC 10036 | NA | 2304196 | 77.4 | 77.4 | 95% | 1e-12 | 13.88% | 388 | WP\_155746387.1(scored below threshold on previous iteration) | Select seq WP\_155746387.1 for PSI blast |  |  |
  | 251Select seq ref|WP\_167725777.1| | DNA polymerase III subunit beta [Tolypothrix sp. PCC 7910] | Tolypothrix sp. PCC 7910 | NA | 2099387 | 77.4 | 77.4 | 95% | 2e-12 | 14.57% | 385 | WP\_167725777.1(scored below threshold on previous iteration) | Select seq WP\_167725777.1 for PSI blast |  |  |
  | 252Select seq ref|WP\_151536503.1| | DNA polymerase III subunit beta [Cytobacillus depressus] | Cytobacillus depressus | NA | 1602942 | 77.0 | 77.0 | 95% | 2e-12 | 18.83% | 378 | WP\_151536503.1(scored below threshold on previous iteration) | Select seq WP\_151536503.1 for PSI blast |  |  |
  | 253Select seq ref|WP\_096575924.1| | MULTISPECIES: DNA polymerase III subunit beta [Nostocales] | Nostocales | NA | 1161 | 77.0 | 77.0 | 95% | 2e-12 | 14.98% | 383 | WP\_096575924.1(scored below threshold on previous iteration) | Select seq WP\_096575924.1 for PSI blast |  |  |
  | 254Select seq gb|NBS18941.1| | hypothetical protein [Flavobacteriia bacterium] | Flavobacteriia bacterium | NA | 2044941 | 72.4 | 72.4 | 46% | 2e-12 | 26.83% | 123 | NBS18941.1(scored below threshold on previous iteration) | Select seq NBS18941.1 for PSI blast |  |  |
  | 255Select seq ref|WP\_008275522.1| | DNA polymerase III subunit beta [Crocosphaera chwakensis] | Crocosphaera chwakensis | NA | 2546361 | 77.0 | 77.0 | 95% | 2e-12 | 17.39% | 393 | WP\_008275522.1(scored below threshold on previous iteration) | Select seq WP\_008275522.1 for PSI blast |  |  |
  | 256Select seq ref|WP\_190583646.1| | MULTISPECIES: DNA polymerase III subunit beta [Nostocales] | Nostocales | NA | 1161 | 77.0 | 77.0 | 95% | 2e-12 | 14.57% | 385 | WP\_190583646.1(scored below threshold on previous iteration) | Select seq WP\_190583646.1 for PSI blast |  |  |
  | 257Select seq ref|WP\_045869792.1| | MULTISPECIES: DNA polymerase III subunit beta [Nostocales] | Nostocales | NA | 1161 | 77.0 | 77.0 | 95% | 2e-12 | 14.98% | 383 | WP\_045869792.1(scored below threshold on previous iteration) | Select seq WP\_045869792.1 for PSI blast |  |  |
  | 258Select seq tpg|HBL13963.1| | TPA: DNA polymerase III subunit beta [Cyanobacteria bacterium UBA11162] | Cyanobacteria bacterium UBA11162 | NA | 2055781 | 77.0 | 77.0 | 98% | 3e-12 | 15.53% | 398 | HBL13963.1(scored below threshold on previous iteration) | Select seq HBL13963.1 for PSI blast |  |  |
  | 259Select seq ref|WP\_190695666.1| | MULTISPECIES: DNA polymerase III subunit beta [unclassified Nostoc] | unclassified Nostoc | NA | 2593658 | 76.6 | 76.6 | 95% | 3e-12 | 16.60% | 384 | WP\_190695666.1(scored below threshold on previous iteration) | Select seq WP\_190695666.1 for PSI blast |  |  |
  | 260Select seq gb|NJN72345.1| | DNA polymerase III subunit beta [Limnothrix sp. RL\_2\_0] | Limnothrix sp. RL\_2\_0 | NA | 2720421 | 76.6 | 153 | 93% | 3e-12 | 17.00% | 394 | NJN72345.1(scored below threshold on previous iteration) | Select seq NJN72345.1 for PSI blast |  |  |
  | 261Select seq ref|WP\_026134740.1| | DNA polymerase III subunit beta [Scytonema hofmannii] | Scytonema hofmannii | NA | 34078 | 76.2 | 76.2 | 95% | 4e-12 | 14.29% | 388 | WP\_026134740.1(scored below threshold on previous iteration) | Select seq WP\_026134740.1 for PSI blast |  |  |
  | 262Select seq ref|WP\_144479456.1| | DNA polymerase III subunit beta [Cytobacillus oceanisediminis] | Cytobacillus oceanisediminis | NA | 665099 | 76.2 | 76.2 | 95% | 4e-12 | 18.75% | 378 | WP\_144479456.1(scored below threshold on previous iteration) | Select seq WP\_144479456.1 for PSI blast |  |  |
  | 263Select seq gb|NJL80793.1| | DNA polymerase III subunit beta [Richelia sp. SM2\_1\_7] | Richelia sp. SM2\_1\_7 | NA | 2720465 | 76.2 | 76.2 | 95% | 4e-12 | 13.77% | 390 | NJL80793.1(scored below threshold on previous iteration) | Select seq NJL80793.1 for PSI blast |  |  |
  | 264Select seq gb|OCQ89407.1| | DNA polymerase III subunit beta [Nostoc sp. MBR 210] | Nostoc sp. MBR 210 | NA | 1881015 | 76.2 | 76.2 | 95% | 4e-12 | 15.10% | 384 | OCQ89407.1(scored below threshold on previous iteration) | Select seq OCQ89407.1 for PSI blast |  |  |
  | 265Select seq gb|MBF2013737.1| | DNA polymerase III subunit beta [Rivularia sp. T60\_A2020\_040] | Rivularia sp. T60\_A2020\_040 | NA | 2774335 | 76.2 | 76.2 | 95% | 4e-12 | 13.77% | 390 | MBF2013737.1(scored below threshold on previous iteration) | Select seq MBF2013737.1 for PSI blast |  |  |
  | 266Select seq tpg|HCF26097.1| | TPA: DNA polymerase III subunit beta [Cyanobacteria bacterium UBA11049] | Cyanobacteria bacterium UBA11049 | NA | 2055787 | 75.8 | 75.8 | 95% | 5e-12 | 13.88% | 379 | HCF26097.1(scored below threshold on previous iteration) | Select seq HCF26097.1 for PSI blast |  |  |
  | 267Select seq ref|WP\_104545376.1| | DNA polymerase III subunit beta [Chroococcidiopsis sp. TS-821] | Chroococcidiopsis sp. TS-821 | NA | 1378066 | 75.8 | 75.8 | 98% | 5e-12 | 15.69% | 379 | WP\_104545376.1(scored below threshold on previous iteration) | Select seq WP\_104545376.1 for PSI blast |  |  |
  | 268Select seq gb|NES99152.1| | DNA polymerase III subunit beta [Sphaerospermopsis sp. SIO1G1] | Sphaerospermopsis sp. SIO1G1 | NA | 2607814 | 75.8 | 75.8 | 95% | 5e-12 | 16.60% | 382 | NES99152.1(scored below threshold on previous iteration) | Select seq NES99152.1 for PSI blast |  |  |
  | 269Select seq gb|AZB71840.1| | DNA polymerase III subunit beta [Synechococcus elongatus PCC 11801] | Synechococcus elongatus PCC 11801 | NA | 2219813 | 75.8 | 75.8 | 95% | 5e-12 | 16.12% | 390 | AZB71840.1(scored below threshold on previous iteration) | Select seq AZB71840.1 for PSI blast |  |  |
  | 270Select seq ref|WP\_045056711.1| | DNA polymerase III subunit beta [Aliterella atlantica] | Aliterella atlantica | NA | 1827278 | 75.8 | 75.8 | 95% | 5e-12 | 16.87% | 376 | WP\_045056711.1(scored below threshold on previous iteration) | Select seq WP\_045056711.1 for PSI blast |  |  |
  | 271Select seq gb|NET70044.1| | DNA polymerase III subunit beta [Sphaerospermopsis sp. SIO1G2] | Sphaerospermopsis sp. SIO1G2 | NA | 2607815 | 75.8 | 75.8 | 95% | 6e-12 | 16.60% | 382 | NET70044.1(scored below threshold on previous iteration) | Select seq NET70044.1 for PSI blast |  |  |
  | 272Select seq ref|WP\_132947232.1| | DNA polymerase III subunit beta [Thermolongibacillus altinsuensis] | Thermolongibacillus altinsuensis | NA | 575256 | 75.4 | 75.4 | 95% | 6e-12 | 17.92% | 378 | WP\_132947232.1(scored below threshold on previous iteration) | Select seq WP\_132947232.1 for PSI blast |  |  |
  | 273Select seq tpg|HIK28708.1| | TPA: DNA polymerase III subunit beta [Oscillatoriaceae cyanobacterium M7585\_C2015\_266] | Oscillatoriaceae cyanobacterium M7585\_C2015\_266 | NA | 2774341 | 75.8 | 75.8 | 95% | 6e-12 | 16.27% | 395 | HIK28708.1(scored below threshold on previous iteration) | Select seq HIK28708.1 for PSI blast |  |  |
  | 274Select seq ref|WP\_066310701.1| | DNA polymerase III subunit beta [Bacillus sp. FJAT-29814] | Bacillus sp. FJAT-29814 | NA | 1729688 | 75.4 | 75.4 | 95% | 6e-12 | 16.32% | 378 | WP\_066310701.1(scored below threshold on previous iteration) | Select seq WP\_066310701.1 for PSI blast |  |  |
  | 275Select seq ref|WP\_009545676.1| | DNA polymerase III subunit beta [Crocosphaera subtropica] | Crocosphaera subtropica | NA | 2546360 | 75.4 | 75.4 | 95% | 7e-12 | 17.00% | 393 | WP\_009545676.1(scored below threshold on previous iteration) | Select seq WP\_009545676.1 for PSI blast |  |  |
  | 276Select seq ref|WP\_193918749.1| | DNA polymerase III subunit beta [Plectonema radiosum] | Plectonema radiosum | NA | 945768 | 75.4 | 75.4 | 95% | 7e-12 | 13.77% | 391 | WP\_193918749.1(scored below threshold on previous iteration) | Select seq WP\_193918749.1 for PSI blast |  |  |
  | 277Select seq ref|WP\_073551314.1| | DNA polymerase III subunit beta [Chroogloeocystis siderophila] | Chroogloeocystis siderophila | NA | 329163 | 75.4 | 75.4 | 98% | 7e-12 | 14.45% | 379 | WP\_073551314.1(scored below threshold on previous iteration) | Select seq WP\_073551314.1 for PSI blast |  |  |
  | 278Select seq ref|WP\_102176059.1| | DNA polymerase III subunit beta [Fischerella thermalis] | Fischerella thermalis | NA | 372787 | 75.4 | 75.4 | 98% | 7e-12 | 12.84% | 388 | WP\_102176059.1(scored below threshold on previous iteration) | Select seq WP\_102176059.1 for PSI blast |  |  |
  | 279Select seq ref|WP\_096582000.1| | DNA polymerase III subunit beta [Anabaenopsis circularis] | Anabaenopsis circularis | NA | 109266 | 75.4 | 75.4 | 95% | 8e-12 | 14.75% | 383 | WP\_096582000.1(scored below threshold on previous iteration) | Select seq WP\_096582000.1 for PSI blast |  |  |
  | 280Select seq ref|WP\_053433291.1| | DNA polymerase III subunit beta [Sporosarcina globispora] | Sporosarcina globispora | NA | 1459 | 75.1 | 75.1 | 95% | 9e-12 | 19.58% | 378 | WP\_053433291.1(scored below threshold on previous iteration) | Select seq WP\_053433291.1 for PSI blast |  |  |
  | 281Select seq ref|WP\_113885471.1| | MULTISPECIES: DNA polymerase III subunit beta [Cytobacillus] | Cytobacillus | NA | 2675230 | 75.1 | 75.1 | 95% | 9e-12 | 19.58% | 378 | WP\_113885471.1(scored below threshold on previous iteration) | Select seq WP\_113885471.1 for PSI blast |  |  |
  | 282Select seq ref|WP\_015188090.1| | DNA polymerase III subunit beta [Gloeocapsa sp. PCC 7428] | Gloeocapsa sp. PCC 7428 | NA | 1173026 | 75.1 | 75.1 | 98% | 9e-12 | 15.35% | 379 | WP\_015188090.1(scored below threshold on previous iteration) | Select seq WP\_015188090.1 for PSI blast |  |  |
  | 283Select seq ref|WP\_103123458.1| | DNA polymerase III subunit beta [Nostoc cycadae] | Nostoc cycadae | NA | 246795 | 75.1 | 75.1 | 95% | 9e-12 | 14.75% | 383 | WP\_103123458.1(scored below threshold on previous iteration) | Select seq WP\_103123458.1 for PSI blast |  |  |
  | 284Select seq ref|WP\_190831965.1| | DNA polymerase III subunit beta [Iningainema sp. BLCCT55] | Iningainema sp. BLCCT55 | NA | 2748662 | 75.1 | 75.1 | 95% | 1e-11 | 14.63% | 392 | WP\_190831965.1(scored below threshold on previous iteration) | Select seq WP\_190831965.1 for PSI blast |  |  |
  | 285Select seq gb|NRB06830.1| | DNA polymerase III subunit beta [Richelia sp.] | Richelia sp. | NA | 2053605 | 75.1 | 75.1 | 95% | 1e-11 | 16.39% | 387 | NRB06830.1(scored below threshold on previous iteration) | Select seq NRB06830.1 for PSI blast |  |  |
  | 286Select seq gb|KKQ92173.1| | polymerase III subunit beta protein [Candidatus Woesebacteria bacterium GW2011\_GWB1\_39\_10] | Candidatus Woesebacteria bacterium GW2011\_GWB1\_39\_10 | NA | 1618572 | 75.1 | 75.1 | 92% | 1e-11 | 18.64% | 374 | KKQ92173.1(scored below threshold on previous iteration) | Select seq KKQ92173.1 for PSI blast |  |  |
  | 287Select seq tpg|HER12275.1| | TPA: DNA polymerase III subunit beta [Fischerella sp.] | Fischerella sp. | NA | 1191 | 74.3 | 74.3 | 98% | 1e-11 | 12.84% | 300 | HER12275.1(scored below threshold on previous iteration) | Select seq HER12275.1 for PSI blast |  |  |
  | 288Select seq ref|WP\_015110936.1| | DNA polymerase III subunit beta [Nostoc sp. PCC 7107] | Nostoc sp. PCC 7107 | NA | 317936 | 75.1 | 75.1 | 95% | 1e-11 | 15.85% | 383 | WP\_015110936.1(scored below threshold on previous iteration) | Select seq WP\_015110936.1 for PSI blast |  |  |
  | 289Select seq ref|WP\_101650072.1| | DNA polymerase III subunit beta [Neobacillus cucumis] | Neobacillus cucumis | NA | 1740721 | 75.1 | 75.1 | 95% | 1e-11 | 16.74% | 378 | WP\_101650072.1(scored below threshold on previous iteration) | Select seq WP\_101650072.1 for PSI blast |  |  |
  | 290Select seq gb|NEP51899.1| | DNA polymerase III subunit beta [Moorea sp. SIO3C2] | Moorea sp. SIO3C2 | NA | 2607842 | 74.7 | 74.7 | 95% | 1e-11 | 15.75% | 393 | NEP51899.1(scored below threshold on previous iteration) | Select seq NEP51899.1 for PSI blast |  |  |
  | 291Select seq ref|WP\_061793265.1| | DNA polymerase III subunit beta [Cytobacillus firmus] | Cytobacillus firmus | NA | 1399 | 74.7 | 74.7 | 95% | 1e-11 | 19.58% | 378 | WP\_061793265.1(scored below threshold on previous iteration) | Select seq WP\_061793265.1 for PSI blast |  |  |
  | 292Select seq ref|WP\_066295990.1| | MULTISPECIES: DNA polymerase III subunit beta [Bacteria] | Bacteria | eubacteria | 2 | 74.7 | 74.7 | 95% | 1e-11 | 18.33% | 378 | WP\_066295990.1(scored below threshold on previous iteration) | Select seq WP\_066295990.1 for PSI blast |  |  |
  | 293Select seq ref|WP\_053478301.1| | MULTISPECIES: DNA polymerase III subunit beta [Bacillaceae] | Bacillaceae | NA | 186817 | 74.7 | 74.7 | 95% | 1e-11 | 18.41% | 378 | WP\_053478301.1(scored below threshold on previous iteration) | Select seq WP\_053478301.1 for PSI blast |  |  |
  | 294Select seq gb|NET56745.1| | DNA polymerase III subunit beta [Symploca sp. SIO2E6] | Symploca sp. SIO2E6 | NA | 2607809 | 74.7 | 74.7 | 95% | 1e-11 | 15.48% | 394 | NET56745.1(scored below threshold on previous iteration) | Select seq NET56745.1 for PSI blast |  |  |
  | 295Select seq ref|WP\_009332491.1| | MULTISPECIES: DNA polymerase III subunit beta [Bacillaceae] | Bacillaceae | NA | 186817 | 74.7 | 74.7 | 95% | 2e-11 | 18.75% | 378 | WP\_009332491.1(scored below threshold on previous iteration) | Select seq WP\_009332491.1 for PSI blast |  |  |
  | 296Select seq ref|WP\_102218973.1| | DNA polymerase III subunit beta [Fischerella thermalis] | Fischerella thermalis | NA | 372787 | 74.7 | 74.7 | 98% | 2e-11 | 12.84% | 388 | WP\_102218973.1(scored below threshold on previous iteration) | Select seq WP\_102218973.1 for PSI blast |  |  |
  | 297Select seq gb|OUU14720.1| | hypothetical protein CBB97\_24650 [Candidatus Endolissoclinum sp. TMED37] | Candidatus Endolissoclinum sp. TMED37 | NA | 1986638 | 70.8 | 70.8 | 55% | 2e-11 | 13.99% | 146 | OUU14720.1(scored below threshold on previous iteration) | Select seq OUU14720.1 for PSI blast |  |  |
  | 298Select seq ref|WP\_102273351.1| | DNA polymerase III subunit beta [Bacillus massiliogabonensis] | Bacillus massiliogabonensis | NA | 1871011 | 74.3 | 74.3 | 95% | 2e-11 | 18.41% | 378 | WP\_102273351.1(scored below threshold on previous iteration) | Select seq WP\_102273351.1 for PSI blast |  |  |
  | 299Select seq ref|WP\_096544253.1| | DNA polymerase III subunit beta [Raphidiopsis curvata] | Raphidiopsis curvata | NA | 312883 | 74.3 | 74.3 | 95% | 2e-11 | 15.57% | 382 | WP\_096544253.1(scored below threshold on previous iteration) | Select seq WP\_096544253.1 for PSI blast |  |  |
  | 300Select seq gb|NEO65636.1| | DNA polymerase III subunit beta [Moorea sp. SIO4G2] | Moorea sp. SIO4G2 | NA | 2607820 | 73.9 | 73.9 | 95% | 2e-11 | 15.75% | 310 | NEO65636.1(scored below threshold on previous iteration) | Select seq NEO65636.1 for PSI blast |  |  |
  | 301Select seq gb|NEO16607.1| | DNA polymerase III subunit beta [Moorea sp. SIO3E8] | Moorea sp. SIO3E8 | NA | 2607830 | 74.3 | 74.3 | 95% | 2e-11 | 15.75% | 393 | NEO16607.1(scored below threshold on previous iteration) | Select seq NEO16607.1 for PSI blast |  |  |
  | 302Select seq ref|WP\_159345479.1| | DNA polymerase III subunit beta [Cytobacillus firmus] | Cytobacillus firmus | NA | 1399 | 74.3 | 74.3 | 95% | 2e-11 | 19.58% | 378 | WP\_159345479.1(scored below threshold on previous iteration) | Select seq WP\_159345479.1 for PSI blast |  |  |
  | 303Select seq ref|WP\_079505943.1| | DNA polymerase III subunit beta [Mesobacillus jeotgali] | Mesobacillus jeotgali | NA | 129985 | 73.9 | 73.9 | 95% | 2e-11 | 18.33% | 378 | WP\_079505943.1(scored below threshold on previous iteration) | Select seq WP\_079505943.1 for PSI blast |  |  |
  | 304Select seq ref|WP\_159863634.1| | DNA polymerase III subunit beta [Bacillus sp. ZZV12-4809] | Bacillus sp. ZZV12-4809 | NA | 2575830 | 73.9 | 73.9 | 95% | 2e-11 | 19.58% | 378 | WP\_159863634.1(scored below threshold on previous iteration) | Select seq WP\_159863634.1 for PSI blast |  |  |
  | 305Select seq ref|WP\_096660216.1| | DNA polymerase III subunit beta [Calothrix parasitica] | Calothrix parasitica | NA | 1973486 | 73.9 | 73.9 | 94% | 2e-11 | 13.82% | 390 | WP\_096660216.1(scored below threshold on previous iteration) | Select seq WP\_096660216.1 for PSI blast |  |  |
  | 306Select seq ref|WP\_174751886.1| | DNA polymerase III subunit beta [Cytobacillus firmus] | Cytobacillus firmus | NA | 1399 | 73.9 | 73.9 | 95% | 2e-11 | 19.58% | 378 | WP\_174751886.1(scored below threshold on previous iteration) | Select seq WP\_174751886.1 for PSI blast |  |  |
  | 307Select seq ref|WP\_149868010.1| | DNA polymerase III subunit beta [Bacillus sp. S3] | Bacillus sp. S3 | NA | 486398 | 73.9 | 147 | 95% | 3e-11 | 15.90% | 378 | WP\_149868010.1(scored below threshold on previous iteration) | Select seq WP\_149868010.1 for PSI blast |  |  |
  | 308Select seq ref|WP\_057768018.1| | DNA polymerase III subunit beta [Cytobacillus praedii] | Cytobacillus praedii | NA | 1742358 | 73.9 | 73.9 | 95% | 3e-11 | 18.41% | 378 | WP\_057768018.1(scored below threshold on previous iteration) | Select seq WP\_057768018.1 for PSI blast |  |  |
  | 309Select seq ref|WP\_012597758.1| | DNA polymerase III subunit beta [Gloeothece citriformis] | Gloeothece citriformis | NA | 2546356 | 73.9 | 73.9 | 95% | 3e-11 | 16.41% | 394 | WP\_012597758.1(scored below threshold on previous iteration) | Select seq WP\_012597758.1 for PSI blast |  |  |
  | 310Select seq ref|WP\_048010199.1| | MULTISPECIES: DNA polymerase III subunit beta [Bacillaceae] | Bacillaceae | NA | 186817 | 73.5 | 73.5 | 95% | 3e-11 | 19.58% | 378 | WP\_048010199.1(scored below threshold on previous iteration) | Select seq WP\_048010199.1 for PSI blast |  |  |
  | 311Select seq ref|WP\_023613367.1| | MULTISPECIES: DNA polymerase III subunit beta [Bacillaceae] | Bacillaceae | NA | 186817 | 73.5 | 73.5 | 95% | 3e-11 | 17.92% | 378 | WP\_023613367.1(scored below threshold on previous iteration) | Select seq WP\_023613367.1 for PSI blast |  |  |
  | 312Select seq ref|WP\_076262910.1| | DNA polymerase III subunit beta [Paenibacillus sp. FSL R5-0490] | Paenibacillus sp. FSL R5-0490 | NA | 1920424 | 73.5 | 73.5 | 95% | 3e-11 | 19.17% | 378 | WP\_076262910.1(scored below threshold on previous iteration) | Select seq WP\_076262910.1 for PSI blast |  |  |
  | 313Select seq ref|WP\_043906388.1| | DNA polymerase III subunit beta [Parageobacillus genomosp. 1] | Parageobacillus genomosp. 1 | NA | 1295642 | 73.5 | 73.5 | 95% | 3e-11 | 18.67% | 378 | WP\_043906388.1(scored below threshold on previous iteration) | Select seq WP\_043906388.1 for PSI blast |  |  |
  | 314Select seq ref|WP\_173062385.1| | DNA polymerase III subunit beta [Bacillus sp. BRMEA1] | Bacillus sp. BRMEA1 | NA | 2738405 | 73.5 | 73.5 | 95% | 3e-11 | 17.28% | 378 | WP\_173062385.1(scored below threshold on previous iteration) | Select seq WP\_173062385.1 for PSI blast |  |  |
  | 315Select seq ref|WP\_035328331.1| | DNA polymerase III subunit beta [Cytobacillus firmus] | Cytobacillus firmus | NA | 1399 | 73.5 | 73.5 | 95% | 3e-11 | 19.58% | 378 | WP\_035328331.1(scored below threshold on previous iteration) | Select seq WP\_035328331.1 for PSI blast |  |  |
  | 316Select seq ref|WP\_026584872.1| | DNA polymerase III subunit beta [Bacillus sp. J33] | Bacillus sp. J33 | NA | 935836 | 73.1 | 73.1 | 95% | 4e-11 | 17.15% | 378 | WP\_026584872.1(scored below threshold on previous iteration) | Select seq WP\_026584872.1 for PSI blast |  |  |
  | 317Select seq gb|NEO43960.1| | DNA polymerase III subunit beta [Moorea sp. SIO4A3] | Moorea sp. SIO4A3 | NA | 2607836 | 73.5 | 73.5 | 95% | 4e-11 | 15.75% | 393 | NEO43960.1(scored below threshold on previous iteration) | Select seq NEO43960.1 for PSI blast |  |  |
  | 318Select seq gb|NJL62612.1| | DNA polymerase III subunit beta [Methylacidiphilales bacterium] | Methylacidiphilales bacterium | NA | 2720631 | 73.1 | 73.1 | 95% | 4e-11 | 13.47% | 386 | NJL62612.1(scored below threshold on previous iteration) | Select seq NJL62612.1 for PSI blast |  |  |
  | 319Select seq gb|TRV09811.1| | DNA polymerase III subunit beta [Microcystis wesenbergii Mw\_MB\_S\_20031200\_S109] | Microcystis wesenbergii Mw\_MB\_S\_20031200\_S109 | NA | 2486240 | 73.1 | 73.1 | 95% | 4e-11 | 14.34% | 382 | TRV09811.1(scored below threshold on previous iteration) | Select seq TRV09811.1 for PSI blast |  |  |
  | 320Select seq ref|WP\_144566182.1| | DNA polymerase III subunit beta [Neobacillus bataviensis] | Neobacillus bataviensis | NA | 220685 | 73.1 | 73.1 | 95% | 4e-11 | 16.81% | 378 | WP\_144566182.1(scored below threshold on previous iteration) | Select seq WP\_144566182.1 for PSI blast |  |  |
  | 321Select seq ref|WP\_166907456.1| | DNA polymerase III subunit beta [Saccharococcus thermophilus] | Saccharococcus thermophilus | NA | 29396 | 73.1 | 73.1 | 95% | 4e-11 | 18.67% | 378 | WP\_166907456.1(scored below threshold on previous iteration) | Select seq WP\_166907456.1 for PSI blast |  |  |
  | 322Select seq ref|WP\_110063570.1| | DNA polymerase III subunit beta [Cytobacillus oceanisediminis] | Cytobacillus oceanisediminis | NA | 665099 | 73.1 | 73.1 | 95% | 4e-11 | 17.57% | 378 | WP\_110063570.1(scored below threshold on previous iteration) | Select seq WP\_110063570.1 for PSI blast |  |  |
  | 323Select seq emb|CDN15341.1| | DNA polymerase III beta subunit [Richelia intracellularis] | Richelia intracellularis | NA | 1164990 | 73.5 | 73.5 | 95% | 4e-11 | 16.39% | 436 | CDN15341.1(scored below threshold on previous iteration) | Select seq CDN15341.1 for PSI blast |  |  |
  | 324Select seq gb|NEO72801.1| | DNA polymerase III subunit beta [Moorea sp. SIO3H5] | Moorea sp. SIO3H5 | NA | 2607834 | 73.1 | 73.1 | 95% | 5e-11 | 15.35% | 393 | NEO72801.1(scored below threshold on previous iteration) | Select seq NEO72801.1 for PSI blast |  |  |
  | 325Select seq ref|WP\_026573833.1| | DNA polymerase III subunit beta [Bacillus sp. UNC438CL73TsuS30] | Bacillus sp. UNC438CL73TsuS30 | NA | 1340434 | 73.1 | 73.1 | 95% | 5e-11 | 16.87% | 378 | WP\_026573833.1(scored below threshold on previous iteration) | Select seq WP\_026573833.1 for PSI blast |  |  |
  | 326Select seq ref|WP\_125482043.1| | DNA polymerase III subunit beta [Mesobacillus subterraneus] | Mesobacillus subterraneus | NA | 285983 | 73.1 | 73.1 | 95% | 5e-11 | 18.75% | 378 | WP\_125482043.1(scored below threshold on previous iteration) | Select seq WP\_125482043.1 for PSI blast |  |  |
  | 327Select seq ref|WP\_144544652.1| | DNA polymerase III subunit beta [Cytobacillus oceanisediminis] | Cytobacillus oceanisediminis | NA | 665099 | 73.1 | 73.1 | 95% | 5e-11 | 17.99% | 378 | WP\_144544652.1(scored below threshold on previous iteration) | Select seq WP\_144544652.1 for PSI blast |  |  |
  | 328Select seq gb|NET85241.1| | DNA polymerase III subunit beta [Moorea sp. SIO1F2] | Moorea sp. SIO1F2 | NA | 2607819 | 73.1 | 73.1 | 95% | 5e-11 | 16.14% | 393 | NET85241.1(scored below threshold on previous iteration) | Select seq NET85241.1 for PSI blast |  |  |
  | 329Select seq ref|WP\_038538167.1| | MULTISPECIES: DNA polymerase III subunit beta [unclassified Bacillus] | unclassified Bacillus | NA | 185979 | 73.1 | 73.1 | 95% | 5e-11 | 16.39% | 378 | WP\_038538167.1(scored below threshold on previous iteration) | Select seq WP\_038538167.1 for PSI blast |  |  |
  | 330Select seq ref|WP\_098530204.1| | MULTISPECIES: DNA polymerase III subunit beta [unclassified Bacillus] | unclassified Bacillus | NA | 185979 | 73.1 | 73.1 | 95% | 5e-11 | 16.39% | 378 | WP\_098530204.1(scored below threshold on previous iteration) | Select seq WP\_098530204.1 for PSI blast |  |  |
  | 331Select seq ref|WP\_163251973.1| | DNA polymerase III subunit beta [Neobacillus thermocopriae] | Neobacillus thermocopriae | NA | 1215031 | 73.1 | 73.1 | 95% | 5e-11 | 15.97% | 378 | WP\_163251973.1(scored below threshold on previous iteration) | Select seq WP\_163251973.1 for PSI blast |  |  |
  | 332Select seq ref|WP\_071104982.1| | DNA polymerase III subunit beta [Moorea producens] | Moorea producens | NA | 1155739 | 73.1 | 73.1 | 95% | 5e-11 | 16.14% | 393 | WP\_071104982.1(scored below threshold on previous iteration) | Select seq WP\_071104982.1 for PSI blast |  |  |
  | 333Select seq ref|WP\_070393639.1| | DNA polymerase III subunit beta [Moorea producens] | Moorea producens | NA | 1155739 | 72.8 | 72.8 | 95% | 6e-11 | 15.75% | 393 | WP\_070393639.1(scored below threshold on previous iteration) | Select seq WP\_070393639.1 for PSI blast |  |  |
  | 334Select seq ref|WP\_041965163.1| | DNA polymerase III subunit beta [Mesobacillus selenatarsenatis] | Mesobacillus selenatarsenatis | NA | 388741 | 72.8 | 72.8 | 95% | 6e-11 | 18.33% | 378 | WP\_041965163.1(scored below threshold on previous iteration) | Select seq WP\_041965163.1 for PSI blast |  |  |
  | 335Select seq ref|WP\_160726010.1| | DNA polymerase III subunit beta [Bacillus sp. USDA818B3\_A] | Bacillus sp. USDA818B3\_A | NA | 2698834 | 72.8 | 72.8 | 95% | 6e-11 | 16.32% | 378 | WP\_160726010.1(scored below threshold on previous iteration) | Select seq WP\_160726010.1 for PSI blast |  |  |
  | 336Select seq ref|WP\_133332786.1| | DNA polymerase III subunit beta [Bacillus sp. WN066] | Bacillus sp. WN066 | NA | 2547811 | 72.8 | 72.8 | 95% | 6e-11 | 16.87% | 378 | WP\_133332786.1(scored below threshold on previous iteration) | Select seq WP\_133332786.1 for PSI blast |  |  |
  | 337Select seq gb|NEN96443.1| | DNA polymerase III subunit beta [Moorea sp. SIO3I7] | Moorea sp. SIO3I7 | NA | 2607832 | 72.8 | 72.8 | 95% | 6e-11 | 15.35% | 393 | NEN96443.1(scored below threshold on previous iteration) | Select seq NEN96443.1 for PSI blast |  |  |
  | 338Select seq ref|WP\_036504199.1| | DNA polymerase [Nitrosococcus oceani] | Nitrosococcus oceani | NA | 1229 | 68.1 | 68.1 | 40% | 6e-11 | 22.55% | 102 | WP\_036504199.1(scored below threshold on previous iteration) | Select seq WP\_036504199.1 for PSI blast |  |  |
  | 339Select seq ref|WP\_048827549.1| | DNA polymerase III subunit beta [Bacillus sp. B-jedd] | Bacillus sp. B-jedd | NA | 1476857 | 72.8 | 72.8 | 95% | 6e-11 | 16.39% | 378 | WP\_048827549.1(scored below threshold on previous iteration) | Select seq WP\_048827549.1 for PSI blast |  |  |
  | 340Select seq gb|NEO82181.1| | DNA polymerase III subunit beta [Moorea sp. SIO4G3] | Moorea sp. SIO4G3 | NA | 2607821 | 72.8 | 72.8 | 95% | 6e-11 | 15.75% | 393 | NEO82181.1(scored below threshold on previous iteration) | Select seq NEO82181.1 for PSI blast |  |  |
  | 341Select seq gb|TAF47008.1| | DNA polymerase III subunit beta [Oscillatoriales cyanobacterium] | Oscillatoriales cyanobacterium | NA | 1151 | 72.8 | 72.8 | 95% | 6e-11 | 15.10% | 379 | TAF47008.1(scored below threshold on previous iteration) | Select seq TAF47008.1 for PSI blast |  |  |
  | 342Select seq gb|TVQ47881.1| | DNA polymerase III subunit beta [Gloeocapsa sp. DLM2.Bin57] | Gloeocapsa sp. DLM2.Bin57 | NA | 2480214 | 72.8 | 72.8 | 95% | 6e-11 | 17.28% | 379 | TVQ47881.1(scored below threshold on previous iteration) | Select seq TVQ47881.1 for PSI blast |  |  |
  | 343Select seq gb|TAD81354.1| | DNA polymerase III subunit beta [Oscillatoriales cyanobacterium] | Oscillatoriales cyanobacterium | NA | 1151 | 72.8 | 72.8 | 95% | 7e-11 | 15.10% | 379 | TAD81354.1(scored below threshold on previous iteration) | Select seq TAD81354.1 for PSI blast |  |  |
  | 344Select seq ref|WP\_190591433.1| | MULTISPECIES: DNA polymerase III subunit beta [Nostocales] | Nostocales | NA | 1161 | 72.8 | 72.8 | 95% | 7e-11 | 15.85% | 383 | WP\_190591433.1(scored below threshold on previous iteration) | Select seq WP\_190591433.1 for PSI blast |  |  |
  | 345Select seq ref|WP\_167830951.1| | DNA polymerase III subunit beta [Mesobacillus selenatarsenatis] | Mesobacillus selenatarsenatis | NA | 388741 | 72.8 | 72.8 | 95% | 7e-11 | 17.92% | 378 | WP\_167830951.1(scored below threshold on previous iteration) | Select seq WP\_167830951.1 for PSI blast |  |  |
  | 346Select seq ref|WP\_075900693.1| | DNA polymerase III subunit beta [Moorea bouillonii] | Moorea bouillonii | NA | 207920 | 72.4 | 72.4 | 95% | 8e-11 | 15.75% | 393 | WP\_075900693.1(scored below threshold on previous iteration) | Select seq WP\_075900693.1 for PSI blast |  |  |
  | 347Select seq gb|NEO06684.1| | DNA polymerase III subunit beta [Moorea sp. SIO3I8] | Moorea sp. SIO3I8 | NA | 2607833 | 72.4 | 72.4 | 95% | 8e-11 | 16.14% | 393 | NEO06684.1(scored below threshold on previous iteration) | Select seq NEO06684.1 for PSI blast |  |  |
  | 348Select seq ref|WP\_006527520.1| | DNA polymerase III subunit beta [Gloeocapsa sp. PCC 73106] | Gloeocapsa sp. PCC 73106 | NA | 102232 | 72.4 | 72.4 | 95% | 8e-11 | 16.87% | 377 | WP\_006527520.1(scored below threshold on previous iteration) | Select seq WP\_006527520.1 for PSI blast |  |  |
  | 349Select seq gb|NEO24005.1| | DNA polymerase III subunit beta [Moorea sp. SIO4A5] | Moorea sp. SIO4A5 | NA | 2607838 | 72.4 | 72.4 | 95% | 8e-11 | 16.14% | 393 | NEO24005.1(scored below threshold on previous iteration) | Select seq NEO24005.1 for PSI blast |  |  |
  | 350Select seq ref|WP\_095245542.1| | DNA polymerase III subunit beta [Bacillus sp. 7894-2] | Bacillus sp. 7894-2 | NA | 2021695 | 72.4 | 72.4 | 95% | 9e-11 | 19.17% | 378 | WP\_095245542.1(scored below threshold on previous iteration) | Select seq WP\_095245542.1 for PSI blast |  |  |
  | 351Select seq ref|WP\_198311027.1| | DNA polymerase III subunit beta [Neobacillus cucumis] | Neobacillus cucumis | NA | 1740721 | 72.4 | 72.4 | 95% | 9e-11 | 17.28% | 378 | WP\_198311027.1(scored below threshold on previous iteration) | Select seq WP\_198311027.1 for PSI blast |  |  |
  | 352Select seq gb|NEO02554.1| | DNA polymerase III subunit beta [Moorea sp. SIO3I7] | Moorea sp. SIO3I7 | NA | 2607832 | 72.0 | 72.0 | 95% | 9e-11 | 16.14% | 317 | NEO02554.1(scored below threshold on previous iteration) | Select seq NEO02554.1 for PSI blast |  |  |
  | 353Select seq ref|WP\_131843359.1| | DNA polymerase III subunit beta [Bacillus sp. BK245] | Bacillus sp. BK245 | NA | 2512184 | 72.4 | 72.4 | 95% | 9e-11 | 16.32% | 381 | WP\_131843359.1(scored below threshold on previous iteration) | Select seq WP\_131843359.1 for PSI blast |  |  |
  | 354Select seq ref|WP\_102264757.1| | DNA polymerase III subunit beta [Mesobacillus jeotgali] | Mesobacillus jeotgali | NA | 129985 | 72.4 | 72.4 | 95% | 9e-11 | 17.92% | 378 | WP\_102264757.1(scored below threshold on previous iteration) | Select seq WP\_102264757.1 for PSI blast |  |  |
  | 355Select seq ref|WP\_100332693.1| | DNA polymerase III subunit beta [Bacillus xiapuensis] | Bacillus xiapuensis | NA | 2014075 | 72.0 | 72.0 | 95% | 1e-10 | 16.32% | 378 | WP\_100332693.1(scored below threshold on previous iteration) | Select seq WP\_100332693.1 for PSI blast |  |  |
  | 356Select seq gb|NEQ16245.1| | DNA polymerase III subunit beta [Moorea sp. SIO3E2] | Moorea sp. SIO3E2 | NA | 2607829 | 72.0 | 72.0 | 95% | 1e-10 | 15.35% | 393 | NEQ16245.1(scored below threshold on previous iteration) | Select seq NEQ16245.1 for PSI blast |  |  |
  | 357Select seq ref|WP\_192472743.1| | unnamed protein product |  |  | 0 | 72.0 | 143 | 95% | 1e-10 | 17.92% | 378 | WP\_192472743.1(scored below threshold on previous iteration) | Select seq WP\_192472743.1 for PSI blast |  |  |
  | 358Select seq ref|WP\_075983368.1| | DNA polymerase III subunit beta [Bacillus massilionigeriensis] | Bacillus massilionigeriensis | NA | 1805475 | 72.0 | 72.0 | 95% | 1e-10 | 17.43% | 378 | WP\_075983368.1(scored below threshold on previous iteration) | Select seq WP\_075983368.1 for PSI blast |  |  |
  | 359Select seq ref|WP\_101548481.1| | DNA polymerase III subunit beta [Bacillus sp. UMB0728] | Bacillus sp. UMB0728 | NA | 2066052 | 72.0 | 72.0 | 95% | 1e-10 | 16.81% | 378 | WP\_101548481.1(scored below threshold on previous iteration) | Select seq WP\_101548481.1 for PSI blast |  |  |
  | 360Select seq ref|WP\_166245254.1| | DNA polymerase III subunit beta [Bacillus sp. MM2020\_4] | Bacillus sp. MM2020\_4 | NA | 2714039 | 72.0 | 72.0 | 95% | 1e-10 | 16.74% | 378 | WP\_166245254.1(scored below threshold on previous iteration) | Select seq WP\_166245254.1 for PSI blast |  |  |
  | 361Select seq ref|WP\_118921193.1| | DNA polymerase III subunit beta [Neobacillus notoginsengisoli] | Neobacillus notoginsengisoli | NA | 1578198 | 72.0 | 72.0 | 95% | 1e-10 | 15.97% | 378 | WP\_118921193.1(scored below threshold on previous iteration) | Select seq WP\_118921193.1 for PSI blast |  |  |
  | 362Select seq ref|WP\_119548028.1| | DNA polymerase III subunit beta [Bacillus salacetis] | Bacillus salacetis | NA | 2315464 | 72.0 | 72.0 | 95% | 1e-10 | 16.67% | 378 | WP\_119548028.1(scored below threshold on previous iteration) | Select seq WP\_119548028.1 for PSI blast |  |  |
  | 363Select seq ref|WP\_009791671.1| | MULTISPECIES: DNA polymerase III subunit beta [Bacillus] | Bacillus | NA | 1386 | 72.0 | 72.0 | 95% | 1e-10 | 16.39% | 378 | WP\_009791671.1(scored below threshold on previous iteration) | Select seq WP\_009791671.1 for PSI blast |  |  |
  | 364Select seq ref|WP\_044392047.1| | DNA polymerase III subunit beta [Mesobacillus subterraneus] | Mesobacillus subterraneus | NA | 285983 | 71.6 | 71.6 | 95% | 1e-10 | 18.33% | 378 | WP\_044392047.1(scored below threshold on previous iteration) | Select seq WP\_044392047.1 for PSI blast |  |  |
  | 365Select seq ref|WP\_040206650.1| | MULTISPECIES: DNA polymerase III subunit beta [Bacillaceae] | Bacillaceae | NA | 186817 | 71.6 | 71.6 | 95% | 1e-10 | 17.15% | 378 | WP\_040206650.1(scored below threshold on previous iteration) | Select seq WP\_040206650.1 for PSI blast |  |  |
  | 366Select seq ref|WP\_148976131.1| | DNA polymerase III subunit beta [Bacillus infantis] | Bacillus infantis | NA | 324767 | 71.6 | 71.6 | 95% | 1e-10 | 16.81% | 378 | WP\_148976131.1(scored below threshold on previous iteration) | Select seq WP\_148976131.1 for PSI blast |  |  |
  | 367Select seq ref|WP\_044745144.1| | MULTISPECIES: DNA polymerase III subunit beta [Anoxybacillus] | Anoxybacillus | NA | 150247 | 71.6 | 71.6 | 95% | 2e-10 | 18.67% | 378 | WP\_044745144.1(scored below threshold on previous iteration) | Select seq WP\_044745144.1 for PSI blast |  |  |
  | 368Select seq ref|WP\_098808620.1| | DNA polymerase III subunit beta [Bacillus sp. AFS041924] | Bacillus sp. AFS041924 | NA | 2033503 | 71.6 | 71.6 | 95% | 2e-10 | 15.83% | 381 | WP\_098808620.1(scored below threshold on previous iteration) | Select seq WP\_098808620.1 for PSI blast |  |  |
  | 369Select seq ref|WP\_066205231.1| | DNA polymerase III subunit beta [Bacillus sp. FJAT-27225] | Bacillus sp. FJAT-27225 | NA | 1743144 | 71.6 | 71.6 | 95% | 2e-10 | 17.23% | 378 | WP\_066205231.1(scored below threshold on previous iteration) | Select seq WP\_066205231.1 for PSI blast |  |  |
  | 370Select seq ref|WP\_094767786.1| | MULTISPECIES: DNA polymerase III subunit beta [Bacillus] | Bacillus | NA | 1386 | 71.2 | 71.2 | 95% | 2e-10 | 16.81% | 378 | WP\_094767786.1(scored below threshold on previous iteration) | Select seq WP\_094767786.1 for PSI blast |  |  |
  | 371Select seq ref|WP\_007084524.1| | DNA polymerase III subunit beta [Neobacillus bataviensis] | Neobacillus bataviensis | NA | 220685 | 71.2 | 71.2 | 95% | 2e-10 | 17.15% | 378 | WP\_007084524.1(scored below threshold on previous iteration) | Select seq WP\_007084524.1 for PSI blast |  |  |
  | 372Select seq ref|WP\_071289881.1| | proliferating cell nuclear antigen (pcna) [Acinetobacter baumannii] | Acinetobacter baumannii | NA | 470 | 67.0 | 67.0 | 40% | 2e-10 | 14.29% | 108 | WP\_071289881.1(scored below threshold on previous iteration) | Select seq WP\_071289881.1 for PSI blast |  |  |
  | 373Select seq gb|TAG92537.1| | DNA polymerase III subunit beta [Oscillatoriales cyanobacterium] | Oscillatoriales cyanobacterium | NA | 1151 | 71.2 | 71.2 | 95% | 2e-10 | 16.73% | 379 | TAG92537.1(scored below threshold on previous iteration) | Select seq TAG92537.1 for PSI blast |  |  |
  | 374Select seq ref|WP\_080862673.1| | DNA polymerase III subunit beta [Anoxybacillus sp. UARK-01] | Anoxybacillus sp. UARK-01 | NA | 1895648 | 71.2 | 71.2 | 95% | 2e-10 | 18.67% | 378 | WP\_080862673.1(scored below threshold on previous iteration) | Select seq WP\_080862673.1 for PSI blast |  |  |
  | 375Select seq gb|KPC97898.1| | DNA polymerase III subunit beta [Geobacillus sp. BCO2] | Geobacillus sp. BCO2 | NA | 1547578 | 71.2 | 71.2 | 95% | 2e-10 | 17.84% | 378 | KPC97898.1(scored below threshold on previous iteration) | Select seq KPC97898.1 for PSI blast |  |  |
  | 376Select seq gb|MBA1334753.1| | DNA polymerase III beta subunit [Firmicutes bacterium] | Firmicutes bacterium | NA | 1879010 | 70.8 | 70.8 | 95% | 2e-10 | 18.11% | 367 | MBA1334753.1(scored below threshold on previous iteration) | Select seq MBA1334753.1 for PSI blast |  |  |
  | 377Select seq ref|WP\_098171591.1| | DNA polymerase III subunit beta [Bacillus sp. AFS017336] | Bacillus sp. AFS017336 | NA | 2033489 | 71.2 | 71.2 | 95% | 2e-10 | 16.25% | 381 | WP\_098171591.1(scored below threshold on previous iteration) | Select seq WP\_098171591.1 for PSI blast |  |  |
  | 378Select seq ref|WP\_049683504.1| | DNA polymerase III subunit beta [Peribacillus loiseleuriae] | Peribacillus loiseleuriae | NA | 1679170 | 70.8 | 70.8 | 95% | 3e-10 | 17.50% | 378 | WP\_049683504.1(scored below threshold on previous iteration) | Select seq WP\_049683504.1 for PSI blast |  |  |
  | 379Select seq ref|WP\_096624290.1| | DNA polymerase III subunit beta [Calothrix sp. NIES-3974] | Calothrix sp. NIES-3974 | NA | 2005462 | 70.8 | 70.8 | 95% | 3e-10 | 14.29% | 385 | WP\_096624290.1(scored below threshold on previous iteration) | Select seq WP\_096624290.1 for PSI blast |  |  |
  | 380Select seq gb|NEO39042.1| | DNA polymerase III subunit beta [Moorea sp. SIOASIH] | Moorea sp. SIOASIH | NA | 2607817 | 70.8 | 70.8 | 95% | 3e-10 | 15.35% | 393 | NEO39042.1(scored below threshold on previous iteration) | Select seq NEO39042.1 for PSI blast |  |  |
  | 381Select seq ref|WP\_098119092.1| | DNA polymerase III subunit beta [Bacillus sp. AFS002410] | Bacillus sp. AFS002410 | NA | 2033481 | 70.8 | 70.8 | 95% | 3e-10 | 16.25% | 381 | WP\_098119092.1(scored below threshold on previous iteration) | Select seq WP\_098119092.1 for PSI blast |  |  |
  | 382Select seq ref|WP\_095247370.1| | DNA polymerase III subunit beta [Bacillus sp. 7884-1] | Bacillus sp. 7884-1 | NA | 2021693 | 70.8 | 70.8 | 95% | 3e-10 | 16.74% | 378 | WP\_095247370.1(scored below threshold on previous iteration) | Select seq WP\_095247370.1 for PSI blast |  |  |
  | 383Select seq ref|WP\_069033636.1| | MULTISPECIES: DNA polymerase III subunit beta [Bacillus] | Bacillus | NA | 1386 | 70.8 | 70.8 | 95% | 3e-10 | 16.25% | 381 | WP\_069033636.1(scored below threshold on previous iteration) | Select seq WP\_069033636.1 for PSI blast |  |  |
  | 384Select seq ref|WP\_066074203.1| | DNA polymerase III subunit beta [Neobacillus soli] | Neobacillus soli | NA | 220688 | 70.8 | 70.8 | 95% | 3e-10 | 17.15% | 378 | WP\_066074203.1(scored below threshold on previous iteration) | Select seq WP\_066074203.1 for PSI blast |  |  |
  | 385Select seq ref|WP\_088001664.1| | DNA polymerase III subunit beta [Bacillus solisilvae] | Bacillus solisilvae | NA | 1516104 | 70.8 | 70.8 | 95% | 3e-10 | 15.83% | 381 | WP\_088001664.1(scored below threshold on previous iteration) | Select seq WP\_088001664.1 for PSI blast |  |  |
  | 386Select seq gb|MBE1408962.1| | DNA polymerase-3 subunit beta [Bacillus sp. OAE603] | Bacillus sp. OAE603 | NA | 2663872 | 70.8 | 70.8 | 95% | 3e-10 | 15.83% | 381 | MBE1408962.1(scored below threshold on previous iteration) | Select seq MBE1408962.1 for PSI blast |  |  |
  | 387Select seq ref|WP\_133369876.1| | DNA polymerase III subunit beta [Rhodococcus qingshengii] | Rhodococcus qingshengii | NA | 334542 | 70.4 | 70.4 | 95% | 3e-10 | 16.74% | 378 | WP\_133369876.1(scored below threshold on previous iteration) | Select seq WP\_133369876.1 for PSI blast |  |  |
  | 388Select seq ref|WP\_097976410.1| | MULTISPECIES: DNA polymerase III subunit beta [unclassified Bacillus] | unclassified Bacillus | NA | 185979 | 70.4 | 70.4 | 95% | 3e-10 | 16.25% | 381 | WP\_097976410.1(scored below threshold on previous iteration) | Select seq WP\_097976410.1 for PSI blast |  |  |
  | 389Select seq ref|WP\_175244178.1| | DNA polymerase III subunit beta [Parageobacillus sp. VR-IP] | Parageobacillus sp. VR-IP | NA | 2742205 | 70.4 | 70.4 | 95% | 3e-10 | 18.26% | 378 | WP\_175244178.1(scored below threshold on previous iteration) | Select seq WP\_175244178.1 for PSI blast |  |  |
  | 390Select seq ref|WP\_059171609.1| | DNA polymerase III subunit beta [Bacillus sp. FJAT-27445] | Bacillus sp. FJAT-27445 | NA | 1679166 | 70.4 | 70.4 | 95% | 4e-10 | 18.18% | 378 | WP\_059171609.1(scored below threshold on previous iteration) | Select seq WP\_059171609.1 for PSI blast |  |  |
  | 391Select seq ref|WP\_115450924.1| | DNA polymerase III subunit beta [Bacillus piezotolerans] | Bacillus piezotolerans | NA | 2259171 | 70.4 | 70.4 | 95% | 4e-10 | 16.39% | 378 | WP\_115450924.1(scored below threshold on previous iteration) | Select seq WP\_115450924.1 for PSI blast |  |  |
  | 392Select seq ref|WP\_101577100.1| | DNA polymerase III subunit beta [Bacillus canaveralius] | Bacillus canaveralius | NA | 1403243 | 70.4 | 70.4 | 95% | 4e-10 | 16.67% | 378 | WP\_101577100.1(scored below threshold on previous iteration) | Select seq WP\_101577100.1 for PSI blast |  |  |
  | 393Select seq ref|WP\_172446568.1| | DNA polymerase III subunit beta [Arthrobacter citreus] | Arthrobacter citreus | NA | 1670 | 70.4 | 70.4 | 95% | 4e-10 | 16.25% | 381 | WP\_172446568.1(scored below threshold on previous iteration) | Select seq WP\_172446568.1 for PSI blast |  |  |
  | 394Select seq ref|WP\_043934288.1| | DNA polymerase III subunit beta [Bacillus sp. EB01] | Bacillus sp. EB01 | NA | 1347086 | 70.4 | 70.4 | 95% | 4e-10 | 18.52% | 378 | WP\_043934288.1(scored below threshold on previous iteration) | Select seq WP\_043934288.1 for PSI blast |  |  |
  | 395Select seq ref|WP\_094244820.1| | DNA polymerase III subunit beta [Aeribacillus pallidus] | Aeribacillus pallidus | NA | 33936 | 70.4 | 70.4 | 90% | 4e-10 | 15.04% | 378 | WP\_094244820.1(scored below threshold on previous iteration) | Select seq WP\_094244820.1 for PSI blast |  |  |
  | 396Select seq ref|WP\_098859933.1| | DNA polymerase III subunit beta [Bacillus sp. AFS053548] | Bacillus sp. AFS053548 | NA | 2033505 | 70.4 | 70.4 | 95% | 4e-10 | 15.83% | 381 | WP\_098859933.1(scored below threshold on previous iteration) | Select seq WP\_098859933.1 for PSI blast |  |  |
  | 397Select seq ref|WP\_098844404.1| | DNA polymerase III subunit beta [Bacillus sp. AFS055030] | Bacillus sp. AFS055030 | NA | 2033507 | 70.4 | 70.4 | 95% | 4e-10 | 16.25% | 381 | WP\_098844404.1(scored below threshold on previous iteration) | Select seq WP\_098844404.1 for PSI blast |  |  |
  | 398Select seq ref|WP\_053597522.1| | DNA polymerase III subunit beta [Bacillus sp. FJAT-18017] | Bacillus sp. FJAT-18017 | NA | 1705566 | 70.4 | 70.4 | 95% | 4e-10 | 18.52% | 378 | WP\_053597522.1(scored below threshold on previous iteration) | Select seq WP\_053597522.1 for PSI blast |  |  |
  | 399Select seq ref|WP\_066251628.1| | MULTISPECIES: DNA polymerase III subunit beta [Aeribacillus] | Aeribacillus | NA | 1055323 | 70.4 | 70.4 | 90% | 4e-10 | 15.04% | 378 | WP\_066251628.1(scored below threshold on previous iteration) | Select seq WP\_066251628.1 for PSI blast |  |  |
  | 400Select seq ref|WP\_098429558.1| | DNA polymerase III subunit beta [Bacillus sp. AFS088145] | Bacillus sp. AFS088145 | NA | 2033514 | 70.4 | 70.4 | 95% | 4e-10 | 15.83% | 381 | WP\_098429558.1(scored below threshold on previous iteration) | Select seq WP\_098429558.1 for PSI blast |  |  |
  | 401Select seq ref|WP\_101667026.1| | DNA polymerase III subunit beta [Bacillus sp. V33-4] | Bacillus sp. V33-4 | NA | 2054169 | 70.4 | 70.4 | 95% | 4e-10 | 16.67% | 378 | WP\_101667026.1(scored below threshold on previous iteration) | Select seq WP\_101667026.1 for PSI blast |  |  |
  | 402Select seq ref|WP\_101658871.1| | DNA polymerase III subunit beta [Bacillus sp. V3-13] | Bacillus sp. V3-13 | NA | 2053728 | 70.4 | 70.4 | 95% | 4e-10 | 17.08% | 378 | WP\_101658871.1(scored below threshold on previous iteration) | Select seq WP\_101658871.1 for PSI blast |  |  |
  | 403Select seq ref|WP\_015591853.1| | DNA polymerase III subunit beta [Bacillus sp. 1NLA3E] | Bacillus sp. 1NLA3E | NA | 666686 | 70.1 | 70.1 | 95% | 4e-10 | 19.58% | 378 | WP\_015591853.1(scored below threshold on previous iteration) | Select seq WP\_015591853.1 for PSI blast |  |  |
  | 404Select seq ref|WP\_045519334.1| | DNA polymerase III subunit beta [Neobacillus niacini] | Neobacillus niacini | NA | 86668 | 70.1 | 70.1 | 95% | 5e-10 | 16.74% | 378 | WP\_045519334.1(scored below threshold on previous iteration) | Select seq WP\_045519334.1 for PSI blast |  |  |
  | 405Select seq ref|WP\_148939485.1| | DNA polymerase III subunit beta [Bacillus vietnamensis] | Bacillus vietnamensis | NA | 218284 | 70.1 | 70.1 | 95% | 5e-10 | 16.60% | 378 | WP\_148939485.1(scored below threshold on previous iteration) | Select seq WP\_148939485.1 for PSI blast |  |  |
  | 406Select seq ref|WP\_098577012.1| | DNA polymerase III subunit beta [Bacillus sp. AFS073361] | Bacillus sp. AFS073361 | NA | 2033511 | 70.1 | 70.1 | 95% | 5e-10 | 17.15% | 378 | WP\_098577012.1(scored below threshold on previous iteration) | Select seq WP\_098577012.1 for PSI blast |  |  |
  | 407Select seq ref|WP\_129689568.1| | DNA polymerase III subunit beta [Bacillus acidiceler] | Bacillus acidiceler | NA | 371036 | 70.1 | 70.1 | 89% | 5e-10 | 15.86% | 381 | WP\_129689568.1(scored below threshold on previous iteration) | Select seq WP\_129689568.1 for PSI blast |  |  |
  | 408Select seq ref|WP\_098232436.1| | DNA polymerase III subunit beta [Bacillus sp. AFS001701] | Bacillus sp. AFS001701 | NA | 2033480 | 70.1 | 70.1 | 95% | 5e-10 | 16.25% | 381 | WP\_098232436.1(scored below threshold on previous iteration) | Select seq WP\_098232436.1 for PSI blast |  |  |
  | 409Select seq ref|WP\_008232302.1| | DNA polymerase III subunit beta [Richelia intracellularis] | Richelia intracellularis | NA | 1164990 | 70.1 | 70.1 | 95% | 5e-10 | 17.74% | 387 | WP\_008232302.1(scored below threshold on previous iteration) | Select seq WP\_008232302.1 for PSI blast |  |  |
  | 410Select seq ref|WP\_146949895.1| | DNA polymerase III subunit beta [Metabacillus litoralis] | Metabacillus litoralis | NA | 152268 | 70.1 | 70.1 | 91% | 5e-10 | 17.32% | 378 | WP\_146949895.1(scored below threshold on previous iteration) | Select seq WP\_146949895.1 for PSI blast |  |  |
  | 411Select seq gb|MBE1354774.1| | DNA polymerase-3 subunit beta [Bacillus sp. OAE578] | Bacillus sp. OAE578 | NA | 2663871 | 70.1 | 70.1 | 95% | 5e-10 | 16.74% | 378 | MBE1354774.1(scored below threshold on previous iteration) | Select seq MBE1354774.1 for PSI blast |  |  |
  | 412Select seq ref|WP\_017435762.1| | MULTISPECIES: DNA polymerase III subunit beta [Bacillaceae] | Bacillaceae | NA | 186817 | 70.1 | 70.1 | 95% | 5e-10 | 18.26% | 378 | WP\_017435762.1(scored below threshold on previous iteration) | Select seq WP\_017435762.1 for PSI blast |  |  |
  | 413Select seq ref|WP\_056467133.1| | DNA polymerase III subunit beta [Bacillus sp. FJAT-25509] | Bacillus sp. FJAT-25509 | NA | 1712029 | 70.1 | 70.1 | 95% | 5e-10 | 16.25% | 381 | WP\_056467133.1(scored below threshold on previous iteration) | Select seq WP\_056467133.1 for PSI blast |  |  |
  | 414Select seq ref|WP\_020958258.1| | DNA polymerase III subunit beta [Geobacillus genomosp. 3] | Geobacillus genomosp. 3 | NA | 1921421 | 70.1 | 70.1 | 95% | 6e-10 | 16.67% | 378 | WP\_020958258.1(scored below threshold on previous iteration) | Select seq WP\_020958258.1 for PSI blast |  |  |
  | 415Select seq ref|WP\_148947202.1| | MULTISPECIES: DNA polymerase III subunit beta [Bacillus] | Bacillus | NA | 1386 | 69.7 | 69.7 | 95% | 6e-10 | 16.60% | 378 | WP\_148947202.1(scored below threshold on previous iteration) | Select seq WP\_148947202.1 for PSI blast |  |  |
  | 416Select seq ref|WP\_078543249.1| | DNA polymerase III subunit beta [Bacillus alkalitelluris] | Bacillus alkalitelluris | NA | 304268 | 69.7 | 69.7 | 95% | 6e-10 | 18.83% | 378 | WP\_078543249.1(scored below threshold on previous iteration) | Select seq WP\_078543249.1 for PSI blast |  |  |
  | 417Select seq ref|WP\_063255150.1| | DNA polymerase III subunit beta [Neobacillus niacini] | Neobacillus niacini | NA | 86668 | 69.7 | 69.7 | 95% | 7e-10 | 16.74% | 378 | WP\_063255150.1(scored below threshold on previous iteration) | Select seq WP\_063255150.1 for PSI blast |  |  |
  | 418Select seq ref|WP\_090634434.1| | DNA polymerase III subunit beta [Bacillus sp. LF1] | Bacillus sp. LF1 | NA | 1499688 | 69.7 | 69.7 | 95% | 7e-10 | 17.99% | 378 | WP\_090634434.1(scored below threshold on previous iteration) | Select seq WP\_090634434.1 for PSI blast |  |  |
  | 419Select seq ref|WP\_181555257.1| | DNA polymerase III subunit beta [Anoxybacillus caldiproteolyticus] | Anoxybacillus caldiproteolyticus | NA | 247480 | 69.7 | 69.7 | 95% | 7e-10 | 17.50% | 378 | WP\_181555257.1(scored below threshold on previous iteration) | Select seq WP\_181555257.1 for PSI blast |  |  |
  | 420Select seq ref|WP\_185764913.1| | DNA polymerase III subunit beta [Bacillus circulans] | Bacillus circulans | NA | 1397 | 69.7 | 69.7 | 95% | 7e-10 | 16.39% | 377 | WP\_185764913.1(scored below threshold on previous iteration) | Select seq WP\_185764913.1 for PSI blast |  |  |
  | 421Select seq ref|WP\_063387745.1| | DNA polymerase III subunit beta [Aeribacillus pallidus] | Aeribacillus pallidus | NA | 33936 | 69.7 | 69.7 | 90% | 8e-10 | 15.04% | 378 | WP\_063387745.1(scored below threshold on previous iteration) | Select seq WP\_063387745.1 for PSI blast |  |  |
  | 422Select seq ref|WP\_191566550.1| | DNA polymerase III subunit beta [Bacillus idriensis] | Bacillus idriensis | NA | 324768 | 69.3 | 69.3 | 95% | 8e-10 | 17.08% | 378 | WP\_191566550.1(scored below threshold on previous iteration) | Select seq WP\_191566550.1 for PSI blast |  |  |
  | 423Select seq ref|WP\_029761071.1| | MULTISPECIES: DNA polymerase III subunit beta [Geobacillus] | Geobacillus | NA | 129337 | 69.3 | 69.3 | 95% | 9e-10 | 17.43% | 378 | WP\_029761071.1(scored below threshold on previous iteration) | Select seq WP\_029761071.1 for PSI blast |  |  |
  | 424Select seq ref|WP\_053368624.1| | DNA polymerase III subunit beta [Bacillus sp. FJAT-27245] | Bacillus sp. FJAT-27245 | NA | 1684144 | 69.3 | 69.3 | 95% | 9e-10 | 16.81% | 378 | WP\_053368624.1(scored below threshold on previous iteration) | Select seq WP\_053368624.1 for PSI blast |  |  |
  | 425Select seq gb|QPC47933.1| | DNA polymerase III subunit beta [Bacillaceae bacterium R1DC41] | Bacillaceae bacterium R1DC41 | NA | 2593652 | 69.3 | 69.3 | 95% | 9e-10 | 15.90% | 378 | QPC47933.1(scored below threshold on previous iteration) | Select seq QPC47933.1 for PSI blast |  |  |
  | 426Select seq gb|EPR28620.1| | DNA polymerase III, beta chain [Geobacillus sp. WSUCF1] | Geobacillus sp. WSUCF1 | NA | 886559 | 68.1 | 68.1 | 95% | 9e-10 | 17.01% | 245 | EPR28620.1(scored below threshold on previous iteration) | Select seq EPR28620.1 for PSI blast |  |  |
  | 427Select seq gb|MAI63263.1| | hypothetical protein [Alteromonas sp.] | Alteromonas sp. | NA | 232 | 67.0 | 67.0 | 59% | 9e-10 | 15.82% | 175 | MAI63263.1(scored below threshold on previous iteration) | Select seq MAI63263.1 for PSI blast |  |  |
  | 428Select seq gb|AEA77211.1| | DNA polymerase III beta subunit [Bacillus sp. 15.4] | Bacillus sp. 15.4 | NA | 999693 | 69.3 | 69.3 | 95% | 9e-10 | 16.60% | 361 | AEA77211.1(scored below threshold on previous iteration) | Select seq AEA77211.1 for PSI blast |  |  |
  | 429Select seq ref|WP\_179157904.1| | DNA polymerase III subunit beta [Bacillus sp. EB106-08-02-XG196] | Bacillus sp. EB106-08-02-XG196 | NA | 2737049 | 69.3 | 69.3 | 95% | 9e-10 | 16.32% | 378 | WP\_179157904.1(scored below threshold on previous iteration) | Select seq WP\_179157904.1 for PSI blast |  |  |
  | 430Select seq ref|WP\_101567166.1| | MULTISPECIES: DNA polymerase III subunit beta [Bacillus] | Bacillus | NA | 1386 | 69.3 | 69.3 | 95% | 9e-10 | 17.08% | 378 | WP\_101567166.1(scored below threshold on previous iteration) | Select seq WP\_101567166.1 for PSI blast |  |  |
  | 431Select seq ref|WP\_066389915.1| | DNA polymerase III subunit beta [Neobacillus mesonae] | Neobacillus mesonae | NA | 1193713 | 69.3 | 69.3 | 95% | 1e-09 | 15.90% | 378 | WP\_066389915.1(scored below threshold on previous iteration) | Select seq WP\_066389915.1 for PSI blast |  |  |
  | 432Select seq ref|WP\_107901004.1| | DNA polymerase III subunit beta [Bacillus sp. OV186] | Bacillus sp. OV186 | NA | 2135741 | 69.3 | 69.3 | 95% | 1e-09 | 17.08% | 381 | WP\_107901004.1(scored below threshold on previous iteration) | Select seq WP\_107901004.1 for PSI blast |  |  |
  | 433Select seq ref|WP\_071393107.1| | DNA polymerase III subunit beta [Bacillus tuaregi] | Bacillus tuaregi | NA | 1816695 | 69.3 | 69.3 | 95% | 1e-09 | 17.57% | 378 | WP\_071393107.1(scored below threshold on previous iteration) | Select seq WP\_071393107.1 for PSI blast |  |  |
  | 434Select seq ref|WP\_049624130.1| | DNA polymerase III subunit beta [Geobacillus stearothermophilus] | Geobacillus stearothermophilus | NA | 1422 | 69.3 | 69.3 | 95% | 1e-09 | 17.43% | 378 | WP\_049624130.1(scored below threshold on previous iteration) | Select seq WP\_049624130.1 for PSI blast |  |  |
  | 435Select seq ref|WP\_127738797.1| | MULTISPECIES: DNA polymerase III subunit beta [Bacillus] | Bacillus | NA | 1386 | 69.3 | 69.3 | 95% | 1e-09 | 16.39% | 377 | WP\_127738797.1(scored below threshold on previous iteration) | Select seq WP\_127738797.1 for PSI blast |  |  |
  | 436Select seq ref|WP\_008880834.1| | DNA polymerase III subunit beta [Geobacillus thermodenitrificans] | Geobacillus thermodenitrificans | NA | 33940 | 69.3 | 69.3 | 95% | 1e-09 | 17.43% | 378 | WP\_008880834.1(scored below threshold on previous iteration) | Select seq WP\_008880834.1 for PSI blast |  |  |
  | 437Select seq gb|REJ16426.1| | DNA polymerase III subunit beta [Bacillaceae bacterium] | Bacillaceae bacterium | NA | 1889774 | 69.3 | 69.3 | 90% | 1e-09 | 15.04% | 378 | REJ16426.1(scored below threshold on previous iteration) | Select seq REJ16426.1 for PSI blast |  |  |
  | 438Select seq ref|WP\_077215233.1| | DNA polymerase III subunit beta [Bacillus dakarensis] | Bacillus dakarensis | NA | 1926278 | 69.3 | 69.3 | 95% | 1e-09 | 19.17% | 378 | WP\_077215233.1(scored below threshold on previous iteration) | Select seq WP\_077215233.1 for PSI blast |  |  |
  | 439Select seq ref|WP\_062754547.1| | MULTISPECIES: DNA polymerase III subunit beta [Bacillaceae] | Bacillaceae | NA | 186817 | 69.3 | 69.3 | 95% | 1e-09 | 17.01% | 378 | WP\_062754547.1(scored below threshold on previous iteration) | Select seq WP\_062754547.1 for PSI blast |  |  |
  | 440Select seq ref|WP\_144454962.1| | DNA polymerase III subunit beta [Bacillus sp. DE0587] | Bacillus sp. DE0587 | NA | 2586641 | 69.3 | 69.3 | 95% | 1e-09 | 16.39% | 377 | WP\_144454962.1(scored below threshold on previous iteration) | Select seq WP\_144454962.1 for PSI blast |  |  |
  | 441Select seq ref|WP\_006837290.1| | DNA polymerase III subunit beta [Bacillus sp. SG-1] | Bacillus sp. SG-1 | NA | 161544 | 69.3 | 69.3 | 95% | 1e-09 | 16.18% | 378 | WP\_006837290.1(scored below threshold on previous iteration) | Select seq WP\_006837290.1 for PSI blast |  |  |
  | 442Select seq ref|WP\_143416363.1| | DNA polymerase III subunit beta [Geobacillus sp. E263] | Geobacillus sp. E263 | NA | 391290 | 69.3 | 69.3 | 95% | 1e-09 | 17.01% | 378 | WP\_143416363.1(scored below threshold on previous iteration) | Select seq WP\_143416363.1 for PSI blast |  |  |
  | 443Select seq ref|WP\_113927364.1| | MULTISPECIES: DNA polymerase III subunit beta [Bacillus] | Bacillus | NA | 1386 | 69.3 | 69.3 | 95% | 1e-09 | 16.60% | 378 | WP\_113927364.1(scored below threshold on previous iteration) | Select seq WP\_113927364.1 for PSI blast |  |  |
  | 444Select seq ref|WP\_095302033.1| | MULTISPECIES: DNA polymerase III subunit beta [Bacillaceae] | Bacillaceae | NA | 186817 | 69.3 | 69.3 | 92% | 1e-09 | 15.95% | 378 | WP\_095302033.1(scored below threshold on previous iteration) | Select seq WP\_095302033.1 for PSI blast |  |  |
  | 445Select seq ref|WP\_033011986.1| | MULTISPECIES: DNA polymerase III subunit beta [Geobacillus] | Geobacillus | NA | 129337 | 69.3 | 69.3 | 95% | 1e-09 | 17.43% | 378 | WP\_033011986.1(scored below threshold on previous iteration) | Select seq WP\_033011986.1 for PSI blast |  |  |
  | 446Select seq ref|WP\_144556091.1| | DNA polymerase III subunit beta [Bacillus sp. X1(2014)] | Bacillus sp. X1(2014) | NA | 1565991 | 69.3 | 69.3 | 95% | 1e-09 | 17.15% | 378 | WP\_144556091.1(scored below threshold on previous iteration) | Select seq WP\_144556091.1 for PSI blast |  |  |
  | 447Select seq ref|WP\_066338790.1| | MULTISPECIES: DNA polymerase III subunit beta [Metabacillus] | Metabacillus | NA | 2675233 | 69.3 | 69.3 | 91% | 1e-09 | 17.32% | 378 | WP\_066338790.1(scored below threshold on previous iteration) | Select seq WP\_066338790.1 for PSI blast |  |  |
  | 448Select seq gb|MBB3868452.1| | DNA polymerase-3 subunit beta [Parageobacillus toebii NBRC 107807] | Parageobacillus toebii NBRC 107807 | NA | 1223503 | 68.9 | 68.9 | 95% | 1e-09 | 17.01% | 379 | MBB3868452.1(scored below threshold on previous iteration) | Select seq MBB3868452.1 for PSI blast |  |  |
  | 449Select seq ref|WP\_185771847.1| | DNA polymerase III subunit beta [Bacillus sp. PAMC26568] | Bacillus sp. PAMC26568 | NA | 2760090 | 68.9 | 68.9 | 95% | 1e-09 | 17.08% | 378 | WP\_185771847.1(scored below threshold on previous iteration) | Select seq WP\_185771847.1 for PSI blast |  |  |
  | 450Select seq tpg|HIK37546.1| | TPA: DNA polymerase III subunit beta [Geminocystis sp. M7585\_C2015\_104] | Geminocystis sp. M7585\_C2015\_104 | NA | 2774334 | 68.9 | 68.9 | 95% | 1e-09 | 16.33% | 382 | HIK37546.1(scored below threshold on previous iteration) | Select seq HIK37546.1 for PSI blast |  |  |
  | 451Select seq ref|WP\_027408147.1| | DNA polymerase III subunit beta [Anoxybacillus tepidamans] | Anoxybacillus tepidamans | NA | 265948 | 68.9 | 68.9 | 95% | 1e-09 | 17.43% | 378 | WP\_027408147.1(scored below threshold on previous iteration) | Select seq WP\_027408147.1 for PSI blast |  |  |
  | 452Select seq ref|WP\_132007753.1| | DNA polymerase III subunit beta [Mesobacillus foraminis] | Mesobacillus foraminis | NA | 279826 | 68.9 | 68.9 | 95% | 1e-09 | 17.15% | 378 | WP\_132007753.1(scored below threshold on previous iteration) | Select seq WP\_132007753.1 for PSI blast |  |  |
  | 453Select seq ref|WP\_017547447.1| | DNA polymerase III subunit beta [Salinicoccus carnicancri] | Salinicoccus carnicancri | NA | 558170 | 68.9 | 68.9 | 92% | 1e-09 | 15.68% | 377 | WP\_017547447.1(scored below threshold on previous iteration) | Select seq WP\_017547447.1 for PSI blast |  |  |
  | 454Select seq ref|WP\_111617261.1| | DNA polymerase III subunit beta [Bacillus sp. YR335] | Bacillus sp. YR335 | NA | 2135743 | 68.9 | 68.9 | 95% | 1e-09 | 16.74% | 378 | WP\_111617261.1(scored below threshold on previous iteration) | Select seq WP\_111617261.1 for PSI blast |  |  |
  | 455Select seq ref|WP\_050368030.1| | unnamed protein product |  |  | 0 | 68.9 | 68.9 | 95% | 1e-09 | 17.43% | 378 | WP\_050368030.1(scored below threshold on previous iteration) | Select seq WP\_050368030.1 for PSI blast |  |  |
  | 456Select seq ref|WP\_168241429.1| | DNA polymerase III subunit beta [Bacillus megaterium] | Bacillus megaterium | NA | 1404 | 68.9 | 137 | 95% | 1e-09 | 17.57% | 378 | WP\_168241429.1(scored below threshold on previous iteration) | Select seq WP\_168241429.1 for PSI blast |  |  |
  | 457Select seq ref|WP\_090762336.1| | DNA polymerase III subunit beta [Bacillus sp. OK048] | Bacillus sp. OK048 | NA | 1882761 | 68.9 | 68.9 | 95% | 1e-09 | 16.67% | 378 | WP\_090762336.1(scored below threshold on previous iteration) | Select seq WP\_090762336.1 for PSI blast |  |  |
  | 458Select seq ref|WP\_033014240.1| | MULTISPECIES: DNA polymerase III subunit beta [Geobacillus] | Geobacillus | NA | 129337 | 68.9 | 68.9 | 95% | 1e-09 | 17.43% | 378 | WP\_033014240.1(scored below threshold on previous iteration) | Select seq WP\_033014240.1 for PSI blast |  |  |
  | 459Select seq gb|MBE3569485.1| | DNA polymerase III subunit beta [Bacillales bacterium] | Bacillales bacterium | NA | 1904864 | 68.9 | 68.9 | 95% | 1e-09 | 16.32% | 380 | MBE3569485.1(scored below threshold on previous iteration) | Select seq MBE3569485.1 for PSI blast |  |  |
  | 460Select seq ref|WP\_174734645.1| | DNA polymerase III subunit beta [Mesobacillus harenae] | Mesobacillus harenae | NA | 2213203 | 68.9 | 68.9 | 95% | 1e-09 | 18.41% | 378 | WP\_174734645.1(scored below threshold on previous iteration) | Select seq WP\_174734645.1 for PSI blast |  |  |
  | 461Select seq ref|WP\_121610412.1| | DNA polymerase III subunit beta [Mesobacillus foraminis] | Mesobacillus foraminis | NA | 279826 | 68.9 | 68.9 | 95% | 1e-09 | 17.15% | 378 | WP\_121610412.1(scored below threshold on previous iteration) | Select seq WP\_121610412.1 for PSI blast |  |  |
  | 462Select seq ref|WP\_060665627.1| | DNA polymerase III subunit beta [Bacillus sp. CHD6a] | Bacillus sp. CHD6a | NA | 1643452 | 68.9 | 137 | 95% | 1e-09 | 15.55% | 378 | WP\_060665627.1(scored below threshold on previous iteration) | Select seq WP\_060665627.1 for PSI blast |  |  |
  | 463Select seq ref|WP\_042464271.1| | DNA polymerase III subunit beta [Bacillus dielmoensis] | Bacillus dielmoensis | NA | 1347369 | 68.9 | 137 | 95% | 1e-09 | 17.08% | 378 | WP\_042464271.1(scored below threshold on previous iteration) | Select seq WP\_042464271.1 for PSI blast |  |  |
  | 464Select seq ref|WP\_136356338.1| | DNA polymerase III subunit beta [Bacillus sp. DSL-17] | Bacillus sp. DSL-17 | NA | 2567941 | 68.9 | 137 | 95% | 1e-09 | 18.41% | 378 | WP\_136356338.1(scored below threshold on previous iteration) | Select seq WP\_136356338.1 for PSI blast |  |  |
  | 465Select seq ref|WP\_114344907.1| | DNA polymerase III subunit beta [Bacillus sp. NFR08] | Bacillus sp. NFR08 | NA | 1566284 | 68.9 | 68.9 | 89% | 1e-09 | 15.42% | 381 | WP\_114344907.1(scored below threshold on previous iteration) | Select seq WP\_114344907.1 for PSI blast |  |  |
  | 466Select seq ref|WP\_088072069.1| | DNA polymerase III subunit beta [Bacillus luciferensis] | Bacillus luciferensis | NA | 178774 | 68.9 | 68.9 | 89% | 1e-09 | 15.86% | 381 | WP\_088072069.1(scored below threshold on previous iteration) | Select seq WP\_088072069.1 for PSI blast |  |  |
  | 467Select seq ref|WP\_095858682.1| | DNA polymerase III subunit beta [Geobacillus stearothermophilus] | Geobacillus stearothermophilus | NA | 1422 | 68.9 | 68.9 | 95% | 1e-09 | 17.43% | 378 | WP\_095858682.1(scored below threshold on previous iteration) | Select seq WP\_095858682.1 for PSI blast |  |  |
  | 468Select seq ref|WP\_132087266.1| | DNA polymerase III subunit beta [Bacillus sp. OK085] | Bacillus sp. OK085 | NA | 2135740 | 68.5 | 68.5 | 95% | 2e-09 | 17.08% | 378 | WP\_132087266.1(scored below threshold on previous iteration) | Select seq WP\_132087266.1 for PSI blast |  |  |
  | 469Select seq ref|WP\_160656499.1| | DNA polymerase III subunit beta [Salinicoccus hispanicus] | Salinicoccus hispanicus | NA | 157225 | 68.5 | 68.5 | 90% | 2e-09 | 17.67% | 377 | WP\_160656499.1(scored below threshold on previous iteration) | Select seq WP\_160656499.1 for PSI blast |  |  |
  | 470Select seq ref|WP\_046176377.1| | DNA polymerase III subunit beta [Domibacillus indicus] | Domibacillus indicus | NA | 1437523 | 68.5 | 68.5 | 95% | 2e-09 | 16.25% | 378 | WP\_046176377.1(scored below threshold on previous iteration) | Select seq WP\_046176377.1 for PSI blast |  |  |
  | 471Select seq ref|WP\_090832847.1| | MULTISPECIES: DNA polymerase III subunit beta [unclassified Bacillus] | unclassified Bacillus | NA | 185979 | 68.5 | 68.5 | 95% | 2e-09 | 17.57% | 378 | WP\_090832847.1(scored below threshold on previous iteration) | Select seq WP\_090832847.1 for PSI blast |  |  |
  | 472Select seq ref|WP\_042354589.1| | DNA polymerase III subunit beta [Bacillus rubiinfantis] | Bacillus rubiinfantis | NA | 1499680 | 68.5 | 68.5 | 95% | 2e-09 | 15.90% | 378 | WP\_042354589.1(scored below threshold on previous iteration) | Select seq WP\_042354589.1 for PSI blast |  |  |
  | 473Select seq ref|WP\_062679408.1| | DNA polymerase III subunit beta [Parageobacillus toebii] | Parageobacillus toebii | NA | 153151 | 68.5 | 68.5 | 95% | 2e-09 | 17.01% | 378 | WP\_062679408.1(scored below threshold on previous iteration) | Select seq WP\_062679408.1 for PSI blast |  |  |
  | 474Select seq ref|WP\_078378434.1| | DNA polymerase III subunit beta [Bacillus halmapalus] | Bacillus halmapalus | NA | 79882 | 68.5 | 68.5 | 95% | 2e-09 | 15.55% | 378 | WP\_078378434.1(scored below threshold on previous iteration) | Select seq WP\_078378434.1 for PSI blast |  |  |
  | 475Select seq gb|KYD21175.1| | DNA polymerase III beta subunit [Parageobacillus toebii] | Parageobacillus toebii | NA | 153151 | 68.5 | 68.5 | 95% | 2e-09 | 17.01% | 379 | KYD21175.1(scored below threshold on previous iteration) | Select seq KYD21175.1 for PSI blast |  |  |
  | 476Select seq ref|WP\_150442531.1| | DNA polymerase III subunit beta [Bacillus endozanthoxylicus] | Bacillus endozanthoxylicus | NA | 2036016 | 68.5 | 68.5 | 95% | 2e-09 | 17.50% | 378 | WP\_150442531.1(scored below threshold on previous iteration) | Select seq WP\_150442531.1 for PSI blast |  |  |
  | 477Select seq ref|WP\_066188545.1| | MULTISPECIES: DNA polymerase III subunit beta [Bacillaceae] | Bacillaceae | NA | 186817 | 68.5 | 68.5 | 95% | 2e-09 | 18.52% | 378 | WP\_066188545.1(scored below threshold on previous iteration) | Select seq WP\_066188545.1 for PSI blast |  |  |
  | 478Select seq ref|WP\_044748977.1| | DNA polymerase III subunit beta [Bacillus alveayuensis] | Bacillus alveayuensis | NA | 279215 | 68.5 | 68.5 | 91% | 2e-09 | 18.10% | 378 | WP\_044748977.1(scored below threshold on previous iteration) | Select seq WP\_044748977.1 for PSI blast |  |  |
  | 479Select seq ref|WP\_064099798.1| | DNA polymerase III subunit beta [Bacillus horikoshii] | Bacillus horikoshii | NA | 79883 | 68.5 | 68.5 | 95% | 2e-09 | 15.55% | 378 | WP\_064099798.1(scored below threshold on previous iteration) | Select seq WP\_064099798.1 for PSI blast |  |  |
  | 480Select seq ref|WP\_004888456.1| | DNA polymerase III subunit beta [Anoxybacillus flavithermus] | Anoxybacillus flavithermus | NA | 33934 | 68.5 | 68.5 | 94% | 2e-09 | 16.74% | 378 | WP\_004888456.1(scored below threshold on previous iteration) | Select seq WP\_004888456.1 for PSI blast |  |  |
  | 481Select seq ref|WP\_062186637.1| | MULTISPECIES: DNA polymerase III subunit beta [Bacillales] | Bacillales | NA | 1385 | 68.1 | 68.1 | 95% | 2e-09 | 17.50% | 378 | WP\_062186637.1(scored below threshold on previous iteration) | Select seq WP\_062186637.1 for PSI blast |  |  |
  | 482Select seq ref|WP\_009361093.1| | MULTISPECIES: DNA polymerase III subunit beta [Anoxybacillus] | Anoxybacillus | NA | 150247 | 68.1 | 68.1 | 94% | 2e-09 | 17.15% | 378 | WP\_009361093.1(scored below threshold on previous iteration) | Select seq WP\_009361093.1 for PSI blast |  |  |
  | 483Select seq ref|WP\_031546774.1| | DNA polymerase III subunit beta [Salinicoccus luteus] | Salinicoccus luteus | NA | 367840 | 68.1 | 68.1 | 89% | 2e-09 | 16.16% | 377 | WP\_031546774.1(scored below threshold on previous iteration) | Select seq WP\_031546774.1 for PSI blast |  |  |
  | 484Select seq ref|WP\_055440879.1| | DNA polymerase III subunit beta [Anoxybacillus suryakundensis] | Anoxybacillus suryakundensis | NA | 1325335 | 68.1 | 68.1 | 94% | 2e-09 | 17.15% | 378 | WP\_055440879.1(scored below threshold on previous iteration) | Select seq WP\_055440879.1 for PSI blast |  |  |
  | 485Select seq ref|WP\_076559704.1| | DNA polymerase III subunit beta [Salimicrobium flavidum] | Salimicrobium flavidum | NA | 570947 | 68.1 | 68.1 | 95% | 2e-09 | 17.21% | 379 | WP\_076559704.1(scored below threshold on previous iteration) | Select seq WP\_076559704.1 for PSI blast |  |  |
  | 486Select seq ref|WP\_011124156.1| | MULTISPECIES: DNA polymerase III subunit beta [Prochlorococcus] | Prochlorococcus | NA | 1218 | 68.1 | 68.1 | 96% | 2e-09 | 15.48% | 385 | WP\_011124156.1(scored below threshold on previous iteration) | Select seq WP\_011124156.1 for PSI blast |  |  |
  | 487Select seq ref|WP\_020007131.1| | DNA polymerase III subunit beta [Salinicoccus albus] | Salinicoccus albus | NA | 418756 | 68.1 | 136 | 89% | 2e-09 | 14.85% | 377 | WP\_020007131.1(scored below threshold on previous iteration) | Select seq WP\_020007131.1 for PSI blast |  |  |
  | 488Select seq ref|WP\_179598994.1| | DNA polymerase III subunit beta [Neobacillus niacini] | Neobacillus niacini | NA | 86668 | 68.1 | 68.1 | 95% | 2e-09 | 16.67% | 378 | WP\_179598994.1(scored below threshold on previous iteration) | Select seq WP\_179598994.1 for PSI blast |  |  |
  | 489Select seq ref|WP\_146550421.1| | MULTISPECIES: DNA polymerase III subunit beta [Rummeliibacillus] | Rummeliibacillus | NA | 648802 | 68.1 | 68.1 | 95% | 2e-09 | 15.55% | 378 | WP\_146550421.1(scored below threshold on previous iteration) | Select seq WP\_146550421.1 for PSI blast |  |  |
  | 490Select seq ref|WP\_102693423.1| | DNA polymerase III subunit beta [Rummeliibacillus pycnus] | Rummeliibacillus pycnus | NA | 101070 | 68.1 | 68.1 | 93% | 2e-09 | 16.24% | 378 | WP\_102693423.1(scored below threshold on previous iteration) | Select seq WP\_102693423.1 for PSI blast |  |  |
  | 491Select seq ref|WP\_012748781.1| | MULTISPECIES: DNA polymerase III subunit beta [unclassified Geobacillus] | unclassified Geobacillus | NA | 2642459 | 68.1 | 68.1 | 95% | 2e-09 | 17.43% | 378 | WP\_012748781.1(scored below threshold on previous iteration) | Select seq WP\_012748781.1 for PSI blast |  |  |
  | 492Select seq ref|WP\_124051718.1| | DNA polymerase III subunit beta [Bacillus endophyticus] | Bacillus endophyticus | NA | 135735 | 68.1 | 68.1 | 95% | 2e-09 | 16.74% | 378 | WP\_124051718.1(scored below threshold on previous iteration) | Select seq WP\_124051718.1 for PSI blast |  |  |
  | 493Select seq ref|WP\_044749249.1| | DNA polymerase III subunit beta [Bacillus alveayuensis] | Bacillus alveayuensis | NA | 279215 | 68.1 | 68.1 | 95% | 2e-09 | 19.17% | 378 | WP\_044749249.1(scored below threshold on previous iteration) | Select seq WP\_044749249.1 for PSI blast |  |  |
  | 494Select seq ref|WP\_075687039.1| | DNA polymerase III subunit beta [Bacillus sp. MRMR6] | Bacillus sp. MRMR6 | NA | 1928617 | 68.1 | 68.1 | 95% | 2e-09 | 16.67% | 378 | WP\_075687039.1(scored below threshold on previous iteration) | Select seq WP\_075687039.1 for PSI blast |  |  |
  | 495Select seq ref|WP\_026568047.1| | DNA polymerase III subunit beta [Bacillus sp. UNC41MFS5] | Bacillus sp. UNC41MFS5 | NA | 1449046 | 68.1 | 68.1 | 95% | 2e-09 | 17.15% | 378 | WP\_026568047.1(scored below threshold on previous iteration) | Select seq WP\_026568047.1 for PSI blast |  |  |
  | 496Select seq ref|WP\_006321151.1| | DNA polymerase III subunit beta [Anoxybacillus flavithermus] | Anoxybacillus flavithermus | NA | 33934 | 68.1 | 68.1 | 94% | 3e-09 | 16.74% | 378 | WP\_006321151.1(scored below threshold on previous iteration) | Select seq WP\_006321151.1 for PSI blast |  |  |
  | 497Select seq ref|WP\_077429081.1| | MULTISPECIES: DNA polymerase III subunit beta [Bacillaceae] | Bacillaceae | NA | 186817 | 68.1 | 68.1 | 94% | 3e-09 | 16.74% | 378 | WP\_077429081.1(scored below threshold on previous iteration) | Select seq WP\_077429081.1 for PSI blast |  |  |
  | 498Select seq ref|WP\_183242679.1| | DNA polymerase III subunit beta [Anoxybacillus mongoliensis] | Anoxybacillus mongoliensis | NA | 452565 | 67.7 | 67.7 | 94% | 3e-09 | 17.15% | 378 | WP\_183242679.1(scored below threshold on previous iteration) | Select seq WP\_183242679.1 for PSI blast |  |  |
  | 499Select seq ref|WP\_136381724.1| | DNA polymerase III subunit beta [Bacillus timonensis] | Bacillus timonensis | NA | 1033734 | 67.7 | 67.7 | 89% | 3e-09 | 17.62% | 378 | WP\_136381724.1(scored below threshold on previous iteration) | Select seq WP\_136381724.1 for PSI blast |  |  |
  | 500Select seq ref|WP\_003397595.1| | DNA polymerase III subunit beta [Anoxybacillus flavithermus] | Anoxybacillus flavithermus | NA | 33934 | 67.7 | 67.7 | 94% | 3e-09 | 16.74% | 378 | WP\_003397595.1(scored below threshold on previous iteration) | Select seq WP\_003397595.1 for PSI blast |  |  |

  Run PSI-BLAST Iteration 4 with max number of sequences

  Run

  Sequences with E-value WORSE than threshold

  - select all
  - 0 sequences selected
  - PSI-BLAST iteration 3

  Sequences with E-value WORSE than threshold

  | Select for downloading or viewing reports | Description | Scientific Name | Common Name | Taxid |  |
  | --- | --- | --- | --- | --- | --- |
