# Supplementary material for: Evolution and origin of sliding clamp in bacteria, archaea and eukarya
Source: PLoS One. 2021 Aug 11;16(8):e0241093. doi: 10.1371/journal.pone.0241093 (PMC8357120; doi:10.1371/journal.pone.0241093)
Supplement: Attachment — Submitted filename: Response to Reviewers.docx [file pone.0241093.s009.docx]

Rebuttal Letter:

Jan 29, 2021

To,

The Reviewers,

Plos One Journal

Sub: Rebuttal Letter

We would like to extend our gratitude to both our reviewers for providing their valuable suggestions to make our paper more informative. Following the recommendations from both our reviewers, the following contents were added to the manuscript.

- The procedure for second iteration of PSI-Blast was provided.
- Full Blast Results of the Second Iteration was provided as a hyperlink.
- Domain Information (Secondary Structures including helix, turns, sheets) was provided with the alignment to help the readers understand the conservation of secondary structures across the domains of life.
- A tree of life was constructed using sequences from all three domains of life.
- From the multiple sequence alignment files of sliding clamp proteins of each domain, the residues which were conserved in the respective domain were identified. Furthermore, the multiple alignment of sequences across all domains of life was generated, and the residues which were conserved across all domains were identified and compared with the former.
- The evolutionary history of *Sulfolobus solfataricus* was explained with reference to Sulfolobales and Desulfurococcales.

Following the suggestion by the reviewers, the paper was sent for copyediting. The paper was copyedited by Dr. Anusha Thapa. Furthermore, the contents of the manuscript were formatted as per the PLOS One Author Guidelines.

Sincerely,

The Authors
