# Supplementary material for: Evolution and origin of sliding clamp in bacteria, archaea and eukarya
Source: PLoS One. 2021 Aug 11;16(8):e0241093. doi: 10.1371/journal.pone.0241093 (PMC8357120; doi:10.1371/journal.pone.0241093)
Supplement: Attachment — Submitted filename: Response to Reviewers.docx [file pone.0241093.s010.docx]

Rebuttal Letter:

April 14, 2021

To,

The Reviewers,

Plos One Journal

Sub: Rebuttal Letter

We would like to extend our gratitude to both our reviewers for providing their valuable suggestions to make our paper more informative. Following the recommendations from both our reviewers, the following contents were added to the manuscript. The line numbers mentioned here are from Revised Manuscript with Track Changes file. The revised manuscript also contains the figures for easier access to the contents. However, we have revised our final manucript (Manuscript.docx) as per the formatting requirement of the PLOS One Journal.

- The procedure for second iteration of PSI-Blast was provided. (Line 83-88)
- Full Blast Results of the Second Iteration was provided as a hyperlink. (Line 100-109). Separate hyperlink files are attached with the manuscript in the earlier revision.
- Domain Information (Secondary Structures including helix, turns, sheets) was provided with the alignment to help the readers understand the conservation of secondary structures across the domains of life. (Figures 2 (A) and (D) and Line Number 325-330). It was observed that Domain 2 and 3 of beta clamp align with the two domains of PCNA. Study of secondary structures in the aligned domains in both beta clamp and PCNA revealed that secondary structures in both proteins are almost similar and conserved in all regions. Preservation of structure and function in two proteins could result because of preservation of secondary structure.
- A tree of life was constructed using sequences from all three domains of life. [Tree Construction Method: Line 141-148, Tree Discussion: Line 492-507 and Tree: Fig 6]. It can be inferred from the tree that sliding clamp proteins have sufficiently diverged across different domains of life remaining fairly conserved in its own domain.
- From the multiple sequence alignment files of sliding clamp proteins of each domain, the residues which were conserved in the respective domain were identified. Furthermore, the multiple alignment of sequences across all domains of life was generated, and the residues which were conserved across all domains were identified and compared with the former. (Line Number 509-530) and Supplementary Information S2, S3, S4 and S5. It was observed only the terminal residues were conserved across all domains of life. Thus, those terminal residues could be studied further in relation to structural and functional property of sliding clamps. Although inter-domain residue conservation was low, we could observe that secondary structure was highly conserved in the two proteins. Thus, the proteins have diverged sufficiently enough to show very less sequence identity, whilst preserving the secondary structures.
- The issue of interdomain sequence conservation not being the same as interdomain sequence conservation is addressed (Line number 532-534).
- The evolutionary history of *Sulfolobus solfataricus* was explained with reference to Sulfolobales and Desulfurococcales. (Line Number 439-451).

Following the suggestion by the reviewers, the paper was sent for copyediting. The paper was copyedited by Dr. Anusa Thapa. Furthermore, the contents of the manuscript were formatted as per the PLOS One Author Guidelines.

Sincerely,

The Authors
