# Supplementary material for: Evolution and origin of sliding clamp in bacteria, archaea and eukarya
Source: PLoS One. 2021 Aug 11;16(8):e0241093. doi: 10.1371/journal.pone.0241093 (PMC8357120; doi:10.1371/journal.pone.0241093)
Supplement: Attachment — Submitted filename: Response to Reviewers.docx [file pone.0241093.s011.docx]

Rebuttal Letter:

June 27, 2021

To,

The Reviewers,

Plos One Journal

Sub: Rebuttal Letter

Following the issue about inter and intra domain conservation, we did a thorough review on the multiple alignments from different angles. Last time, we included only a selected few species for inter-domain alignment. In correction, we included all species (from bacteria, archaea and eukarya domain under study) for inter-domain alignment. Among 10 conserved residues in inter-domain alignment, 8 were conserved in Eukarya, 7 in Archaea and 6 in Bacteria. The main reason for this was the use of low conservation threshold.

The notion that intradomain conservation must precede inter-domain conservation holds true when the conservation level is very high. In our case, the threshold conservation level is set comparatively low to identify many important residues that might serve some functionality in sliding clamp proteins. We have three domains of life: bacteria, archaea and eukarya in our inter-domain multiple alignment. Since our conservation threshold is around 60%, it is possible that some of the residues might be conserved in only two of the three domains, but still identify as conserved in inter-domain alignment. A multiple alignment between archaea and eukarya (all PCNAs) was done, and it was identified that all residues of concern (observed conserved in the inter-domain alignment, but unconserved in bacteria, archaea and eukarya) were conserved in multiple alignment of PCNAs. This issue, along with its biological significance is addressed in line no 530-543 of revised manuscript with track changes.

Finally, we would like to thank the reviewers for thorough review of our paper, and all their recommendations. Hope it meets all requirements.

Thank You.

Sincerely,

The Authors
